# Molecular Taxonomy of Sporadic Amyotrophic Lateral Sclerosis Using Disease-Associated Genes

**DOI:** 10.3389/fneur.2017.00152

**Published:** 2017-04-19

**Authors:** Giovanna Morello, Antonio Gianmaria Spampinato, Sebastiano Cavallaro

**Affiliations:** ^1^Institute of Neurological Sciences, Italian National Research Council, Catania, Italy

**Keywords:** amyotrophic lateral sclerosis, genomics, expression profiling, molecular classification of disease subtypes, gene ontology analysis

## Abstract

Amyotrophic lateral sclerosis (ALS) is a fatal neurodegenerative disease characterized by selective loss of upper and lower motor neurons. Despite intensive research, the origin and progression of ALS remain largely unknown, suggesting that the traditional clinical diagnosis and treatment strategies might not be adequate to completely capture the molecular complexity underlying the disease. In our previous work, comprehensive genomic profiling of 41 motor cortex samples enabled to discriminate control from sporadic ALS patients and segregated these latter into two distinct subgroups, each associated with different deregulated genes and pathways. Interestingly, some deregulated genes in sporadic ALS were previously associated with familiar ALS, indicating shared pathogenic mechanisms between the two forms of disease. In this, we performed cluster analysis on the same whole-genome expression profiles using a restricted (203) subset of genes extensively implicated in monogenic forms of ALS. Surprisingly, this short and unbiased gene list was sufficiently representative to allow the accurate separation of SALS patients from controls and the stratification of SALS patients into two molecularly distinct subgroups. Overall, our findings support the existence of a molecular taxonomy for ALS and represent a further step toward the establishment of a molecular-based diagnosis and patient-tailored therapies.

## Introduction

Amyotrophic lateral sclerosis (ALS) is a genetically and clinically heterogeneous neurodegenerative disease characterized by progressive muscular paralysis reflecting degeneration of motor neurons in both the motor cortex and the spinal cord (1). Although ~10% of cases have a clear family history of ALS (FALS), the remaining 90% is considered sporadic (SALS) and probably associated with a polygenic and multifactorial etiology. Despite intensive research, the origin and progression of ALS remain largely unknown, suggesting that the traditional clinical diagnosis and treatment strategies might not be adequate to completely capture the molecular complexity underlying disease (2).

In the last few years, significant advances toward understanding the complex architecture of ALS have been achieved, thanks to the development of high-throughput profiling methods that have started to decipher genes and pathways involved in the disease pathogenesis (3–6). However, the analysis was often limited to small sample sizes, thus not allowing a transcriptionally driven classification of ALS subtypes.

In a previous work, for the first time, we demonstrated the remarkable power of genomic profiling to characterize and explore the hidden biological and molecular heterogeneity of SALS, enabling the identification of etiopathogenic mechanisms and potential therapeutic targets that were not put in evidence by considering SALS pathology as a single entity (7). In particular, we used an unsupervised hierarchical algorithm to cluster 41 motor cortex samples from control and SALS patients on the basis of their similarities measured over the most “hypervariable genes” (9,646 genes with a SD > 1.5). The transcriptome profiles enabled to differentiate controls from SALS patients and clearly distinguished two SALS subgroups, each associated with differentially expressed genes and pathways. Interestingly, some of deregulated genes in SALS patients were previously associated with FALS, indicating that pathological events associated with both forms of the disease may converge on common signaling pathways.

In this study, we used the full list of genes that have been implicated, up-to-date, in the causation and/or susceptibility of monogenic forms of ALS, investigating their expression patterns in SALS patients and evaluating their effectiveness in confirming the existence of a molecular taxonomy for ALS. Surprisingly, this short and unbiased list of genes was sufficiently representative to accurately separate SALS patients from controls and confirmed the presence of two molecularly distinct SALS subgroups.

## Materials and Methods

### Data Sources and Gene Selection

For this study, we referred to our previously described transcriptome data set (7), available at ArrayExpress^1^ with the accession number E-MTAB-2325. Briefly, this data set consists of mRNA expression profiles of 41,059 genes from 41 motor cortex samples of SALS and control subjects hybridized onto 4 × 44K Whole Human Genome Oligo expression microarrays (Agilent Technologies). Once obtained, raw intensity values were thresholded to 1, log2-transformed, normalized, and baselined to the median of all samples by using GeneSpring GX (Agilent Technologies). In addition, the fold change (FC) of each gene was calculated between SALS patients and individual controls. A detailed description of the study design, subject characteristics, and experimental procedures is reported in the original publication (7).

In the present work, we reanalyzed our genomic data focusing on a list of 203 genes (here referred as *SGALS*) that were reported to significantly influence ALS susceptibility (Table S1 in Supplementary Material). The *SGALS* gene set was obtained by integrating data from various source databases, including ALSoD^2^ and ALSGene.^3^ These are two freely available online bioinformatics repositories that provide systematic and in-depth qualitative and quantitative overviews of ALS genetic research, embracing known and putative risk factors, disease-causing genes, and other related genetic regions affected by different types of mutations (8).

### Clustering Analysis and Statistical Investigation of SGALS Gene Expression Data

Once processed, normalized expression data were imported into R and filtered for the set of probes mapping to *SGALS* genes. The resulting expression profiles were clustered for visualization by using a Pearson centered correlation as a distance metric with average linkage rules in the tree building algorithm. In particular, unsupervised hierarchical cluster analysis was performed using the *hclust* function in R, while heatmaps were rendered using *heatmap.3* package from CRAN (9).

**Figure 1:**
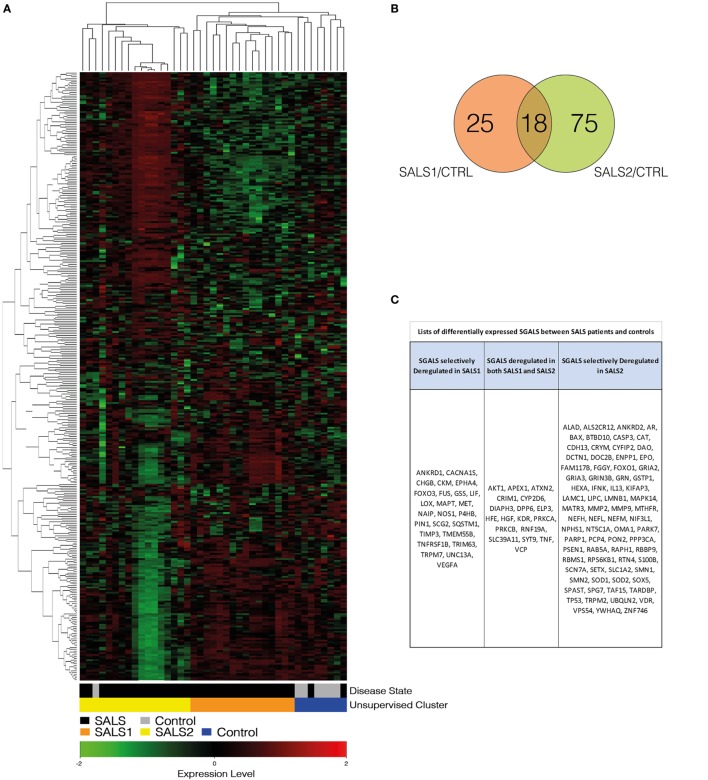
**Unsupervised hierarchical clustering of control and SALS patients**. **(A)** Unsupervised hierarchical clustering was used to cluster control and SALS patients on the basis of the similarity in their *SGALS* expression profiles. In this two-dimensional presentation, each column represents a motor cortex from control or SALS patients, while each row represents a single gene probe. As shown in the color bar, red indicates upregulation, green downregulation, and black no change. In the dendrograms shown (left and top), the length and the subdivision of the branches display the relatedness of the expression of the probes and the motor cortex (top). **(B)** Venn diagrams of differentially expressed *SGALS* in SALS1 and SALS2 *versus* controls (Tables S2 and S3 in Supplementary Material). **(C)** Lists of the *SGALS* differentially expressed in each of the two SALS patient subsets in comparison with controls, identifying those commonly affected in both classes and those selectively affected in a single patient subgroup. Details are provided in Tables S1–S3 in Supplementary Material.

**Figure 2:**
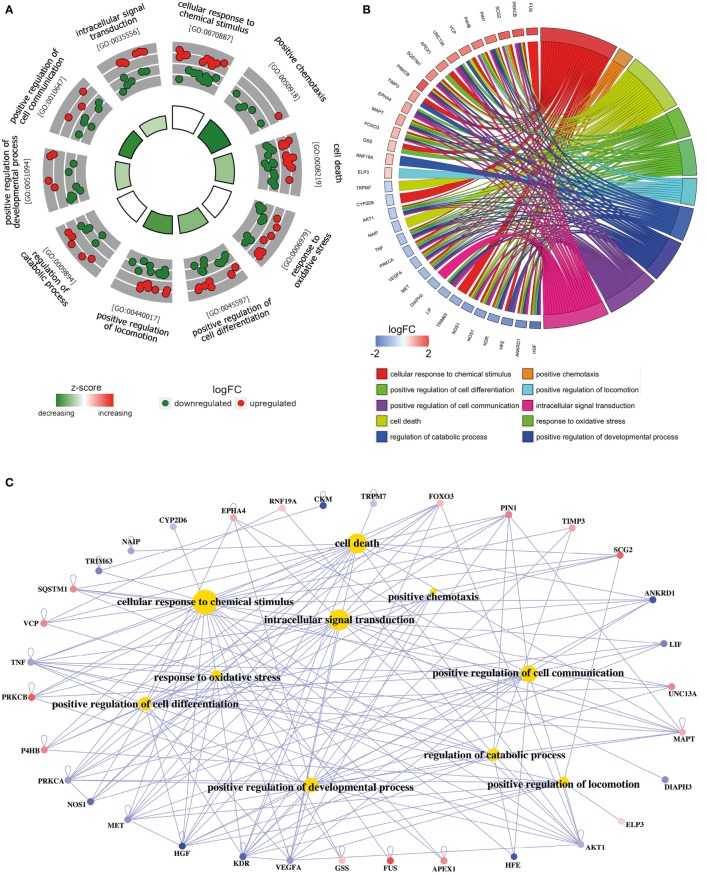
**Functional analysis of deregulated *SGALS* in SALS1**. **(A)** The outer circle shows a scatter plot of the expression levels (logFC) for SALS1-related differentially expressed SGALS in each enriched gene ontology (GO) term: red circles indicate upregulation and blue ones downregulation. The inner ring is a bar plot where the height of the bar indicates the significance of GO terms (log10-adjusted *P* value), and color corresponds to the z-score: green, decreased; red, increased; and white, unchanged. **(B)** The plot shows the relationship between statistically significant *SGALS* in SALS1 and their related GO terms; for each gene, the logFC value is shown by red/blue colored rectangles. **(C)** Gene-concept networks by GO analysis for differentially expressed *SGALS* in SALS1, using *GeneAnswers* Package. Yellow hubs correspond to the most enriched GO terms; red and blue nodes represent downregulation and upregulation of *SGALS* in SALS1, respectively. The size of each yellow hub is proportional to the statistical significance of the identified genes in corresponding GO categories. Details are provided in Table S4 in Supplementary Material.

**Figure 3:**
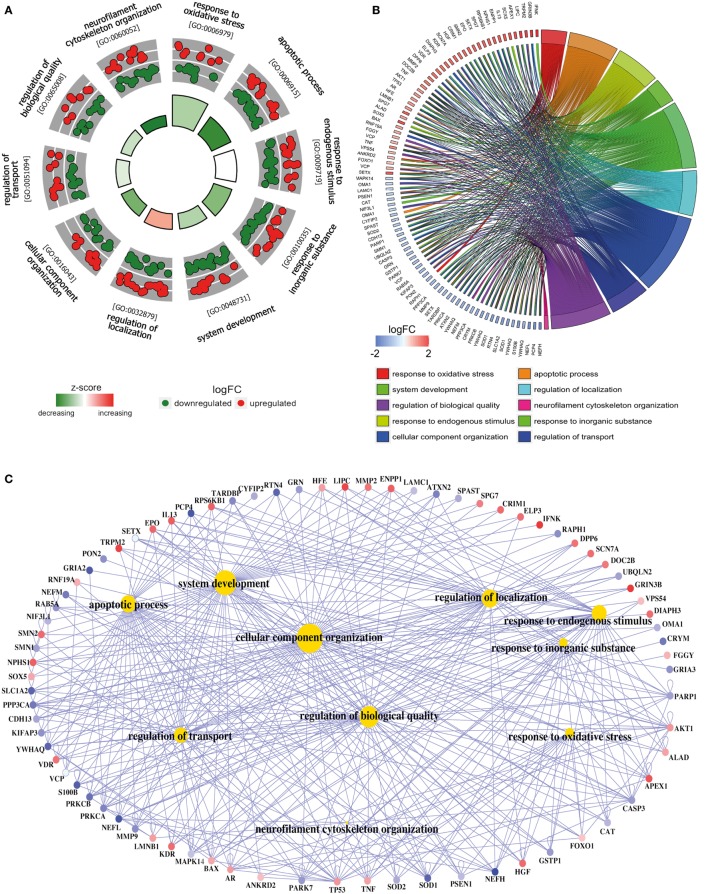
**Functional analysis of deregulated *SGALS* in SALS2**. **(A)** The outer circle shows a scatter plot of the expression levels (logFC) for SALS2-related differentially expressed SGALS in each enriched gene ontology (GO) term: red circles indicate upregulation and blue ones downregulation. The inner ring is a bar plot where the height of the bar indicates the significance of GO terms (log10-adjusted *P* value), and color corresponds to the z-score: green, decreased; red, increased; and white, unchanged. **(B)** The plot shows the relationship between statistically significant *SGALS* in SALS2 and their related GO terms; for each gene, the logFC value is shown by red/blue colored rectangles. **(C)** Gene-concept networks by GO analysis for differentially expressed *SGALS* in SALS2, using *GeneAnswers* Package. Yellow hubs correspond to the most enriched GO terms; red and blue nodes represent downregulation and upregulation of *SGALS* in SALS2, respectively. The size of each yellow hub is proportional to the statistical significance of the identified genes in corresponding GO categories. Details are provided in Table S5 in Supplementary Material.

To assess the statistical significance of *SGALS* expression changes in SALS patients compared to healthy controls, statistical analysis was performed by using GeneSpring GX software package (version 14.5; Agilent Technologies). In particular, we used a one-way analysis of variance with a Benjamini–Hochberg’s false discovery rate (FDR)-controlling procedure followed by a *post hoc* Tukey’s test. Differentially expressed genes with an FDR-adjusted *P* ≤ 0.05 and fold changes (FC) ≥1.5 were deemed as significant. Moreover, to achieve a precise estimation of the differential gene expression, multiple probes per gene were used. For hierarchical clustering (Figure 1A) and gene ontology (GO) enrichment analysis (Figures 2 and 3), relationships among genes are represented. When multiple probes corresponded to the same gene, the expression changes of probe sets were averaged.

### Functional Characterization of Differentially Expressed SGALS Genes

To interpret the biological significance of gene expression changes, we performed a functional characterization of *SGALS* differentially expressed in both SALS subgroups. In particular, GO enrichment analysis of biological process (BP) was performed by using a hypergeometric test in the R Bioconductor package *GeneAnswers* (10). *P* values were then adjusted by using multiple testing adjustments with an FDR <0.05 as the significance threshold. In addition, to reduce potential errors due to the use of preselected gene sets (11), we also performed a “control” GO enrichment analysis on both the entire list of differentially expressed *SGALS* in the two SALS subgroups (Table S6 in Supplementary Material) and two randomly selected subsets of *SGALS* (*n* = 100) (Tables S7 and S8 in Supplementary Material).

To add quantitative molecular information to GO terms of interest, we used *GOCircle* and *GOChord* plot functions of *GOplot* R package (12), which permit to incorporate data derived from expression level measurements with those obtained from the functional annotation enrichment analysis. In addition, to provide a readable graphic representation of the complex relationship between *SGALS* and relative GO terms, the “concept-and-gene network” was constructed by Bioconductor package *GeneAnswers* (13).

## Results and Discussion

In this study, we reanalyzed our transcriptome data set (7), by focusing on a restricted subset of genes, previously identified as causative or susceptibility genes in ALS (here referred as *SGALS*) (Table S1 in Supplementary Material). Hierarchical clustering analysis for this minimal and unbiased gene panel resulted highly informative in supporting the existence of prominent molecular signatures capable of accurately separating controls from SALS patients and subdividing these latter into two greatly divergent subgroups (SALS1 and SALS2) (Figure 1A). These findings demonstrate that *SGALS* may not only cause the monogenic form of ALS but also act in the sporadic form, supporting the existence of shared pathogenic mechanisms between the two forms of the disease.

Next, a statistical significance test was performed to define genes that were differentially expressed between SALS patients and controls. Our analysis demonstrated that 118 of 203 *SGALS* were differentially deregulated in SALS patients and, interestingly, the majority of these genes were cluster specific (25 in SALS1 and 75 in SALS2), suggestive of a great divergence of SALS patient subgroups at the molecular level (Figures 1B,C; Tables S2 and S3 in Supplementary Material).

To provide a biologically meaningful interpretation of our results, we further performed a GO enrichment analysis for detecting significantly overrepresented GO categories (BP) among the set of differentially expressed *SGALS*, by using the TopGO package in R (12). The enrichment of GO annotation terms revealed that, although some processes were commonly deregulated in both SALS subgroups (e.g., *regulation of apoptosis* and *cellular response to external/endogenous stimulus*), the majority of them were subgroup specific (Figures 2 and 3; Tables S4 and S5 in Supplementary Material). In particular, the most representative functional processes in SALS1 were annotated as involved in the *regulation of chemotaxis, regulation of cellular communication/differentiation*, and *intracellular signal transduction* (Figures 2A,B; Table S4 in Supplementary Material). Deregulated genes in SALS2 were associated with *response to oxidative stress, cytoskeleton organization, regulation of transport*, and *regulation of cellular localization* (Figures 3A,B; Table S5 in Supplementary Material). While these findings are consistent with previous evidence about the crucial role of these pathogenetic mechanisms in ALS (4, 7, 14–18), they suggest for the first time the differential involvement of these mechanisms in specific subsets of ALS patients, offering an useful starting point for the further development of personalized diagnostics and targeted therapies.

The existing interconnections between multiple differentially expressed *SGALS* and their related GO BPs were made more evident thanks to the generation of SALS subgroup-specific “Concept-and-Gene Networks” (Figures 2C and 3C). The functional interpretation of these GO-based genetic networks not only highlights the possibility that *SGALS* can exert their pathogenic effects *via* different multifactorial combinations but mostly provides important insights into candidate driver genes that may truly exert a fundamental influence on ALS (e.g., genes with a high number of connections/edges in the network). Among these, an interesting example is represented by *TNF*, a gene encoding a pro-inflammatory factor whose expression levels were found significantly elevated in serum of ALS patients (19). In agreement with these findings, we observed deregulated expression of *TNF* in both clusters of SALS patients, suggesting its potential role as a marker in SALS. *NEFH* is another attractive candidate gene encoding a neurofilament protein that plays an important role in axonal and dendritic intracellular transport and in the maintenance of neuronal caliber. Consistent with the observation that the decreased activity of NEFH was observed only in a small group of ALS patients (20, 21), we found low transcriptional levels of this gene in SALS2 patients, suggesting that this protein may represent a susceptibility factor for specific subgroups of SALS patients.

In summary, the *SGALS* panel was sufficiently representative to accurately differentiate SALS patients from controls, suggesting that sporadic and monogenic forms of ALS share common etiopathogenic mechanisms. In addition, the same unbiased gene list confirmed the stratification of SALS patients into two molecularly distinct subgroups associated with a good number of cluster-specific candidate genes and unique biological features. Overall, our findings indicate the existence of a molecular taxonomy for ALS and represent a further step toward the establishment of a molecular-based diagnosis and patient-tailored therapies.

## Author Contributions

GM and AS wrote the manuscript. AS performed bioinformatics analyses. SC conceived, directed, and supervised the project. All authors have read and approved the final version of this manuscript and agreed to be accountable for all aspects of the work.

## Conflict of Interest Statement

The authors declare that the research was conducted in the absence of any commercial or financial relationships that could be construed as a potential conflict of interest.

## Footnotes

^1^http://www.ebi.ac.uk/arrayexpress/.

^2^http://alsod.iop.kcl.ac.uk/.

^3^http://www.alsgene.org.
