# Supplementary material for: Molecular Taxonomy of Sporadic Amyotrophic Lateral Sclerosis Using Disease-Associated Genes
Source: Front Neurol. 2017 Apr 19;8:152. doi: 10.3389/fneur.2017.00152 (PMC5395696; doi:10.3389/fneur.2017.00152)
Supplement: Supplementary file 1 [file Table_1.PDF]

Supplementary Table 1. List of SGALS genes and related probe IDs.

ID	EntrezGeneID	GENE_SYMBOL	GENE_NAME
A_23_P400078	4524	MTHFR	5,10-methylenetetrahydrofolate reductase (NADPH)""
A_24_P6552	1804	DPP6	dipeptidyl-peptidase 6
A_23_P426292	1432	MAPK14	mitogen-activated protein kinase 14
A_23_P88470	54822	TRPM7	transient receptor potential cation channel, subfamily M, member 7""
A_24_P154948	2617	GARS	glycyl-tRNA synthetase
A_23_P155404	27255	CNTN6	contactin 6
A_24_P472007	23025	UNC13A	unc-13 homolog A (C. elegans)
A_23_P127475	9973	CCS	copper chaperone for superoxide dismutase
A_23_P91293	9217	VAPB	VAMP (vesicle-associated membrane protein)-associated protein B and C
A_23_P49708	2896	GRN	granulin
A_24_P50759	7124	TNF	tumor necrosis factor (TNF superfamily, member 2)""
A_24_P928061	27185	DISC1	disrupted in schizophrenia 1
A_24_P213478	57556	SEMA6A	sema domain, transmembrane domain (TM), and cytoplasmic domain, (semaphorin) 6A""
A_23_P215897	25897	RNF19A	ring finger protein 19A
A_23_P207699	4137	MAPT	microtubule-associated protein tau
A_23_P208706	581	BAX	BCL2-associated X protein
A_23_P108180	116444	GRIN3B	glutamate receptor, ionotropic, N-methyl-D-aspartate 3B
A_23_P31399	5445	PON2	paraoxonase 2
A_23_P40174	4318	MMP9	matrix metallopeptidase 9 (gelatinase B, 92kDa gelatinase, 92kDa type IV collagenase)""
A_24_P151464	6647	SOD1	superoxide dismutase 1, soluble""
A_23_P205686	5663	PSEN1	presenilin 1
A_23_P501007	2202	EFEMP1	EGF-containing fibulin-like extracellular matrix protein 1
A_24_P916496	5578	PRKCA	protein kinase C, alpha""
A_24_P54174	7133	TNFRSF1B	tumor necrosis factor receptor superfamily, member 1B""
A_23_P82814	114907	FBXO32	F-box protein 32
A_24_P930507		ATXN2	ataxin 2
A_24_P941988	6683	SPAST	spastin
A_23_P202658	2950	GSTP1	glutathione S-transferase pi 1
A_24_P111996	3077	HFE	hemochromatosis
A_23_P93787	3082	HGF	hepatocyte growth factor (hepapoietin A; scatter factor)
A_23_P349343	130540	ALS2CR12	amyotrophic lateral sclerosis 2 (juvenile) chromosome region, candidate 12""
A_23_P26810	7157	TP53	tumor protein p53
A_23_P2960	207	AKT1	v-akt murine thymoma viral oncogene homolog 1
A_23_P62920	22920	KIFAP3	kinesin-associated protein 3
A_23_P89431	6347	CCL2	chemokine (C-C motif) ligand 2
A_23_P212545	5868	RAB5A	RAB5A, member RAS oncogene family""
A_23_P164650	348	APOE	apolipoprotein E
A_23_P103522	6402	SELL	selectin L
A_23_P428738	283	ANG	angiogenin, ribonuclease, RNase A family, 5""
A_24_P71973	3791	KDR	kinase insert domain receptor (a type III receptor tyrosine kinase)
A_23_P205531	6038	RNASE4	ribonuclease, RNase A family, 4""
A_23_P115261	183	AGT	angiotensinogen (serpin peptidase inhibitor, clade A, member 8)""
A_23_P104555	26287	ANKRD2	ankyrin repeat domain 2 (stretch responsive muscle)
A_23_P204791	4842	NOS1	nitric oxide synthase 1 (neuronal)
A_23_P342891	4741	NEFM	neurofilament, medium polypeptide""
A_23_P340728	5663	PSEN1	presenilin 1
A_23_P344392	4968	OGG1	8-oxoguanine DNA glycosylase
A_24_P27977	7226	TRPM2	transient receptor potential cation channel, subfamily M, member 2""
A_23_P81805	7422	VEGFA	vascular endothelial growth factor A
A_23_P324633	203228	C9orf72	chromosome 9 open reading frame 72
A_32_P102062	2309	FOXO3	forkhead box O3
A_23_P168598	5444	PON1	paraoxonase 1
A_23_P382584	1114	CHGB	chromogranin B (secretogranin 1)
A_24_P322756	7226	TRPM2	transient receptor potential cation channel, subfamily M, member 2""
A_23_P405873	203228	C9orf72	chromosome 9 open reading frame 72
A_23_P114983	84676	TRIM63	tripartite motif-containing 63
A_23_P359245	4233	MET	met proto-oncogene (hepatocyte growth factor receptor)
A_23_P324278	210	ALAD	aminolevulinate, delta-, dehydratase""
A_24_P940921	23064	SETX	senataxin
A_23_P90944	6332	SCN7A	sodium channel, voltage-gated, type VII, alpha""
A_23_P119899	2043	EPHA4	EPH receptor A4
A_24_P314159	351	APP	amyloid beta (A4) precursor protein
A_23_P416705	259173	ALS2CL	ALS2 C-terminal like
A_23_P342009	3077	HFE	hemochromatosis
A_24_P397566	1432	MAPK14	mitogen-activated protein kinase 14
A_23_P28730	57473	ZNF512B	zinc finger protein 512B
A_23_P113111	367	AR	androgen receptor
A_23_P114783	142	PARP1	poly (ADP-ribose) polymerase 1

A_23_P201628	3915	LAMC1	laminin, gamma 1 (formerly LAMB2)""
A_24_P59111		DPP6	dipeptidyl-peptidase 6
A_24_P50829	54822	TRPM7	transient receptor potential cation channel, subfamily M, member 7""
A_23_P122216	4015	LOX	lysyl oxidase
A_32_P172339	2891	GRIA2	glutamate receptor, ionotropic, AMPA 2""
A_23_P346309	581	BAX	BCL2-associated X protein
A_23_P139722	7132	TNFRSF1A	tumor necrosis factor receptor superfamily, member 1A""
A_24_P914688	2891	GRIA2	glutamate receptor, ionotropic, AMPA 2""
A_32_P76247	3315	HSPB1	heat shock 27kDa protein 1
A_32_P192376	5167	ENPP1	ectonucleotide pyrophosphatase/phosphodiesterase 1
A_23_P92410	836	CASP3	caspase 3, apoptosis-related cysteine peptidase""
A_32_P64919	81624	DIAPH3	diaphanous homolog 3 (Drosophila)
A_23_P162589	7421	VDR	vitamin D (1,25- dihydroxyvitamin D3) receptor""
A_24_P934355	4842	NOS1	nitric oxide synthase 1 (neuronal)
A_24_P65616	5817	PVR	poliovirus receptor
A_23_P51105	51232	CRIM1	cysteine rich transmembrane BMP regulator 1 (chordin-like)
A_23_P57856	604	BCL6	B-cell CLL/lymphoma 6
A_24_P283221	4671	NAIP	NLR family, apoptosis inhibitory protein""
A_24_P364363	7132	TNFRSF1A	tumor necrosis factor receptor superfamily, member 1A""
A_23_P83045	7415	VCP	valosin-containing protein
A_23_P77731	1428	CRYM	crystallin, mu
A_23_P251342	2932	GSK3B	glycogen synthase kinase 3 beta
A_23_P138139	115209	OMA1	OMA1 homolog, zinc metallopeptidase (S. cerevisiae)""
A_23_P36100	1270	CNTF	ciliary neurotrophic factor
A_24_P188116	26287	ANKRD2	ankyrin repeat domain 2 (stretch responsive muscle)
A_24_P61537	1152	CKB	creatine kinase, brain""
A_24_P857430		SQSTM1	sequestosome 1
A_23_P70047	9782	MATR3	matrin 3
A_23_P107401	7077	TIMP2	TIMP metallopeptidase inhibitor 2
A_23_P163787	4313	MMP2	matrix metallopeptidase 2 (gelatinase A, 72kDa gelatinase, 72kDa type IV collagenase)""
A_24_P57528	201266	SLC39A11	solute carrier family 39 (metal ion transporter), member 11
A_23_P13438	84280	BTBD10	BTB (POZ) domain containing 10
A_23_P346311	581	BAX	BCL2-associated X protein
A_23_P145669	2056	EPO	erythropoietin
A_24_P208809	5868	RAB5A	RAB5A, member RAS oncogene family""
A_23_P206585	5579	PRKCB	protein kinase C, beta""
A_23_P145844	4233	MET	met proto-oncogene (hepatocyte growth factor receptor)
A_23_P143734	1565	CYP2D6	cytochrome P450, family 2, subfamily D, polypeptide 6""
A_24_P72139	4671	NAIP	NLR family, apoptosis inhibitory protein""
A_23_P43034	55140	ELP3	elongation protein 3 homolog (S. cerevisiae)
A_24_P206344	155061	ZNF746	zinc finger protein 746
A_24_P122874	66008	TRAK2	trafficking protein, kinesin binding 2""
A_23_P55099	5578	PRKCA	protein kinase C, alpha""
A_23_P99063	4060	LUM	lumican
A_23_P108501	2043	EPHA4	EPH receptor A4
A_24_P497226	6198	RPS6KB1	ribosomal protein S6 kinase, 70kDa, polypeptide 1""
A_23_P62115	7076	TIMP1	TIMP metallopeptidase inhibitor 1
A_23_P5415	60491	NIF3L1	NIF3 NGG1 interacting factor 3-like 1 (S. pombe)
A_24_P276703	23435	TARDBP	TAR DNA binding protein
A_24_P143127	2892	GRIA3	glutamate receptor, ionotrophic, AMPA 3""
A_23_P414793	1356	CP	ceruloplasmin (ferroxidase)
A_24_P274219	2043	EPHA4	EPH receptor A4
A_23_P149626	57449	PLEKHG5	pleckstrin homology domain containing, family G (with RhoGef domain) member 5
A_24_P5305	6687	SPG7	spastic paraplegia 7 (pure and complicated autosomal recessive)
A_23_P74241	4520	MTF1	metal-regulatory transcription factor 1
A_23_P88559	3990	LIPC	lipase, hepatic""
A_23_P154526	2888	GRB14	growth factor receptor-bound protein 14
A_24_P12401	7422	VEGFA	vascular endothelial growth factor A
A_23_P130333	7276	TTR	transthyretin
A_23_P212535	25978	CHMP2B	chromatin modifying protein 2B
A_23_P1461	10133	OPTN	optineurin
A_23_P67162	5300	PIN1	peptidylprolyl cis/trans isomerase, NIMA-interacting 1""
A_24_P932579	4520	MTF1	metal-regulatory transcription factor 1
A_24_P83787	27185	DISC1	disrupted in schizophrenia 1
A_24_P414183	4968	OGG1	8-oxoguanine DNA glycosylase
A_23_P154037	316	AOX1	aldehyde oxidase 1
A_24_P208730	152330	CNTN4	contactin 4
A_23_P81399	8878	SQSTM1	sequestosome 1
A_24_P122137	3976	LIF	leukemia inhibitory factor (cholinergic differentiation factor)
A_23_P433990	6687	SPG7	spastic paraplegia 7 (pure and complicated autosomal recessive)
A_23_P300600	4744	NEFH	neurofilament, heavy polypeptide""

A_24_P22079	2308	FOXO1	forkhead box O1
A_23_P131723	10971	YWHAQ	tyrosine 3-monooxygenase/tryptophan 5-monooxygenase activation protein, theta polypeptide""
A_23_P85765	779	CACNA1S	calcium channel, voltage-dependent, L type, alpha 1S subunit""
A_24_P917261	23064	SETX	senataxin
A_24_P944788	3082	HGF	hepatocyte growth factor (hepapoietin A; scatter factor)
A_23_P90679	55437	STRADB	STE20-related kinase adaptor beta
A_24_P213684		P4HB	prolyl 4-hydroxylase, beta polypeptide""
A_32_P234935	23435	TARDBP	TAR DNA binding protein
A_24_P935819		SOD2	superoxide dismutase 2, mitochondrial""
A_24_P337846	5444	PON1	paraoxonase 1
A_32_P154256	10971	YWHAQ	tyrosine 3-monooxygenase/tryptophan 5-monooxygenase activation protein, theta polypeptide""
A_23_P106887	2521	FUS	fusion (involved in t(12;16) in malignant liposarcoma)
A_24_P387179	6198	RPS6KB1	ribosomal protein S6 kinase, 70kDa, polypeptide 1""
A_32_P453971		SYT9	synaptotagmin IX
A_23_P409966	65059	RAPH1	Ras association (RalGDS/AF-6) and pleckstrin homology domains 1
A_23_P116235	4192	MDK	midkine (neurite growth-promoting factor 2)
A_23_P258493	4001	LMNB1	lamin B1
A_23_P142045	7001	PRDX2	peroxiredoxin 2
A_23_P162719	81624	DIAPH3	diaphanous homolog 3 (Drosophila)
A_23_P58466	6606	SMN1	survival of motor neuron 1, telomeric""
A_23_P106174		PSEN1	presenilin 1
A_23_P7752	57556	SEMA6A	sema domain, transmembrane domain (TM), and cytoplasmic domain, (semaphorin) 6A""
A_24_P929570	65059	RAPH1	Ras association (RalGDS/AF-6) and pleckstrin homology domains 1
A_23_P36496	5937	RBMS1	RNA binding motif, single stranded interacting protein 1
A_23_P209426	66008	TRAK2	trafficking protein, kinesin binding 2""
A_23_P139635	1610	DAO	D-amino-acid oxidase
A_23_P214459	5961	PRPH2	peripherin 2 (retinal degeneration, slow)""
A_23_P161218	27063	ANKRD1	ankyrin repeat domain 1 (cardiac muscle)
A_23_P51187	5590	PRKCZ	protein kinase C, zeta""
A_32_P13555	4868	NPHS1	nephrosis 1, congenital, Finnish type (nephrin)
A_23_P118946	9331	B4GALT6	UDP-Gal:betaGlcNAc beta 1,4- galactosyltransferase, polypeptide 6""
A_24_P114617	25978	CHMP2B	chromatin modifying protein 2B
A_24_P236753	8447	DOC2B	double C2-like domains, beta""
A_23_P310372	56832	IFNK	interferon, kappa""
A_23_P151649	328	APEX1	APEX nuclease (multifunctional DNA repair enzyme) 1
A_24_P411899	25897	RNF19A	ring finger protein 19A
A_23_P156117	26999	CYFIP2	cytoplasmic FMR1 interacting protein 2
A_23_P151426	2308	FOXO1	forkhead box O1
A_23_P134176	6648	SOD2	superoxide dismutase 2, mitochondrial""
A_32_P315770	5937	RBMS1	RNA binding motif, single stranded interacting protein 1""
A_24_P323395	51232	CRIM1	cysteine rich transmembrane BMP regulator 1 (chordin-like)
A_23_P110473	4671	NAIP	NLR family, apoptosis inhibitory protein""
A_24_P928068		TAF15	TAF15 RNA polymerase II, TATA box binding protein (TBP)-associated factor, 68kDa""
A_24_P295412	7415	VCP	valosin-containing protein
A_24_P405631	55079	FEZF2	FEZ family zinc finger 2
A_23_P501877	55734	ZFP64	zinc finger protein 64 homolog (mouse)
A_23_P399078	7078	TIMP3	TIMP metallopeptidase inhibitor 3
A_23_P143526	6285	S100B	S100 calcium binding protein B
A_23_P93780	3082	HGF	hepatocyte growth factor (hepapoietin A; scatter factor)
A_32_P202057	10971	YWHAQ	tyrosine 3-monooxygenase/tryptophan 5-monooxygenase activation protein, theta polypeptide""
A_23_P25674	1152	CKB	creatine kinase, brain""
A_23_P147465	5071	PARK2	Parkinson disease (autosomal recessive, juvenile) 2, parkin""
A_24_P53976	2752	GLUL	glutamate-ammonia ligase (glutamine synthetase)
A_24_P343736	143425	SYT9	synaptotagmin IX
A_23_P201547	22854	NTNG1	netrin G1
A_23_P87952	6660	SOX5	SRY (sex determining region Y)-box 5
A_32_P27046	1113	CHGA	chromogranin A (parathyroid secretory protein 1)
A_32_P148824	54953	C1orf27	chromosome 1 open reading frame 27
A_32_P150891	81624	DIAPH3	diaphanous homolog 3 (Drosophila)
A_23_P67271	5585	PKN1	protein kinase N1
A_23_P92623	5530	PPP3CA	protein phosphatase 3 (formerly 2B), catalytic subunit, alpha isoform""
A_32_P420009	259173	ALS2CL	ALS2 C-terminal like
A_24_P199929	51542	VPS54	vacuolar protein sorting 54 homolog (S. cerevisiae)
A_32_P378278	150864	FAM117B	family with sequence similarity 117, member B""
A_23_P154840	6647	SOD1	superoxide dismutase 1, soluble""
A_23_P40192	64405	CDH22	cadherin-like 22
A_23_P332178	2912	GRM2	glutamate receptor, metabotropic 2""
A_23_P159305	8148	TAF15	TAF15 RNA polymerase II, TATA box binding protein (TBP)-associated factor, 68kDa""
A_23_P145204	3077	HFE	hemochromatosis
A_23_P213602	1456	CSNK1G3	casein kinase 1, gamma 3
A_23_P120467	55734	ZFP64	zinc finger protein 64 homolog (mouse)

A_23_P85008	4129	MAOB	monoamine oxidase B
A_23_P363885		BCL11B	B-cell CLL/lymphoma 11B (zinc finger protein)
A_23_P136077	5071	PARK2	Parkinson disease (autosomal recessive, juvenile) 2, parkin""
A_23_P423926	26039	SS18L1	synovial sarcoma translocation gene on chromosome 18-like 1
A_23_P27096	5216	PFN1	profilin 1
A_24_P354748	3077	HFE	hemochromatosis
A_24_P289648	3077	HFE	hemochromatosis
A_24_P911255	4524	MTHFR	5,10-methylenetetrahydrofolate reductase (NADPH)""
A_24_P234792	1456	CSNK1G3	casein kinase 1, gamma 3
A_23_P345575	2309	FOXO3	forkhead box O3
A_24_P216294	1471	CST3	cystatin C
A_24_P103448	3077	HFE	hemochromatosis
A_23_P145541	9896	FIG4	FIG4 homolog (S. cerevisiae)
A_24_P224488	4137	MAPT	microtubule-associated protein tau
A_23_P146637	10280	SIGMAR1	sigma non-opioid intracellular receptor 1
A_23_P403955	23435	TARDBP	TAR DNA binding protein
A_24_P283288	1432	MAPK14	mitogen-activated protein kinase 14
A_24_P935330	5579	PRKCB	protein kinase C, beta""
A_23_P205738	64919	BCL11B	B-cell CLL/lymphoma 11B (zinc finger protein)
A_23_P107412	5034	P4HB	prolyl 4-hydroxylase, beta polypeptide""
A_32_P72110	5817	PVR	poliovirus receptor
A_23_P312840	57556	SEMA6A	sema domain, transmembrane domain (TM), and cytoplasmic domain, (semaphorin) 6A""
A_24_P228228	9331	B4GALT6	UDP-Gal:betaGlcNAc beta 1,4- galactosyltransferase, polypeptide 6""
A_23_P359131	123606	NIPA1	non imprinted in Prader-Willi/Angelman syndrome 1
A_23_P156880	5167	ENPP1	ectonucleotide pyrophosphatase/phosphodiesterase 1
A_24_P11575	51232	CRIM1	cysteine rich transmembrane BMP regulator 1 (chordin-like)
A_23_P216610	64420	SUSD1	sushi domain containing 1
A_24_P5653	81832	NETO1	neuropilin (NRP) and tolloid (TLL)-like 1
A_23_P170888	1804	DPP6	dipeptidyl-peptidase 6
A_24_P169896	100289922	TIMP2	TIMP metallopeptidase inhibitor 2
A_23_P5200	4868	NPHS1	nephrosis 1, congenital, Finnish type (nephrin)
A_32_P177750	55437	STRADB	STE20-related kinase adaptor beta
A_23_P158969	201266	SLC39A11	solute carrier family 39 (metal ion transporter), member 11
A_23_P257704	3315	HSPB1	heat shock 27kDa protein 1
A_23_P3627	6687	SPG7	spastic paraplegia 7 (pure and complicated autosomal recessive)
A_23_P209356	6683	SPAST	spastin
A_24_P350759	6506	SLC1A2	solute carrier family 1 (glial high affinity glutamate transporter), member 2""
A_23_P56933	57142	RTN4	reticulon 4
A_23_P112296	1621	DBH	dopamine beta-hydroxylase (dopamine beta-monooxygenase)
A_23_P57155	1114	CHGB	chromogranin B (secretogranin 1)
A_23_P419254	81624	DIAPH3	diaphanous homolog 3 (Drosophila)
A_23_P17624	7074	TIAM1	T-cell lymphoma invasion and metastasis 1
A_23_P365494	23025	UNC13A	unc-13 homolog A (C. elegans)
A_24_P264832	4741	NEFM	neurofilament, medium polypeptide""
A_24_P27234	6660	SOX5	SRY (sex determining region Y)-box 5
A_23_P102331	6332	SCN7A	sodium channel, voltage-gated, type VII, alpha""
A_23_P83328	2022	ENG	endoglin
A_23_P144020	152330	CNTN4	contactin 4
A_23_P250671	2876	GPX1	glutathione peroxidase 1
A_23_P404045	4524	MTHFR	5,10-methylenetetrahydrofolate reductase (NADPH)""
A_32_P85999	1012	CDH13	cadherin 13, H-cadherin (heart)""
A_23_P150207	572	BAD	BCL2-associated agonist of cell death
A_23_P4489	81832	NETO1	neuropilin (NRP) and tolloid (TLL)-like 1
A_24_P925186	6660	SOX5	SRY (sex determining region Y)-box 5
A_23_P50250	1158	CKM	creatine kinase, muscle""
A_24_P18137	4747	NEFL	neurofilament, light polypeptide""
A_24_P521409	6198	RPS6KB1	ribosomal protein S6 kinase, 70kDa, polypeptide 1""
A_23_P210920	2937	GSS	glutathione synthetase
A_23_P212196	4968	OGG1	8-oxoguanine DNA glycosylase
A_23_P142835	1639	DCTN1	dynactin 1 (p150, glued homolog, Drosophila)""
A_23_P111737	10268	RAMP3	receptor (G protein-coupled) activity modifying protein 3
A_24_P255471	143425	SYT9	synaptotagmin IX
A_23_P40184	64405	CDH22	cadherin-like 22
A_23_P376488	7124	TNF	tumor necrosis factor (TNF superfamily, member 2)""
A_24_P113960	84618	NT5C1A	5'-nucleotidase, cytosolic IA
A_23_P167509	26999	CYFIP2	cytoplasmic FMR1 interacting protein 2
A_23_P65699	80208	SPG11	spastic paraplegia 11 (autosomal recessive)
A_23_P328642	151254	ALS2CR11	amyotrophic lateral sclerosis 2 (juvenile) chromosome region, candidate 11
A_23_P109322	5121	PCP4	Purkinje cell protein 4
A_24_P199905	10971	YWHAQ	tyrosine 3-monooxygenase/tryptophan 5-monooxygenase activation protein, theta polypeptide""
A_23_P85015	4129	MAOB	monoamine oxidase B

A_23_P217114	210	ALAD	aminolevulinate, delta-, dehydratase""
A_23_P138137	115209	OMA1	OMA1 homolog, zinc metallopeptidase (S. cerevisiae)""
A_23_P145846	4233	MET	met proto-oncogene (hepatocyte growth factor receptor)
A_23_P389907	6311	ATXN2	ataxin 2
A_23_P54079	55644	OSGEP	O-sialoglycoprotein endopeptidase
A_23_P251031	3596	IL13	interleukin 13
A_24_P924862	65059	RAPH1	Ras association (RalGDS/AF-6) and pleckstrin homology domains 1
A_23_P215549	5446	PON3	paraoxonase 3
A_23_P128706	1778	DYNC1H1	dynein, cytoplasmic 1, heavy chain 1""
A_23_P251293	6623	SNCG	synuclein, gamma (breast cancer-specific protein 1)""
A_24_P205120	6687	SPG7	spastic paraplegia 7 (pure and complicated autosomal recessive)
A_24_P127828	7415	VCP	valosin-containing protein
A_32_P195401	150864	FAM117B	family with sequence similarity 117, member B""
A_24_P924462		PRKCZ	protein kinase C, zeta""
A_23_P141329	6198	RPS6KB1	ribosomal protein S6 kinase, 70kDa, polypeptide 1""
A_23_P30976	2911	GRM1	glutamate receptor, metabotropic 1""
A_23_P141894	5817	PVR	poliovirus receptor
A_23_P105138	847	CAT	catalase
A_23_P70398	7422	VEGFA	vascular endothelial growth factor A
A_23_P303072	2890	GRIA1	glutamate receptor, ionotropic, AMPA 1""
A_24_P95273	23064	SETX	senataxin
A_24_P912058		HEXA	hexosaminidase A (alpha polypeptide)
A_23_P162068	6506	SLC1A2	solute carrier family 1 (glial high affinity glutamate transporter), member 2""
A_24_P222441	3073	HEXA	hexosaminidase A (alpha polypeptide)
A_23_P131737	51542	VPS54	vacuolar protein sorting 54 homolog (S. cerevisiae)
A_32_P208078	4524	MTHFR	5,10-methylenetetrahydrofolate reductase (NADPH)""
A_24_P229025		GRIA3	glutamate receptor, ionotrophic, AMPA 3""
A_24_P126682	6607	SMN2	survival of motor neuron 2, centromeric""
A_23_P71492	4747	NEFL	neurofilament, light polypeptide""
A_24_P414371	5530	PPP3CA	protein phosphatase 3 (formerly 2B), catalytic subunit, alpha isoform""
A_32_P209094	55277	FGGY	FGGY carbohydrate kinase domain containing
A_23_P48610	90809	TMEM55B	transmembrane protein 55B
A_24_P168416	7001	PRDX2	peroxiredoxin 2
A_24_P917744		DCTN1	dynactin 1 (p150, glued homolog, Drosophila)
A_23_P58419	3791	KDR	kinase insert domain receptor (a type III receptor tyrosine kinase)
A_23_P155123	1565	CYP2D6	cytochrome P450, family 2, subfamily D, polypeptide 6""
A_23_P74740	11315	PARK7	Parkinson disease (autosomal recessive, early onset) 7""
A_32_P139654	26039	SS18L1	synovial sarcoma translocation gene on chromosome 18-like 1
A_24_P88696	7857	SCG2	secretogranin II (chromogranin C)
A_24_P294842	6310	ATXN1	ataxin 1
A_23_P359504		RBBP9	retinoblastoma binding protein 9
A_23_P420281	5579	PRKCB	protein kinase C, beta""
A_23_P400081	4524	MTHFR	5,10-methylenetetrahydrofolate reductase (NADPH)""
A_23_P13713	5630	PRPH	peripherin
A_23_P62967	27185	DISC1	disrupted in schizophrenia 1
A_24_P4110	55140	ELP3	elongation protein 3 homolog (S. cerevisiae)
A_24_P86537	3315	HSPB1	heat shock 27kDa protein 1
A_24_P179400	7422	VEGFA	vascular endothelial growth factor A
A_23_P151653	328	APEX1	APEX nuclease (multifunctional DNA repair enzyme) 1
A_23_P114164	29978	UBQLN2	ubiquilin 2
A_23_P340617	2891	GRIA2	glutamate receptor, ionotropic, AMPA 2""
A_23_P98369	2893	GRIA4	glutamate receptor, ionotrophic, AMPA 4""
A_23_P203933	3709	ITPR2	inositol 1,4,5-triphosphate receptor, type 2
A_32_P234604	5216	PFN1	profilin 1
A_23_P129101	3073	HEXA	hexosaminidase A (alpha polypeptide)
A_23_P22169	55277	FGGY	FGGY carbohydrate kinase domain containing
A_23_P382460	123606	NIPA1	non imprinted in Prader-Willi/Angelman syndrome 1
A_23_P257538	10741	RBBP9	retinoblastoma binding protein 9
A_24_P162172	80208	SPG11	spastic paraplegia 11 (autosomal recessive)
A_24_P233488	3976	LIF	leukemia inhibitory factor (cholinergic differentiation factor)
A_23_P284	54953	C1orf27	chromosome 1 open reading frame 27
A_32_P177897	2892	GRIA3	glutamate receptor, ionotrophic, AMPA 3""
