# Supplementary material for: Molecular Taxonomy of Sporadic Amyotrophic Lateral Sclerosis Using Disease-Associated Genes
Source: Front Neurol. 2017 Apr 19;8:152. doi: 10.3389/fneur.2017.00152 (PMC5395696; doi:10.3389/fneur.2017.00152)
Supplement: Supplementary file 2 [file Table_2.PDF]

Supplementary Table 2. Differentially expressed SGALS genes: SALS1 vs control

ID	p (Corr)	p	FC ([SALS1] vs [Control])	Log FC ([SALS1] vs [Control])	EntrezGeneID	GENE_SYMBOL	GENE_NAME
A_23_P88470	7,39E-04	2,15E-04	-1,5466504	-0,6291472	54822	TRPM7	transient receptor potential cation channel, subfamily M, member 7""
A_24_P50759	1,75E-08	5,09E-11	-1,9160252	-0,93811655	7124	TNF	tumor necrosis factor (TNF superfamily, member 2)""
A_23_P215897	0,004382418	0,001839849	1,5462277	0,62875277	25897	RNF19A	ring finger protein 19A
A_23_P207699	4,89E-04	1,20E-04	1,8306175	0,87233037	4137	MAPT	microtubule-associated protein tau
A_24_P54174	5,44E-04	1,43E-04	-2,3312705	-1,2211164	7133	TNFRSF1B	tumor necrosis factor receptor superfamily, member 1B""
A_24_P930507	8,40E-04	2,52E-04	-4,017004	-2,00612		ATXN2	ataxin 2
A_23_P2960	3,09E-07	6,30E-09	-1,7097816	-0,77381206	207	AKT1	v-akt murine thymoma viral oncogene homolog 1
A_23_P204791	0,002680218	9,92E-04	-2,763346	-1,4664162	4842	NOS1	nitric oxide synthase 1 (neuronal)
A_23_P114983	1,33E-04	1,81E-05	-2,3613644	-1,2396207	84676	TRIM63	tripartite motif-containing 63
A_24_P59111	2,55E-04	4,77E-05	-2,1665769	-1,1154174		DPP6	dipeptidyl-peptidase 6
A_23_P122216	4,38E-04	1,02E-04	-1,674319	-0,74357444	4015	LOX	lysyl oxidase
A_24_P934355	0,020287285	0,012006761	-2,741434	-1,4549308	4842	NOS1	nitric oxide synthase 1 (neuronal)
A_24_P57528	0,011130347	0,005743648	1,6100664	0,6871202	201266	SLC39A11	solute carrier family 39 (metal ion transporter), member 11
A_23_P145844	0,040594555	0,026629081	-2,0381851	-1,0272851	4233	MET	met proto-oncogene (hepatocyte growth factor receptor)
A_24_P72139	4,56E-05	3,59E-06	-1,8157015	-0,86052704	4671	NAIP	NLR family, apoptosis inhibitory protein""
A_23_P43034	0,019799586	0,011544948	1,4007767	0,4862271	55140	ELP3	elongation protein 3 homolog (S. cerevisiae)
A_23_P55099	1,07E-07	6,26E-10	-1,9752959	-0,9820688	5578	PRKCA	protein kinase C, alpha""
A_24_P274219	1,11E-04	1,29E-05	1,8516288	0,8887949	2043	EPHA4	EPH receptor A4
A_23_P67162	0,002477097	8,81E-04	2,5454855	1,3479408	5300	PIN1	peptidylprolyl cis/trans isomerase, NIMA-interacting 1""
A_23_P81399	3,23E-04	6,77E-05	2,306888	1,205948	8878	SQSTM1	sequestosome 1
A_23_P85765	1,83E-05	9,58E-07	-2,221334	-1,1514263	779	CACNA1S	calcium channel, voltage-dependent, L type, alpha 1S subunit""
A_24_P944788	0,006792288	0,003049599	-4,403738	-2,1387286	3082	HGF	hepatocyte growth factor (hepapoietin A; scatter factor)
A_23_P106887	1,88E-06	5,60E-08	3,6590698	1,8714769	2521	FUS	fusion (involved in t(12;16) in malignant liposarcoma)
A_23_P162719	0,004202363	0,001752006	-2,1550186	-1,1077003	81624	DIAPH3	diaphanous homolog 3 (Drosophila)
A_23_P161218	1,55E-04	2,35E-05	-3,3911185	-1,7617612	27063	ANKRD1	ankyrin repeat domain 1 (cardiac muscle)
A_23_P151649	9,19E-05	9,91E-06	2,3168833	1,2121854	328	APEX1	APEX nuclease (multifunctional DNA repair enzyme) 1
A_24_P295412	0,001730506	5,85E-04	2,394609	1,2597901	7415	VCP	valosin-containing protein
A_23_P399078	2,55E-04	4,84E-05	1,9378449	0,95445305	7078	TIMP3	TIMP metallopeptidase inhibitor 3
A_24_P343736	0,00717739	0,003267917	-1,857872	-0,8936511	143425	SYT9	synaptotagmin IX
A_23_P145204	1,63E-05	7,62E-07	-3,3319786	-1,7363791	3077	HFE	hemochromatosis
A_23_P345575	0,00533332	0,002316807	1,6535977	0,72560835	2309	FOXO3	forkhead box O3
A_24_P935330	3,33E-05	2,33E-06	3,3117352	1,7275873	5579	PRKCB	protein kinase C, beta""
A_23_P107412	7,39E-04	2,14E-04	2,4107664	1,2694919	5034	P4HB	prolyl 4-hydroxylase, beta polypeptide""
A_24_P11575	0,008292533	0,003969521	-2,9593217	-1,5652666	51232	CRIM1	cysteine rich transmembrane BMP regulator 1 (chordin-like)
A_23_P57155	0,004847983	0,002091841	2,9571762	1,5642202	1114	CHGB	chromogranin B (secretogranin 1)
A_23_P365494	0,008292533	0,00398912	2,3765817	1,248888	23025	UNC13A	unc-13 homolog A (C. elegans)
A_23_P50250	0,003024885	0,001172915	-3,8966951	-1,9622511	1158	CKM	creatine kinase, muscle""
A_23_P210920	0,0181454	0,010474604	1,5563183	0,6381371	2937	GSS	glutathione synthetase
A_23_P70398	0,004498513	0,001901704	-2,0016377	-1,0011809	7422	VEGFA	vascular endothelial growth factor A
A_23_P48610	0,001561218	5,19E-04	1,459866	0,54583603	90809	TMEM55B	transmembrane protein 55B
A_23_P58419	1,88E-06	6,02E-08	-2,9041567	-1,5381193	3791	KDR	kinase insert domain receptor (a type III receptor tyrosine kinase)
A_23_P155123	2,87E-07	4,19E-09	-1,5697337	-0,6505199	1565	CYP2D6	cytochrome P450, family 2, subfamily D, polypeptide 6""
A_24_P88696	0,002680218	9,87E-04	3,1003363	1,6324247	7857	SCG2	secretogranin II (chromogranin C)
A_23_P420281	3,57E-05	2,60E-06	2,1769001	1,1222752	5579	PRKCB	protein kinase C, beta""
A_24_P233488	1,49E-05	5,66E-07	-2,3043983	-1,2043902	3976	LIF	leukemia inhibitory factor (cholinergic differentiation factor)
