# Supplementary material for: Molecular Taxonomy of Sporadic Amyotrophic Lateral Sclerosis Using Disease-Associated Genes
Source: Front Neurol. 2017 Apr 19;8:152. doi: 10.3389/fneur.2017.00152 (PMC5395696; doi:10.3389/fneur.2017.00152)
Supplement: Supplementary file 3 [file Table_3.PDF]

Supplementary Table 3. Differentially expressed SGALS genes: SALS2 vs control

ID	p (Corr)	p	FC ([SALS2] vs [Control])	Log FC ([SALS2] vs [Control])	EntrezGeneID	GENE_SYMBOL	GENE_NAME
A_24_P6552	6,68E-05	5,84E-06	3,1805472	1,669275	1804	DPP6	dipeptidyl-peptidase 6
A_23_P426292	0,002545361	9,20E-04	-1,6161122	-0,6925274	1432	MAPK14	mitogen-activated protein kinase 14
A_23_P49708	0,014197025	0,007947023	-2,16333	-1,1132538	2896	GRN	granulin
A_24_P50759	1,75E-08	5,09E-11	1,757953	0,81389654	7124	TNF	tumor necrosis factor (TNF superfamily, member 2)""
A_23_P215897	0,004382418	0,001839849	1,9561126	0,96798944	25897	RNF19A	ring finger protein 19A
A_23_P108180	1,82E-05	9,04E-07	5,432469	2,441608	116444	GRIN3B	glutamate receptor, ionotropic, N-methyl-D-aspartate 3B
A_23_P31399	0,002272276	7,95E-04	-2,3581867	-1,2376779	5445	PON2	paraoxonase 2
A_23_P40174	0,035136346	0,02253643	-2,425446	-1,2782501	4318	MMP9	matrix metallopeptidase 9 (gelatinase B, 92kDa gelatinase, 92kDa type IV collagenase)""
A_24_P151464	1,74E-04	2,83E-05	-3,338445	-1,7391763	6647	SOD1	superoxide dismutase 1, soluble""
A_23_P205686	3,24E-04	6,90E-05	-1,6902367	-0,75722533	5663	PSEN1	presenilin 1
A_24_P916496	0,006690355	0,002984327	-2,6009574	-1,3790427	5578	PRKCA	protein kinase C, alpha""
A_24_P941988	0,013309642	0,007175743	-1,9057021	-0,93032265	6683	SPAST	spastin
A_23_P202658	0,009007729	0,004411949	-2,1759374	-1,1216371	2950	GSTP1	glutathione S-transferase pi 1
A_23_P93787	6,47E-07	1,70E-08	3,433028	1,7794816	3082	HGF	hepatocyte growth factor (hepapoietin A; scatter factor)
A_23_P349343	9,19E-05	9,77E-06	4,3196487	2,110914	130540	ALS2CR12	amyotrophic lateral sclerosis 2 (juvenile) chromosome region, candidate 12""
A_23_P26810	2,68E-07	2,88E-09	2,3440552	1,2290065	7157	TP53	tumor protein p53
A_23_P2960	3,09E-07	6,30E-09	2,3610075	1,2394025	207	AKT1	v-akt murine thymoma viral oncogene homolog 1
A_23_P62920	2,27E-04	3,98E-05	-2,3532848	-1,234676	22920	KIFAP3	kinesin-associated protein 3
A_23_P212545	1,42E-05	4,95E-07	-2,2941494	-1,1979593	5868	RAB5A	RAB5A, member RAS oncogene family""
A_23_P104555	0,009449332	0,004738441	1,687021	0,754478	26287	ANKRD2	ankyrin repeat domain 2 (stretch responsive muscle)
A_24_P322756	3,99E-04	8,83E-05	4,958597	2,309932	7226	TRPM2	transient receptor potential cation channel, subfamily M, member 2""
A_23_P324278	3,16E-04	6,53E-05	2,1316707	1,0919846	210	ALAD	aminolevulinate, delta-, dehydratase""
A_24_P940921	4,50E-04	1,06E-04	-2,5073752	-1,326178	23064	SETX	senataxin
A_23_P90944	1,55E-04	2,34E-05	3,3666546	1,7513157	6332	SCN7A	sodium channel, voltage-gated, type VII, alpha""
A_23_P113111	5,58E-05	4,56E-06	2,2885811	1,1944535	367	AR	androgen receptor
A_23_P114783	0,003387186	0,001352899	-2,0625455	-1,044426	142	PARP1	poly (ADP-ribose) polymerase 1
A_23_P201628	0,006110224	0,002689924	-1,6493939	-0,721936	3915	LAMC1	laminin, gamma 1 (formerly LAMB2)""
A_32_P172339	4,80E-04	1,16E-04	-3,685582	-1,8818924	2891	GRIA2	glutamate receptor, ionotropic, AMPA 2""
A_32_P192376	5,25E-04	1,33E-04	3,8172944	1,9325504	5167	ENPP1	ectonucleotide pyrophosphatase/phosphodiesterase 1
A_23_P92410	0,001843362	6,34E-04	-2,161732	-1,1121876	836	CASP3	caspase 3, apoptosis-related cysteine peptidase""
A_23_P162589	2,33E-05	1,43E-06	3,2154086	1,6850021	7421	VDR	vitamin D (1,25- dihydroxyvitamin D3) receptor""
A_23_P83045	0,003147322	0,001247918	-2,2703903	-1,1829402	7415	VCP	valosin-containing protein
A_23_P77731	0,001730506	5,82E-04	-2,8763428	-1,5242356	1428	CRYM	crystallin, mu
A_23_P138139	0,003771385	0,001550336	-1,8129317	-0,8583245	115209	OMA1	OMA1 homolog, zinc metallopeptidase (S. cerevisiae)""
A_23_P70047	1,63E-05	7,11E-07	-1,7630785	-0,81809664	9782	MATR3	matrin 3
A_23_P163787	3,09E-07	6,18E-09	3,1335099	1,6477796	4313	MMP2	matrix metallopeptidase 2 (gelatinase A, 72kDa gelatinase, 72kDa type IV collagenase)""
A_23_P13438	0,001561218	5,18E-04	-1,7821485	-0,83361757	84280	BTBD10	BTB (POZ) domain containing 10
A_23_P346311	1,63E-05	7,59E-07	2,0248234	1,0177962	581	BAX	BCL2-associated X protein
A_23_P145669	1,33E-04	1,76E-05	3,5295646	1,8194902	2056	EPO	erythropoietin
A_23_P206585	7,02E-04	1,98E-04	-3,0330238	-1,6007569	5579	PRKCB	protein kinase C, beta""
A_24_P206344	0,009120864	0,004547136	-1,904674	-0,92954415	155061	ZNF746	zinc finger protein 746
A_23_P55099	1,07E-07	6,26E-10	2,1201537	1,0841689	5578	PRKCA	protein kinase C, alpha""
A_23_P5415	7,37E-05	6,88E-06	-1,8007386	-0,8485887	60491	NIF3L1	NIF3 NGG1 interacting factor 3-like 1 (S. pombe)
A_24_P5305	0,001835131	6,26E-04	2,2232065	1,152642	6687	SPG7	spastic paraplegia 7 (pure and complicated autosomal recessive)
A_23_P88559	2,78E-04	5,40E-05	4,7824616	2,2577534	3990	LIPC	lipase, hepatic""
A_23_P300600	0,001231429	3,88E-04	-4,986074	-2,3179042	4744	NEFH	neurofilament, heavy polypeptide""
A_24_P22079	0,002801909	0,001045611	1,6037631	0,68146104	2308	FOXO1	forkhead box O1
A_23_P131723	7,96E-05	7,99E-06	-2,7670584	-1,468353	10971	YWHAQ	tyrosine 3-monooxygenase/tryptophan 5-monooxygenase activation protein, theta polypeptide""
A_24_P917261	2,27E-04	4,04E-05	3,610761	1,8523029	23064	SETX	senataxin
A_32_P234935	0,001126653	3,51E-04	-2,5431569	-1,3466204	23435	TARDBP	TAR DNA binding protein
A_32_P154256	2,79E-05	1,87E-06	-3,4622328	-1,7917027	10971	YWHAQ	tyrosine 3-monooxygenase/tryptophan 5-monooxygenase activation protein, theta polypeptide""
A_32_P453971	4,38E-04	1,02E-04	6,3930607	2,6765068		SYT9	synaptotagmin IX
A_23_P258493	4,57E-04	1,09E-04	2,2320185	1,158349	4001	LMNB1	lamin B1

A_23_P58466	1,44E-04	2,01E-05	-2,0797548	-1,0564135	6606	SMN1	survival of motor neuron 1, telomeric""
A_23_P106174	1,86E-05	1,03E-06	1,5645905	0,6457851		PSEN1	presenilin 1
A_23_P139635	0,002900638	0,001090911	3,0877092	1,6265368	1610	DAO	D-amino-acid oxidase
A_32_P13555	0,001515609	4,95E-04	3,7944536	1,9238921	4868	NPHS1	nephrosis 1, congenital, Finnish type (nephrin)
A_24_P236753	6,51E-05	5,50E-06	3,0168989	1,5930663	8447	DOC2B	double C2-like domains, beta""
A_23_P310372	2,55E-04	4,69E-05	5,46847	2,4511373	56832	IFNK	interferon, kappa""
A_23_P134176	2,78E-04	5,56E-05	-1,9216988	-0,9423823	6648	SOD2	superoxide dismutase 2, mitochondrial""
A_32_P315770	5,88E-04	1,61E-04	6,4432964	2,687799	5937	RBMS1	RNA binding motif, single stranded interacting protein 1""
A_24_P323395	1,49E-04	2,13E-05	3,4471574	1,7854072	51232	CRIM1	cysteine rich transmembrane BMP regulator 1 (chordin-like)
A_24_P295412	0,001730506	5,85E-04	1,8225719	0,86597574	7415	VCP	valosin-containing protein
A_23_P143526	1,13E-04	1,35E-05	-3,5331843	-1,820969	6285	S100B	S100 calcium binding protein B
A_32_P202057	1,33E-04	1,82E-05	-3,9645512	-1,9871576	10971	YWHAQ	tyrosine 3-monooxygenase/tryptophan 5-monooxygenase activation protein, theta polypeptide""
A_23_P87952	0,00771387	0,003575817	2,0451076	1,0321767	6660	SOX5	SRY (sex determining region Y)-box 5
A_32_P150891	2,26E-04	3,82E-05	3,3596296	1,7483022	81624	DIAPH3	diaphanous homolog 3 (Drosophila)
A_23_P92623	8,15E-04	2,42E-04	-2,8511763	-1,5115572	5530	PPP3CA	protein phosphatase 3 (formerly 2B), catalytic subunit, alpha isoform""
A_23_P154840	8,92E-04	2,73E-04	-3,4372497	-1,7812546	6647	SOD1	superoxide dismutase 1, soluble""
A_23_P159305	0,0070597	0,003190243	-2,3940203	-1,2594354	8148	TAF15	TAF15 RNA polymerase II, TATA box binding protein (TBP)-associated factor, 68kDa""
A_24_P354748	3,96E-05	3,00E-06	2,2376497	1,1619842	3077	HFE	hemochromatosis
A_23_P158969	0,008068056	0,003787047	2,4005866	1,263387	201266	SLC39A11	solute carrier family 39 (metal ion transporter), member 11
A_24_P350759	0,004154357	0,00171988	-3,3412654	-1,7403946	6506	SLC1A2	solute carrier family 1 (glial high affinity glutamate transporter), member 2""
A_23_P56933	7,96E-05	7,76E-06	-3,3409104	-1,7402413	57142	RTN4	reticulon 4
A_24_P264832	0,013309642	0,007295081	-2,8369915	-1,5043619	4741	NEFM	neurofilament, medium polypeptide""
A_32_P85999	6,94E-05	6,27E-06	-1,9784964	-0,98440444	1012	CDH13	cadherin 13, H-cadherin (heart)""
A_24_P925186	2,79E-05	1,80E-06	4,0984325	2,0350723	6660	SOX5	SRY (sex determining region Y)-box 5
A_24_P18137	2,78E-04	5,60E-05	-3,9714756	-1,9896752	4747	NEFL	neurofilament, light polypeptide""
A_24_P521409	7,96E-05	8,12E-06	3,7616074	1,9113493	6198	RPS6KB1	ribosomal protein S6 kinase, 70kDa, polypeptide 1""
A_23_P376488	0,003642231	0,001476006	2,6811032	1,4228268	7124	TNF	tumor necrosis factor (TNF superfamily, member 2)""
A_24_P113960	1,00E-04	1,14E-05	2,3335369	1,2225182	84618	NT5C1A	5'-nucleotidase, cytosolic IA
A_23_P167509	2,78E-04	5,49E-05	-1,9006348	-0,9264813	26999	CYFIP2	cytoplasmic FMR1 interacting protein 2
A_23_P109322	7,32E-04	2,09E-04	-4,0190945	-2,0068705	5121	PCP4	Purkinje cell protein 4
A_24_P199905	1,61E-04	2,58E-05	-3,31067	-1,7271231	10971	YWHAQ	tyrosine 3-monooxygenase/tryptophan 5-monooxygenase activation protein, theta polypeptide""
A_23_P138137	0,013361487	0,007362452	-1,647275	-0,7200814	115209	OMA1	OMA1 homolog, zinc metallopeptidase (S. cerevisiae)""
A_23_P389907	0,012214252	0,006481031	-2,6799395	-1,4222004	6311	ATXN2	ataxin 2
A_23_P251031	1,58E-04	2,46E-05	3,8872347	1,9587442	3596	IL13	interleukin 13
A_24_P924862	0,038454387	0,025000958	-2,401026	-1,263651	65059	RAPH1	Ras association (RalGDS/AF-6) and pleckstrin homology domains 1
A_24_P205120	2,27E-04	3,92E-05	3,7162259	1,8938382	6687	SPG7	spastic paraplegia 7 (pure and complicated autosomal recessive)
A_24_P127828	1,58E-04	2,49E-05	1,3979285	0,48329058	7415	VCP	valosin-containing protein
A_32_P195401	9,31E-04	2,88E-04	-2,3279521	-1,2190614	150864	FAM117B	family with sequence similarity 117, member B""
A_23_P105138	0,002968861	0,00113388	-1,7643337	-0,81912345	847	CAT	catalase
A_24_P95273	1,15E-04	1,41E-05	-1,5528747	-0,63494146	23064	SETX	senataxin
A_24_P222441	2,68E-07	3,13E-09	9,439334	3,2386851	3073	HEXA	hexosaminidase A (alpha polypeptide)
A_23_P131737	0,029022144	0,018022498	1,7087352	0,77292883	51542	VPS54	vacuolar protein sorting 54 homolog (S. cerevisiae)
A_24_P126682	0,001973818	6,85E-04	3,4763114	1,7975574	6607	SMN2	survival of motor neuron 2, centromeric""
A_24_P414371	9,36E-05	1,04E-05	-2,4066088	-1,2670016	5530	PPP3CA	protein phosphatase 3 (formerly 2B), catalytic subunit, alpha isoform""
A_24_P917744	2,52E-04	4,55E-05	5,710562	2,5136328		DCTN1	dynactin 1 (p150, glued homolog, Drosophila)
A_23_P58419	1,88E-06	6,02E-08	3,3630815	1,7497838	3791	KDR	kinase insert domain receptor (a type III receptor tyrosine kinase)
A_23_P155123	2,87E-07	4,19E-09	1,6266361	0,7018916	1565	CYP2D6	cytochrome P450, family 2, subfamily D, polypeptide 6""
A_23_P74740	0,001240885	3,94E-04	-2,222999	-1,1525073	11315	PARK7	Parkinson disease (autosomal recessive, early onset) 7""
A_23_P359504	6,47E-07	1,52E-08	48,894844	5,6116104		RBBP9	retinoblastoma binding protein 9
A_23_P400081	2,33E-05	1,42E-06	2,9265203	1,5491863	4524	MTHFR	5,10-methylenetetrahydrofolate reductase (NADPH)""
A_24_P4110	0,00717739	0,003285278	3,2669985	1,7079659	55140	ELP3	elongation protein 3 homolog (S. cerevisiae)
A_23_P151653	0,01784091	0,010246821	4,1269608	2,0450797	328	APEX1	APEX nuclease (multifunctional DNA repair enzyme) 1
A_23_P114164	6,87E-04	1,90E-04	-2,0961998	-1,0677762	29978	UBQLN2	ubiquilin 2
A_23_P22169	3,31E-04	7,15E-05	1,8724585	0,9049337	55277	FGGY	FGGY carbohydrate kinase domain containing
A_32_P177897	8,91E-04	2,70E-04	-2,3198671	-1,2140422	2892	GRIA3	glutamate receptor, ionotrophic, AMPA 3""
