# Supplementary material for: Molecular Taxonomy of Sporadic Amyotrophic Lateral Sclerosis Using Disease-Associated Genes
Source: Front Neurol. 2017 Apr 19;8:152. doi: 10.3389/fneur.2017.00152 (PMC5395696; doi:10.3389/fneur.2017.00152)
Supplement: Supplementary file 4 [file Table_4.PDF]

Supplementary Table 4. List of the significantly enriched GO biological processes for differentially expressed SGALS genes in SALS1 patients

GO biological process	SGALS genes in category	p value	fdr p value	-LOG(corr.pValue)
**cellular response to chemical stimulus**	24	8,30E-10	2,34E-06	12,96699605
**positive chemotaxis**	6	2,33E-09	3,29E-06	12,62582507
response to chemical	28	4,39E-09	4,12E-06	12,39877977
programmed cell death	19	6,58E-09	4,63E-06	12,28351066
**cell death**	19	1,63E-08	6,63E-06	11,92412661
death	19	1,63E-08	6,63E-06	11,92412661
**response to oxidative stress**	10	1,65E-08	6,63E-06	11,92412661
**positive regulation of cell differentiation**	13	2,59E-08	8,44E-06	11,68294954
**positive regulation of locomotion**	10	2,95E-08	8,44E-06	11,68294954
**regulation of catabolic process**	12	3,00E-08	8,44E-06	11,68294954
positive regulation of multicellular organismal process	16	4,42E-08	1,09E-05	11,42448963
cellular response to oxidative stress	8	4,66E-08	1,09E-05	11,42448963
regulation of cellular catabolic process	11	5,35E-08	1,16E-05	11,36619539
regulation of programmed cell death	16	6,21E-08	1,25E-05	11,29163091
regulation of cellular protein metabolic process	20	8,16E-08	1,53E-05	11,08640084
regulation of locomotion	12	9,24E-08	1,63E-05	11,02709406
regulation of cell death	16	1,43E-07	2,24E-05	10,70812282
peptidyl-amino acid modification	15	1,61E-07	2,24E-05	10,70812282
**positive regulation of developmental process**	14	1,62E-07	2,24E-05	10,70812282
regulation of cellular response to stress	11	1,71E-07	2,24E-05	10,70812282
positive regulation of phosphorus metabolic process	14	1,75E-07	2,24E-05	10,70812282
positive regulation of phosphate metabolic process	14	1,75E-07	2,24E-05	10,70812282
regulation of cellular response to oxidative stress	5	2,46E-07	2,81E-05	10,48134384
**positive regulation of cell communication**	16	2,47E-07	2,81E-05	10,48134384
regulation of multicellular organismal development	16	2,52E-07	2,81E-05	10,48134384
regulation of protein metabolic process	20	2,59E-07	2,81E-05	10,48134384
apoptotic process	17	2,73E-07	2,85E-05	10,46671493
**intracellular signal transduction**	20	2,92E-07	2,87E-05	10,45928072
positive regulation of protein modification process	14	3,02E-07	2,87E-05	10,45928072
positive regulation of positive chemotaxis	4	3,13E-07	2,87E-05	10,45928072
cell death in response to oxidative stress	5	3,17E-07	2,87E-05	10,45928072
regulation of response to oxidative stress	5	3,44E-07	2,87E-05	10,45928072
regulation of localization	19	3,50E-07	2,87E-05	10,45928072
negative regulation of programmed cell death	12	3,57E-07	2,87E-05	10,45928072
regulation of positive chemotaxis	4	3,70E-07	2,87E-05	10,45928072
positive regulation of phosphorylation	13	3,85E-07	2,87E-05	10,45928072
regulation of apoptotic process	15	3,99E-07	2,87E-05	10,45928072
positive regulation of endothelial cell migration	5	4,02E-07	2,87E-05	10,45928072
phosphorylation	18	4,04E-07	2,87E-05	10,45928072
enzyme linked receptor protein signaling pathway	14	4,08E-07	2,87E-05	10,45928072
regulation of signaling	21	4,70E-07	3,23E-05	10,34146797
positive regulation of endothelial cell proliferation	5	5,05E-07	3,30E-05	10,31915953
positive regulation of cellular protein metabolic process	15	5,16E-07	3,30E-05	10,31915953
regulation of cell communication	21	5,18E-07	3,30E-05	10,31915953
response to organic substance	21	5,27E-07	3,30E-05	10,31915953
cellular response to growth factor stimulus	12	7,09E-07	4,34E-05	10,04482433

positive regulation of neuron projection development	7	7,73E-07	4,56E-05	9,995027908
negative regulation of cell death	12	7,78E-07	4,56E-05	9,995027908
response to stress	24	8,10E-07	4,58E-05	9,992020028
regulation of cellular component movement	11	8,29E-07	4,58E-05	9,992020028
system development	25	8,43E-07	4,58E-05	9,992020028
negative regulation of catabolic process	7	8,45E-07	4,58E-05	9,992020028
protein autophosphorylation	7	8,71E-07	4,62E-05	9,981578054
positive regulation of cellular component organization	13	9,10E-07	4,74E-05	9,955963413
macromolecule modification	24	9,41E-07	4,80E-05	9,944692033
response to growth factor	12	9,55E-07	4,80E-05	9,944692033
regulation of multicellular organismal process	19	1,11E-06	5,47E-05	9,81286949
positive regulation of protein metabolic process	15	1,15E-06	5,56E-05	9,797790763
positive regulation of response to stimulus	17	1,20E-06	5,72E-05	9,769808743
protein phosphorylation	16	1,23E-06	5,72E-05	9,769808743
cardiovascular system development	12	1,26E-06	5,72E-05	9,769808743
circulatory system development	12	1,26E-06	5,72E-05	9,769808743
endothelial cell migration	6	1,33E-06	5,91E-05	9,735603364
positive regulation of signaling	15	1,34E-06	5,91E-05	9,735603364
regulation of branching involved in salivary gland morphogenesis	3	1,41E-06	6,10E-05	9,704686526
muscle cell differentiation	8	1,51E-06	6,44E-05	9,650023105
striated muscle cell differentiation	7	1,57E-06	6,59E-05	9,62687298
regulation of signal transduction	19	1,65E-06	6,81E-05	9,594330097
positive regulation of protein phosphorylation	12	1,89E-06	7,58E-05	9,487698487
positive regulation of endothelial cell chemotaxis	3	1,93E-06	7,58E-05	9,487698487
cellular protein modification process	23	1,99E-06	7,58E-05	9,487698487
protein modification process	23	1,99E-06	7,58E-05	9,487698487
regulation of biological quality	22	1,99E-06	7,58E-05	9,487698487
regulation of endopeptidase activity	8	2,01E-06	7,58E-05	9,487698487
regulation of intracellular signal transduction	15	2,02E-06	7,58E-05	9,487698487
regulation of cell development	11	2,12E-06	7,85E-05	9,452955311
negative regulation of apoptotic process	11	2,61E-06	9,51E-05	9,260204544
phosphate-containing compound metabolic process	20	2,67E-06	9,51E-05	9,260204544
positive regulation of cell migration	8	2,69E-06	9,51E-05	9,260204544
positive regulation of signal transduction	14	2,70E-06	9,51E-05	9,260204544
regulation of endothelial cell proliferation	5	2,82E-06	9,80E-05	9,230461315
MAPK cascade	11	2,85E-06	9,80E-05	9,230461315
regulation of neuron projection development	8	2,90E-06	9,84E-05	9,226262399
cellular protein metabolic process	26	2,95E-06	9,89E-05	9,221630857
regulation of peptidase activity	8	3,01E-06	9,98E-05	9,212452784
positive regulation of nucleobase-containing compound metabolic process	15	3,24E-06	0,000105961	9,152437655
positive regulation of cell motility	8	3,37E-06	0,000107721	9,135964459
regulation of cell motility	10	3,39E-06	0,000107721	9,135964459
positive regulation of cellular process	25	3,43E-06	0,000107721	9,135964459
regulation of phosphorylation	14	3,47E-06	0,000107721	9,135964459
regulation of phosphate metabolic process	15	3,48E-06	0,000107721	9,135964459
negative regulation of hydrolase activity	8	3,56E-06	0,000108925	9,124855091
phosphorus metabolic process	20	3,70E-06	0,00011096	9,106336436
regulation of cell differentiation	14	3,78E-06	0,00011096	9,106336436

regulation of glucose transport	5	3,81E-06	0,00011096	9,106336436
positive regulation of epithelial cell migration	5	3,81E-06	0,00011096	9,106336436
regulation of phosphorus metabolic process	15	3,83E-06	0,00011096	9,106336436
regulation of proteolysis	10	3,89E-06	0,00011096	9,106336436
regulation of oxidative stress-induced cell death	4	3,90E-06	0,00011096	9,106336436
signal transduction by protein phosphorylation	11	3,98E-06	0,000111978	9,09720725
positive regulation of cellular component movement	8	4,11E-06	0,000114678	9,073379854
positive regulation of response to external stimulus	7	4,61E-06	0,000127193	8,969801515
cell motility	13	4,77E-06	0,000129196	8,954180122
localization of cell	13	4,77E-06	0,000129196	8,954180122
positive regulation of cell projection organization	7	5,15E-06	0,000137987	8,888353993
negative regulation of molecular function	12	5,25E-06	0,000137987	8,888353993
induction of positive chemotaxis	3	5,30E-06	0,000137987	8,888353993
regulation of stress-activated MAPK cascade	6	5,32E-06	0,000137987	8,888353993
histone H3-T6 phosphorylation	2	5,34E-06	0,000137987	8,888353993
response to endogenous stimulus	15	5,45E-06	0,000138904	8,881730719
regulation of stress-activated protein kinase signaling cascade	6	5,48E-06	0,000138904	8,881730719
regulation of protein modification process	15	5,57E-06	0,000139893	8,87463071
regulation of anatomical structure morphogenesis	11	5,82E-06	0,00014417	8,844515533
positive regulation of neuron differentiation	7	5,87E-06	0,00014417	8,844515533
muscle structure development	9	5,89E-06	0,00014417	8,844515533
regulation of autophagy	6	5,98E-06	0,000145111	8,838010658
regulation of endothelial cell migration	5	6,03E-06	0,000145111	8,838010658
positive regulation of nitrogen compound metabolic process	15	6,26E-06	0,000147782	8,819775289
transmembrane receptor protein tyrosine kinase signaling pathway	11	6,26E-06	0,000147782	8,819775289
endothelial cell proliferation	5	6,30E-06	0,000147782	8,819775289
positive regulation of biosynthetic process	15	6,75E-06	0,00015698	8,759389364
positive regulation of macromolecule metabolic process	19	7,07E-06	0,000163037	8,721533034
single-organism organelle organization	18	7,17E-06	0,000164034	8,715435825
regulation of cysteine-type endopeptidase activity involved in apoptotic process	6	7,31E-06	0,000165903	8,70410433
secretion by cell	11	7,51E-06	0,000168468	8,688764197
blood vessel development	9	7,54E-06	0,000168468	8,688764197
positive regulation of cellular metabolic process	19	7,68E-06	0,00017019	8,678593133
regulation of endothelial cell chemotaxis	3	7,89E-06	0,000173544	8,659080782
cell-type specific apoptotic process	8	7,99E-06	0,000173953	8,656724111
regulation of morphogenesis of a branching structure	4	8,03E-06	0,000173953	8,656724111
positive regulation of angiogenesis	5	8,80E-06	0,000187835	8,579947355
regulation of angiogenesis	6	8,86E-06	0,000187835	8,579947355
nervous system development	17	8,88E-06	0,000187835	8,579947355
negative regulation of response to stimulus	13	8,94E-06	0,000187835	8,579947355
multicellular organism development	25	9,05E-06	0,00018869	8,575403292
positive regulation of chemotaxis	5	9,16E-06	0,000189636	8,57040285
negative regulation of glucose transport	3	9,45E-06	0,000193901	8,54816485
response to oxygen-containing compound	14	9,51E-06	0,000193901	8,54816485
cellular response to organic substance	17	9,67E-06	0,00019509	8,542050925
regulation of protein phosphorylation	13	9,75E-06	0,00019509	8,542050925
regulation of MAPK cascade	10	9,77E-06	0,00019509	8,542050925
vasculature development	9	1,02E-05	0,000202542	8,504563833

regulation of cysteine-type endopeptidase activity	6	1,04E-05	0,000202567	8,504438133
single-organism developmental process	27	1,04E-05	0,000202567	8,504438133
peptidyl-tyrosine phosphorylation	7	1,04E-05	0,000202567	8,504438133
peptidyl-tyrosine modification	7	1,08E-05	0,000209188	8,472279026
positive regulation of cell development	8	1,10E-05	0,00020998	8,468497638
regulation of developmental process	16	1,11E-05	0,000211714	8,460273495
epithelial cell migration	6	1,12E-05	0,000212499	8,45657424
cell migration	12	1,17E-05	0,000218809	8,42731237
response to reactive oxygen species	6	1,18E-05	0,000220595	8,419180153
apoptotic signaling pathway	9	1,19E-05	0,000220595	8,419180153
epithelium migration	6	1,21E-05	0,000223525	8,405985799
regulation of cellular component organization	16	1,27E-05	0,000232053	8,368543859
regulation of peptide hormone secretion	6	1,28E-05	0,000232132	8,368205453
cellular response to stimulus	30	1,32E-05	0,000238869	8,339594403
tissue migration	6	1,38E-05	0,000247084	8,305781478
positive regulation of vasculature development	5	1,39E-05	0,000248393	8,300496742
regulation of peptide secretion	6	1,45E-05	0,000256375	8,268868735
regulation of vasculature development	6	1,48E-05	0,000261121	8,250527248
cellular response to stress	15	1,52E-05	0,000265236	8,234890443
positive regulation of transcription from RNA polymerase II promoter	11	1,54E-05	0,00026807	8,224263119
cell chemotaxis	6	1,56E-05	0,000269154	8,220225311
regulation of neurogenesis	9	1,58E-05	0,000271878	8,210155666
regulation of response to stimulus	21	1,66E-05	0,000282477	8,171912686
cellular catabolic process	14	1,67E-05	0,000282477	8,171912686
regulation of cell migration	9	1,68E-05	0,00028363	8,167838429
regulation of ERK1 and ERK2 cascade	6	1,72E-05	0,000287574	8,154030407
negative regulation of proteolysis	7	1,73E-05	0,000288359	8,151304668
striated muscle cell development	5	1,79E-05	0,000294636	8,12977064
negative regulation of endopeptidase activity	6	1,80E-05	0,000294636	8,12977064
negative regulation of cellular protein metabolic process	11	1,80E-05	0,000294636	8,12977064
regulation of cellular localization	12	1,91E-05	0,000310606	8,07698494
response to inorganic substance	8	1,98E-05	0,000319866	8,047608821
response to nutrient levels	8	2,01E-05	0,000322543	8,039274201
regulation of hydrolase activity	12	2,02E-05	0,000322786	8,038519784
positive regulation of blood vessel endothelial cell migration	3	2,04E-05	0,000323691	8,035720105
response to external stimulus	17	2,06E-05	0,000325012	8,031649978
regulation of molecular function	18	2,09E-05	0,000327038	8,025434651
regulation of system process	8	2,09E-05	0,000327038	8,025434651
response to wounding	11	2,11E-05	0,000327486	8,024065829
glucose transport	5	2,12E-05	0,000327486	8,024065829
peptidyl-serine phosphorylation	6	2,17E-05	0,00033344	8,006046179
anatomical structure development	25	2,19E-05	0,000334667	8,002374617
regulation of cell projection organization	8	2,24E-05	0,000340491	7,985122286
positive regulation of cell growth	5	2,26E-05	0,000340491	7,985122286
hexose transport	5	2,26E-05	0,000340491	7,985122286
positive regulation of neurogenesis	7	2,29E-05	0,000342262	7,979933175
ERK1 and ERK2 cascade	6	2,32E-05	0,000342262	7,979933175
negative regulation of peptidase activity	6	2,32E-05	0,000342262	7,979933175

branching involved in salivary gland morphogenesis	3	2,32E-05	0,000342262	7,979933175
positive regulation of behavior	5	2,34E-05	0,000342635	7,978844789
blood vessel endothelial cell migration	4	2,35E-05	0,000343487	7,976359948
regulation of transport	14	2,39E-05	0,000347228	7,965527835
monosaccharide transport	5	2,41E-05	0,000348484	7,96191936
secretion	11	2,52E-05	0,000361263	7,925904177
regulation of p38MAPK cascade	3	2,63E-05	0,000374577	7,889714073
endothelial cell chemotaxis	3	2,63E-05	0,000374577	7,889714073
muscle cell development	5	2,74E-05	0,00038789	7,854789823
positive regulation of cellular biosynthetic process	14	2,79E-05	0,000392741	7,842359167
peptidyl-serine modification	6	2,96E-05	0,000413376	7,791153419
response to extracellular stimulus	8	2,98E-05	0,000413376	7,791153419
protein metabolic process	26	2,98E-05	0,000413376	7,791153419
regulation of neuron differentiation	8	3,14E-05	0,000430691	7,750118958
regulation of protein catabolic process	7	3,14E-05	0,000430691	7,750118958
stress-activated MAPK cascade	6	3,15E-05	0,000430691	7,750118958
positive regulation of epithelial cell proliferation	5	3,20E-05	0,000435147	7,739826
regulation of transcription from RNA polymerase II promoter	14	3,23E-05	0,000436469	7,736793727
negative regulation of protein metabolic process	11	3,24E-05	0,000436865	7,735887346
peptide hormone secretion	6	3,50E-05	0,000469313	7,664240832
cellular component organization	27	3,59E-05	0,000478463	7,644931895
positive regulation of myeloid cell differentiation	4	3,75E-05	0,000494806	7,611344696
muscle adaptation	4	3,75E-05	0,000494806	7,611344696
positive regulation of transport	10	3,76E-05	0,000494806	7,611344696
negative regulation of cellular process	22	3,79E-05	0,000496595	7,607736555
homeostatic process	13	3,83E-05	0,000499487	7,601928788
regulation of hormone secretion	6	3,88E-05	0,000503446	7,59403435
regulation of epithelial cell migration	5	3,94E-05	0,000508504	7,584037602
regulation of peptide transport	6	4,12E-05	0,000527718	7,546948829
peptide secretion	6	4,12E-05	0,000527718	7,546948829
proteolysis	13	4,18E-05	0,000532109	7,538662692
regulation of nervous system development	9	4,32E-05	0,000547852	7,509505873
p38MAPK cascade	3	4,61E-05	0,000582345	7,448446799
generation of neurons	13	4,70E-05	0,000590637	7,434308132
negative regulation of cellular metabolic process	16	4,78E-05	0,0005979	7,422087333
stress-activated protein kinase signaling cascade	6	5,03E-05	0,000625957	7,376229671
angiogenesis	7	5,09E-05	0,00063116	7,36795083
negative regulation of metabolic process	17	5,24E-05	0,000647198	7,342858249
regulation of branching involved in salivary gland morphogenesis by mesenchymal-epithelial signaling	2	5,32E-05	0,00065018	7,338260725
positive regulation of endothelial cell chemotaxis by VEGF-activated vascular endothelial growth factor receptor signaling pathway	2	5,32E-05	0,00065018	7,338260725
organelle organization	20	5,34E-05	0,00065018	7,338260725
positive regulation of protein serine/threonine kinase activity	6	5,43E-05	0,000656235	7,328991251
negative regulation of cysteine-type endopeptidase activity involved in apoptotic process	4	5,43E-05	0,000656235	7,328991251
single organism signaling	27	5,52E-05	0,000664247	7,316855771
regulation of protein localization	10	5,64E-05	0,000675404	7,300200169
negative regulation of cellular catabolic process	5	5,81E-05	0,000692505	7,275194886
positive regulation of peptidyl-serine phosphorylation	4	5,93E-05	0,000700883	7,263169424
negative regulation of extrinsic apoptotic signaling pathway	4	5,93E-05	0,000700883	7,263169424

regeneration	5	5,96E-05	0,000701552	7,262216149
positive regulation of nervous system development	7	5,98E-05	0,000701552	7,262216149
regulation of cellular metabolic process	26	6,09E-05	0,0007112	7,248557
positive regulation of macromolecule biosynthetic process	13	6,15E-05	0,000711515	7,248114601
negative regulation of oxidative stress-induced cell death	3	6,17E-05	0,000711515	7,248114601
negative regulation of cysteine-type endopeptidase activity	4	6,18E-05	0,000711515	7,248114601
response to hypoxia	6	6,19E-05	0,000711515	7,248114601
regulation of chemotaxis	5	6,28E-05	0,000716698	7,240856045
response to abiotic stimulus	11	6,29E-05	0,000716698	7,240856045
cell development	15	6,32E-05	0,000717621	7,23956923
signal release	7	6,52E-05	0,000737015	7,212902126
positive regulation of MAPK cascade	8	6,57E-05	0,000739916	7,208973553
positive regulation of transcription, DNA-templated	12	6,74E-05	0,000749463	7,196154121
positive regulation of nucleic acid-templated transcription	12	6,74E-05	0,000749463	7,196154121
regulation of response to reactive oxygen species	3	6,76E-05	0,000749463	7,196154121
carbohydrate transport	5	6,79E-05	0,000749463	7,196154121
regulation of organ morphogenesis	5	6,79E-05	0,000749463	7,196154121
positive regulation of intracellular signal transduction	10	7,11E-05	0,000782018	7,153632922
catabolic process	15	7,15E-05	0,000782439	7,153093996
cellular metabolic process	35	7,17E-05	0,000782439	7,153093996
response to decreased oxygen levels	6	7,30E-05	0,000793517	7,139034973
negative regulation of cellular response to oxidative stress	3	7,38E-05	0,00079563	7,136376335
negative regulation of response to oxidative stress	3	7,38E-05	0,00079563	7,136376335
regulation of response to stress	12	7,76E-05	0,000834004	7,089272866
movement of cell or subcellular component	14	7,85E-05	0,000836825	7,085895978
protein catabolic process	9	7,88E-05	0,000836825	7,085895978
neurogenesis	13	7,88E-05	0,000836825	7,085895978
negative regulation of hydrogen peroxide-mediated programmed cell death	2	7,97E-05	0,00083998	7,082132569
positive regulation of cell migration by vascular endothelial growth factor signaling pathway	2	7,97E-05	0,00083998	7,082132569
macrophage differentiation	3	8,03E-05	0,000840784	7,081175493
salivary gland morphogenesis	3	8,03E-05	0,000840784	7,081175493
lung development	5	8,09E-05	0,000843851	7,077535201
positive regulation of RNA biosynthetic process	12	8,13E-05	0,00084487	7,0763278
positive regulation of cell death	8	8,25E-05	0,000854155	7,065397649
chemical homeostasis	10	8,34E-05	0,000859575	7,05907208
tissue morphogenesis	8	8,73E-05	0,000896454	7,017063301
respiratory tube development	5	8,92E-05	0,000913231	6,998521354
negative regulation of transport	7	9,08E-05	0,00092651	6,984085667
regulation of secretion by cell	8	9,12E-05	0,000926782	6,983792536
hormone secretion	6	9,50E-05	0,000958144	6,950512991
ameboidal-type cell migration	6	9,50E-05	0,000958144	6,950512991
response to oxygen levels	6	9,66E-05	0,000971132	6,937048428
female pregnancy	5	9,81E-05	0,000979547	6,9284207
morphogenesis of a branching epithelium	5	9,81E-05	0,000979547	6,9284207
regulation of growth	8	9,95E-05	0,00098994	6,917865845
positive regulation of cellular catabolic process	6	0,00010335	0,001024404	6,883644272
positive regulation of RNA metabolic process	12	0,00010566	0,001043622	6,865057661
cellular response to vascular endothelial growth factor stimulus	3	0,00011046	0,001076724	6,833832578

salivary gland development	3	0,00011046	0,001076724	6,833832578
hormone transport	6	0,000110476	0,001076724	6,833832578
transcription from RNA polymerase II promoter	14	0,000110541	0,001076724	6,833832578
histone-threonine phosphorylation	2	0,000111375	0,001081102	6,829774075
positive regulation of gene expression	13	0,000114022	0,001102993	6,80972823
positive regulation of secretion by cell	6	0,000117988	0,001137457	6,778960592
positive regulation of MAP kinase activity	5	0,000120689	0,001159524	6,759745694
peptide transport	6	0,000123885	0,001186179	6,737017885
negative regulation of catalytic activity	9	0,000124954	0,001192355	6,731825352
morphogenesis of an epithelium	7	0,000127396	0,001211553	6,71585223
tissue development	13	0,000128007	0,001213267	6,714438658
regulation of organelle organization	10	0,000128458	0,001213453	6,714285293
response to amino acid	4	0,00013758	0,001293115	6,650701438
morphogenesis of a branching structure	5	0,00013781	0,001293115	6,650701438
epithelial cell proliferation	6	0,000140763	0,001316443	6,632822223
blood vessel morphogenesis	7	0,000147905	0,001373051	6,590720051
post-embryonic camera-type eye development	2	0,00014828	0,001373051	6,590720051
regulation of hydrogen peroxide-mediated programmed cell death	2	0,00014828	0,001373051	6,590720051
intrinsic apoptotic signaling pathway in response to oxidative stress	3	0,000157367	0,001452422	6,534522683
negative regulation of signal transduction	10	0,000157887	0,001452454	6,534500952
single-organism transport	20	0,000160835	0,001474754	6,519264124
respiratory system development	5	0,000163467	0,001494022	6,506283606
signal transduction	25	0,000164724	0,001500641	6,501863116
regulation of secretion	8	0,000165516	0,001502991	6,500298154
positive regulation of metabolic process	19	0,00016857	0,001525804	6,485233828
regulation of macromolecule metabolic process	25	0,000169454	0,001528891	6,483212645
regulation of peptidyl-serine phosphorylation	4	0,000173474	0,001559771	6,463216561
neuron projection development	10	0,000173985	0,001559771	6,463216561
amide transport	6	0,000177311	0,001584541	6,447460655
mesenchymal-epithelial cell signaling	2	0,000190363	0,001695799	6,379601196
positive regulation of secretion	6	0,00019388	0,001721681	6,36445396
extrinsic apoptotic signaling pathway	5	0,00020051	0,00177495	6,333982759
regulation of blood vessel endothelial cell migration	3	0,000203004	0,001791398	6,324758919
lipopolysaccharide-mediated signaling pathway	3	0,000215612	0,001890807	6,270751675
negative regulation of autophagy	3	0,000215612	0,001890807	6,270751675
regulation of metabolic process	27	0,000217332	0,001899966	6,265919215
negative regulation of macromolecule metabolic process	15	0,000228658	0,001992796	6,218216778
positive regulation of growth	5	0,00023003	0,001998564	6,215326216
hepatocyte growth factor receptor signaling pathway	2	0,000237602	0,002045411	6,192156454
hydrogen peroxide-mediated programmed cell death	2	0,000237602	0,002045411	6,192156454
programmed cell death in response to reactive oxygen species	2	0,000237602	0,002045411	6,192156454
positive regulation of myeloid leukocyte differentiation	3	0,000242329	0,002079741	6,175512091
cell communication	26	0,000257203	0,00218889	6,124360916
reactive oxygen species metabolic process	5	0,000257877	0,00218889	6,124360916
regulation of cell growth	6	0,000258157	0,00218889	6,124360916
positive regulation of catabolic process	6	0,000258157	0,00218889	6,124360916
multi-multicellular organism process	5	0,000262758	0,002205958	6,116593468
regulation of behavior	5	0,000262758	0,002205958	6,116593468

anatomical structure morphogenesis	16	0,0002629	0,002205958	6,116593468
wound healing	8	0,000264253	0,002205958	6,116593468
positive regulation of stress-activated MAPK cascade	4	0,000264872	0,002205958	6,116593468
cellular response to reactive oxygen species	4	0,000264872	0,002205958	6,116593468
muscle system process	6	0,000269097	0,002234538	6,103720976
anatomical structure formation involved in morphogenesis	10	0,000271319	0,002246364	6,098442468
positive regulation of stress-activated protein kinase signaling cascade	4	0,000272502	0,002249538	6,097030625
cellular response to hypoxia	4	0,000280289	0,002307054	6,071783909
single-organism metabolic process	24	0,000284943	0,002338525	6,058234846
single-organism localization	20	0,000300105	0,002455803	6,009301283
exocrine system development	3	0,000301992	0,002464079	6,005937046
platelet activation	5	0,000315524	0,002559655	5,967882925
regulation of neuron death	5	0,000315524	0,002559655	5,967882925
positive regulation of cellular component biogenesis	6	0,000329412	0,002660911	5,929086743
response to organonitrogen compound	9	0,000329896	0,002660911	5,929086743
positive regulation of organelle organization	7	0,000331389	0,002665315	5,927432891
cell surface receptor signaling pathway	16	0,000334348	0,00268145	5,921397673
cellular response to decreased oxygen levels	4	0,000339349	0,002713832	5,909393652
surfactant homeostasis	2	0,000347452	0,002770761	5,888633163
negative regulation of signaling	10	0,000351177	0,002792549	5,880800421
negative regulation of MAPK cascade	4	0,000357745	0,002836766	5,865090782
cell-cell signaling	10	0,000365435	0,002889604	5,846635968
adult behavior	4	0,000367206	0,002895473	5,844606767
regulation of glucose import	3	0,000370401	0,002912514	5,838738586
positive regulation of apoptotic process	7	0,000375462	0,002944084	5,827957568
negative regulation of cell communication	10	0,000380177	0,002972774	5,818259688
cardiac muscle cell development	3	0,000388931	0,003032798	5,798269713
positive regulation of programmed cell death	7	0,000395165	0,003072901	5,785133333
positive regulation of macrophage differentiation	2	0,000410018	0,003179618	5,750994079
divalent metal ion transport	6	0,000415313	0,00320303	5,743657986
histone modification	6	0,000415313	0,00320303	5,743657986
cellular response to oxygen levels	4	0,000417219	0,003208935	5,741816038
single-multicellular organism process	25	0,000420346	0,003224176	5,737077722
cardiac cell development	3	0,000427757	0,003272107	5,722321293
divalent inorganic cation transport	6	0,000431194	0,003289458	5,717032395
neuron development	10	0,00044711	0,003398516	5,684416473
regulation of muscle adaptation	3	0,000448069	0,003398516	5,684416473
cellular response to lipopolysaccharide	4	0,000449464	0,003398516	5,684416473
establishment of localization in cell	15	0,000450318	0,003398516	5,684416473
ovarian follicle development	3	0,000468991	0,003529972	5,646465338
regulation of extrinsic apoptotic signaling pathway	4	0,000471926	0,003542591	5,642896835
drug catabolic process	2	0,000477647	0,003557969	5,638565378
chemical homeostasis within a tissue	2	0,000477647	0,003557969	5,638565378
regulation of protein serine/threonine kinase activity	7	0,000477766	0,003557969	5,638565378
regulation of binding	5	0,000482657	0,003584907	5,631022693
covalent chromatin modification	6	0,000487596	0,003612063	5,623476222
neuron differentiation	11	0,000493749	0,003648037	5,613565981
regulation of proteasomal ubiquitin-dependent protein catabolic process	4	0,000495179	0,003649025	5,613295212

regulation of epithelial cell proliferation	5	0,000506481	0,003707803	5,597315841
positive regulation of cell morphogenesis involved in differentiation	4	0,000507106	0,003707803	5,597315841
positive regulation of developmental growth	4	0,000507106	0,003707803	5,597315841
nitric oxide biosynthetic process	3	0,000512695	0,003738951	5,588950234
aging	5	0,000522849	0,003803153	5,571924869
cellular response to molecule of bacterial origin	4	0,000531574	0,003856654	5,557955322
regulation of cell junction assembly	3	0,000535491	0,003875084	5,553188054
regulation of catalytic activity	14	0,000538708	0,003888364	5,549766828
tube development	7	0,000541869	0,003901179	5,546476352
negative regulation of glucose import	2	0,000550316	0,003928287	5,539551795
positive regulation of p38MAPK cascade	2	0,000550316	0,003928287	5,539551795
spongiotrophoblast layer development	2	0,000550316	0,003928287	5,539551795
regulation of primary metabolic process	24	0,000551216	0,003928287	5,539551795
reproductive structure development	6	0,000556144	0,003947953	5,534558114
neuron death	5	0,000556781	0,003947953	5,534558114
regulation of transferase activity	9	0,000564901	0,003995469	5,52259432
reproductive system development	6	0,000582855	0,004092753	5,498537541
branching morphogenesis of an epithelial tube	4	0,000583017	0,004092753	5,498537541
positive regulation of hemopoiesis	4	0,000583017	0,004092753	5,498537541
positive regulation of cell proliferation	8	0,000589006	0,004124507	5,490808806
positive regulation of molecular function	12	0,000600292	0,004193106	5,474313551
positive regulation of neuron death	3	0,000607749	0,004224285	5,466905353
gland development	6	0,000610557	0,004224285	5,466905353
vascular endothelial growth factor receptor signaling pathway	5	0,000610758	0,004224285	5,466905353
intrinsic apoptotic signaling pathway	5	0,000610758	0,004224285	5,466905353
response to nitrogen compound	9	0,000626344	0,004290847	5,451271091
negative regulation of growth of symbiont in host	2	0,000628003	0,004290847	5,451271091
myoblast proliferation	2	0,000628003	0,004290847	5,451271091
positive regulation of vascular endothelial growth factor receptor signaling pathway	2	0,000628003	0,004290847	5,451271091
negative regulation of hydrogen peroxide-induced cell death	2	0,000628003	0,004290847	5,451271091
cellular localization	16	0,000655854	0,004470289	5,410302235
glucose import	3	0,000659218	0,004482366	5,407604157
positive regulation of ERK1 and ERK2 cascade	4	0,000681419	0,004622158	5,376893681
protein localization	14	0,000707881	0,004774649	5,344434903
negative regulation of release of cytochrome c from mitochondria	2	0,000710685	0,004774649	5,344434903
regulation of growth of symbiont in host	2	0,000710685	0,004774649	5,344434903
negative regulation of growth of symbiont involved in interaction with host	2	0,000710685	0,004774649	5,344434903
response to cytokine	8	0,000715683	0,004785432	5,342178963
regulation of MAP kinase activity	6	0,00071569	0,004785432	5,342178963
chemotaxis	8	0,000721176	0,004799316	5,339281878
taxis	8	0,000721176	0,004799316	5,339281878
cellular response to oxygen-containing compound	9	0,000726365	0,004822448	5,334473644
cellular response to biotic stimulus	4	0,000791075	0,005226333	5,25404533
regulation of protein kinase activity	8	0,000795764	0,005226333	5,25404533
modulation of growth of symbiont involved in interaction with host	2	0,000798339	0,005226333	5,25404533
negative regulation of response to reactive oxygen species	2	0,000798339	0,005226333	5,25404533
regulation of neuron projection regeneration	2	0,000798339	0,005226333	5,25404533
post-embryonic organ development	2	0,000798339	0,005226333	5,25404533

cardiac muscle tissue development	4	0,000824569	0,005359331	5,228916118
peptidyl-threonine phosphorylation	3	0,000830078	0,005359331	5,228916118
cellular response to hydrogen peroxide	3	0,000830078	0,005359331	5,228916118
extrinsic apoptotic signaling pathway in absence of ligand	3	0,000830078	0,005359331	5,228916118
signal transduction in absence of ligand	3	0,000830078	0,005359331	5,228916118
positive regulation of histone modification	3	0,000830078	0,005359331	5,228916118
positive regulation of proteasomal ubiquitin-dependent protein catabolic process	3	0,000861027	0,005533766	5,196886596
cellular response to mechanical stimulus	3	0,000861027	0,005533766	5,196886596
regulation of hemopoiesis	5	0,000866903	0,005546207	5,194641009
response to acid chemical	5	0,000866903	0,005546207	5,194641009
regulation of response to external stimulus	8	0,000870054	0,005553747	5,193282383
regulation of myeloid cell differentiation	4	0,000876674	0,005583341	5,187967939
cell growth	6	0,00088972	0,005648654	5,176338012
regulation of hydrogen peroxide-induced cell death	2	0,000890942	0,005648654	5,176338012
response to lipopolysaccharide	5	0,000903813	0,00571738	5,164244706
muscle contraction	5	0,000916374	0,005783812	5,152692317
protein secretion	6	0,000918424	0,005783812	5,152692317
organ regeneration	3	0,000925105	0,005799935	5,149908584
positive regulation of chromatin modification	3	0,000925105	0,005799935	5,149908584
regulation of hormone levels	6	0,000937955	0,005867432	5,138338231
developmental cell growth	4	0,000949713	0,005927807	5,128100927
nitric oxide metabolic process	3	0,000958248	0,005954676	5,123578557
peptidyl-threonine modification	3	0,000958248	0,005954676	5,123578557
regulation of proteasomal protein catabolic process	4	0,000968623	0,006005886	5,115015224
single-organism cellular process	37	0,000972977	0,006010521	5,114243797
organic substance metabolic process	34	0,00097364	0,006010521	5,114243797
myeloid leukocyte differentiation	4	0,000987797	0,006062204	5,105681897
positive regulation of focal adhesion assembly	2	0,000988473	0,006062204	5,105681897
regulation of macrophage differentiation	2	0,000988473	0,006062204	5,105681897
cellular response to endogenous stimulus	10	0,001009275	0,006176325	5,087031863
response to hormone	9	0,001015917	0,006203487	5,082643781
transport	20	0,001029809	0,006274705	5,071228876
epithelial tube morphogenesis	5	0,001035375	0,006294991	5,068001073
response to molecule of bacterial origin	5	0,001063313	0,00645092	5,043532501
organonitrogen compound metabolic process	13	0,001071356	0,0064671	5,041027569
cell differentiation	18	0,001072397	0,0064671	5,041027569
blood circulation	6	0,001072872	0,0064671	5,041027569
negative regulation of lipid catabolic process	2	0,001090909	0,006492406	5,03712216
negative regulation of cytokine production involved in immune response	2	0,001090909	0,006492406	5,03712216
growth of symbiont in host	2	0,001090909	0,006492406	5,03712216
regulation of membrane protein ectodomain proteolysis	2	0,001090909	0,006492406	5,03712216
cell death in response to hydrogen peroxide	2	0,001090909	0,006492406	5,03712216
positive regulation of adherens junction organization	2	0,001090909	0,006492406	5,03712216
reactive nitrogen species metabolic process	3	0,00109833	0,006514476	5,033728428
regulation of cell morphogenesis	6	0,001106033	0,006514476	5,033728428
circulatory system process	6	0,001106033	0,006514476	5,033728428
animal organ development	16	0,001108076	0,006514476	5,033728428
regulation of insulin secretion	4	0,001108502	0,006514476	5,033728428

regulation of muscle system process	4	0,001108502	0,006514476	5,033728428
striated muscle tissue development	5	0,001120871	0,006573443	5,024717543
regulation of kinase activity	8	0,001135185	0,006632281	5,015806473
myeloid cell differentiation	5	0,001135616	0,006632281	5,015806473
negative regulation of apoptotic signaling pathway	4	0,001150943	0,006707879	5,004472539
autophagy	6	0,001163052	0,006764444	4,996075228
positive regulation of protein complex assembly	4	0,001194514	0,006897768	4,976557413
positive regulation of cardiac muscle hypertrophy	2	0,001198227	0,006897768	4,976557413
growth involved in symbiotic interaction	2	0,001198227	0,006897768	4,976557413
growth of symbiont involved in interaction with host	2	0,001198227	0,006897768	4,976557413
positive regulation of muscle hypertrophy	2	0,001198227	0,006897768	4,976557413
reactive oxygen species biosynthetic process	3	0,001211459	0,006959707	4,96761797
positive regulation of transferase activity	7	0,0012278	0,00703922	4,956257939
regulation of purine nucleotide metabolic process	4	0,001239231	0,007090318	4,949025148
macromolecule catabolic process	9	0,001251937	0,007148485	4,940854874
response to lipid	8	0,001259678	0,007178124	4,936717272
phosphatidylinositol-mediated signaling	4	0,001285111	0,007308258	4,918750312
cell redox homeostasis	3	0,001290813	0,007325886	4,916341219
lymph vessel development	2	0,001310405	0,007422114	4,903291293
regulation of biosynthetic process	19	0,001318212	0,007451336	4,899361894
endothelial cell differentiation	3	0,00133169	0,007497413	4,893197215
positive regulation of proteasomal protein catabolic process	3	0,00133169	0,007497413	4,893197215
muscle tissue development	5	0,001340719	0,00753318	4,888438062
inositol lipid-mediated signaling	4	0,001356146	0,007604685	4,878990805
regulation of nucleotide metabolic process	4	0,001380424	0,007725432	4,863237505
muscle organ development	5	0,00139171	0,007773143	4,857080695
response to organic cyclic compound	8	0,001413806	0,007880918	4,843310895
vascular endothelial growth factor signaling pathway	2	0,001427422	0,007925429	4,837678803
positive regulation of cell junction assembly	2	0,001427422	0,007925429	4,837678803
regulation of protein localization to nucleus	4	0,001480582	0,008204405	4,803084077
cardiac muscle cell differentiation	3	0,001503331	0,008314102	4,789802167
establishment of localization	20	0,001533643	0,008451844	4,773370655
regulation of cellular process	33	0,001542547	0,008451844	4,773370655
positive regulation of nucleotide biosynthetic process	3	0,001548305	0,008451844	4,773370655
positive regulation of purine nucleotide biosynthetic process	3	0,001548305	0,008451844	4,773370655
ovulation cycle process	3	0,001548305	0,008451844	4,773370655
skeletal muscle adaptation	2	0,001549255	0,008451844	4,773370655
embryonic hemopoiesis	2	0,001549255	0,008451844	4,773370655
regulation of mitochondrion organization	4	0,001558959	0,008471947	4,770994924
response to mechanical stimulus	4	0,001558959	0,008471947	4,770994924
regulation of cellular protein localization	6	0,001583575	0,008584211	4,757830712
regulation of peptidyl-tyrosine phosphorylation	4	0,001585716	0,008584211	4,757830712
primary metabolic process	33	0,001632814	0,008822209	4,73048298
metal ion homeostasis	6	0,001658597	0,008944348	4,716733427
negative regulation of endothelial cell apoptotic process	2	0,001675881	0,009020278	4,708280134
negative regulation of cytokine production	4	0,001724328	0,009263325	4,681692194
granulocyte chemotaxis	3	0,00173663	0,009311646	4,676489447
proteasome-mediated ubiquitin-dependent protein catabolic process	5	0,001748731	0,009340945	4,673347895

tube morphogenesis	5	0,001748731	0,009340945	4,673347895
regulation of astrocyte differentiation	2	0,00180728	0,009599043	4,646091912
regulation of protein localization to cell surface	2	0,00180728	0,009599043	4,646091912
cell differentiation involved in embryonic placenta development	2	0,00180728	0,009599043	4,646091912
positive regulation of sequence-specific DNA binding transcription factor activity	4	0,001811429	0,009602962	4,6456837
regulation of nucleobase-containing compound metabolic process	18	0,001851004	0,009786271	4,626774766
regulation of apoptotic signaling pathway	5	0,00185296	0,009786271	4,626774766
positive regulation of cell adhesion	5	0,001874343	0,009880664	4,617175516
blood coagulation	6	0,001883173	0,00990866	4,614346114
cell projection organization	10	0,001905114	0,010005402	4,604630132
ubiquitin-dependent protein catabolic process	6	0,001917059	0,010049389	4,600243434
insulin secretion	4	0,001932267	0,010056529	4,599533245
cell-cell junction organization	4	0,001932267	0,010056529	4,599533245
protein ubiquitination	7	0,001934885	0,010056529	4,599533245
myotube differentiation	3	0,001938712	0,010056529	4,599533245
regulation of endothelial cell differentiation	2	0,001943429	0,010056529	4,599533245
negative regulation of production of molecular mediator of immune response	2	0,001943429	0,010056529	4,599533245
regulation of vascular endothelial growth factor receptor signaling pathway	2	0,001943429	0,010056529	4,599533245
hemostasis	6	0,001968758	0,010150281	4,590253913
coagulation	6	0,001968758	0,010150281	4,590253913
extracellular matrix organization	5	0,002052003	0,010555465	4,551111573
modification-dependent protein catabolic process	6	0,002057276	0,010555465	4,551111573
cell junction assembly	4	0,002058597	0,010555465	4,551111573
extracellular structure organization	5	0,002075049	0,010571817	4,549563547
positive regulation of histone acetylation	2	0,002084306	0,010571817	4,549563547
regulation of oxidative stress-induced intrinsic apoptotic signaling pathway	2	0,002084306	0,010571817	4,549563547
regulation of cGMP metabolic process	2	0,002084306	0,010571817	4,549563547
positive regulation of monooxygenase activity	2	0,002084306	0,010571817	4,549563547
cellular macromolecule metabolic process	29	0,002084319	0,010571817	4,549563547
regulation of cell proliferation	10	0,002114257	0,010704376	4,537102637
regulation of myeloid leukocyte differentiation	3	0,002154901	0,01089057	4,519858045
cellular homeostasis	7	0,002204196	0,011096355	4,501138632
modification-dependent macromolecule catabolic process	6	0,002205171	0,011096355	4,501138632
granulocyte migration	3	0,002211193	0,011096355	4,501138632
positive regulation of peptidyl-lysine acetylation	2	0,00222989	0,011096355	4,501138632
endothelium development	3	0,002268393	0,011096355	4,501138632
female gonad development	3	0,002268393	0,011096355	4,501138632
developmental maturation	4	0,002293275	0,011096355	4,501138632
proteasomal protein catabolic process	5	0,002316092	0,011096355	4,501138632
positive regulation of catalytic activity	10	0,002319308	0,011096355	4,501138632
negative regulation of branching involved in lung morphogenesis	1	0,002341639	0,011096355	4,501138632
glycogen cell differentiation involved in embryonic placenta development	1	0,002341639	0,011096355	4,501138632
positive regulation of adrenergic receptor signaling pathway involved in heart process	1	0,002341639	0,011096355	4,501138632
positive regulation of Rho guanyl-nucleotide exchange factor activity	1	0,002341639	0,011096355	4,501138632
flavin adenine dinucleotide catabolic process	1	0,002341639	0,011096355	4,501138632
positive regulation of protein K63-linked deubiquitination	1	0,002341639	0,011096355	4,501138632
positive regulation of Lys63-specific deubiquitinase activity	1	0,002341639	0,011096355	4,501138632
negative regulation of T cell antigen processing and presentation	1	0,002341639	0,011096355	4,501138632

cellular response to iron ion starvation	1	0,002341639	0,011096355	4,501138632
negative regulation of antigen processing and presentation of endogenous peptide antigen via MHC class I	1	0,002341639	0,011096355	4,501138632
response to iron ion starvation	1	0,002341639	0,011096355	4,501138632
initiation of primordial ovarian follicle growth	1	0,002341639	0,011096355	4,501138632
phosphocreatine biosynthetic process	1	0,002341639	0,011096355	4,501138632
basophil chemotaxis	1	0,002341639	0,011096355	4,501138632
positive regulation of protein kinase D signaling	1	0,002341639	0,011096355	4,501138632
negative regulation of cellular organofluorine metabolic process	1	0,002341639	0,011096355	4,501138632
positive regulation of histone H3-K27 acetylation	1	0,002341639	0,011096355	4,501138632
flavin-containing compound catabolic process	1	0,002341639	0,011096355	4,501138632
regulation of protein K63-linked deubiquitination	1	0,002341639	0,011096355	4,501138632
positive regulation of ubiquitin-specific protease activity	1	0,002341639	0,011096355	4,501138632
regulation of antigen processing and presentation of endogenous peptide antigen via MHC class I	1	0,002341639	0,011096355	4,501138632
phosphagen metabolic process	1	0,002341639	0,011096355	4,501138632
phosphocreatine metabolic process	1	0,002341639	0,011096355	4,501138632
phosphagen biosynthetic process	1	0,002341639	0,011096355	4,501138632
regulation of protein kinase D signaling	1	0,002341639	0,011096355	4,501138632
regulation of cellular organohalogen metabolic process	1	0,002341639	0,011096355	4,501138632
regulation of cellular organofluorine metabolic process	1	0,002341639	0,011096355	4,501138632
negative regulation of cellular organohalogen metabolic process	1	0,002341639	0,011096355	4,501138632
cellular developmental process	18	0,002345411	0,011096355	4,501138632
metal ion transport	7	0,002349369	0,011096435	4,501131411
developmental growth	6	0,002361012	0,01112981	4,498128157
divalent inorganic cation homeostasis	5	0,002366658	0,01112981	4,498128157
organic substance catabolic process	11	0,002372317	0,01112981	4,498128157
regulation of the force of heart contraction	2	0,002380158	0,01112981	4,498128157
negative regulation of insulin receptor signaling pathway	2	0,002380158	0,01112981	4,498128157
positive regulation of mesenchymal cell proliferation	2	0,002380158	0,01112981	4,498128157
cellular component morphogenesis	10	0,002433577	0,011360727	4,477592891
regulation of protein secretion	5	0,002444015	0,011378612	4,47601981
positive regulation of endocytosis	3	0,002445492	0,011378612	4,47601981
epithelial cell differentiation	6	0,002504107	0,011632116	4,453985414
negative regulation of extrinsic apoptotic signaling pathway in absence of ligand	2	0,00253509	0,011679672	4,449905367
maternal placenta development	2	0,00253509	0,011679672	4,449905367
negative regulation of signal transduction in absence of ligand	2	0,00253509	0,011679672	4,449905367
negative regulation of anion transport	2	0,00253509	0,011679672	4,449905367
histone phosphorylation	2	0,00253509	0,011679672	4,449905367
cation homeostasis	6	0,002546173	0,011711565	4,447178476
development of primary female sexual characteristics	3	0,002568192	0,011793572	4,440200654
chromatin modification	6	0,002610273	0,011967294	4,425577836
cardiocyte differentiation	3	0,002630946	0,012042461	4,419316439
response to interleukin-1	3	0,002694644	0,012271698	4,400459635
cell migration involved in sprouting angiogenesis	2	0,002694664	0,012271698	4,400459635
negative regulation of cellular response to insulin stimulus	2	0,002694664	0,012271698	4,400459635
regulation of proteolysis involved in cellular protein catabolic process	4	0,002698466	0,012271698	4,400459635
macromolecule localization	14	0,002783488	0,012637935	4,371052237
ovulation cycle	3	0,002824887	0,012805245	4,357900412
inorganic ion homeostasis	6	0,00283279	0,012820424	4,35671572

positive regulation of protein kinase activity	6	0,002855805	0,0128763	4,352366877
regulation of myelination	2	0,002858859	0,0128763	4,352366877
negative regulation of epithelial cell apoptotic process	2	0,002858859	0,0128763	4,352366877
positive regulation of protein localization to nucleus	3	0,002958959	0,013305864	4,31955045
necroptotic process	2	0,003027652	0,013528318	4,302970127
adult walking behavior	2	0,003027652	0,013528318	4,302970127
walking behavior	2	0,003027652	0,013528318	4,302970127
positive regulation of protein acetylation	2	0,003027652	0,013528318	4,302970127
positive regulation of protein catabolic process	4	0,003064676	0,01367205	4,29240167
regulation of epithelial cell differentiation	3	0,003096898	0,013793936	4,283526235
cell junction organization	4	0,003107361	0,013818677	4,2817342
heart contraction	4	0,003150453	0,013988209	4,269540516
positive regulation of axon extension	2	0,003201023	0,01416805	4,256765825
drug metabolic process	2	0,003201023	0,01416805	4,256765825
regulation of cellular biosynthetic process	18	0,0032211	0,014223166	4,25288326
heart process	4	0,003237861	0,014223166	4,25288326
positive regulation of nucleotide metabolic process	3	0,003238739	0,014223166	4,25288326
positive regulation of purine nucleotide metabolic process	3	0,003238739	0,014223166	4,25288326
response to hydrogen peroxide	3	0,003238739	0,014223166	4,25288326
regulation of histone modification	3	0,003311134	0,014518444	4,232335432
proteolysis involved in cellular protein catabolic process	6	0,003320484	0,014536802	4,231071765
positive regulation of sodium ion transport	2	0,003378951	0,014701307	4,21981886
hyaluronan metabolic process	2	0,003378951	0,014701307	4,21981886
insulin-like growth factor receptor signaling pathway	2	0,003378951	0,014701307	4,21981886
regulation of cardiac muscle hypertrophy	2	0,003378951	0,014701307	4,21981886
negative regulation of protein modification process	6	0,00342515	0,014879317	4,207783145
regulation of cell adhesion	6	0,003451699	0,014956665	4,202598282
embryonic organ development	5	0,003455015	0,014956665	4,202598282
negative regulation of binding	3	0,003458895	0,014956665	4,202598282
positive regulation of kinase activity	6	0,003478403	0,01501795	4,198509095
posttranscriptional regulation of gene expression	5	0,003522617	0,015185555	4,187410619
positive regulation of macroautophagy	2	0,003561413	0,015305921	4,179515522
regulation of mesenchymal cell proliferation	2	0,003561413	0,015305921	4,179515522
regulation of cellular protein catabolic process	4	0,003604119	0,01543873	4,170875985
gland morphogenesis	3	0,003610646	0,01543873	4,170875985
female sex differentiation	3	0,003610646	0,01543873	4,170875985
inflammatory response	6	0,003614253	0,01543873	4,170875985
negative regulation of protein catabolic process	3	0,003688029	0,015730002	4,152185451
developmental process involved in reproduction	6	0,003725768	0,015866923	4,143518678
membrane protein ectodomain proteolysis	2	0,00374839	0,015915111	4,140486272
regulation of muscle hypertrophy	2	0,00374839	0,015915111	4,140486272
positive regulation of leukocyte differentiation	3	0,003766422	0,015919635	4,140201998
regulation of nucleotide biosynthetic process	3	0,003766422	0,015919635	4,140201998
regulation of purine nucleotide biosynthetic process	3	0,003766422	0,015919635	4,140201998
epithelium development	8	0,003776702	0,015939156	4,138976531
nucleobase-containing compound biosynthetic process	18	0,003849464	0,016221917	4,121392078
regulation of nucleic acid-templated transcription	16	0,003923759	0,01649613	4,104629472
regulation of mRNA stability	3	0,003926255	0,01649613	4,104629472

response to tumor necrosis factor	4	0,004048864	0,016960643	4,076859715
cellular response to external stimulus	4	0,004048864	0,016960643	4,076859715
cell proliferation	11	0,004086596	0,017093264	4,069070796
programmed necrotic cell death	2	0,004135802	0,01717151	4,064503674
negative regulation of mitochondrion organization	2	0,004135802	0,01717151	4,064503674
striated muscle adaptation	2	0,004135802	0,01717151	4,064503674
positive regulation of cell-matrix adhesion	2	0,004135802	0,01717151	4,064503674
regulation of endothelial cell apoptotic process	2	0,004135802	0,01717151	4,064503674
regulation of RNA biosynthetic process	16	0,00415065	0,017207774	4,062394013
activation of MAPK activity	3	0,004173683	0,017277819	4,058331712
protein modification by small protein conjugation	7	0,004194846	0,017339928	4,054743438
cellular protein catabolic process	6	0,004228681	0,017454158	4,048177364
regulation of RNA stability	3	0,004258222	0,01755036	4,042680799
regulation of nitrogen compound metabolic process	18	0,004304715	0,017716041	4,033284756
myoblast fusion	2	0,004336196	0,017793573	4,028917939
positive regulation of kidney development	2	0,004336196	0,017793573	4,028917939
liver development	3	0,0043438	0,017798832	4,028622437
regulation of chromatin modification	3	0,00443042	0,01807483	4,013234918
positive regulation of chromosome organization	3	0,00443042	0,01807483	4,013234918
kidney epithelium development	3	0,00443042	0,01807483	4,013234918
ion homeostasis	6	0,004482129	0,018213538	4,005590124
positive regulation of glucose transport	2	0,00454102	0,018213538	4,005590124
heterocycle biosynthetic process	18	0,0046042	0,018213538	4,005590124
hepaticobiliary system development	3	0,004606801	0,018213538	4,005590124
macromolecular complex subunit organization	13	0,004631109	0,018213538	4,005590124
aromatic compound biosynthetic process	18	0,004653723	0,018213538	4,005590124
calcium-dependent cell-matrix adhesion	1	0,004677935	0,018213538	4,005590124
positive regulation of chronic inflammatory response to antigenic stimulus	1	0,004677935	0,018213538	4,005590124
positive regulation of translational initiation by iron	1	0,004677935	0,018213538	4,005590124
negative regulation of adrenergic receptor signaling pathway involved in heart process	1	0,004677935	0,018213538	4,005590124
positive regulation of dense core granule biogenesis	1	0,004677935	0,018213538	4,005590124
fasciculation of motor neuron axon	1	0,004677935	0,018213538	4,005590124
positive regulation of ferrous iron import into cell	1	0,004677935	0,018213538	4,005590124
positive regulation of ferrous iron binding	1	0,004677935	0,018213538	4,005590124
positive regulation of transferrin receptor binding	1	0,004677935	0,018213538	4,005590124
negative regulation of CD8-positive, alpha-beta T cell activation	1	0,004677935	0,018213538	4,005590124
coronary vein morphogenesis	1	0,004677935	0,018213538	4,005590124
VEGF-activated neuropilin signaling pathway	1	0,004677935	0,018213538	4,005590124
positive regulation of retinal ganglion cell axon guidance	1	0,004677935	0,018213538	4,005590124
regulation of adrenergic receptor signaling pathway involved in heart process	1	0,004677935	0,018213538	4,005590124
regulation of Rho guanyl-nucleotide exchange factor activity	1	0,004677935	0,018213538	4,005590124
flavin adenine dinucleotide metabolic process	1	0,004677935	0,018213538	4,005590124
positive regulation of protein deubiquitination	1	0,004677935	0,018213538	4,005590124
regulation of T cell antigen processing and presentation	1	0,004677935	0,018213538	4,005590124
positive regulation of iron ion transport	1	0,004677935	0,018213538	4,005590124
regulation of iron ion import	1	0,004677935	0,018213538	4,005590124
regulation of ferrous iron import into cell	1	0,004677935	0,018213538	4,005590124
negative regulation of antigen processing and presentation of peptide antigen via MHC class I	1	0,004677935	0,018213538	4,005590124

regulation of ferrous iron binding	1	0,004677935	0,018213538	4,005590124
regulation of transferrin receptor binding	1	0,004677935	0,018213538	4,005590124
neuropilin signaling pathway	1	0,004677935	0,018213538	4,005590124
cellular organohalogen metabolic process	1	0,004677935	0,018213538	4,005590124
cellular organofluorine metabolic process	1	0,004677935	0,018213538	4,005590124
cellular response to abiotic stimulus	4	0,004697976	0,018266303	4,0026973
positive regulation of nitric oxide biosynthetic process	2	0,004750253	0,018292699	4,001253244
mitotic nuclear envelope disassembly	2	0,004750253	0,018292699	4,001253244
peptidyl-tyrosine autophosphorylation	2	0,004750253	0,018292699	4,001253244
positive regulation of nitric oxide metabolic process	2	0,004750253	0,018292699	4,001253244
regulation of release of cytochrome c from mitochondria	2	0,004750253	0,018292699	4,001253244
positive regulation of response to extracellular stimulus	2	0,004750253	0,018292699	4,001253244
positive regulation of response to nutrient levels	2	0,004750253	0,018292699	4,001253244
neuron projection extension	3	0,004879282	0,018763907	3,975820072
neuromuscular junction development	2	0,004963876	0,018965903	3,965112479
import into cell	2	0,004963876	0,018965903	3,965112479
regulation of insulin receptor signaling pathway	2	0,004963876	0,018965903	3,965112479
endothelial cell apoptotic process	2	0,004963876	0,018965903	3,965112479
striated muscle contraction	3	0,004972233	0,018965903	3,965112479
histone acetylation	3	0,004972233	0,018965903	3,965112479
system process	11	0,005045305	0,019218586	3,951877461
regulation of chromatin organization	3	0,005066252	0,019272296	3,949086672
positive regulation of receptor-mediated endocytosis	2	0,005181867	0,01955356	3,934597931
regulation of extrinsic apoptotic signaling pathway in absence of ligand	2	0,005181867	0,01955356	3,934597931
cGMP metabolic process	2	0,005181867	0,01955356	3,934597931
membrane disassembly	2	0,005181867	0,01955356	3,934597931
nuclear envelope disassembly	2	0,005181867	0,01955356	3,934597931
mesenchymal cell proliferation	2	0,005181867	0,01955356	3,934597931
regulation of developmental growth	4	0,005228608	0,019703524	3,926957797
regulation of cyclic nucleotide metabolic process	3	0,005257506	0,019784831	3,922839759
cellular metal ion homeostasis	5	0,005264241	0,019784831	3,922839759
regulation of blood circulation	4	0,00528995	0,019854944	3,919302218
central nervous system development	7	0,005300656	0,019856788	3,919209357
regulation of macromolecule biosynthetic process	17	0,005304549	0,019856788	3,919209357
placenta development	3	0,005354749	0,020018086	3,911119099
positive regulation of oxidoreductase activity	2	0,005404206	0,020176179	3,903252607
internal peptidyl-lysine acetylation	3	0,005453074	0,020304765	3,896899685
DNA biosynthetic process	3	0,005453074	0,020304765	3,896899685
cell maturation	3	0,005552483	0,020647609	3,880155749
regulation of RNA metabolic process	16	0,005566465	0,020672294	3,87896093
lung alveolus development	2	0,005630872	0,020801712	3,872719986
protein localization to cell surface	2	0,005630872	0,020801712	3,872719986
regulation of focal adhesion assembly	2	0,005630872	0,020801712	3,872719986
regulation of cell-substrate junction assembly	2	0,005630872	0,020801712	3,872719986
camera-type eye development	4	0,005668199	0,020912161	3,8674244
regulation of reactive oxygen species metabolic process	3	0,005857254	0,021485799	3,840363059
regulation of JNK cascade	3	0,005857254	0,021485799	3,840363059
myeloid leukocyte migration	3	0,005857254	0,021485799	3,840363059

necrotic cell death	2	0,005861845	0,021485799	3,840363059
neuron projection regeneration	2	0,005861845	0,021485799	3,840363059
cell morphogenesis	9	0,005906254	0,021620423	3,834116922
positive regulation of immune system process	7	0,005947246	0,021742203	3,828500066
peptidyl-lysine acetylation	3	0,005961036	0,021764355	3,827481747
multi-organism reproductive process	7	0,005981072	0,021809219	3,825422531
organ morphogenesis	7	0,00604916	0,02202896	3,815397308
internal protein amino acid acetylation	3	0,00606592	0,022061452	3,813923438
protein import into nucleus, translocation	2	0,006097105	0,022117719	3,811376229
regulation of adherens junction organization	2	0,006097105	0,022117719	3,811376229
positive regulation of peptidyl-tyrosine phosphorylation	3	0,006171907	0,022360256	3,800470178
regulation of body fluid levels	6	0,006205872	0,022454409	3,796268276
regulation of axonogenesis	3	0,006279002	0,022689847	3,785837702
regulation of cellular component biogenesis	6	0,006288659	0,02269561	3,785583753
negative regulation of ERK1 and ERK2 cascade	2	0,006336631	0,022781118	3,781823233
positive regulation of reactive oxygen species biosynthetic process	2	0,006336631	0,022781118	3,781823233
regulation of histone acetylation	2	0,006336631	0,022781118	3,781823233
nucleic acid-templated transcription	16	0,006396012	0,022965272	3,773772132
organic cyclic compound biosynthetic process	18	0,006413498	0,022998721	3,77231666
regulation of cell size	3	0,006496526	0,023266821	3,760726908
hemopoiesis	6	0,006541897	0,023399542	3,755038819
response to nicotine	2	0,006580403	0,023418248	3,75423974
positive regulation of neuron apoptotic process	2	0,006580403	0,023418248	3,75423974
regulation of lipid catabolic process	2	0,006580403	0,023418248	3,75423974
syncytium formation by plasma membrane fusion	2	0,006580403	0,023418248	3,75423974
skeletal muscle tissue development	3	0,006606962	0,023483077	3,751475263
cellular response to organic cyclic compound	5	0,0066495	0,023604467	3,7463193
response to heat	3	0,006831193	0,024061042	3,727161263
positive regulation of vitamin D biosynthetic process	1	0,0070089	0,024061042	3,727161263
positive regulation of calcidiol 1-monooxygenase activity	1	0,0070089	0,024061042	3,727161263
positive regulation of mononuclear cell migration	1	0,0070089	0,024061042	3,727161263
positive regulation of superoxide dismutase activity	1	0,0070089	0,024061042	3,727161263
positive regulation of endodeoxyribonuclease activity	1	0,0070089	0,024061042	3,727161263
response to insulin-like growth factor stimulus	1	0,0070089	0,024061042	3,727161263
fasciculation of sensory neuron axon	1	0,0070089	0,024061042	3,727161263
positive regulation of cell proliferation by VEGF-activated platelet derived growth factor receptor signaling pathway	1	0,0070089	0,024061042	3,727161263
primitive erythrocyte differentiation	1	0,0070089	0,024061042	3,727161263
cardiac vascular smooth muscle cell development	1	0,0070089	0,024061042	3,727161263
positive regulation of peptidyl-tyrosine autophosphorylation	1	0,0070089	0,024061042	3,727161263
alkaloid catabolic process	1	0,0070089	0,024061042	3,727161263
isoquinoline alkaloid metabolic process	1	0,0070089	0,024061042	3,727161263
regulation of chronic inflammatory response to antigenic stimulus	1	0,0070089	0,024061042	3,727161263
regulation of translational initiation by iron	1	0,0070089	0,024061042	3,727161263
positive regulation of vitamin metabolic process	1	0,0070089	0,024061042	3,727161263
positive regulation of removal of superoxide radicals	1	0,0070089	0,024061042	3,727161263
negative regulation of adrenergic receptor signaling pathway	1	0,0070089	0,024061042	3,727161263
positive regulation of adrenergic receptor signaling pathway	1	0,0070089	0,024061042	3,727161263
regulation of dense core granule biogenesis	1	0,0070089	0,024061042	3,727161263

regulation of ubiquitin-specific protease activity	1	0,0070089	0,024061042	3,727161263
regulation of iron ion transport	1	0,0070089	0,024061042	3,727161263
VEGF-activated platelet-derived growth factor receptor signaling pathway	1	0,0070089	0,024061042	3,727161263
protein kinase D signaling	1	0,0070089	0,024061042	3,727161263
histone H3-K27 acetylation	1	0,0070089	0,024061042	3,727161263
regulation of histone H3-K27 acetylation	1	0,0070089	0,024061042	3,727161263
cellular response to lipid	5	0,007032497	0,024092776	3,72584324
cellular nitrogen compound biosynthetic process	19	0,007035262	0,024092776	3,72584324
acute-phase response	2	0,007080605	0,024218593	3,720634615
vascular process in circulatory system	3	0,007175985	0,024515046	3,708468239
cellular cation homeostasis	5	0,007258204	0,024764556	3,698341848
heart development	5	0,007315418	0,024764556	3,698341848
membrane protein proteolysis	2	0,007336995	0,024764556	3,698341848
endothelial cell development	2	0,007336995	0,024764556	3,698341848
positive regulation of cyclase activity	2	0,007336995	0,024764556	3,698341848
positive regulation of lyase activity	2	0,007336995	0,024764556	3,698341848
regulation of peptidyl-lysine acetylation	2	0,007336995	0,024764556	3,698341848
muscle fiber development	2	0,007336995	0,024764556	3,698341848
regulation of mitochondrial membrane potential	2	0,007336995	0,024764556	3,698341848
cellular modified amino acid biosynthetic process	2	0,007336995	0,024764556	3,698341848
regulation of cell morphogenesis involved in differentiation	4	0,007362334	0,024820322	3,696092512
negative regulation of neuron death	3	0,007411509	0,024926402	3,69182773
positive regulation of proteolysis involved in cellular protein catabolic process	3	0,007411509	0,024926402	3,69182773
response to steroid hormone	5	0,007547454	0,02535332	3,674845609
regulation of DNA metabolic process	4	0,00759521	0,02537023	3,674178847
regulation of nitric oxide biosynthetic process	2	0,00759755	0,02537023	3,674178847
syncytium formation	2	0,00759755	0,02537023	3,674178847
epithelial cell morphogenesis	2	0,00759755	0,02537023	3,674178847
regulation of kidney development	2	0,00759755	0,02537023	3,674178847
chromatin organization	6	0,007726511	0,025770295	3,658532807
peptidyl-proline modification	2	0,007862252	0,026191999	3,642301306
negative regulation of secretion by cell	3	0,007896245	0,026268591	3,639381301
endomembrane system organization	5	0,00790514	0,026268591	3,639381301
response to metal ion	4	0,007913238	0,026268591	3,639381301
skeletal muscle organ development	3	0,008020296	0,026583425	3,627467383
cellular ion homeostasis	5	0,008026967	0,026583425	3,627467383
multicellular organismal homeostasis	4	0,008075506	0,026712749	3,622614337
negative regulation of multicellular organismal process	7	0,008118956	0,026770748	3,620445485
regulation of cytokine production involved in immune response	2	0,00813108	0,026770748	3,620445485
lung morphogenesis	2	0,00813108	0,026770748	3,620445485
release of cytochrome c from mitochondria	2	0,00813108	0,026770748	3,620445485
positive regulation of DNA metabolic process	3	0,008145498	0,026786889	3,619842743
protein modification by small protein conjugation or removal	7	0,008377958	0,027519197	3,592871462
cardiac muscle hypertrophy	2	0,008404014	0,027540512	3,592097203
regulation of epithelial cell apoptotic process	2	0,008404014	0,027540512	3,592097203
regulation of immune system process	9	0,00851887	0,027882036	3,579772661
leukocyte chemotaxis	3	0,00852804	0,027882036	3,579772661
protein localization to nucleus	4	0,00857547	0,02797213	3,576546628

positive regulation of cellular protein localization	4	0,00857547	0,02797213	3,576546628
negative regulation of phosphorus metabolic process	5	0,008655903	0,028088637	3,572390166
negative regulation of phosphate metabolic process	5	0,008655903	0,028088637	3,572390166
histone H3 acetylation	2	0,008681035	0,028088637	3,572390166
regulation of pathway-restricted SMAD protein phosphorylation	2	0,008681035	0,028088637	3,572390166
regulation of monooxygenase activity	2	0,008681035	0,028088637	3,572390166
regulation of cellular response to insulin stimulus	2	0,008681035	0,028088637	3,572390166
regulation of glial cell differentiation	2	0,008681035	0,028088637	3,572390166
glial cell differentiation	3	0,008788872	0,028381946	3,562002044
hematopoietic or lymphoid organ development	6	0,00879185	0,028381946	3,562002044
RNA biosynthetic process	16	0,008837153	0,028495516	3,558008543
striated muscle hypertrophy	2	0,008962123	0,028717756	3,550239655
macromolecule metabolic process	29	0,009022879	0,028717756	3,550239655
protein acetylation	3	0,009054367	0,028717756	3,550239655
eye development	4	0,009095426	0,028717756	3,550239655
cation transport	7	0,009098786	0,028717756	3,550239655
multicellular organismal reproductive process	6	0,009224425	0,028717756	3,550239655
positive regulation of phosphatidylinositol 3-kinase signaling	2	0,009247259	0,028717756	3,550239655
maternal process involved in female pregnancy	2	0,009247259	0,028717756	3,550239655
response to bacterium	5	0,009250695	0,028717756	3,550239655
positive regulation of cellular protein catabolic process	3	0,009324545	0,028717756	3,550239655
cellular magnesium ion homeostasis	1	0,009334546	0,028717756	3,550239655
negative regulation of alkaline phosphatase activity	1	0,009334546	0,028717756	3,550239655
negative regulation of bicellular tight junction assembly	1	0,009334546	0,028717756	3,550239655
negative regulation of plasma membrane long-chain fatty acid transport	1	0,009334546	0,028717756	3,550239655
negative regulation of serotonin uptake	1	0,009334546	0,028717756	3,550239655
regulation of generation of L-type calcium current	1	0,009334546	0,028717756	3,550239655
response to electrical stimulus involved in regulation of muscle adaptation	1	0,009334546	0,028717756	3,550239655
desmosome assembly	1	0,009334546	0,028717756	3,550239655
negative regulation of axon regeneration	1	0,009334546	0,028717756	3,550239655
positive regulation of cell growth involved in cardiac muscle cell development	1	0,009334546	0,028717756	3,550239655
extraocular skeletal muscle development	1	0,009334546	0,028717756	3,550239655
positive regulation of mitochondrial membrane potential	1	0,009334546	0,028717756	3,550239655
negative regulation of metalloendopeptidase activity	1	0,009334546	0,028717756	3,550239655
regulation of retinal ganglion cell axon guidance	1	0,009334546	0,028717756	3,550239655
positive regulation of histone deacetylase activity	1	0,009334546	0,028717756	3,550239655
post-embryonic camera-type eye morphogenesis	1	0,009334546	0,028717756	3,550239655
leukemia inhibitory factor signaling pathway	1	0,009334546	0,028717756	3,550239655
positive regulation of mesenchymal to epithelial transition involved in metanephros morphogenesis	1	0,009334546	0,028717756	3,550239655
positive regulation of chronic inflammatory response	1	0,009334546	0,028717756	3,550239655
regulation of superoxide dismutase activity	1	0,009334546	0,028717756	3,550239655
regulation of plasma membrane long-chain fatty acid transport	1	0,009334546	0,028717756	3,550239655
positive regulation of deoxyribonuclease activity	1	0,009334546	0,028717756	3,550239655
negative regulation of neurotransmitter uptake	1	0,009334546	0,028717756	3,550239655
regulation of serotonin uptake	1	0,009334546	0,028717756	3,550239655
adrenergic receptor signaling pathway involved in heart process	1	0,009334546	0,028717756	3,550239655
generation of L-type calcium current	1	0,009334546	0,028717756	3,550239655
dense core granule biogenesis	1	0,009334546	0,028717756	3,550239655

negative regulation of neuron projection regeneration	1	0,009334546	0,028717756	3,550239655
regulation of metalloendopeptidase activity	1	0,009334546	0,028717756	3,550239655
ferrous iron import into cell	1	0,009334546	0,028717756	3,550239655
negative regulation of antigen processing and presentation of peptide antigen	1	0,009334546	0,028717756	3,550239655
regulation of peptidyl-tyrosine autophosphorylation	1	0,009334546	0,028717756	3,550239655
regulation of protein complex assembly	4	0,009362999	0,028742468	3,549379518
leukocyte migration	4	0,009362999	0,028742468	3,549379518
regulation of protein acetylation	2	0,009536423	0,029171536	3,534561856
muscle hypertrophy	2	0,009536423	0,029171536	3,534561856
pathway-restricted SMAD protein phosphorylation	2	0,009536423	0,029171536	3,534561856
positive regulation of proteolysis	4	0,009544222	0,029171536	3,534561856
regulation of transcription, DNA-templated	15	0,009777711	0,029852772	3,51147757
positive regulation of JUN kinase activity	2	0,009829596	0,029946226	3,508351962
positive regulation of response to DNA damage stimulus	2	0,009829596	0,029946226	3,508351962
JNK cascade	3	0,009879017	0,03006425	3,504418505
cellular response to DNA damage stimulus	6	0,009901876	0,030101275	3,503187756
regulation of gene expression	17	0,010007648	0,030389192	3,493668266
nitrogen compound transport	6	0,010018178	0,030389192	3,493668266
response to axon injury	2	0,010126758	0,030619575	3,486115769
astrocyte differentiation	2	0,010126758	0,030619575	3,486115769
regulation of neurotransmitter transport	2	0,010126758	0,030619575	3,486115769
cyclic nucleotide metabolic process	3	0,010163344	0,030697224	3,483583054
cell morphogenesis involved in differentiation	7	0,010265213	0,030971675	3,474682186
regulation of sequence-specific DNA binding transcription factor activity	4	0,010388162	0,031309075	3,463847294
muscle cell migration	2	0,01042789	0,031361657	3,462169237
sprouting angiogenesis	2	0,01042789	0,031361657	3,462169237
exocytosis	4	0,010484834	0,03149926	3,457791232
response to nutrient	3	0,010598744	0,03180753	3,44805222
multicellular organism reproduction	6	0,0106145	0,03182089	3,447632273
positive regulation of axonogenesis	2	0,010732973	0,032039576	3,440783404
regulation of vasoconstriction	2	0,010732973	0,032039576	3,440783404
demethylation	2	0,010732973	0,032039576	3,440783404
positive regulation of stem cell proliferation	2	0,010732973	0,032039576	3,440783404
cell-matrix adhesion	3	0,01074626	0,032045257	3,440606087
positive regulation of lipid biosynthetic process	2	0,011041988	0,032822805	3,416631745
regulation of neurological system process	2	0,011041988	0,032822805	3,416631745
regulation of DNA biosynthetic process	2	0,011041988	0,032822805	3,416631745
negative regulation of secretion	3	0,011195987	0,033175476	3,405944357
regulation of endocytosis	3	0,011195987	0,033175476	3,405944357
regulation of neuron apoptotic process	3	0,011195987	0,033175476	3,405944357
oxidation-reduction process	7	0,011210026	0,033182148	3,405743259
calcium ion homeostasis	4	0,011279383	0,033352377	3,400626235
regulation of reactive oxygen species biosynthetic process	2	0,011354915	0,033470247	3,397098384
cell-substrate adherens junction assembly	2	0,011354915	0,033470247	3,397098384
focal adhesion assembly	2	0,011354915	0,033470247	3,397098384
calcium ion transport	4	0,011381369	0,03351313	3,39581797
immune system development	6	0,011491639	0,033684291	3,390723693
chronic inflammatory response to antigenic stimulus	1	0,011654884	0,033684291	3,390723693

positive regulation of humoral immune response mediated by circulating immunoglobulin	1	0,011654884	0,033684291	3,390723693
negative regulation of myosin-light-chain-phosphatase activity	1	0,011654884	0,033684291	3,390723693
positive regulation of chemokine (C-X-C motif) ligand 2 production	1	0,011654884	0,033684291	3,390723693
negative regulation of fatty acid beta-oxidation	1	0,011654884	0,033684291	3,390723693
maintenance of protein location in mitochondrion	1	0,011654884	0,033684291	3,390723693
positive regulation of the force of heart contraction	1	0,011654884	0,033684291	3,390723693
aggresome assembly	1	0,011654884	0,033684291	3,390723693
positive regulation of ATP biosynthetic process	1	0,011654884	0,033684291	3,390723693
positive regulation of transcription from RNA polymerase II promoter in response to hypoxia	1	0,011654884	0,033684291	3,390723693
positive regulation of protein localization to early endosome	1	0,011654884	0,033684291	3,390723693
spongiotrophoblast differentiation	1	0,011654884	0,033684291	3,390723693
regulation of RNA polymerase II regulatory region sequence-specific DNA binding	1	0,011654884	0,033684291	3,390723693
plasma membrane long-chain fatty acid transport	1	0,011654884	0,033684291	3,390723693
serotonin uptake	1	0,011654884	0,033684291	3,390723693
ferrous iron import	1	0,011654884	0,033684291	3,390723693
regulation of protein localization to early endosome	1	0,011654884	0,033684291	3,390723693
regulation of establishment of protein localization	6	0,011882993	0,034308332	3,372367026
muscle organ morphogenesis	2	0,011992431	0,034518091	3,366271707
cytokine production involved in immune response	2	0,011992431	0,034518091	3,366271707
positive regulation of autophagy	2	0,011992431	0,034518091	3,366271707
cellular divalent inorganic cation homeostasis	4	0,012112057	0,034826802	3,357368011
vasculogenesis	2	0,012316982	0,035379902	3,341611356
response to peptide hormone	5	0,012488302	0,035835444	3,328817818
regulation of cell-cell adhesion	4	0,012652129	0,036231682	3,317821361
negative regulation of protein phosphorylation	4	0,012652129	0,036231682	3,317821361
positive regulation of hydrolase activity	6	0,012763443	0,036504502	3,310319677
osteoblast differentiation	3	0,012773334	0,036504502	3,310319677
organic substance transport	12	0,012803434	0,036553416	3,308980649
negative regulation of hormone secretion	2	0,012977575	0,036975582	3,297497542
cellular response to ketone	2	0,012977575	0,036975582	3,297497542
nuclear envelope organization	2	0,01331358	0,037875762	3,273443893
regulation of vesicle-mediated transport	4	0,013320428	0,037875762	3,273443893
chromosome organization	7	0,013351167	0,037924858	3,272148488
organonitrogen compound biosynthetic process	8	0,013410882	0,038056081	3,268694394
developmental growth involved in morphogenesis	3	0,01360751	0,038365445	3,260598088
protein acylation	3	0,01360751	0,038365445	3,260598088
negative regulation of protein binding	2	0,013653366	0,038365445	3,260598088
negative regulation of cellular component organization	5	0,013958576	0,038365445	3,260598088
negative regulation of cytokine secretion involved in immune response	1	0,013969927	0,038365445	3,260598088
epithelial cell proliferation involved in salivary gland morphogenesis	1	0,013969927	0,038365445	3,260598088
necroptotic signaling pathway	1	0,013969927	0,038365445	3,260598088
response to UV-A	1	0,013969927	0,038365445	3,260598088
corticospinal tract morphogenesis	1	0,013969927	0,038365445	3,260598088
regulation of protein complex stability	1	0,013969927	0,038365445	3,260598088
positive regulation of neuron projection regeneration	1	0,013969927	0,038365445	3,260598088
ER-associated misfolded protein catabolic process	1	0,013969927	0,038365445	3,260598088
regulation of aerobic respiration	1	0,013969927	0,038365445	3,260598088
positive regulation of oxidative phosphorylation	1	0,013969927	0,038365445	3,260598088

negative regulation of metalloenzyme activity	1	0,013969927	0,038365445	3,260598088
negative regulation of membrane protein ectodomain proteolysis	1	0,013969927	0,038365445	3,260598088
negative regulation of T cell cytokine production	1	0,013969927	0,038365445	3,260598088
positive regulation of receptor binding	1	0,013969927	0,038365445	3,260598088
peptidyl-proline hydroxylation to 4-hydroxy-L-proline	1	0,013969927	0,038365445	3,260598088
positive regulation of mast cell chemotaxis	1	0,013969927	0,038365445	3,260598088
coronary artery morphogenesis	1	0,013969927	0,038365445	3,260598088
coumarin metabolic process	1	0,013969927	0,038365445	3,260598088
monoterpenoid metabolic process	1	0,013969927	0,038365445	3,260598088
positive regulation of corticotropin secretion	1	0,013969927	0,038365445	3,260598088
regulation of branching involved in lung morphogenesis	1	0,013969927	0,038365445	3,260598088
positive regulation of response to reactive oxygen species	1	0,013969927	0,038365445	3,260598088
regulation of adrenergic receptor signaling pathway	1	0,013969927	0,038365445	3,260598088
flavin-containing compound metabolic process	1	0,013969927	0,038365445	3,260598088
regulation of ATP biosynthetic process	1	0,013969927	0,038365445	3,260598088
T cell antigen processing and presentation	1	0,013969927	0,038365445	3,260598088
negative regulation of antigen processing and presentation	1	0,013969927	0,038365445	3,260598088
regulation of histone deacetylase activity	1	0,013969927	0,038365445	3,260598088
protein localization to early endosome	1	0,013969927	0,038365445	3,260598088
regulation of receptor-mediated endocytosis	2	0,013996914	0,038365445	3,260598088
nephron epithelium morphogenesis	2	0,013996914	0,038365445	3,260598088
mitotic cell cycle process	6	0,014057853	0,038494997	3,257227
neurotrophin TRK receptor signaling pathway	4	0,014128304	0,038650317	3,253200292
regulation of neural precursor cell proliferation	2	0,014344205	0,039202852	3,239005791
neurotrophin signaling pathway	4	0,014483925	0,039546312	3,230282833
regulation of cyclase activity	2	0,014695221	0,039968162	3,219672084
regulation of phosphatidylinositol 3-kinase signaling	2	0,014695221	0,039968162	3,219672084
adherens junction assembly	2	0,014695221	0,039968162	3,219672084
nephron morphogenesis	2	0,014695221	0,039968162	3,219672084
epithelial cell development	3	0,014826747	0,040286962	3,211727384
neuron projection morphogenesis	6	0,015048683	0,040814637	3,198714515
negative regulation of lipid metabolic process	2	0,015049944	0,040814637	3,198714515
neuron apoptotic process	3	0,015186163	0,041144417	3,190667032
transcription, DNA-templated	15	0,015320843	0,041469396	3,18279957
regulation of sodium ion transport	2	0,015408356	0,041586309	3,179984266
regulation of lyase activity	2	0,015408356	0,041586309	3,179984266
epithelial cell apoptotic process	2	0,015408356	0,041586309	3,179984266
response to peptide	5	0,015542547	0,041809639	3,174628357
glucose homeostasis	3	0,015550512	0,041809639	3,174628357
regulation of symbiosis, encompassing mutualism through parasitism	3	0,015550512	0,041809639	3,174628357
carbohydrate homeostasis	3	0,015550512	0,041809639	3,174628357
nitrogen compound metabolic process	23	0,015700275	0,042172018	3,165998368
artery development	2	0,015770437	0,042320095	3,162493251
response to temperature stimulus	3	0,016106303	0,042391615	3,160804689
gonad development	3	0,016106303	0,042391615	3,160804689
immune response	9	0,016112814	0,042391615	3,160804689
regulation of JUN kinase activity	2	0,01613617	0,042391615	3,160804689
vasoconstriction	2	0,01613617	0,042391615	3,160804689

transformed cell apoptotic process	1	0,016279686	0,042391615	3,160804689
cellular response to nicotine	1	0,016279686	0,042391615	3,160804689
peripheral nervous system myelin maintenance	1	0,016279686	0,042391615	3,160804689
elastic fiber assembly	1	0,016279686	0,042391615	3,160804689
negative regulation of transforming growth factor beta production	1	0,016279686	0,042391615	3,160804689
peptidyl-serine autophosphorylation	1	0,016279686	0,042391615	3,160804689
protein hexamerization	1	0,016279686	0,042391615	3,160804689
iron ion import into cell	1	0,016279686	0,042391615	3,160804689
positive regulation of B cell receptor signaling pathway	1	0,016279686	0,042391615	3,160804689
positive regulation of viral entry into host cell	1	0,016279686	0,042391615	3,160804689
positive regulation of axon extension involved in axon guidance	1	0,016279686	0,042391615	3,160804689
regulation of metanephric nephron tubule epithelial cell differentiation	1	0,016279686	0,042391615	3,160804689
regulation of myosin-light-chain-phosphatase activity	1	0,016279686	0,042391615	3,160804689
regulation of calcidiol 1-monooxygenase activity	1	0,016279686	0,042391615	3,160804689
regulation of chemokine (C-X-C motif) ligand 2 production	1	0,016279686	0,042391615	3,160804689
regulation of endodeoxyribonuclease activity	1	0,016279686	0,042391615	3,160804689
G-protein coupled receptor signaling pathway involved in heart process	1	0,016279686	0,042391615	3,160804689
regulation of protein deubiquitination	1	0,016279686	0,042391615	3,160804689
positive regulation of axon guidance	1	0,016279686	0,042391615	3,160804689
primitive hemopoiesis	1	0,016279686	0,042391615	3,160804689
regulation of mast cell chemotaxis	1	0,016279686	0,042391615	3,160804689
vascular smooth muscle cell development	1	0,016279686	0,042391615	3,160804689
positive regulation of deacetylase activity	1	0,016279686	0,042391615	3,160804689
post-embryonic eye morphogenesis	1	0,016279686	0,042391615	3,160804689
phenylpropanoid metabolic process	1	0,016279686	0,042391615	3,160804689
regulation of corticotropin secretion	1	0,016279686	0,042391615	3,160804689
metanephric nephron tubule epithelial cell differentiation	1	0,016279686	0,042391615	3,160804689
regulation of heart contraction	3	0,016294042	0,042391615	3,160804689
homeostasis of number of cells	3	0,016483019	0,042843675	3,15019726
adult locomotory behavior	2	0,016878519	0,043831209	3,127409188
regulation of I-kappaB kinase/NF-kappaB signaling	3	0,017057395	0,044173474	3,119630807
synapse organization	3	0,017057395	0,044173474	3,119630807
development of primary sexual characteristics	3	0,017057395	0,044173474	3,119630807
cell adhesion	8	0,017196615	0,04449308	3,112421612
extrinsic apoptotic signaling pathway via death domain receptors	2	0,017255098	0,044552681	3,111082952
cell-substrate junction assembly	2	0,017255098	0,044552681	3,111082952
negative regulation of phosphorylation	4	0,017267131	0,044552681	3,111082952
positive regulation of ion transport	3	0,017446522	0,044974321	3,10166359
platelet degranulation	2	0,017635258	0,045419259	3,091819049
gliogenesis	3	0,017840624	0,045906176	3,081155616
regulation of leukocyte differentiation	3	0,018039543	0,046375628	3,070981222
single-organism behavior	4	0,018080639	0,046438866	3,069618535
positive regulation of peptide hormone secretion	2	0,018406243	0,046584546	3,06648643
positive regulation of reactive oxygen species metabolic process	2	0,018406243	0,046584546	3,06648643
positive regulation of interleukin-8 biosynthetic process	1	0,018584172	0,046584546	3,06648643
positive regulation of cyclin-dependent protein serine/threonine kinase activity involved in G1/S transition of mitotic cell cycle	1	0,018584172	0,046584546	3,06648643
cellular response to granulocyte macrophage colony-stimulating factor stimulus	1	0,018584172	0,046584546	3,06648643
skeletal muscle atrophy	1	0,018584172	0,046584546	3,06648643

tRNA wobble uridine modification	1	0,018584172	0,046584546	3,06648643
positive regulation of lipopolysaccharide-mediated signaling pathway	1	0,018584172	0,046584546	3,06648643
negative regulation of glial cell apoptotic process	1	0,018584172	0,046584546	3,06648643
negative regulation of protein ubiquitination involved in ubiquitin-dependent protein catabolic process	1	0,018584172	0,046584546	3,06648643
synaptic vesicle docking	1	0,018584172	0,046584546	3,06648643
cardiac muscle fiber development	1	0,018584172	0,046584546	3,06648643
commissural neuron axon guidance	1	0,018584172	0,046584546	3,06648643
positive regulation of protein kinase C signaling	1	0,018584172	0,046584546	3,06648643
positive regulation of cell migration involved in sprouting angiogenesis	1	0,018584172	0,046584546	3,06648643
lung vasculature development	1	0,018584172	0,046584546	3,06648643
lung lobe morphogenesis	1	0,018584172	0,046584546	3,06648643
negative regulation of anion transmembrane transport	1	0,018584172	0,046584546	3,06648643
negative regulation of fatty acid transport	1	0,018584172	0,046584546	3,06648643
regulation of deoxyribonuclease activity	1	0,018584172	0,046584546	3,06648643
response to granulocyte macrophage colony-stimulating factor	1	0,018584172	0,046584546	3,06648643
regulation of glial cell apoptotic process	1	0,018584172	0,046584546	3,06648643
ferrous iron transport	1	0,018584172	0,046584546	3,06648643
regulation of antigen processing and presentation of peptide antigen via MHC class I	1	0,018584172	0,046584546	3,06648643
regulation of CD8-positive, alpha-beta T cell activation	1	0,018584172	0,046584546	3,06648643
cardiac vascular smooth muscle cell differentiation	1	0,018584172	0,046584546	3,06648643
regulation of mesenchymal to epithelial transition involved in metanephros morphogenesis	1	0,018584172	0,046584546	3,06648643
cellular chemical homeostasis	5	0,018735558	0,046922239	3,059263528
regulation of axon extension	2	0,018797034	0,047034356	3,056876976
purine ribonucleotide biosynthetic process	3	0,019052836	0,047622824	3,044443144
cellular response to organonitrogen compound	5	0,019066047	0,047622824	3,044443144
activation of cysteine-type endopeptidase activity involved in apoptotic process	2	0,019191333	0,047667001	3,043515926
regulation of oxidoreductase activity	2	0,019191333	0,047667001	3,043515926
regulation of generation of precursor metabolites and energy	2	0,019191333	0,047667001	3,043515926
positive regulation of blood circulation	2	0,019191333	0,047667001	3,043515926
regulation of gliogenesis	2	0,019191333	0,047667001	3,043515926
regulation of cell-matrix adhesion	2	0,019191333	0,047667001	3,043515926
response to drug	4	0,019202266	0,047667001	3,043515926
cellular nitrogen compound metabolic process	22	0,019458488	0,048260479	3,031142294
regulation of cellular response to growth factor stimulus	3	0,019675792	0,048756474	3,020917286
embryonic placenta development	2	0,019990384	0,04936222	3,008569919
positive regulation of cyclic nucleotide biosynthetic process	2	0,019990384	0,04936222	3,008569919
membrane depolarization	2	0,019990384	0,04936222	3,008569919
positive regulation of peptide secretion	2	0,019990384	0,04936222	3,008569919
negative regulation of intracellular signal transduction	4	0,020071452	0,049518963	3,005399599
gene expression	19	0,020089182	0,049519305	3,005392686
regulation of homeostatic process	4	0,020218661	0,049794866	2,999843391
purine nucleotide biosynthetic process	3	0,020310001	0,049976094	2,996210505
