# Supplementary material for: Molecular Taxonomy of Sporadic Amyotrophic Lateral Sclerosis Using Disease-Associated Genes
Source: Front Neurol. 2017 Apr 19;8:152. doi: 10.3389/fneur.2017.00152 (PMC5395696; doi:10.3389/fneur.2017.00152)
Supplement: Supplementary file 5 [file Table_5.PDF]

Supplementary Table 5. List of the significantly enriched GO biological processes for differentially expressed SGALS genes in SALS2 patients

GO biological process	SGALS genes in category	p value	fdr p value	-LOG(corr.pValue)
**response to oxidative stress**	20	6,11E-15	2,35E-11	24,4730177
**apoptotic process**	35	1,11E-12	1,15E-09	20,58471301
cell death	36	1,32E-12	1,15E-09	20,58471301
death	36	1,32E-12	1,15E-09	20,58471301
programmed cell death	35	1,49E-12	1,15E-09	20,58471301
**response to endogenous stimulus**	34	1,97E-12	1,26E-09	20,49016696
negative regulation of apoptotic process	24	3,34E-12	1,83E-09	20,11693995
negative regulation of programmed cell death	24	4,17E-12	2,00E-09	20,02768544
**response to inorganic substance**	19	8,33E-12	2,89E-09	19,66317002
regulation of cell death	31	8,63E-12	2,89E-09	19,66317002
cellular response to oxidative stress	14	8,76E-12	2,89E-09	19,66317002
regulation of apoptotic process	30	9,00E-12	2,89E-09	19,66317002
cellular response to chemical stimulus	42	1,06E-11	2,99E-09	19,62684594
regulation of programmed cell death	30	1,09E-11	2,99E-09	19,62684594
negative regulation of cell death	24	1,93E-11	4,95E-09	19,12460308
response to oxygen-containing compound	31	2,69E-11	6,48E-09	18,85455652
response to toxic substance	14	3,36E-11	7,60E-09	18,69494308
**system development**	51	6,50E-11	1,39E-08	18,09093283
**regulation of localization**	37	7,09E-11	1,44E-08	18,0585313
regulation of apoptotic signaling pathway	16	7,69E-11	1,48E-08	18,02948047
**cellular component organization**	60	1,99E-10	3,65E-08	17,12718171
**regulation of transport**	31	2,08E-10	3,65E-08	17,12718171
response to organic cyclic compound	23	2,41E-10	4,03E-08	17,02704371
response to nitrogen compound	24	3,32E-10	5,25E-08	16,76218388
negative regulation of apoptotic signaling pathway	12	3,52E-10	5,25E-08	16,76218388
response to organonitrogen compound	23	3,55E-10	5,25E-08	16,76218388
regulation of neuron death	13	5,72E-10	8,15E-08	16,32274917
**regulation of biological quality**	44	7,21E-10	9,91E-08	16,12706824
**neurofilament cytoskeleton organization**	5	7,59E-10	1,01E-07	16,1108668
response to organic substance	40	8,84E-10	1,13E-07	15,99195288
positive regulation of cellular process	51	9,78E-10	1,21E-07	15,92401545
response to lipid	22	1,07E-09	1,28E-07	15,8691959
response to steroid hormone	17	1,18E-09	1,35E-07	15,81493604
single-organism transport	46	1,20E-09	1,35E-07	15,81493604
response to chemical	49	1,25E-09	1,37E-07	15,80101826
response to reactive oxygen species	12	1,31E-09	1,40E-07	15,78444574
single-organism localization	47	1,39E-09	1,44E-07	15,75411526
cellular response to organic substance	35	1,42E-09	1,44E-07	15,75411526
response to extracellular stimulus	17	1,60E-09	1,58E-07	15,65893749
regulation of cellular component organization	33	2,00E-09	1,92E-07	15,46338402
homeostatic process	28	2,12E-09	1,99E-07	15,42928388

apoptotic signaling pathway	18	2,41E-09	2,21E-07	15,32707255
neuron death	13	2,60E-09	2,32E-07	15,27454334
single-multicellular organism process	58	2,66E-09	2,32E-07	15,27454334
response to stress	46	3,12E-09	2,67E-07	15,13722912
intrinsic apoptotic signaling pathway	13	3,32E-09	2,78E-07	15,0953309
negative regulation of cellular process	47	4,31E-09	3,53E-07	14,85819961
response to alcohol	14	5,08E-09	4,08E-07	14,71319144
positive regulation of cellular metabolic process	38	5,35E-09	4,21E-07	14,68177639
response to nutrient levels	16	5,60E-09	4,31E-07	14,65643226
multicellular organism development	51	6,32E-09	4,77E-07	14,55633269
response to hormone	23	7,02E-09	5,20E-07	14,47024418
cell-type specific apoptotic process	15	7,76E-09	5,64E-07	14,38870379
transport	48	8,26E-09	5,88E-07	14,34591232
anatomical structure development	52	9,79E-09	6,85E-07	14,19346405
response to glucocorticoid	10	1,08E-08	7,42E-07	14,11445527
regulation of phosphorylation	26	1,22E-08	8,27E-07	14,00565678
negative regulation of neuron death	10	1,61E-08	1,07E-06	13,74871668
positive regulation of macromolecule metabolic process	37	1,76E-08	1,15E-06	13,6783631
regulation of cellular response to oxidative stress	7	1,93E-08	1,24E-06	13,60179831
response to corticosteroid	10	2,01E-08	1,27E-06	13,57814729
regulation of glucose import	7	2,18E-08	1,35E-06	13,51464061
establishment of localization	48	2,30E-08	1,41E-06	13,47389001
cellular response to reactive oxygen species	9	2,65E-08	1,59E-06	13,3509962
phosphorylation	31	2,98E-08	1,76E-06	13,24949758
regulation of response to oxidative stress	7	3,08E-08	1,80E-06	13,23000192
positive regulation of cellular component organization	22	3,18E-08	1,83E-06	13,21385908
cellular response to stress	29	4,28E-08	2,40E-06	12,94027786
protein phosphorylation	28	4,30E-08	2,40E-06	12,94027786
regulation of glucose transport	8	4,89E-08	2,69E-06	12,82718542
positive regulation of signaling	26	5,08E-08	2,75E-06	12,80358109
regulation of cell differentiation	25	6,42E-08	3,43E-06	12,58258706
positive regulation of cell communication	26	6,53E-08	3,44E-06	12,57955462
regulation of intrinsic apoptotic signaling pathway	9	6,65E-08	3,46E-06	12,57488702
regulation of protein phosphorylation	24	7,14E-08	3,66E-06	12,51729365
nervous system development	32	7,82E-08	3,95E-06	12,44274805
cellular response to oxygen-containing compound	21	7,91E-08	3,95E-06	12,44274805
response to acid chemical	12	8,00E-08	3,95E-06	12,44274805
glucose import	7	8,71E-08	4,24E-06	12,37075265
response to amino acid	8	1,09E-07	5,24E-06	12,15868042
regulation of oxidative stress-induced cell death	6	1,18E-07	5,58E-06	12,09575771
neurofilament bundle assembly	3	1,28E-07	6,02E-06	12,02031889
regulation of cellular response to stress	16	1,42E-07	6,52E-06	11,94057381
regulation of multicellular organismal process	33	1,44E-07	6,52E-06	11,94057381

animal organ development	37	1,44E-07	6,52E-06	11,94057381
regulation of signaling	36	1,48E-07	6,56E-06	11,93375853
single-organism developmental process	53	1,48E-07	6,56E-06	11,93375853
cellular response to stimulus	60	1,53E-07	6,68E-06	11,91685114
positive regulation of response to stimulus	29	1,61E-07	6,98E-06	11,87311458
transmembrane receptor protein tyrosine kinase signaling pathway	19	1,71E-07	7,26E-06	11,83364911
regulation of cell communication	36	1,72E-07	7,26E-06	11,83364911
neurogenesis	26	1,78E-07	7,44E-06	11,80888575
enzyme linked receptor protein signaling pathway	22	1,84E-07	7,53E-06	11,79694687
cellular response to superoxide	5	1,86E-07	7,53E-06	11,79694687
cellular response to oxygen radical	5	1,86E-07	7,53E-06	11,79694687
cell differentiation	41	2,20E-07	8,83E-06	11,63708295
aging	11	2,26E-07	8,98E-06	11,62059667
regulation of phosphate metabolic process	26	2,29E-07	9,01E-06	11,61753074
intracellular signal transduction	33	2,52E-07	9,74E-06	11,53951589
cellular response to endogenous stimulus	23	2,53E-07	9,74E-06	11,53951589
regulation of phosphorus metabolic process	26	2,66E-07	1,01E-05	11,49935687
response to hydrogen peroxide	8	2,69E-07	1,02E-05	11,49639846
generation of neurons	25	2,74E-07	1,02E-05	11,48939537
single organism signaling	55	3,00E-07	1,11E-05	11,40941938
response to superoxide	5	3,31E-07	1,21E-05	11,31830259
positive regulation of reactive oxygen species metabolic process	7	3,45E-07	1,25E-05	11,28847375
regulation of cell proliferation	24	3,53E-07	1,27E-05	11,27321552
response to abiotic stimulus	21	3,94E-07	1,39E-05	11,18612973
response to oxygen radical	5	3,96E-07	1,39E-05	11,18612973
regulation of developmental process	29	3,96E-07	1,39E-05	11,18612973
negative regulation of extrinsic apoptotic signaling pathway	7	4,02E-07	1,39E-05	11,1817593
tissue development	26	4,49E-07	1,54E-05	11,07897167
positive regulation of multicellular organismal process	23	4,65E-07	1,58E-05	11,05346279
release of cytochrome c from mitochondria	6	4,84E-07	1,63E-05	11,02281598
reactive oxygen species metabolic process	10	4,88E-07	1,63E-05	11,02264385
cell communication	55	4,97E-07	1,65E-05	11,01293084
negative regulation of metabolic process	33	5,07E-07	1,67E-05	11,00169407
regulation of cellular protein metabolic process	31	5,29E-07	1,73E-05	10,96731598
positive regulation of cell death	15	5,49E-07	1,78E-05	10,93896251
cell development	29	5,70E-07	1,82E-05	10,91510079
phosphate-containing compound metabolic process	35	5,74E-07	1,82E-05	10,91510079
positive regulation of nitrogen compound metabolic process	26	5,76E-07	1,82E-05	10,91510079
positive regulation of cellular response to oxidative stress	4	6,08E-07	1,89E-05	10,87766538
positive regulation of response to oxidative stress	4	6,08E-07	1,89E-05	10,87766538
chemical homeostasis	19	6,14E-07	1,89E-05	10,87638723
cellular response to growth factor stimulus	18	6,67E-07	2,03E-05	10,80383991
positive regulation of signal transduction	23	6,71E-07	2,03E-05	10,80383991

positive regulation of protein metabolic process	24	6,76E-07	2,03E-05	10,80353557
positive regulation of transport	18	7,22E-07	2,14E-05	10,75249403
cell death in response to oxidative stress	6	7,23E-07	2,14E-05	10,75249403
response to oxygen levels	11	7,32E-07	2,15E-05	10,74740029
glucose transport	8	7,43E-07	2,17E-05	10,73958667
regulation of neuron apoptotic process	9	7,79E-07	2,25E-05	10,70036173
positive regulation of nucleobase-containing compound metabolic process	25	7,96E-07	2,29E-05	10,68602199
positive regulation of cell differentiation	17	8,02E-07	2,29E-05	10,68602199
hexose transport	8	8,25E-07	2,33E-05	10,66536727
positive regulation of cellular protein metabolic process	23	8,82E-07	2,48E-05	10,6055454
monosaccharide transport	8	9,14E-07	2,53E-05	10,58466922
response to transition metal nanoparticle	8	9,14E-07	2,53E-05	10,58466922
phosphorus metabolic process	35	9,46E-07	2,60E-05	10,55733058
positive regulation of phosphorylation	19	9,96E-07	2,71E-05	10,51654639
response to growth factor	18	9,99E-07	2,71E-05	10,51654639
regulation of multicellular organismal development	24	1,09E-06	2,93E-05	10,43933373
glial cell apoptotic process	4	1,10E-06	2,93E-05	10,4371493
response to wounding	19	1,22E-06	3,23E-05	10,34154624
regulation of extrinsic apoptotic signaling pathway	8	1,29E-06	3,41E-05	10,28664135
cell surface receptor signaling pathway	33	1,31E-06	3,41E-05	10,2849898
negative regulation of cellular metabolic process	30	1,31E-06	3,41E-05	10,2849898
cellular developmental process	41	1,35E-06	3,48E-05	10,26548996
regulation of reactive oxygen species metabolic process	8	1,36E-06	3,48E-05	10,26548996
positive regulation of apoptotic process	14	1,48E-06	3,75E-05	10,19005606
regulation of mitochondrion organization	9	1,48E-06	3,75E-05	10,19005606
cellular component disassembly	16	1,50E-06	3,78E-05	10,1844518
regulation of intracellular signal transduction	24	1,51E-06	3,78E-05	10,1844518
cellular localization	34	1,59E-06	3,95E-05	10,13936374
regulation of cysteine-type endopeptidase activity	9	1,60E-06	3,95E-05	10,13936374
positive regulation of programmed cell death	14	1,63E-06	4,01E-05	10,12494633
positive regulation of metabolic process	38	1,74E-06	4,24E-05	10,06788654
negative regulation of macromolecule metabolic process	30	1,77E-06	4,28E-05	10,05863841
regulation of protein modification process	25	1,78E-06	4,28E-05	10,05856861
negative regulation of glucose transport	4	1,83E-06	4,38E-05	10,03694933
response to carbohydrate	9	1,93E-06	4,59E-05	9,988876108
negative regulation of signal transduction	19	1,95E-06	4,59E-05	9,988428792
neuron apoptotic process	9	2,01E-06	4,71E-05	9,964076131
negative regulation of signaling	20	2,04E-06	4,75E-05	9,95478616
apoptotic mitochondrial changes	7	2,09E-06	4,84E-05	9,935701125
cell-cell signaling	20	2,20E-06	5,07E-05	9,890543363
response to drug	12	2,34E-06	5,35E-05	9,83503318
positive regulation of biosynthetic process	25	2,37E-06	5,35E-05	9,83503318
negative regulation of cell communication	20	2,37E-06	5,35E-05	9,83503318

regulation of protein metabolic process	31	2,38E-06	5,35E-05	9,83503318
negative regulation of molecular function	19	2,41E-06	5,39E-05	9,827532626
intermediate filament bundle assembly	3	2,54E-06	5,63E-05	9,785160136
cell proliferation	26	2,54E-06	5,63E-05	9,785160136
cellular catabolic process	24	2,73E-06	5,98E-05	9,724339975
regulation of proteolysis	15	2,74E-06	5,98E-05	9,724339975
response to hypoxia	10	2,77E-06	6,02E-05	9,717962323
protein insertion into membrane	5	2,85E-06	6,10E-05	9,704156438
regulation of release of cytochrome c from mitochondria	5	2,85E-06	6,10E-05	9,704156438
intermediate filament cytoskeleton organization	5	2,85E-06	6,10E-05	9,704156438
extrinsic apoptotic signaling pathway	9	2,88E-06	6,12E-05	9,702058859
regulation of signal transduction	31	2,90E-06	6,12E-05	9,700598672
single-organism intracellular transport	24	3,06E-06	6,43E-05	9,651575924
intermediate filament-based process	5	3,20E-06	6,69E-05	9,612354052
response to salt stress	4	3,54E-06	7,36E-05	9,516737079
response to decreased oxygen levels	10	3,61E-06	7,47E-05	9,502619286
negative regulation of phosphorus metabolic process	13	3,88E-06	7,94E-05	9,441122379
negative regulation of phosphate metabolic process	13	3,88E-06	7,94E-05	9,441122379
regulation of cellular localization	20	3,93E-06	8,00E-05	9,433723382
negative regulation of transport	12	4,13E-06	8,36E-05	9,38952192
positive regulation of developmental process	19	4,37E-06	8,81E-05	9,33742603
negative regulation of protein phosphorylation	11	4,44E-06	8,90E-05	9,326728353
neuron differentiation	22	4,47E-06	8,91E-05	9,325533106
carbohydrate transport	8	4,62E-06	9,14E-05	9,300206969
peptidyl-serine phosphorylation	9	4,63E-06	9,14E-05	9,300206969
positive regulation of phosphorus metabolic process	19	4,77E-06	9,32E-05	9,280603814
positive regulation of phosphate metabolic process	19	4,77E-06	9,32E-05	9,280603814
removal of superoxide radicals	4	5,20E-06	0,000100461	9,205743029
regulation of superoxide metabolic process	4	5,20E-06	0,000100461	9,205743029
response to lipopolysaccharide	10	5,81E-06	0,000111831	9,098518544
embryo implantation	5	6,00E-06	0,000114162	9,077893116
positive regulation of cellular biosynthetic process	24	6,02E-06	0,000114162	9,077893116
negative regulation of neuron apoptotic process	7	6,02E-06	0,000114162	9,077893116
positive regulation of macromolecule biosynthetic process	23	6,14E-06	0,000115752	9,064063834
establishment of localization in cell	30	6,28E-06	0,000117745	9,046989526
positive regulation of cellular catabolic process	10	6,30E-06	0,000117745	9,046989526
regulation of cellular catabolic process	13	6,66E-06	0,00012375	8,997248685
peptidyl-serine modification	9	7,24E-06	0,000133479	8,921568261
positive regulation of transcription, DNA-templated	21	7,28E-06	0,000133479	8,921568261
positive regulation of nucleic acid-templated transcription	21	7,28E-06	0,000133479	8,921568261
single organism cell adhesion	15	7,43E-06	0,000135439	8,906991482
catabolic process	27	7,92E-06	0,000143837	8,84682746
response to molecule of bacterial origin	10	8,00E-06	0,000144494	8,842275005

female pregnancy	8	8,20E-06	0,000147467	8,821903529
response to metal ion	10	8,64E-06	0,000154718	8,773903471
superoxide metabolic process	5	8,75E-06	0,000155179	8,770933366
regulation of mitochondrial membrane potential	5	8,75E-06	0,000155179	8,770933366
execution phase of apoptosis	6	8,93E-06	0,00015771	8,754754922
regulation of response to stress	21	9,13E-06	0,00015996	8,740584014
positive regulation of intracellular signal transduction	17	9,15E-06	0,00015996	8,740584014
regulation of cysteine-type endopeptidase activity involved in apoptotic process	8	9,48E-06	0,000165039	8,709327696
neuron projection development	18	9,53E-06	0,000165188	8,708423317
positive regulation of RNA biosynthetic process	21	9,83E-06	0,000169569	8,682252833
epithelial cell proliferation	10	1,03E-05	0,000177476	8,636676849
cellular homeostasis	15	1,04E-05	0,000177698	8,635423626
positive regulation of protein phosphorylation	17	1,07E-05	0,00018178	8,612715101
negative regulation of phosphorylation	11	1,07E-05	0,00018178	8,612715101
cellular response to organic cyclic compound	12	1,11E-05	0,000187961	8,579275611
response to external stimulus	29	1,14E-05	0,000191193	8,562226896
signal transduction	49	1,17E-05	0,000195412	8,540402487
response to copper ion	4	1,18E-05	0,000195412	8,540402487
protein insertion into mitochondrial membrane involved in apoptotic signaling pathway	4	1,18E-05	0,000195412	8,540402487
neuron development	19	1,25E-05	0,000207079	8,482409083
inorganic ion homeostasis	13	1,32E-05	0,000216784	8,436610898
regulation of intracellular transport	13	1,34E-05	0,00021962	8,423612454
regulation of oxidative stress-induced intrinsic apoptotic signaling pathway	4	1,36E-05	0,000221034	8,417193739
protein insertion into mitochondrial membrane	4	1,36E-05	0,000221034	8,417193739
negative regulation of cellular component organization	13	1,39E-05	0,000224446	8,401877303
intracellular transport	24	1,40E-05	0,000224701	8,400738556
positive regulation of RNA metabolic process	21	1,49E-05	0,00023834	8,341812415
cell projection organization	21	1,50E-05	0,000239772	8,335824068
single-organism cellular localization	19	1,52E-05	0,000241544	8,328457765
regulation of proteasomal ubiquitin-dependent protein catabolic process	7	1,61E-05	0,000253732	8,27923061
negative regulation of response to stimulus	20	1,61E-05	0,000253732	8,27923061
single organismal cell-cell adhesion	14	1,62E-05	0,000253846	8,27878178
central nervous system development	16	1,63E-05	0,000253846	8,27878178
cellular response to hormone stimulus	15	1,63E-05	0,000253846	8,27878178
positive regulation of gene expression	23	1,64E-05	0,000253846	8,27878178
cellular component assembly	28	1,64E-05	0,000253846	8,27878178
negative regulation of cellular protein metabolic process	17	1,69E-05	0,000260226	8,253959466
negative regulation of insulin receptor signaling pathway	4	1,79E-05	0,000274349	8,201110958
regulation of cell development	15	1,83E-05	0,000279224	8,183497001
ion transport	21	1,84E-05	0,000279224	8,183497001
response to peptide	13	1,85E-05	0,000280224	8,179919898
negative regulation of protein modification process	13	1,98E-05	0,000298207	8,117723419
response to axon injury	5	2,00E-05	0,000300083	8,111453043

regulation of endopeptidase activity	10	2,02E-05	0,000303054	8,101599836
positive regulation of mitochondrion organization	7	2,05E-05	0,000305133	8,094763855
positive regulation of oxidative stress-induced cell death	3	2,06E-05	0,000305347	8,094062202
homeostasis of number of cells	8	2,26E-05	0,000333474	8,005946949
metal ion homeostasis	12	2,26E-05	0,000333474	8,005946949
positive regulation of mitochondrial outer membrane permeabilization involved in apoptotic signaling pathway	4	2,31E-05	0,000336592	7,996637768
negative regulation of oxidative stress-induced cell death	4	2,31E-05	0,000336592	7,996637768
negative regulation of cellular response to insulin stimulus	4	2,31E-05	0,000336592	7,996637768
mitochondrion organization	14	2,43E-05	0,000353108	7,948736143
regulation of transcription from RNA polymerase II promoter	23	2,47E-05	0,000356898	7,938059526
response to osmotic stress	5	2,49E-05	0,000358781	7,932798982
histone H3-T6 phosphorylation	2	2,57E-05	0,000369603	7,903081571
regulation of response to stimulus	36	2,63E-05	0,000376065	7,885749167
secretion	17	2,68E-05	0,000381691	7,870898545
positive regulation of catabolic process	10	2,71E-05	0,000384905	7,862514445
extracellular matrix organization	10	2,89E-05	0,000409549	7,800454548
negative regulation of cellular response to oxidative stress	4	2,93E-05	0,000411762	7,795064703
negative regulation of response to oxidative stress	4	2,93E-05	0,000411762	7,795064703
extracellular structure organization	10	2,96E-05	0,000414	7,789644349
cellular chemical homeostasis	13	3,05E-05	0,000424814	7,763858204
regulation of neuron projection development	10	3,09E-05	0,000429223	7,753532929
regulation of protein kinase activity	15	3,16E-05	0,000437466	7,734511441
regulation of macromolecule metabolic process	48	3,17E-05	0,000437466	7,734511441
cellular response to organonitrogen compound	13	3,19E-05	0,000438776	7,731521192
regulation of peptidase activity	10	3,23E-05	0,000441745	7,724777487
regulation of molecular function	30	3,35E-05	0,000457187	7,690419059
positive regulation of protein modification process	18	3,38E-05	0,000459057	7,686335633
ion homeostasis	13	3,50E-05	0,000474592	7,653055722
leukocyte cell-cell adhesion	11	3,68E-05	0,000497144	7,606631289
multi-multicellular organism process	8	3,76E-05	0,000505671	7,5896252
negative regulation of protein metabolic process	17	3,79E-05	0,000508467	7,584110743
positive regulation of protein localization to nucleus	6	3,90E-05	0,000520962	7,559833627
regulation of catabolic process	13	4,14E-05	0,000551224	7,503370154
single-organism cellular process	78	4,23E-05	0,000560652	7,486410551
cell-cell adhesion	15	4,27E-05	0,000564081	7,480311896
positive regulation of establishment of protein localization	11	4,51E-05	0,000594036	7,428570076
regulation of ion transport	12	4,55E-05	0,000597765	7,422313072
positive regulation of apoptotic signaling pathway	7	4,58E-05	0,000599866	7,418804742
regulation of cellular metabolic process	48	4,74E-05	0,000618449	7,388295715
transcription from RNA polymerase II promoter	24	4,80E-05	0,000624011	7,379342111
organelle organization	35	4,97E-05	0,000644173	7,347543687
regulation of proteasomal protein catabolic process	7	5,07E-05	0,000655071	7,330766319
response to peptide hormone	12	5,10E-05	0,000656041	7,329287763

cellular response to hydrogen peroxide	5	5,15E-05	0,000660704	7,322204257
cation homeostasis	12	5,18E-05	0,000662224	7,319906353
positive regulation of secretion by cell	9	5,20E-05	0,000662799	7,319039165
positive regulation of proteasomal ubiquitin-dependent protein catabolic process	5	5,47E-05	0,000692707	7,274902827
cellular oxidant detoxification	5	5,47E-05	0,000692707	7,274902827
negative regulation of mitochondrion organization	4	5,54E-05	0,000695419	7,270995611
regulation of mitochondrial outer membrane permeabilization involved in apoptotic signaling pathway	4	5,54E-05	0,000695419	7,270995611
cation transport	16	5,57E-05	0,000695419	7,270995611
negative regulation of glucose import	3	5,58E-05	0,000695419	7,270995611
response to mercury ion	3	5,58E-05	0,000695419	7,270995611
divalent metal ion transport	10	5,74E-05	0,000712949	7,246101126
regulation of kinase activity	15	5,91E-05	0,000731391	7,220561682
synaptic transmission	13	5,98E-05	0,000732502	7,219044699
synaptic signaling	13	5,98E-05	0,000732502	7,219044699
trans-synaptic signaling	13	5,98E-05	0,000732502	7,219044699
divalent inorganic cation transport	10	6,09E-05	0,000744305	7,203059859
homotypic cell-cell adhesion	11	6,42E-05	0,000782101	7,153526308
cellular detoxification	5	6,53E-05	0,000790707	7,142582833
regulation of cell projection organization	11	6,53E-05	0,000790707	7,142582833
regulation of autophagy	7	6,59E-05	0,000795288	7,136806209
cellular response to lipid	11	6,65E-05	0,000799406	7,13164134
cellular component biogenesis	28	6,83E-05	0,000813268	7,11444965
regulation of transferase activity	16	6,84E-05	0,000813268	7,11444965
response to ethanol	6	6,84E-05	0,000813268	7,11444965
response to salt	3	6,85E-05	0,000813268	7,11444965
regulation of establishment of protein localization	14	7,14E-05	0,000845421	7,07567553
cellular response to nitrogen compound	13	7,19E-05	0,000848417	7,072138227
positive regulation of molecular function	22	7,53E-05	0,000886687	7,028018656
regulation of primary metabolic process	47	7,69E-05	0,000888728	7,025719578
positive regulation of superoxide dismutase activity	2	7,70E-05	0,000888728	7,025719578
response to sodium arsenite	2	7,70E-05	0,000888728	7,025719578
response to acrylamide	2	7,70E-05	0,000888728	7,025719578
positive regulation of removal of superoxide radicals	2	7,70E-05	0,000888728	7,025719578
regulation of ubiquitin-specific protease activity	2	7,70E-05	0,000888728	7,025719578
positive regulation of cell proliferation	14	7,71E-05	0,000888728	7,025719578
negative regulation of biosynthetic process	20	7,75E-05	0,000889641	7,02469301
hemopoiesis	13	7,81E-05	0,000894992	7,018695992
regulation of membrane potential	9	7,95E-05	0,000907563	7,004747045
intrinsic apoptotic signaling pathway in response to oxidative stress	4	8,03E-05	0,000910972	7,000998773
regulation of insulin receptor signaling pathway	4	8,03E-05	0,000910972	7,000998773
organonitrogen compound catabolic process	9	8,12E-05	0,000919153	6,99205776
signal release	10	8,28E-05	0,000932265	6,977893899
secretion by cell	15	8,29E-05	0,000932265	6,977893899

regulation of organelle organization	16	8,55E-05	0,000959454	6,949146611
positive regulation of proteolysis	9	8,66E-05	0,00096872	6,939534598
cellular ion homeostasis	11	8,85E-05	0,000987357	6,920479156
lymphocyte activation	12	8,90E-05	0,000989663	6,918146111
cellular response to external stimulus	8	9,07E-05	0,001001683	6,90607332
T cell activation	10	9,08E-05	0,001001683	6,90607332
T cell aggregation	10	9,08E-05	0,001001683	6,90607332
positive regulation of intracellular transport	9	9,23E-05	0,001014515	6,893344246
lymphocyte aggregation	10	9,25E-05	0,001014515	6,893344246
response to alkaloid	6	9,39E-05	0,00102649	6,881609715
regulation of binding	8	9,53E-05	0,00103626	6,872137095
positive regulation of oxidoreductase activity	4	9,53E-05	0,00103626	6,872137095
regulation of neuron differentiation	11	9,93E-05	0,001076082	6,834428731
response to insulin	10	9,96E-05	0,001076305	6,834221751
cell adhesion	19	0,000100112	0,001079079	6,831647642
regulation of protein localization	15	0,000101047	0,001079977	6,830815858
negative regulation of intrinsic apoptotic signaling pathway	5	0,000101318	0,001079977	6,830815858
regulation of oxidoreductase activity	5	0,000101318	0,001079977	6,830815858
regulation of generation of precursor metabolites and energy	5	0,000101318	0,001079977	6,830815858
regulation of epithelial cell proliferation	8	0,000102552	0,001090111	6,821476038
positive regulation of secretion	9	0,000104599	0,001108482	6,804763627
gland development	10	0,000105144	0,001108482	6,804763627
leukocyte aggregation	10	0,000105144	0,001108482	6,804763627
MAPK cascade	14	0,000105609	0,001110334	6,803094574
positive regulation of proteasomal protein catabolic process	5	0,000112329	0,00117497	6,746512838
mitochondrial outer membrane permeabilization	4	0,000112367	0,00117497	6,746512838
cell activation	15	0,000117232	0,001222515	6,706845414
mitochondrial membrane organization	5	0,000118167	0,00122894	6,701603471
regulation of neurogenesis	12	0,000119195	0,001236282	6,695647035
leukocyte differentiation	10	0,000121306	0,001248449	6,685853062
lipopolysaccharide-mediated signaling pathway	4	0,000121665	0,001248449	6,685853062
positive regulation of mitochondrial membrane permeability involved in apoptotic process	4	0,000121665	0,001248449	6,685853062
mitochondrial outer membrane permeabilization involved in programmed cell death	4	0,000121665	0,001248449	6,685853062
proteolysis	20	0,000123406	0,001259676	6,676900725
single-organism catabolic process	16	0,000123414	0,001259676	6,676900725
calcium ion homeostasis	9	0,00012564	0,001278998	6,661678107
calcium ion transport	9	0,000128182	0,001301439	6,644284825
organophosphate catabolic process	6	0,000131076	0,001327316	6,624596223
intermediate filament organization	3	0,000137357	0,001387272	6,580415906
positive regulation of mitochondrial membrane permeability	4	0,000141917	0,001429568	6,550382982
single organism reproductive process	17	0,000143281	0,001439542	6,543430477
activation of cysteine-type endopeptidase activity	5	0,000143883	0,001441826	6,541844697
macromolecular complex assembly	20	0,000145019	0,001449438	6,536579228

hematopoietic or lymphoid organ development	13	0,000146049	0,001455947	6,532098754
positive regulation of sequence-specific DNA binding transcription factor activity	7	0,000146981	0,001457687	6,530904406
positive regulation of neuron projection development	7	0,000146981	0,001457687	6,530904406
regulation of peptide hormone secretion	7	0,000151081	0,001479647	6,515951813
signal transduction by protein phosphorylation	14	0,000151525	0,001479647	6,515951813
positive chemotaxis	4	0,000152908	0,001479647	6,515951813
positive regulation of apoptotic process involved in mammary gland involution	2	0,000153425	0,001479647	6,515951813
post-embryonic camera-type eye morphogenesis	2	0,000153425	0,001479647	6,515951813
regulation of superoxide dismutase activity	2	0,000153425	0,001479647	6,515951813
apoptotic process involved in mammary gland involution	2	0,000153425	0,001479647	6,515951813
positive regulation of apoptotic process involved in morphogenesis	2	0,000153425	0,001479647	6,515951813
regulation of mammary gland involution	2	0,000153425	0,001479647	6,515951813
positive regulation of mammary gland involution	2	0,000153425	0,001479647	6,515951813
positive regulation of apoptotic process involved in development	2	0,000153425	0,001479647	6,515951813
positive regulation of epithelial cell proliferation	6	0,000156278	0,001503397	6,500027887
positive regulation of organelle organization	11	0,000158973	0,001524498	6,48608982
negative regulation of catabolic process	7	0,00015956	0,001524498	6,48608982
negative regulation of oxidative stress-induced intrinsic apoptotic signaling pathway	3	0,00015966	0,001524498	6,48608982
macromolecule localization	28	0,000161021	0,001533685	6,480082076
positive regulation of cell migration	9	0,000162216	0,001541251	6,475161176
establishment of localization by movement along microtubule	5	0,000165825	0,001567799	6,458082469
response to vitamin	5	0,000165825	0,001567799	6,458082469
single-organism membrane organization	14	0,000168313	0,001584557	6,447450275
glucose metabolic process	7	0,000168421	0,001584557	6,447450275
regulation of peptide secretion	7	0,000172999	0,001623655	6,423075349
regulation of mitochondrial membrane permeability involved in apoptotic process	4	0,000176717	0,001654517	6,404245943
regulation of protein catabolic process	9	0,000178472	0,00166689	6,396795883
regulation of biosynthetic process	37	0,00017912	0,001668893	6,395594644
cellular metal ion homeostasis	10	0,000179965	0,001672719	6,393305013
positive regulation of cardiac muscle hypertrophy	3	0,00018419	0,001703753	6,374921697
positive regulation of muscle hypertrophy	3	0,00018419	0,001703753	6,374921697
pallium development	6	0,000185174	0,001708752	6,371992102
positive regulation of intrinsic apoptotic signaling pathway	4	0,000189574	0,001745172	6,350902095
skeletal muscle tissue development	6	0,000191429	0,00175804	6,343555638
positive regulation of transcription from RNA polymerase II promoter	15	0,000192099	0,001758509	6,343288953
nitrogen compound transport	13	0,000192394	0,001758509	6,343288953
regulation of secretion	12	0,000193492	0,001764353	6,339971489
regulation of cellular process	68	0,000196334	0,001786032	6,327759094
negative regulation of protein kinase activity	7	0,000197424	0,001787503	6,326935674
positive regulation of ion transport	7	0,000197424	0,001787503	6,326935674
nucleoside phosphate catabolic process	5	0,000198955	0,001797131	6,321563609
positive regulation of cell motility	9	0,000203498	0,001833862	6,30133129
metal ion transport	13	0,000204539	0,001838937	6,298567467

cellular response to insulin stimulus	9	0,000207301	0,001859424	6,287488465
regulation of fat cell differentiation	5	0,000207974	0,001861123	6,28657502
response to hyperoxia	3	0,000211039	0,001876569	6,278310356
response to increased oxygen levels	3	0,000211039	0,001876569	6,278310356
divalent inorganic cation homeostasis	9	0,000211163	0,001876569	6,278310356
blood vessel development	11	0,000223375	0,001980527	6,224392314
regulation of anatomical structure morphogenesis	14	0,000228733	0,00202337	6,202991078
cellular response to peptide hormone stimulus	10	0,000229881	0,002028861	6,200280898
telencephalon development	7	0,000236235	0,002080164	6,175308658
ionotropic glutamate receptor signaling pathway	3	0,0002403	0,002111128	6,16053305
protein processing	8	0,000241552	0,002117293	6,157616826
cellular component morphogenesis	19	0,000245939	0,002147763	6,143328634
movement of cell or subcellular component	22	0,000246504	0,002147763	6,143328634
positive regulation of smooth muscle cell proliferation	4	0,000247819	0,002147763	6,143328634
regulation of monooxygenase activity	4	0,000247819	0,002147763	6,143328634
regulation of cellular response to insulin stimulus	4	0,000247819	0,002147763	6,143328634
positive regulation of cellular component movement	9	0,0002487	0,00215056	6,142026953
organic substance catabolic process	21	0,000249848	0,00215564	6,139667592
negative regulation of cysteine-type endopeptidase activity involved in apoptotic signaling pathway	2	0,000254859	0,002193956	6,122049144
immune system development	13	0,000256911	0,00220668	6,116266
regulation of immune system process	19	0,000258438	0,002214855	6,11256851
response to ketone	6	0,000263626	0,002254296	6,094917364
positive regulation of positive chemotaxis	3	0,000272064	0,002321289	6,065632491
positive regulation of locomotion	9	0,000276686	0,002355505	6,050999946
cerebral cortex development	5	0,000280139	0,002376111	6,042290203
skeletal muscle organ development	6	0,000280342	0,002376111	6,042290203
protein localization	25	0,000284321	0,00240391	6,030658534
hormone transport	8	0,000284871	0,00240391	6,030658534
regulation of proteolysis involved in cellular protein catabolic process	7	0,000287898	0,002424137	6,022279742
regulation of cellular component biogenesis	12	0,000293131	0,00246281	6,00645223
cellular response to peptide	10	0,000295487	0,002477199	6,000626629
negative regulation of kinase activity	7	0,000302138	0,002527452	5,980543593
positive regulation of protein complex disassembly	3	0,00030642	0,002529344	5,979795399
anterograde axonal transport	3	0,00030642	0,002529344	5,979795399
positive regulation of protein insertion into mitochondrial membrane involved in apoptotic signaling pathway	3	0,00030642	0,002529344	5,979795399
regulation of positive chemotaxis	3	0,00030642	0,002529344	5,979795399
regulation of protein insertion into mitochondrial membrane involved in apoptotic signaling pathway	3	0,00030642	0,002529344	5,979795399
cellular response to biotic stimulus	6	0,000306966	0,002529344	5,979795399
negative regulation of cellular catabolic process	6	0,000306966	0,002529344	5,979795399
oxidation-reduction process	15	0,000308674	0,002532825	5,978419973
vasculature development	11	0,000308705	0,002532825	5,978419973
regeneration	6	0,000316268	0,00258936	5,956344425
regulation of mitochondrial membrane permeability	4	0,000317938	0,002592003	5,955324483

regulation of muscle adaptation	4	0,000317938	0,002592003	5,955324483
regulation of transmembrane transport	9	0,000329124	0,002677525	5,922862295
cellular cation homeostasis	10	0,000333773	0,002709614	5,910949231
negative regulation of fibroblast proliferation	3	0,000343455	0,002782345	5,884461089
myeloid cell differentiation	8	0,00034777	0,002805492	5,876176482
multicellular organismal homeostasis	8	0,00034777	0,002805492	5,876176482
positive regulation of protein catabolic process	7	0,000356507	0,002868752	5,853878235
negative regulation of macromolecule biosynthetic process	18	0,000357103	0,002868752	5,853878235
regulation of metabolic process	50	0,000360773	0,002892197	5,845738701
cardiovascular system development	14	0,000363648	0,00290315	5,841958899
circulatory system development	14	0,000363648	0,00290315	5,841958899
regulation of secretion by cell	11	0,000368108	0,002932674	5,831840682
regulation of peptidyl-serine phosphorylation	5	0,000369902	0,002934811	5,83111239
cytoskeleton-dependent intracellular transport	5	0,000369902	0,002934811	5,83111239
leukocyte activation	12	0,000370843	0,002936223	5,830631252
cell morphogenesis	18	0,000375281	0,002960241	5,822484579
negative regulation of catalytic activity	13	0,000375415	0,002960241	5,822484579
cellular component disassembly involved in execution phase of apoptosis	4	0,000379113	0,002961949	5,821907739
positive regulation of receptor binding	2	0,00038102	0,002961949	5,821907739
negative regulation of hydrogen peroxide-mediated programmed cell death	2	0,00038102	0,002961949	5,821907739
mitochondrial DNA repair	2	0,00038102	0,002961949	5,821907739
positive regulation of oxidative phosphorylation	2	0,00038102	0,002961949	5,821907739
positive regulation of response to reactive oxygen species	2	0,00038102	0,002961949	5,821907739
flavin-containing compound metabolic process	2	0,00038102	0,002961949	5,821907739
positive regulation of release of cytochrome c from mitochondria	3	0,000383256	0,002967339	5,820089731
apoptotic nuclear changes	3	0,000383256	0,002967339	5,820089731
regulation of nervous system development	12	0,000384586	0,002971658	5,818635225
positive regulation of cellular protein localization	8	0,000390664	0,003006553	5,806961189
protein maturation	8	0,000390664	0,003006553	5,806961189
stress-activated MAPK cascade	7	0,000399946	0,003065721	5,787472508
hexose metabolic process	7	0,000399946	0,003065721	5,787472508
positive regulation of endothelial cell proliferation	4	0,000401252	0,003069616	5,786202888
regulation of growth	11	0,000409251	0,0031246	5,76844894
posttranscriptional regulation of gene expression	9	0,00041615	0,003170984	5,753713439
peptidyl-amino acid modification	17	0,000421472	0,00320288	5,743704998
myeloid leukocyte differentiation	6	0,000422001	0,00320288	5,743704998
regulation of membrane permeability	4	0,000424299	0,003207218	5,742351347
response to arsenic-containing substance	3	0,000425907	0,003207218	5,742351347
positive regulation of monooxygenase activity	3	0,000425907	0,003207218	5,742351347
regulation of cysteine-type endopeptidase activity involved in apoptotic signaling pathway	3	0,000425907	0,003207218	5,742351347
zymogen activation	5	0,000430104	0,003230963	5,734974992
anatomical structure morphogenesis	27	0,000430739	0,003230963	5,734974992
regulation of cellular component movement	12	0,000443967	0,00332371	5,706673669

myeloid cell homeostasis	5	0,000446246	0,003330916	5,704507926
membrane organization	15	0,000447027	0,003330916	5,704507926
peptide hormone secretion	7	0,000447527	0,003330916	5,704507926
regulation of cellular protein localization	10	0,00046106	0,003425015	5,676649349
regulation of cellular protein catabolic process	7	0,000467782	0,003468257	5,664103088
positive regulation of neuron death	4	0,000473205	0,003501716	5,654502079
response to nutrient	6	0,000484299	0,003576931	5,633250184
negative regulation of proteolysis	8	0,000489423	0,003607856	5,624641557
regulation of insulin secretion	6	0,000497576	0,003647441	5,613729351
response to monosaccharide	6	0,000497576	0,003647441	5,613729351
protein complex assembly	17	0,000499101	0,003647441	5,613729351
protein complex biogenesis	17	0,000499101	0,003647441	5,613729351
regulation of hormone secretion	7	0,000499533	0,003647441	5,613729351
ion transmembrane transport	14	0,000504095	0,003673782	5,606533517
vesicle-mediated transport	17	0,000507506	0,003691653	5,601680918
negative regulation of cell proliferation	11	0,000509667	0,003699373	5,59959205
leukocyte proliferation	7	0,00051049	0,003699373	5,59959205
regulation of cell migration	11	0,000522736	0,00376284	5,582581231
response to bacterium	10	0,000531049	0,00376284	5,582581231
transformed cell apoptotic process	2	0,000531658	0,00376284	5,582581231
retrograde axonal transport	2	0,000531658	0,00376284	5,582581231
peripheral nervous system myelin maintenance	2	0,000531658	0,00376284	5,582581231
positive regulation of oxidative stress-induced intrinsic apoptotic signaling pathway	2	0,000531658	0,00376284	5,582581231
protein hexamerization	2	0,000531658	0,00376284	5,582581231
response to L-ascorbic acid	2	0,000531658	0,00376284	5,582581231
regulation of calcidiol 1-monooxygenase activity	2	0,000531658	0,00376284	5,582581231
histone-threonine phosphorylation	2	0,000531658	0,00376284	5,582581231
regulation of protein deubiquitination	2	0,000531658	0,00376284	5,582581231
post-embryonic eye morphogenesis	2	0,000531658	0,00376284	5,582581231
regulation of peptide transport	7	0,000532981	0,00376284	5,582581231
peptide secretion	7	0,000532981	0,00376284	5,582581231
negative regulation of binding	5	0,000533917	0,00376284	5,582581231
cellular calcium ion homeostasis	8	0,000536149	0,003771663	5,580239188
regulation of endocytosis	6	0,000539118	0,003785631	5,576542808
response to UV	5	0,000552901	0,00387283	5,553769723
positive regulation of protein complex assembly	6	0,000553549	0,00387283	5,553769723
regulation of sequence-specific DNA binding transcription factor activity	8	0,000565879	0,003951912	5,53355581
positive regulation of cysteine-type endopeptidase activity	5	0,000572388	0,003985208	5,525165794
protein oligomerization	9	0,000572718	0,003985208	5,525165794
negative regulation of transferase activity	8	0,000576081	0,004001375	5,52111731
extracellular matrix disassembly	5	0,000592387	0,004107218	5,495009355
phosphatidylinositol-mediated signaling	6	0,000614306	0,004243896	5,462273554
cellular response to extracellular stimulus	6	0,000614306	0,004243896	5,462273554

ossification	8	0,000640473	0,004416283	5,422456782
regulation of MAPK cascade	12	0,000641555	0,004416283	5,422456782
coagulation	10	0,000643698	0,004417204	5,42224846
regulation of immune response	14	0,000643984	0,004417204	5,42224846
regulation of mRNA stability	5	0,000655541	0,004486521	5,406677762
positive regulation of cell development	9	0,000657477	0,004486521	5,406677762
stress-activated protein kinase signaling cascade	7	0,000657588	0,004486521	5,406677762
inositol lipid-mediated signaling	6	0,000663179	0,004516663	5,399981891
positive regulation of protein secretion	6	0,000680124	0,004623879	5,376521337
regulation of response to reactive oxygen species	3	0,000684776	0,004647297	5,371469547
cellular response to DNA damage stimulus	12	0,000693458	0,004697931	5,360633035
energy derivation by oxidation of organic compounds	8	0,000698477	0,004711809	5,357683275
regulation of macromolecule biosynthetic process	34	0,000701597	0,004711809	5,357683275
stress-induced premature senescence	2	0,000706527	0,004711809	5,357683275
positive regulation of superoxide anion generation	2	0,000706527	0,004711809	5,357683275
negative regulation of protein ubiquitination involved in ubiquitin-dependent protein catabolic process	2	0,000706527	0,004711809	5,357683275
activation of cysteine-type endopeptidase activity involved in apoptotic process by cytochrome c	2	0,000706527	0,004711809	5,357683275
negative regulation of receptor internalization	2	0,000706527	0,004711809	5,357683275
regulation of hydrogen peroxide-mediated programmed cell death	2	0,000706527	0,004711809	5,357683275
post-embryonic camera-type eye development	2	0,000706527	0,004711809	5,357683275
extrinsic apoptotic signaling pathway in absence of ligand	4	0,000709459	0,004715022	5,357001625
signal transduction in absence of ligand	4	0,000709459	0,004715022	5,357001625
cellular response to abiotic stimulus	7	0,000728024	0,004830063	5,332895738
negative regulation of ion transmembrane transport	4	0,000743919	0,00492702	5,313020902
regulation of RNA stability	5	0,000747437	0,004941817	5,310022119
regulation of protein localization to nucleus	6	0,000751285	0,004955716	5,307213543
establishment of protein localization	21	0,000752115	0,004955716	5,307213543
cellular divalent inorganic cation homeostasis	8	0,000760655	0,00500342	5,29763352
anatomical structure formation involved in morphogenesis	15	0,000773675	0,005080379	5,282369348
response to fatty acid	4	0,000779546	0,005101518	5,27821716
muscle adaptation	4	0,000779546	0,005101518	5,27821716
vascular endothelial growth factor receptor signaling pathway	7	0,00080432	0,005254709	5,248630596
negative regulation of developmental process	12	0,000807922	0,005263081	5,247038643
morphogenesis of a branching structure	6	0,000808337	0,005263081	5,247038643
macrophage differentiation	3	0,000811076	0,005271997	5,245346087
positive regulation of cell projection organization	7	0,000820312	0,005323038	5,235710996
regulation of cell-cell adhesion	8	0,000827226	0,005355384	5,22965296
regulation of peptidyl-tyrosine phosphorylation	6	0,00082808	0,005355384	5,22965296
cell morphogenesis involved in differentiation	14	0,000838742	0,005412349	5,219072114
regulation of cell motility	11	0,000839702	0,005412349	5,219072114
positive regulation of endopeptidase activity	5	0,00087547	0,005630527	5,179552218
positive regulation of glucose import	3	0,000879404	0,005630527	5,179552218
hyaluronan metabolic process	3	0,000879404	0,005630527	5,179552218

regulation of cardiac muscle hypertrophy	3	0,000879404	0,005630527	5,179552218
organic substance transport	25	0,000895428	0,005714106	5,164817435
macromolecular complex subunit organization	25	0,000895428	0,005714106	5,164817435
death-inducing signaling complex assembly	2	0,00090538	0,005749011	5,158727369
regulation of vitamin D biosynthetic process	2	0,00090538	0,005749011	5,158727369
gas homeostasis	2	0,00090538	0,005749011	5,158727369
regulation of vesicle-mediated transport	8	0,000913227	0,005789285	5,151746452
positive regulation of neuron differentiation	7	0,00092163	0,00583295	5,144232419
response to estradiol	5	0,00093101	0,005872466	5,137480585
response to ionizing radiation	5	0,00093101	0,005872466	5,137480585
response to estrogen	6	0,000932452	0,005872466	5,137480585
extrinsic apoptotic signaling pathway via death domain receptors	4	0,000975962	0,006116451	5,09677323
synaptic vesicle cycle	4	0,000975962	0,006116451	5,09677323
regulation of fibroblast proliferation	4	0,000975962	0,006116451	5,09677323
positive regulation of immune system process	13	0,001006915	0,00630018	5,067177048
multi-organism reproductive process	13	0,00101666	0,006335753	5,061546611
response to radiation	9	0,001017415	0,006335753	5,061546611
protein kinase B signaling	5	0,001019187	0,006335753	5,061546611
receptor metabolic process	5	0,001019187	0,006335753	5,061546611
positive regulation of DNA binding	3	0,001026774	0,006362361	5,057355688
regulation of muscle hypertrophy	3	0,001026774	0,006362361	5,057355688
cellular response to oxygen levels	5	0,001049914	0,006495289	5,03667811
monosaccharide metabolic process	7	0,001052065	0,006498149	5,036237987
B cell proliferation	4	0,001063467	0,006547555	5,028663583
negative regulation of transmembrane transport	4	0,001063467	0,006547555	5,028663583
positive regulation of cellular component biogenesis	8	0,001072433	0,006589804	5,022231634
cell morphogenesis involved in neuron differentiation	12	0,001073754	0,006589804	5,022231634
regulation of protein serine/threonine kinase activity	10	0,001079418	0,006614014	5,018564477
muscle cell proliferation	5	0,001081325	0,006615163	5,018390906
organic cyclic compound biosynthetic process	36	0,001083056	0,006615236	5,018379739
regulation of protein secretion	8	0,001089505	0,006644079	5,014029148
insulin secretion	6	0,001095284	0,006668755	5,010322165
protein metabolic process	43	0,00110095	0,006669204	5,010254828
gene expression	41	0,001105068	0,006669204	5,010254828
cellular response to vascular endothelial growth factor stimulus	3	0,001105956	0,006669204	5,010254828
negative regulation of fat cell differentiation	3	0,001105956	0,006669204	5,010254828
axo-dendritic transport	3	0,001105956	0,006669204	5,010254828
negative regulation of cysteine-type endopeptidase activity involved in apoptotic process	4	0,001109223	0,006669204	5,010254828
positive regulation of peptide hormone secretion	4	0,001109223	0,006669204	5,010254828
fibroblast proliferation	4	0,001109223	0,006669204	5,010254828
negative regulation of cell differentiation	10	0,00112004	0,006698216	5,005914084
positive regulation of NFAT protein import into nucleus	2	0,001127974	0,006698216	5,005914084
regulation of branching involved in salivary gland morphogenesis	2	0,001127974	0,006698216	5,005914084

hydrogen peroxide-mediated programmed cell death	2	0,001127974	0,006698216	5,005914084
programmed cell death in response to reactive oxygen species	2	0,001127974	0,006698216	5,005914084
mammary gland involution	2	0,001127974	0,006698216	5,005914084
regulation of apoptotic process involved in morphogenesis	2	0,001127974	0,006698216	5,005914084
regulation of apoptotic process involved in development	2	0,001127974	0,006698216	5,005914084
positive regulation of protein kinase activity	10	0,001133856	0,006722771	5,002254795
cellular response to lipopolysaccharide	5	0,001146233	0,006785697	4,992938213
reactive oxygen species biosynthetic process	4	0,001156343	0,006835038	4,985693252
DNA-templated transcription, initiation	7	0,001175075	0,006935105	4,971159155
negative regulation of stress-activated MAPK cascade	3	0,001188895	0,006973884	4,965582987
T cell homeostasis	3	0,001188895	0,006973884	4,965582987
programmed necrotic cell death	3	0,001188895	0,006973884	4,965582987
negative regulation of stress-activated protein kinase signaling cascade	3	0,001188895	0,006973884	4,965582987
regulation of ion transmembrane transport	8	0,001196567	0,007008203	4,960673942
regulation of catalytic activity	23	0,00119859	0,007009385	4,960505255
activation of cysteine-type endopeptidase activity involved in apoptotic process	4	0,00120485	0,007024643	4,958330889
positive regulation of peptidyl-serine phosphorylation	4	0,00120485	0,007024643	4,958330889
positive regulation of peptidase activity	5	0,001213987	0,007067203	4,952290521
autophagy	9	0,001233118	0,007167732	4,938165961
negative regulation of cysteine-type endopeptidase activity	4	0,001254766	0,007282562	4,922272613
response to cadmium ion	3	0,001275656	0,007392659	4,907267736
hormone secretion	7	0,001285986	0,007441316	4,900707551
positive regulation of peptide secretion	4	0,001306111	0,00754642	4,886682001
organonitrogen compound metabolic process	22	0,001315279	0,007587995	4,881187857
gliogenesis	6	0,001335778	0,007690913	4,867715731
aromatic compound biosynthetic process	35	0,001337116	0,007690913	4,867715731
B cell activation	6	0,001364806	0,007815751	4,851614165
positive regulation of glucose transport	3	0,001366306	0,007815751	4,851614165
dopamine biosynthetic process	2	0,001374068	0,007815751	4,851614165
vitamin D biosynthetic process	2	0,001374068	0,007815751	4,851614165
regulation of removal of superoxide radicals	2	0,001374068	0,007815751	4,851614165
regulation of oxidative stress-induced neuron death	2	0,001374068	0,007815751	4,851614165
post-embryonic organ morphogenesis	2	0,001374068	0,007815751	4,851614165
head development	11	0,001375069	0,007815751	4,851614165
cellular response to molecule of bacterial origin	5	0,001396326	0,007924872	4,837749171
macromolecule biosynthetic process	40	0,001414243	0,008014736	4,826473441
cytoplasmic transport	15	0,001440009	0,008148755	4,809890068
angiogenesis	8	0,001457466	0,008235434	4,799309192
regulation of endothelial cell proliferation	4	0,001468947	0,008288135	4,792930352
regulation of metal ion transport	7	0,001506192	0,00848584	4,769356433
regulation of cell adhesion	10	0,001509854	0,008494033	4,768391329
neuron projection morphogenesis	12	0,001523651	0,008559138	4,76075584
positive regulation of kinase activity	10	0,001527493	0,00856821	4,759696395

peptidyl-tyrosine phosphorylation	7	0,001532372	0,008583068	4,757963819
negative regulation of RNA biosynthetic process	15	0,001559375	0,008709811	4,743305185
import into cell	3	0,001559527	0,008709811	4,743305185
regulation of cellular biosynthetic process	34	0,001565829	0,008732333	4,740722755
negative regulation of cellular biosynthetic process	17	0,001575858	0,008775546	4,735786238
positive regulation of protein import into nucleus	4	0,001585055	0,008792725	4,73383061
nucleotide catabolic process	4	0,001585055	0,008792725	4,73383061
peptidyl-tyrosine modification	7	0,001585798	0,008792725	4,73383061
regulation of locomotion	11	0,001611994	0,008918133	4,719668697
regulation of DNA metabolic process	7	0,001613051	0,008918133	4,719668697
endomembrane system organization	9	0,001624858	0,008955655	4,715470157
insulin receptor signaling pathway	7	0,001640668	0,008955655	4,715470157
3'-UTR-mediated mRNA stabilization	2	0,001643421	0,008955655	4,715470157
positive regulation of execution phase of apoptosis	2	0,001643421	0,008955655	4,715470157
response to cobalt ion	2	0,001643421	0,008955655	4,715470157
response to methylmercury	2	0,001643421	0,008955655	4,715470157
regulation of vitamin metabolic process	2	0,001643421	0,008955655	4,715470157
myelin maintenance	2	0,001643421	0,008955655	4,715470157
regulation of superoxide anion generation	2	0,001643421	0,008955655	4,715470157
neuron death in response to oxidative stress	2	0,001643421	0,008955655	4,715470157
regulation of smooth muscle cell proliferation	4	0,001645439	0,008955655	4,715470157
response to antibiotic	3	0,001662223	0,008996107	4,710963318
hydrogen peroxide metabolic process	3	0,001662223	0,008996107	4,710963318
regulation of alcohol biosynthetic process	3	0,001662223	0,008996107	4,710963318
regulation of extrinsic apoptotic signaling pathway in absence of ligand	3	0,001662223	0,008996107	4,710963318
peptide transport	7	0,001697009	0,009171478	4,69165681
regulation of protein binding	5	0,001729082	0,009331707	4,674337368
cellular nitrogen compound catabolic process	8	0,001761811	0,009481748	4,658386598
heterocycle catabolic process	8	0,001761811	0,009481748	4,658386598
positive regulation of protein import	4	0,001770975	0,009517756	4,654596218
transcription, DNA-templated	31	0,001786867	0,009589769	4,647058446
positive regulation of proteolysis involved in cellular protein catabolic process	5	0,001820648	0,009757456	4,629723567
positive regulation of epithelial cell migration	4	0,001836171	0,009826963	4,62262536
regulation of macroautophagy	4	0,001903014	0,010156445	4,589646778
smooth muscle cell proliferation	4	0,001903014	0,010156445	4,589646778
nucleic acid-templated transcription	31	0,001912842	0,010185911	4,586749782
organic hydroxy compound metabolic process	9	0,001913829	0,010185911	4,586749782
inorganic cation import into cell	2	0,001935795	0,01026604	4,578913931
inorganic ion import into cell	2	0,001935795	0,01026604	4,578913931
cellular aromatic compound metabolic process	43	0,001936888	0,01026604	4,578913931
positive regulation of protein transport	8	0,001945403	0,010296987	4,575903967
regulation of cellular macromolecule biosynthetic process	32	0,001968297	0,01037815	4,568052655
protein localization to nucleus	7	0,001969474	0,01037815	4,568052655

synaptic vesicle transport	4	0,001971525	0,01037815	4,568052655
establishment of synaptic vesicle localization	4	0,001971525	0,01037815	4,568052655
response to endoplasmic reticulum stress	6	0,001974585	0,010380058	4,567868795
purine nucleotide catabolic process	3	0,001995373	0,010416908	4,564324977
regulation of myoblast differentiation	3	0,001995373	0,010416908	4,564324977
necrotic cell death	3	0,001995373	0,010416908	4,564324977
neuron projection regeneration	3	0,001995373	0,010416908	4,564324977
T cell apoptotic process	3	0,001995373	0,010416908	4,564324977
reproductive structure development	8	0,002000545	0,010416908	4,564324977
negative regulation of intracellular signal transduction	8	0,002000545	0,010416908	4,564324977
platelet activation	6	0,002013653	0,010470996	4,559146118
aromatic compound catabolic process	8	0,002028575	0,010520158	4,554462054
regulation of homeostatic process	8	0,002028575	0,010520158	4,554462054
limbic system development	4	0,002041726	0,010574105	4,549347164
myotube differentiation	4	0,002113637	0,010845731	4,523983733
reproductive system development	8	0,002114535	0,010845731	4,523983733
protein import into nucleus, translocation	3	0,002114971	0,010845731	4,523983733
negative regulation of autophagy	3	0,002114971	0,010845731	4,523983733
base-excision repair	3	0,002114971	0,010845731	4,523983733
response to cocaine	3	0,002114971	0,010845731	4,523983733
regulation of neurotransmitter levels	5	0,002116721	0,010845731	4,523983733
positive regulation of DNA metabolic process	5	0,002116721	0,010845731	4,523983733
lymphocyte proliferation	6	0,002134379	0,010921665	4,51700684
negative regulation of RNA metabolic process	15	0,002139877	0,010935255	4,515763285
protein complex subunit organization	19	0,002145498	0,010949437	4,514467245
negative regulation of nucleobase-containing compound metabolic process	16	0,002153823	0,010977365	4,511919871
regulation of glucose metabolic process	4	0,002187282	0,011133148	4,497828349
cellular protein modification process	32	0,002206427	0,011200964	4,49175546
protein modification process	32	0,002206427	0,011200964	4,49175546
organic cyclic compound metabolic process	44	0,002211988	0,011214401	4,490556518
organelle localization	8	0,002233594	0,011291522	4,483703081
positive regulation of reactive oxygen species biosynthetic process	3	0,002238937	0,011291522	4,483703081
regulation of nucleoside metabolic process	3	0,002238937	0,011291522	4,483703081
regulation of ATP metabolic process	3	0,002238937	0,011291522	4,483703081
system process	20	0,00224529	0,011292924	4,483578907
positive regulation of transcription regulatory region DNA binding	2	0,002250955	0,011292924	4,483578907
fat-soluble vitamin biosynthetic process	2	0,002250955	0,011292924	4,483578907
lactate metabolic process	2	0,002250955	0,011292924	4,483578907
mononuclear cell proliferation	6	0,002260506	0,011326079	4,480647329
regulation of cytoplasmic transport	8	0,00226417	0,011329682	4,480329233
regulation of protein complex assembly	7	0,002274781	0,011367999	4,476953003
macromolecule modification	33	0,002286749	0,011412982	4,473003778
neurotransmitter transport	5	0,002332717	0,011627323	4,454397528

protein homooligomerization	6	0,002347667	0,011671605	4,45059633
cellular response to steroid hormone stimulus	6	0,002347667	0,011671605	4,45059633
blood coagulation	9	0,00235249	0,011680494	4,449835041
positive regulation of neuron apoptotic process	3	0,002367327	0,011708834	4,447411722
response to nicotine	3	0,002367327	0,011708834	4,447411722
regulation of steroid biosynthetic process	3	0,002367327	0,011708834	4,447411722
immune response	18	0,002379083	0,011751877	4,443742342
glial cell differentiation	5	0,002389134	0,011786395	4,4408094
positive regulation of immune response	10	0,002409371	0,011871012	4,433655797
synaptic vesicle localization	4	0,002418819	0,011902323	4,431021711
regulation of nitrogen compound metabolic process	34	0,002452646	0,01205336	4,41841184
amide transport	7	0,002459536	0,012071805	4,416882682
hemostasis	9	0,002495735	0,012233872	4,403546801
establishment of protein localization to membrane	7	0,002536601	0,012418371	4,388578379
female gonad development	4	0,002582226	0,012593147	4,374602535
sequestering of triglyceride	2	0,002588664	0,012593147	4,374602535
response to vitamin E	2	0,002588664	0,012593147	4,374602535
regulation of proton transport	2	0,002588664	0,012593147	4,374602535
positive regulation of nuclease activity	2	0,002588664	0,012593147	4,374602535
generation of precursor metabolites and energy	8	0,002622765	0,012720605	4,364532138
positive regulation of cytoplasmic transport	6	0,002624414	0,012720605	4,364532138
positive regulation of cellular protein catabolic process	5	0,002624782	0,012720605	4,364532138
acute-phase response	3	0,002637585	0,012750538	4,362181845
spliceosomal complex assembly	3	0,002637585	0,012750538	4,362181845
epidermal growth factor receptor signaling pathway	7	0,002655677	0,012798352	4,358438835
response to light stimulus	7	0,002655677	0,012798352	4,358438835
activation of protein kinase activity	8	0,002657454	0,012798352	4,358438835
endothelial cell proliferation	4	0,002666706	0,01280826	4,357664978
protein import into nucleus	6	0,002672826	0,01280826	4,357664978
protein targeting to nucleus	6	0,002672826	0,01280826	4,357664978
single-organism nuclear import	6	0,002672826	0,01280826	4,357664978
regulation of MAP kinase activity	8	0,002692502	0,012886503	4,351574756
heterocycle biosynthetic process	34	0,00271972	0,013000601	4,342759683
positive regulation of nucleocytoplasmic transport	4	0,002753065	0,013143666	4,331815315
anatomical structure homeostasis	7	0,002779019	0,013237303	4,324716463
regulation of morphogenesis of a branching structure	3	0,002779558	0,013237303	4,324716463
transcription initiation from RNA polymerase II promoter	6	0,002822095	0,013423267	4,310765754
nuclear import	6	0,002873217	0,013649552	4,294048557
cell migration	14	0,002877692	0,013653958	4,293725816
purine-containing compound catabolic process	3	0,002926161	0,013866833	4,278255393
myoblast proliferation	2	0,002948691	0,013895572	4,276185077
negative regulation of hydrogen peroxide-induced cell death	2	0,002948691	0,013895572	4,276185077
regulation of NFAT protein import into nucleus	2	0,002948691	0,013895572	4,276185077

regulation of oxidative phosphorylation	2	0,002948691	0,013895572	4,276185077
ERBB signaling pathway	7	0,002950281	0,013895572	4,276185077
brain development	10	0,002968505	0,01396431	4,27125051
cellular nitrogen compound metabolic process	46	0,002973071	0,013968714	4,270935164
regulation of nucleobase-containing compound metabolic process	32	0,002977302	0,013971534	4,27073332
development of primary female sexual characteristics	4	0,003023624	0,014154384	4,257730899
positive regulation of hormone secretion	4	0,003023624	0,014154384	4,257730899
cellular macromolecule metabolic process	56	0,003035384	0,014191357	4,255122196
positive regulation of neurogenesis	7	0,003038898	0,014191357	4,255122196
organic cyclic compound catabolic process	8	0,003063308	0,014288011	4,248334497
regulation of stress-activated MAPK cascade	5	0,003077138	0,014319226	4,246152163
synaptic vesicle exocytosis	3	0,003077443	0,014319226	4,246152163
proteasome-mediated ubiquitin-dependent protein catabolic process	7	0,003083965	0,014332244	4,245243445
regulation of stress-activated protein kinase signaling cascade	5	0,003146076	0,014603257	4,226510715
developmental growth	9	0,003175137	0,014720396	4,218521271
organ morphogenesis	12	0,003183883	0,014743177	4,216974868
RNA biosynthetic process	31	0,003247519	0,015019777	4,198387482
fat cell differentiation	5	0,003287289	0,015167251	4,188616736
regulation of muscle system process	5	0,003287289	0,015167251	4,188616736
negative regulation of release of cytochrome c from mitochondria	2	0,003330804	0,01524011	4,183824539
misfolded or incompletely synthesized protein catabolic process	2	0,003330804	0,01524011	4,183824539
hemoglobin metabolic process	2	0,003330804	0,01524011	4,183824539
NFAT protein import into nucleus	2	0,003330804	0,01524011	4,183824539
regulation of receptor binding	2	0,003330804	0,01524011	4,183824539
post-embryonic morphogenesis	2	0,003330804	0,01524011	4,183824539
negative regulation of receptor-mediated endocytosis	2	0,003330804	0,01524011	4,183824539
positive regulation of cell adhesion	7	0,003365244	0,015361161	4,175912996
forebrain development	7	0,003365244	0,015361161	4,175912996
protein localization to membrane	8	0,003387752	0,01544558	4,170432385
cardiac muscle hypertrophy	3	0,003394233	0,015456813	4,169705421
positive regulation of angiogenesis	4	0,003411918	0,015491707	4,167450402
ovulation cycle	4	0,003411918	0,015491707	4,167450402
cellular protein metabolic process	38	0,003413973	0,015491707	4,167450402
positive regulation of transferase activity	10	0,003487045	0,015804651	4,147451021
positive regulation of cysteine-type endopeptidase activity involved in apoptotic process	4	0,003514046	0,015908292	4,140914819
organelle transport along microtubule	3	0,003559833	0,016090963	4,129497455
regulation of transcription, DNA-templated	29	0,00356276	0,016090963	4,129497455
heterocycle metabolic process	42	0,003579073	0,016145688	4,126102279
morphogenesis of a branching epithelium	5	0,00358335	0,016146054	4,126079593
protein secretion	8	0,003603741	0,01621894	4,121575573
regulation of body fluid levels	10	0,003627368	0,016306208	4,116209414
positive regulation of MAPK cascade	9	0,003664166	0,016452406	4,107283562
regulation of hormone levels	8	0,003693076	0,016562887	4,100590834

positive regulation of protein serine/threonine kinase activity	6	0,003726382	0,016595158	4,098644292
striated muscle hypertrophy	3	0,003730298	0,016595158	4,098644292
apoptotic DNA fragmentation	2	0,003734773	0,016595158	4,098644292
inner mitochondrial membrane organization	2	0,003734773	0,016595158	4,098644292
nitric-oxide synthase biosynthetic process	2	0,003734773	0,016595158	4,098644292
regulation of nitric-oxide synthase biosynthetic process	2	0,003734773	0,016595158	4,098644292
negative regulation of response to reactive oxygen species	2	0,003734773	0,016595158	4,098644292
post-embryonic organ development	2	0,003734773	0,016595158	4,098644292
macromolecule metabolic process	59	0,003748417	0,016636574	4,096151757
muscle system process	7	0,003770352	0,016714647	4,091469898
steroid metabolic process	6	0,003789285	0,016779249	4,087612361
response to biotic stimulus	12	0,003796818	0,016793284	4,086776248
negative regulation of nucleic acid-templated transcription	14	0,003813344	0,016847013	4,083581909
regulation of epithelial cell differentiation	4	0,003832879	0,016894521	4,08076593
positive regulation of binding	4	0,003832879	0,016894521	4,08076593
regulation of nucleic acid-templated transcription	29	0,00388404	0,01710044	4,068651114
positive regulation of phosphatidylinositol 3-kinase signaling	3	0,00390567	0,017156413	4,065383215
regulation of transcription from RNA polymerase II promoter in response to stress	3	0,00390567	0,017156413	4,065383215
nucleobase-containing compound catabolic process	7	0,00393149	0,017250139	4,059935069
organic substance biosynthetic process	44	0,003976587	0,017428141	4,04966908
negative regulation of ion transport	4	0,004056005	0,017715672	4,033305635
regulation of response to extracellular stimulus	4	0,004056005	0,017715672	4,033305635
regulation of response to nutrient levels	4	0,004056005	0,017715672	4,033305635
muscle hypertrophy	3	0,004085992	0,017826416	4,027073902
thymocyte apoptotic process	2	0,004160372	0,018052172	4,014489293
vitamin D metabolic process	2	0,004160372	0,018052172	4,014489293
regulation of hydrogen peroxide-induced cell death	2	0,004160372	0,018052172	4,014489293
epithelium development	13	0,004160996	0,018052172	4,014489293
nucleobase-containing compound biosynthetic process	33	0,004165452	0,018052172	4,014489293
blood vessel morphogenesis	8	0,004165886	0,018052172	4,014489293
positive regulation of NF-kappaB transcription factor activity	4	0,004170798	0,018053127	4,01443637
regulation of RNA biosynthetic process	29	0,004212059	0,018211238	4,005716381
regulation of tissue remodeling	3	0,004271306	0,018446675	3,992871145
regulation of gene expression	33	0,004282054	0,018472359	3,991479788
calcium ion transport into cytosol	4	0,00428777	0,018476302	3,991266345
positive regulation of MAP kinase activity	5	0,00431892	0,01854822	3,987381453
osteoblast differentiation	5	0,00431892	0,01854822	3,987381453
microtubule-based movement	5	0,00431892	0,01854822	3,987381453
nitrogen compound metabolic process	47	0,0043468	0,018647142	3,982062415
wound healing	10	0,004356698	0,01866879	3,980902109
transmembrane transport	15	0,004377922	0,018734631	3,977381535
cell part morphogenesis	13	0,004386239	0,018734631	3,977381535
proteasomal protein catabolic process	7	0,004386878	0,018734631	3,977381535

receptor-mediated endocytosis	6	0,004391538	0,018734631	3,977381535
positive regulation of transmembrane transport	4	0,00440694	0,018779517	3,974988542
neurotrophin TRK receptor signaling pathway	7	0,0044465	0,018927136	3,967158622
ovarian follicle development	3	0,004461653	0,018949714	3,965966434
positive regulation of endothelial cell migration	3	0,004461653	0,018949714	3,965966434
regulation of cation transmembrane transport	5	0,004495991	0,019074503	3,959402755
cytosolic calcium ion transport	4	0,004528327	0,01919053	3,953338369
negative regulation of hydrolase activity	7	0,004567586	0,019335612	3,945806694
mitochondrial DNA metabolic process	2	0,004607373	0,019397344	3,942619111
response to vitamin A	2	0,004607373	0,019397344	3,942619111
response to lead ion	2	0,004607373	0,019397344	3,942619111
catechol-containing compound biosynthetic process	2	0,004607373	0,019397344	3,942619111
catecholamine biosynthetic process	2	0,004607373	0,019397344	3,942619111
neurotrophin signaling pathway	7	0,004629059	0,019405679	3,942189532
carbohydrate metabolic process	12	0,004647841	0,019405679	3,942189532
regulation of lipid biosynthetic process	4	0,00465195	0,019405679	3,942189532
gland morphogenesis	4	0,00465195	0,019405679	3,942189532
female sex differentiation	4	0,00465195	0,019405679	3,942189532
positive regulation of insulin secretion	3	0,004657072	0,019405679	3,942189532
lymphocyte homeostasis	3	0,004657072	0,019405679	3,942189532
regulation of hydrolase activity	14	0,00468451	0,019405679	3,942189532
regulation of system process	8	0,004738746	0,019405679	3,942189532
positive regulation of vasculature development	4	0,004777828	0,019405679	3,942189532
axonogenesis	10	0,004829351	0,019405679	3,942189532
negative regulation of nitrogen compound metabolic process	16	0,004856531	0,019405679	3,942189532
regulation of DNA-templated transcription in response to stress	3	0,004857601	0,019405679	3,942189532
regulation of nucleocytoplasmic transport	5	0,004865584	0,019405679	3,942189532
endocytosis	9	0,004918953	0,019405679	3,942189532
regulation of protein transport	10	0,005057178	0,019405679	3,942189532
negative regulation of lipid catabolic process	2	0,005075554	0,019405679	3,942189532
endoplasmic reticulum calcium ion homeostasis	2	0,005075554	0,019405679	3,942189532
regulation of protein homodimerization activity	2	0,005075554	0,019405679	3,942189532
negative regulation of cytokine production involved in immune response	2	0,005075554	0,019405679	3,942189532
cell death in response to hydrogen peroxide	2	0,005075554	0,019405679	3,942189532
positive regulation of lymphocyte apoptotic process	2	0,005075554	0,019405679	3,942189532
regulation of cellular respiration	2	0,005075554	0,019405679	3,942189532
negative regulation of branching involved in lung morphogenesis	1	0,005103573	0,019405679	3,942189532
Cajal-Retzius cell differentiation	1	0,005103573	0,019405679	3,942189532
nitric oxide storage	1	0,005103573	0,019405679	3,942189532
glycogen cell differentiation involved in embryonic placenta development	1	0,005103573	0,019405679	3,942189532
positive regulation of calcium-dependent cell-cell adhesion	1	0,005103573	0,019405679	3,942189532
positive regulation of DNA-templated transcription, termination	1	0,005103573	0,019405679	3,942189532
positive regulation of termination of RNA polymerase II transcription, poly(A)-coupled	1	0,005103573	0,019405679	3,942189532

negative regulation of integrin biosynthetic process	1	0,005103573	0,019405679	3,942189532
flavin adenine dinucleotide catabolic process	1	0,005103573	0,019405679	3,942189532
positive regulation of protein K63-linked deubiquitination	1	0,005103573	0,019405679	3,942189532
positive regulation of Lys63-specific deubiquitinase activity	1	0,005103573	0,019405679	3,942189532
release of matrix enzymes from mitochondria	1	0,005103573	0,019405679	3,942189532
B cell receptor apoptotic signaling pathway	1	0,005103573	0,019405679	3,942189532
negative regulation of erythrocyte apoptotic process	1	0,005103573	0,019405679	3,942189532
intermediate-density lipoprotein particle remodeling	1	0,005103573	0,019405679	3,942189532
nuclear inner membrane organization	1	0,005103573	0,019405679	3,942189532
D-serine catabolic process	1	0,005103573	0,019405679	3,942189532
D-alanine catabolic process	1	0,005103573	0,019405679	3,942189532
age-dependent response to reactive oxygen species	1	0,005103573	0,019405679	3,942189532
vasodilation by acetylcholine involved in regulation of systemic arterial blood pressure	1	0,005103573	0,019405679	3,942189532
negative regulation of T cell antigen processing and presentation	1	0,005103573	0,019405679	3,942189532
cellular response to iron ion starvation	1	0,005103573	0,019405679	3,942189532
negative regulation of antigen processing and presentation of endogenous peptide antigen via MHC class I	1	0,005103573	0,019405679	3,942189532
response to iron ion starvation	1	0,005103573	0,019405679	3,942189532
positive regulation of connective tissue growth factor production	1	0,005103573	0,019405679	3,942189532
negative regulation of lung ciliated cell differentiation	1	0,005103573	0,019405679	3,942189532
positive regulation of lung goblet cell differentiation	1	0,005103573	0,019405679	3,942189532
positive regulation of pancreatic stellate cell proliferation	1	0,005103573	0,019405679	3,942189532
negative regulation of cellular organofluorine metabolic process	1	0,005103573	0,019405679	3,942189532
enzyme active site formation via L-cysteine sulfinic acid	1	0,005103573	0,019405679	3,942189532
cellular response to glyoxal	1	0,005103573	0,019405679	3,942189532
glycolate biosynthetic process	1	0,005103573	0,019405679	3,942189532
detoxification of mercury ion	1	0,005103573	0,019405679	3,942189532
negative regulation of death-inducing signaling complex assembly	1	0,005103573	0,019405679	3,942189532
negative regulation of TRAIL-activated apoptotic signaling pathway	1	0,005103573	0,019405679	3,942189532
positive regulation of pyrroline-5-carboxylate reductase activity	1	0,005103573	0,019405679	3,942189532
positive regulation of tyrosine 3-monooxygenase activity	1	0,005103573	0,019405679	3,942189532
positive regulation of dopamine biosynthetic process	1	0,005103573	0,019405679	3,942189532
glyoxal catabolic process	1	0,005103573	0,019405679	3,942189532
positive regulation of L-dopa biosynthetic process	1	0,005103573	0,019405679	3,942189532
positive regulation of L-dopa decarboxylase activity	1	0,005103573	0,019405679	3,942189532
positive regulation of oxidative phosphorylation uncoupler activity	1	0,005103573	0,019405679	3,942189532
negative regulation of G-protein coupled receptor internalization	1	0,005103573	0,019405679	3,942189532
regulation of DNA-templated transcription, termination	1	0,005103573	0,019405679	3,942189532
termination of RNA polymerase II transcription, poly(A)-coupled	1	0,005103573	0,019405679	3,942189532
regulation of termination of RNA polymerase II transcription	1	0,005103573	0,019405679	3,942189532
positive regulation of termination of RNA polymerase II transcription	1	0,005103573	0,019405679	3,942189532
regulation of termination of RNA polymerase II transcription, poly(A)-coupled	1	0,005103573	0,019405679	3,942189532
flavin-containing compound catabolic process	1	0,005103573	0,019405679	3,942189532
regulation of protein K63-linked deubiquitination	1	0,005103573	0,019405679	3,942189532

positive regulation of ubiquitin-specific protease activity	1	0,005103573	0,019405679	3,942189532
erythrocyte apoptotic process	1	0,005103573	0,019405679	3,942189532
regulation of erythrocyte apoptotic process	1	0,005103573	0,019405679	3,942189532
D-alanine family amino acid metabolic process	1	0,005103573	0,019405679	3,942189532
D-alanine metabolic process	1	0,005103573	0,019405679	3,942189532
regulation of systemic arterial blood pressure by acetylcholine	1	0,005103573	0,019405679	3,942189532
regulation of systemic arterial blood pressure by neurotransmitter	1	0,005103573	0,019405679	3,942189532
regulation of antigen processing and presentation of endogenous peptide antigen via MHC class I	1	0,005103573	0,019405679	3,942189532
regulation of connective tissue growth factor production	1	0,005103573	0,019405679	3,942189532
regulation of lung goblet cell differentiation	1	0,005103573	0,019405679	3,942189532
regulation of cellular organohalogen metabolic process	1	0,005103573	0,019405679	3,942189532
regulation of cellular organofluorine metabolic process	1	0,005103573	0,019405679	3,942189532
negative regulation of cellular organohalogen metabolic process	1	0,005103573	0,019405679	3,942189532
primary alcohol biosynthetic process	1	0,005103573	0,019405679	3,942189532
regulation of death-inducing signaling complex assembly	1	0,005103573	0,019405679	3,942189532
regulation of pyrroline-5-carboxylate reductase activity	1	0,005103573	0,019405679	3,942189532
regulation of tyrosine 3-monooxygenase activity	1	0,005103573	0,019405679	3,942189532
regulation of dopamine biosynthetic process	1	0,005103573	0,019405679	3,942189532
glyoxal metabolic process	1	0,005103573	0,019405679	3,942189532
L-dopa metabolic process	1	0,005103573	0,019405679	3,942189532
L-dopa biosynthetic process	1	0,005103573	0,019405679	3,942189532
regulation of L-dopa biosynthetic process	1	0,005103573	0,019405679	3,942189532
positive regulation of cellular amino acid biosynthetic process	1	0,005103573	0,019405679	3,942189532
regulation of L-dopa decarboxylase activity	1	0,005103573	0,019405679	3,942189532
regulation of G-protein coupled receptor internalization	1	0,005103573	0,019405679	3,942189532
single-organism organelle organization	23	0,00515409	0,019578421	3,933327311
cellular response to hypoxia	4	0,005169177	0,019616362	3,931391255
regulation of homotypic cell-cell adhesion	6	0,005220578	0,019791906	3,922482203
glutamate receptor signaling pathway	3	0,005274141	0,01995565	3,914242972
regulation of reactive oxygen species biosynthetic process	3	0,005274141	0,01995565	3,914242972
biosynthetic process	44	0,005299608	0,020032311	3,910408747
epithelial cell migration	5	0,005357369	0,02023077	3,900550569
lymphocyte apoptotic process	3	0,005490225	0,020712142	3,877035159
inflammatory response	9	0,005549823	0,0208703	3,869428195
cellular nitrogen compound biosynthetic process	36	0,005563893	0,0208703	3,869428195
positive regulation of protein oligomerization	2	0,00556469	0,0208703	3,869428195
positive regulation of translational initiation	2	0,00556469	0,0208703	3,869428195
myelination in peripheral nervous system	2	0,00556469	0,0208703	3,869428195
peripheral nervous system axon ensheathment	2	0,00556469	0,0208703	3,869428195
activation of MAPK activity	4	0,005581484	0,020872255	3,869334511
neurotransmitter secretion	4	0,005581484	0,020872255	3,869334511
acute inflammatory response	4	0,005581484	0,020872255	3,869334511
establishment of vesicle localization	5	0,005668805	0,021157674	3,855752585

epithelium migration	5	0,005668805	0,021157674	3,855752585
immune response-regulating cell surface receptor signaling pathway	8	0,005678235	0,021172332	3,855060048
transmission of nerve impulse	3	0,005711564	0,021255414	3,851143633
positive regulation of autophagy	3	0,005711564	0,021255414	3,851143633
neural precursor cell proliferation	4	0,005723662	0,021279856	3,849994387
developmental process involved in reproduction	9	0,005773535	0,02144456	3,842284267
cellular macromolecule biosynthetic process	37	0,005920028	0,021949467	3,819012427
single-organism metabolic process	40	0,00592088	0,021949467	3,819012427
blood vessel endothelial cell migration	3	0,005938193	0,021971313	3,81801765
mammary gland epithelium development	3	0,005938193	0,021971313	3,81801765
protein import	6	0,005980193	0,022105458	3,811930713
nucleic acid metabolic process	37	0,006069063	0,022283044	3,803929241
superoxide anion generation	2	0,006074562	0,022283044	3,803929241
L-glutamate transport	2	0,006074562	0,022283044	3,803929241
positive regulation of blood vessel endothelial cell migration	2	0,006074562	0,022283044	3,803929241
mitochondrial fusion	2	0,006074562	0,022283044	3,803929241
vitamin biosynthetic process	2	0,006074562	0,022283044	3,803929241
regulation of nuclease activity	2	0,006074562	0,022283044	3,803929241
DNA catabolic process, endonucleolytic	2	0,006074562	0,022283044	3,803929241
cellular response to decreased oxygen levels	4	0,006164671	0,022584455	3,79049345
macromolecule catabolic process	13	0,006168467	0,022584455	3,79049345
cell motility	14	0,00618722	0,022610088	3,789359105
localization of cell	14	0,00618722	0,022610088	3,789359105
protein autophosphorylation	5	0,006215994	0,022650707	3,78756421
tissue migration	5	0,006215994	0,022650707	3,78756421
response to temperature stimulus	5	0,006215994	0,022650707	3,78756421
nucleocytoplasmic transport	7	0,006223563	0,02265683	3,787293946
organic hydroxy compound biosynthetic process	5	0,006329739	0,023021585	3,771323017
axon development	10	0,006382211	0,023190508	3,764012242
intrinsic apoptotic signaling pathway by p53 class mediator	3	0,006407449	0,023202597	3,763491047
positive regulation of protein binding	3	0,006407449	0,023202597	3,763491047
peripheral nervous system development	3	0,006407449	0,023202597	3,763491047
regulation of RNA metabolic process	29	0,006409657	0,023202597	3,763491047
negative regulation of MAPK cascade	4	0,006470914	0,023402329	3,754919742
multicellular organismal reproductive process	10	0,006494417	0,023465274	3,752233652
regulation of intrinsic apoptotic signaling pathway by p53 class mediator	2	0,006604949	0,023753125	3,740041161
branching involved in salivary gland morphogenesis	2	0,006604949	0,023753125	3,740041161
regulation of execution phase of apoptosis	2	0,006604949	0,023753125	3,740041161
positive regulation of nucleoside metabolic process	2	0,006604949	0,023753125	3,740041161
positive regulation of ATP metabolic process	2	0,006604949	0,023753125	3,740041161
nuclear transport	7	0,006618754	0,023758363	3,739820672
single-organism behavior	7	0,006618754	0,023758363	3,739820672
nuclear envelope organization	3	0,00665014	0,023848779	3,736022245

cell junction assembly	5	0,006679763	0,023932706	3,732509303
positive regulation of nervous system development	7	0,0068647	0,024572432	3,706130111
hippocampus development	3	0,006898247	0,024646663	3,703113753
negative regulation of protein binding	3	0,006898247	0,024646663	3,703113753
immune response-regulating signaling pathway	9	0,00699913	0,024983907	3,689523396
vesicle localization	5	0,00704317	0,025117811	3,684178083
small GTPase mediated signal transduction	11	0,007068034	0,025183142	3,681580487
regulation of receptor-mediated endocytosis	3	0,0071518	0,025424638	3,672036585
cellular response to epidermal growth factor stimulus	2	0,007155635	0,025424638	3,672036585
skeletal muscle adaptation	2	0,007155635	0,025424638	3,672036585
regulation of ERK1 and ERK2 cascade	5	0,007293005	0,02588882	3,653944047
regulation of epidermal growth factor receptor signaling pathway	3	0,007410826	0,026282819	3,638839821
negative regulation of endopeptidase activity	5	0,007548978	0,02672352	3,622211199
negative regulation of growth	5	0,007548978	0,02672352	3,622211199
placenta development	4	0,007622013	0,026907804	3,615338922
endothelial cell migration	4	0,007622013	0,026907804	3,615338922
alcohol biosynthetic process	4	0,007622013	0,026907804	3,615338922
positive regulation of cell cycle	6	0,007631147	0,026915356	3,615058299
peptidyl-threonine phosphorylation	3	0,007675354	0,026954848	3,613592097
endoderm development	3	0,007675354	0,026954848	3,613592097
glial cell development	3	0,007675354	0,026954848	3,613592097
regulation of phosphatidylinositol 3-kinase signaling	3	0,007675354	0,026954848	3,613592097
negative regulation of myoblast differentiation	2	0,007726403	0,026954848	3,613592097
negative regulation of extrinsic apoptotic signaling pathway via death domain receptors	2	0,007726403	0,026954848	3,613592097
response to corticosterone	2	0,007726403	0,026954848	3,613592097
negative regulation of endothelial cell apoptotic process	2	0,007726403	0,026954848	3,613592097
Schwann cell development	2	0,007726403	0,026954848	3,613592097
acidic amino acid transport	2	0,007726403	0,026954848	3,613592097
positive regulation of tissue remodeling	2	0,007726403	0,026954848	3,613592097
apoptotic process involved in morphogenesis	2	0,007726403	0,026954848	3,613592097
protein catabolic process	10	0,007768189	0,02707608	3,609104581
striated muscle tissue development	6	0,007845024	0,027319144	3,600167589
negative regulation of lipid metabolic process	3	0,00794541	0,027618732	3,589261043
regulation of steroid metabolic process	3	0,00794541	0,027618732	3,589261043
multicellular organism reproduction	10	0,007964968	0,027661729	3,587705455
response to other organism	11	0,00800139	0,027738153	3,58494644
response to external biotic stimulus	11	0,00800139	0,027738153	3,58494644
multicellular organism growth	4	0,00815407	0,028241998	3,566945122
positive regulation of exocytosis	3	0,00822102	0,028397203	3,561464627
positive regulation of myeloid cell differentiation	3	0,00822102	0,028397203	3,561464627
regulation of ERBB signaling pathway	3	0,00822102	0,028397203	3,561464627
energy homeostasis	2	0,008317039	0,028677387	3,55164636
regulation of protein localization to cell surface	2	0,008317039	0,028677387	3,55164636

monovalent inorganic cation transport	8	0,008478946	0,029143014	3,535540059
organ regeneration	3	0,008502209	0,029143014	3,535540059
myoblast differentiation	3	0,008502209	0,029143014	3,535540059
negative regulation of proteolysis involved in cellular protein catabolic process	3	0,008502209	0,029143014	3,535540059
leukocyte homeostasis	3	0,008502209	0,029143014	3,535540059
receptor internalization	3	0,008502209	0,029143014	3,535540059
single-organism carbohydrate metabolic process	10	0,008508661	0,029143014	3,535540059
establishment of organelle localization	6	0,008512668	0,029143014	3,535540059
G1/S transition of mitotic cell cycle	5	0,008778827	0,030027491	3,505641953
peptidyl-threonine modification	3	0,008789002	0,030035596	3,505372054
striated muscle cell differentiation	5	0,008923537	0,030427249	3,492416722
regulation of endothelial cell differentiation	2	0,00892733	0,030427249	3,492416722
negative regulation of production of molecular mediator of immune response	2	0,00892733	0,030427249	3,492416722
small molecule catabolic process	6	0,008979914	0,030579388	3,487429108
ERK1 and ERK2 cascade	5	0,009069893	0,030831227	3,479227251
negative regulation of peptidase activity	5	0,009069893	0,030831227	3,479227251
positive regulation of peptidyl-tyrosine phosphorylation	4	0,00909406	0,030886093	3,477449252
cellular response to inorganic substance	4	0,009290181	0,031524353	3,456994934
regulation of cytokine production	8	0,009364384	0,03174815	3,449920836
muscle structure development	8	0,009456598	0,032032562	3,441002317
muscle tissue development	6	0,009465318	0,032033898	3,440960632
cellular response to radiation	4	0,009489044	0,032059444	3,440163461
negative regulation of receptor activity	2	0,009557065	0,032059444	3,440163461
calcium-dependent cell-cell adhesion via plasma membrane cell adhesion molecules	2	0,009557065	0,032059444	3,440163461
mitochondrial genome maintenance	2	0,009557065	0,032059444	3,440163461
positive regulation of leukocyte apoptotic process	2	0,009557065	0,032059444	3,440163461
macroautophagy	6	0,009589549	0,032059444	3,440163461
regulation of anatomical structure size	7	0,009607101	0,032059444	3,440163461
cation transmembrane transport	9	0,009785197	0,032059444	3,440163461
cellular biosynthetic process	42	0,009787945	0,032059444	3,440163461
muscle organ development	6	0,009841511	0,032059444	3,440163461
regulation of cellular carbohydrate metabolic process	4	0,009895056	0,032059444	3,440163461
cell projection morphogenesis	12	0,00997389	0,032059444	3,440163461
negative regulation of cellular protein catabolic process	3	0,00999267	0,032059444	3,440163461
regulation of carbohydrate biosynthetic process	3	0,00999267	0,032059444	3,440163461
branching morphogenesis of an epithelial tube	4	0,010102233	0,032059444	3,440163461
regulation of epithelial cell migration	4	0,010102233	0,032059444	3,440163461
calcium ion transmembrane transport	5	0,010141206	0,032059444	3,440163461
cell cycle G1/S phase transition	5	0,010141206	0,032059444	3,440163461
DNA metabolic process	11	0,010156248	0,032059444	3,440163461
protein transport	17	0,010181254	0,032059444	3,440163461
positive regulation of chronic inflammatory response to antigenic stimulus	1	0,010181403	0,032059444	3,440163461
positive regulation of translational initiation by iron	1	0,010181403	0,032059444	3,440163461

smooth endoplasmic reticulum calcium ion homeostasis	1	0,010181403	0,032059444	3,440163461
positive regulation of dense core granule biogenesis	1	0,010181403	0,032059444	3,440163461
regulation of glycolytic process by positive regulation of transcription from RNA polymerase II promoter	1	0,010181403	0,032059444	3,440163461
negative regulation of macromitophagy	1	0,010181403	0,032059444	3,440163461
negative regulation of glucose catabolic process to lactate via pyruvate	1	0,010181403	0,032059444	3,440163461
calcium ion import across plasma membrane	1	0,010181403	0,032059444	3,440163461
regulation of single strand break repair	1	0,010181403	0,032059444	3,440163461
inorganic diphosphate transport	1	0,010181403	0,032059444	3,440163461
B cell negative selection	1	0,010181403	0,032059444	3,440163461
B cell homeostatic proliferation	1	0,010181403	0,032059444	3,440163461
positive regulation of apoptotic DNA fragmentation	1	0,010181403	0,032059444	3,440163461
retinal cell apoptotic process	1	0,010181403	0,032059444	3,440163461
nuclear fragmentation involved in apoptotic nuclear change	1	0,010181403	0,032059444	3,440163461
D-serine metabolic process	1	0,010181403	0,032059444	3,440163461
positive regulation of cardiac muscle hypertrophy in response to stress	1	0,010181403	0,032059444	3,440163461
positive regulation of ferrous iron import into cell	1	0,010181403	0,032059444	3,440163461
positive regulation of ferrous iron binding	1	0,010181403	0,032059444	3,440163461
positive regulation of transferrin receptor binding	1	0,010181403	0,032059444	3,440163461
negative regulation of CD8-positive, alpha-beta T cell activation	1	0,010181403	0,032059444	3,440163461
low-density lipoprotein particle mediated signaling	1	0,010181403	0,032059444	3,440163461
intermediate filament polymerization or depolymerization	1	0,010181403	0,032059444	3,440163461
cellular response to monosodium glutamate	1	0,010181403	0,032059444	3,440163461
positive regulation of tyrosine phosphorylation of Stat6 protein	1	0,010181403	0,032059444	3,440163461
negative regulation of complement-dependent cytotoxicity	1	0,010181403	0,032059444	3,440163461
detoxification of copper ion	1	0,010181403	0,032059444	3,440163461
positive regulation of mitochondrial electron transport, NADH to ubiquinone	1	0,010181403	0,032059444	3,440163461
negative regulation of protein K48-linked deubiquitination	1	0,010181403	0,032059444	3,440163461
negative regulation of hydrogen peroxide-induced neuron intrinsic apoptotic signaling pathway	1	0,010181403	0,032059444	3,440163461
negative regulation of ubiquitin-specific protease activity	1	0,010181403	0,032059444	3,440163461
positive regulation of androgen receptor activity	1	0,010181403	0,032059444	3,440163461
negative regulation of clathrin-mediated endocytosis	1	0,010181403	0,032059444	3,440163461
negative regulation of NAD metabolic process	1	0,010181403	0,032059444	3,440163461
regulation of calcium-dependent cell-cell adhesion	1	0,010181403	0,032059444	3,440163461
flavin adenine dinucleotide metabolic process	1	0,010181403	0,032059444	3,440163461
positive regulation of protein deubiquitination	1	0,010181403	0,032059444	3,440163461
B cell selection	1	0,010181403	0,032059444	3,440163461
positive regulation of DNA catabolic process	1	0,010181403	0,032059444	3,440163461
positive regulation of cardiac muscle adaptation	1	0,010181403	0,032059444	3,440163461
regulation of T cell antigen processing and presentation	1	0,010181403	0,032059444	3,440163461
positive regulation of iron ion transport	1	0,010181403	0,032059444	3,440163461
regulation of iron ion import	1	0,010181403	0,032059444	3,440163461
regulation of ferrous iron import into cell	1	0,010181403	0,032059444	3,440163461
negative regulation of antigen processing and presentation of peptide antigen via MHC class I	1	0,010181403	0,032059444	3,440163461

regulation of ferrous iron binding	1	0,010181403	0,032059444	3,440163461
regulation of transferrin receptor binding	1	0,010181403	0,032059444	3,440163461
lipoprotein particle mediated signaling	1	0,010181403	0,032059444	3,440163461
response to monosodium glutamate	1	0,010181403	0,032059444	3,440163461
connective tissue growth factor production	1	0,010181403	0,032059444	3,440163461
regulation of lung ciliated cell differentiation	1	0,010181403	0,032059444	3,440163461
regulation of complement-dependent cytotoxicity	1	0,010181403	0,032059444	3,440163461
pancreatic stellate cell proliferation	1	0,010181403	0,032059444	3,440163461
regulation of pancreatic stellate cell proliferation	1	0,010181403	0,032059444	3,440163461
cellular organohalogen metabolic process	1	0,010181403	0,032059444	3,440163461
cellular organofluorine metabolic process	1	0,010181403	0,032059444	3,440163461
stress response to copper ion	1	0,010181403	0,032059444	3,440163461
enzyme active site formation	1	0,010181403	0,032059444	3,440163461
regulation of protein K48-linked deubiquitination	1	0,010181403	0,032059444	3,440163461
regulation of TRAIL-activated apoptotic signaling pathway	1	0,010181403	0,032059444	3,440163461
regulation of cellular amino acid biosynthetic process	1	0,010181403	0,032059444	3,440163461
neuron intrinsic apoptotic signaling pathway in response to hydrogen peroxide	1	0,010181403	0,032059444	3,440163461
regulation of hydrogen peroxide-induced neuron intrinsic apoptotic signaling pathway	1	0,010181403	0,032059444	3,440163461
p38MAPK cascade	2	0,010206034	0,032059444	3,440163461
regulation of glycogen biosynthetic process	2	0,010206034	0,032059444	3,440163461
B cell homeostasis	2	0,010206034	0,032059444	3,440163461
response to epidermal growth factor	2	0,010206034	0,032059444	3,440163461
regulation of glucan biosynthetic process	2	0,010206034	0,032059444	3,440163461
muscle cell differentiation	6	0,010228286	0,032103135	3,438801605
nucleic acid phosphodiester bond hydrolysis	5	0,010301046	0,03228754	3,433073879
T cell activation involved in immune response	3	0,010307819	0,03228754	3,433073879
response to heat	4	0,010312211	0,03228754	3,433073879
cell fate commitment	5	0,01062591	0,033215679	3,404733255
cell junction organization	5	0,01062591	0,033215679	3,404733255
positive regulation of catalytic activity	15	0,010656933	0,033285614	3,402629978
homeostasis of number of cells within a tissue	2	0,01087403	0,033853777	3,385704703
DNA catabolic process	2	0,01087403	0,033853777	3,385704703
apoptotic process involved in development	2	0,01087403	0,033853777	3,385704703
mitochondrial fission	2	0,01087403	0,033853777	3,385704703
cell aging	3	0,010955333	0,034024311	3,380679992
regulation of DNA binding	3	0,010955333	0,034024311	3,380679992
ammonium transport	3	0,010955333	0,034024311	3,380679992
exocytosis	6	0,011312588	0,035105514	3,349397078
epithelial cell differentiation	8	0,011341192	0,035165921	3,347677831
regulated exocytosis	4	0,011404587	0,035334017	3,342909129
defense response	17	0,011457806	0,035470345	3,339058292
negative regulation of extrinsic apoptotic signaling pathway in absence of ligand	2	0,011560847	0,035560462	3,336520883
response to X-ray	2	0,011560847	0,035560462	3,336520883

maternal placenta development	2	0,011560847	0,035560462	3,336520883
multicellular organism aging	2	0,011560847	0,035560462	3,336520883
mRNA stabilization	2	0,011560847	0,035560462	3,336520883
positive regulation of epidermis development	2	0,011560847	0,035560462	3,336520883
negative regulation of signal transduction in absence of ligand	2	0,011560847	0,035560462	3,336520883
histone phosphorylation	2	0,011560847	0,035560462	3,336520883
positive regulation of ERK1 and ERK2 cascade	4	0,011631654	0,035749685	3,331213823
negative regulation of gene expression	15	0,011782505	0,036184421	3,319126604
response to cytokine	10	0,011939589	0,03663759	3,306680523
membrane depolarization	3	0,011969919	0,036642999	3,306532883
regulation of protein complex disassembly	3	0,011969919	0,036642999	3,306532883
regulation of calcium ion transport into cytosol	3	0,011969919	0,036642999	3,306532883
plasma membrane organization	5	0,011995734	0,036692833	3,305173839
regulation of carbohydrate metabolic process	4	0,012094488	0,03696552	3,297769686
neuronal action potential	2	0,012266278	0,037195145	3,291577038
response to amphetamine	2	0,012266278	0,037195145	3,291577038
negative regulation of JNK cascade	2	0,012266278	0,037195145	3,291577038
synaptic vesicle recycling	2	0,012266278	0,037195145	3,291577038
regulation of transcription from RNA polymerase II promoter in response to hypoxia	2	0,012266278	0,037195145	3,291577038
RNA stabilization	2	0,012266278	0,037195145	3,291577038
Schwann cell differentiation	2	0,012266278	0,037195145	3,291577038
response to mineralocorticoid	2	0,012266278	0,037195145	3,291577038
dopamine metabolic process	2	0,012266278	0,037195145	3,291577038
dopamine transport	2	0,012266278	0,037195145	3,291577038
RNA metabolic process	33	0,012284876	0,037222208	3,290849718
cellular response to nutrient levels	4	0,01233028	0,037330383	3,287947724
nucleobase-containing compound metabolic process	39	0,012375684	0,037438391	3,285058616
cell growth	7	0,012425343	0,037559089	3,281839875
response to ammonium ion	3	0,012675381	0,038284826	3,262701656
regulation of protein import into nucleus	4	0,012810676	0,038663124	3,252869001
regulation of cell activation	7	0,012947656	0,03902107	3,243653529
positive regulation of dendritic spine development	2	0,012990122	0,03902107	3,243653529
negative regulation of cation channel activity	2	0,012990122	0,03902107	3,243653529
regulation of myelination	2	0,012990122	0,03902107	3,243653529
regulation of transcription regulatory region DNA binding	2	0,012990122	0,03902107	3,243653529
negative regulation of epithelial cell apoptotic process	2	0,012990122	0,03902107	3,243653529
DNA-templated transcription, termination	3	0,013036879	0,039100477	3,241620615
erythrocyte differentiation	3	0,013036879	0,039100477	3,241620615
T cell proliferation	4	0,013055306	0,039125247	3,240987304
embryo development	11	0,013091469	0,039203091	3,23899968
negative regulation of multicellular organismal process	11	0,013181379	0,039441638	3,232933223
cellular response to acid chemical	4	0,013302906	0,039743465	3,225309857
negative regulation of homeostatic process	4	0,013302906	0,039743465	3,225309857

skeletal system development	7	0,013349685	0,03985228	3,222575661
positive regulation of cation transmembrane transport	3	0,01340424	0,039984122	3,219272853
necroptotic process	2	0,013732177	0,040835715	3,198198205
positive regulation of glucose metabolic process	2	0,013732177	0,040835715	3,198198205
response to iron ion	2	0,013732177	0,040835715	3,198198205
regulation of protein oligomerization	2	0,013732177	0,040835715	3,198198205
chondrocyte differentiation	3	0,013777478	0,040844172	3,197991138
ovulation cycle process	3	0,013777478	0,040844172	3,197991138
regulation of regulated secretory pathway	3	0,013777478	0,040844172	3,197991138
phenol-containing compound metabolic process	3	0,013777478	0,040844172	3,197991138
regulation of protein import	4	0,013807065	0,040900375	3,196616039
negative regulation of transcription from RNA polymerase II promoter	9	0,014001302	0,041443855	3,183415658
mitochondrial transport	5	0,014069149	0,041612673	3,17935053
alcohol metabolic process	6	0,01421062	0,041998821	3,17011373
organic substance metabolic process	64	0,014370929	0,042440011	3,159663711
purine nucleotide metabolic process	7	0,014465757	0,042667287	3,154322755
salivary gland morphogenesis	2	0,014492241	0,042667287	3,154322755
regulation of glycogen metabolic process	2	0,014492241	0,042667287	3,154322755
regulation of receptor internalization	2	0,014492241	0,042667287	3,154322755
response to glucose	4	0,014585877	0,042850127	3,150046666
cellular metabolic process	62	0,014814813	0,042850127	3,150046666
regulation of organ morphogenesis	4	0,014851546	0,042850127	3,150046666
calcium ion regulated exocytosis	3	0,014932569	0,042850127	3,150046666
leukocyte apoptotic process	3	0,014932569	0,042850127	3,150046666
myoblast differentiation involved in skeletal muscle regeneration	1	0,015233621	0,042850127	3,150046666
positive regulation of interleukin-12 secretion	1	0,015233621	0,042850127	3,150046666
blastocyst hatching	1	0,015233621	0,042850127	3,150046666
positive regulation of vitamin D biosynthetic process	1	0,015233621	0,042850127	3,150046666
positive regulation of calcidiol 1-monooxygenase activity	1	0,015233621	0,042850127	3,150046666
positive regulation of mononuclear cell migration	1	0,015233621	0,042850127	3,150046666
response to antipsychotic drug	1	0,015233621	0,042850127	3,150046666
cellular response to cell-matrix adhesion	1	0,015233621	0,042850127	3,150046666
negative regulation of helicase activity	1	0,015233621	0,042850127	3,150046666
oligodendrocyte apoptotic process	1	0,015233621	0,042850127	3,150046666
positive regulation of endodeoxyribonuclease activity	1	0,015233621	0,042850127	3,150046666
response to insulin-like growth factor stimulus	1	0,015233621	0,042850127	3,150046666
receptor internalization involved in canonical Wnt signaling pathway	1	0,015233621	0,042850127	3,150046666
response to vitamin B1	1	0,015233621	0,042850127	3,150046666
response to platinum ion	1	0,015233621	0,042850127	3,150046666
positive regulation of integrin biosynthetic process	1	0,015233621	0,042850127	3,150046666
regulation of growth rate	1	0,015233621	0,042850127	3,150046666
protein poly-ADP-ribosylation	1	0,015233621	0,042850127	3,150046666
positive regulation of vitamin D 24-hydroxylase activity	1	0,015233621	0,042850127	3,150046666

bile acid signaling pathway	1	0,015233621	0,042850127	3,150046666
positive regulation of B cell apoptotic process	1	0,015233621	0,042850127	3,150046666
regulation of nitrogen utilization	1	0,015233621	0,042850127	3,150046666
apoptotic process involved in embryonic digit morphogenesis	1	0,015233621	0,042850127	3,150046666
regulation of mitochondrial membrane permeability involved in programmed necrotic cell death	1	0,015233621	0,042850127	3,150046666
response to acetate	1	0,015233621	0,042850127	3,150046666
ovarian follicle atresia	1	0,015233621	0,042850127	3,150046666
peripheral nervous system neuron axonogenesis	1	0,015233621	0,042850127	3,150046666
negative regulation of NAD(P)H oxidase activity	1	0,015233621	0,042850127	3,150046666
alkaloid catabolic process	1	0,015233621	0,042850127	3,150046666
isoquinoline alkaloid metabolic process	1	0,015233621	0,042850127	3,150046666
regulation of fibril organization	1	0,015233621	0,042850127	3,150046666
hatching	1	0,015233621	0,042850127	3,150046666
organism emergence from protective structure	1	0,015233621	0,042850127	3,150046666
regulation of chronic inflammatory response to antigenic stimulus	1	0,015233621	0,042850127	3,150046666
regulation of translational initiation by iron	1	0,015233621	0,042850127	3,150046666
positive regulation of vitamin metabolic process	1	0,015233621	0,042850127	3,150046666
regulation of dense core granule biogenesis	1	0,015233621	0,042850127	3,150046666
nitric oxide homeostasis	1	0,015233621	0,042850127	3,150046666
regulation of glycolytic process by regulation of transcription from RNA polymerase II promoter	1	0,015233621	0,042850127	3,150046666
regulation of macromitophagy	1	0,015233621	0,042850127	3,150046666
negative regulation of fermentation	1	0,015233621	0,042850127	3,150046666
regulation of NAD metabolic process	1	0,015233621	0,042850127	3,150046666
regulation of glucose catabolic process to lactate via pyruvate	1	0,015233621	0,042850127	3,150046666
regulation of vitamin D 24-hydroxylase activity	1	0,015233621	0,042850127	3,150046666
age-dependent response to oxidative stress	1	0,015233621	0,042850127	3,150046666
age-dependent general metabolic decline	1	0,015233621	0,042850127	3,150046666
regulation of iron ion transport	1	0,015233621	0,042850127	3,150046666
tyrosine phosphorylation of Stat6 protein	1	0,015233621	0,042850127	3,150046666
regulation of tyrosine phosphorylation of Stat6 protein	1	0,015233621	0,042850127	3,150046666
lung ciliated cell differentiation	1	0,015233621	0,042850127	3,150046666
complement-dependent cytotoxicity	1	0,015233621	0,042850127	3,150046666
detoxification of inorganic compound	1	0,015233621	0,042850127	3,150046666
stress response to metal ion	1	0,015233621	0,042850127	3,150046666
peptidyl-cysteine oxidation	1	0,015233621	0,042850127	3,150046666
glycolate metabolic process	1	0,015233621	0,042850127	3,150046666
regulation of oxidative phosphorylation uncoupler activity	1	0,015233621	0,042850127	3,150046666
insulin-like growth factor receptor signaling pathway	2	0,015270118	0,042921412	3,148384462
memory	3	0,01532943	0,043056676	3,145237979
regulation of inflammatory response	5	0,015507434	0,043524876	3,134422652
proteolysis involved in cellular protein catabolic process	8	0,015523727	0,04353885	3,134101625
sex differentiation	5	0,015720641	0,043995343	3,123671492
intrinsic apoptotic signaling pathway in response to DNA damage	3	0,015732222	0,043995343	3,123671492

erythrocyte homeostasis	3	0,015732222	0,043995343	3,123671492
regulation of production of molecular mediator of immune response	3	0,015732222	0,043995343	3,123671492
establishment of protein localization to mitochondrion	4	0,015944841	0,044525216	3,111699589
response to hexose	4	0,015944841	0,044525216	3,111699589
response to testosterone	2	0,016065609	0,044667964	3,108498718
keratinocyte proliferation	2	0,016065609	0,044667964	3,108498718
positive regulation of macroautophagy	2	0,016065609	0,044667964	3,108498718
DNA strand elongation involved in DNA replication	2	0,016065609	0,044667964	3,108498718
regulation of polysaccharide biosynthetic process	2	0,016065609	0,044667964	3,108498718
regulation of activated T cell proliferation	2	0,016065609	0,044667964	3,108498718
regulation of striated muscle tissue development	3	0,016140956	0,044845053	3,104541991
maintenance of location	5	0,016152934	0,044845953	3,104521939
JNK cascade	4	0,016225872	0,045015975	3,100737855
cytokine production	8	0,016619628	0,046075166	3,077481177
membrane protein ectodomain proteolysis	2	0,016878519	0,046691977	3,064182926
axon regeneration	2	0,016878519	0,046691977	3,064182926
negative regulation of endocytosis	2	0,016878519	0,046691977	3,064182926
regulation of muscle organ development	3	0,01697628	0,046861353	3,060561977
regulation of muscle tissue development	3	0,01697628	0,046861353	3,060561977
phosphatidylinositol 3-kinase signaling	3	0,01697628	0,046861353	3,060561977
regulation of lymphocyte activation	6	0,01702523	0,046939623	3,058893115
positive regulation of response to external stimulus	5	0,01704123	0,046939623	3,058893115
regulation of T cell activation	5	0,01704123	0,046939623	3,058893115
protein localization to mitochondrion	4	0,01708763	0,047033762	3,0568896
cellular respiration	4	0,017381136	0,047807442	3,040573959
NADH metabolic process	2	0,017708654	0,048500284	3,026185625
positive regulation of release of sequestered calcium ion into cytosol	2	0,017708654	0,048500284	3,026185625
temperature homeostasis	2	0,017708654	0,048500284	3,026185625
salivary gland development	2	0,017708654	0,048500284	3,026185625
developmental programmed cell death	2	0,017708654	0,048500284	3,026185625
phenol-containing compound biosynthetic process	2	0,017708654	0,048500284	3,026185625
negative regulation of transcription, DNA-templated	12	0,01778777	0,048682318	3,022439399
post-embryonic development	3	0,017835463	0,048778154	3,020472737
myelination	3	0,018274018	0,049942061	2,996891723
