# Supplementary material for: Molecular Taxonomy of Sporadic Amyotrophic Lateral Sclerosis Using Disease-Associated Genes
Source: Front Neurol. 2017 Apr 19;8:152. doi: 10.3389/fneur.2017.00152 (PMC5395696; doi:10.3389/fneur.2017.00152)
Supplement: Supplementary file 6 [file Table_6.PDF]

Supplementary Table 6. List of the significantly enriched GO biological processes for differentially expressed SGALS genes in SALS1/SALS2 patients

GO biological process	SGALS genes in category	p value	fdr p value	-LOG(corr.pValue)
regulation of cell death	44	2.05014E-18	8.45683E-15	32.4038016
cell death	49	5.17E-18	1.07E-14	32.17164484
regulation of programmed cell death	41	5.75E-17	7.91E-14	30.16822791
programmed cell death	46	1.10E-16	1.13E-13	29.80991698
regulation of apoptotic process	40	2.76E-16	2.27E-13	29.11245876
response to oxidative stress	23	6.21E-16	4.27E-13	28.48239607
apoptotic process	44	2.36E-15	1.39E-12	27.30080575
negative regulation of programmed cell death	29	5.80E-14	2.74E-11	24.32210963
negative regulation of cell death	30	5.97E-14	2.74E-11	24.32210963
regulation of neuron death	18	7.56E-14	3.12E-11	24.1913334
negative regulation of apoptotic process	28	2.91E-13	1.09E-10	22.93986155
regulation of cellular response to oxidative stress	11	3.21E-13	1.10E-10	22.92724907
apoptotic signaling pathway	24	4.57E-13	1.45E-10	22.65515919
regulation of apoptotic signaling pathway	20	5.23E-13	1.54E-10	22.59273671
neuron death	18	5.96E-13	1.64E-10	22.53127374
regulation of response to oxidative stress	11	7.89E-13	2.03E-10	22.31554423
response to endogenous stimulus	37	1.30E-12	3.16E-10	21.87500247
regulation of oxidative stress-induced cell death	10	1.70E-12	3.80E-10	21.69010459
cellular response to oxidative stress	16	1.75E-12	3.80E-10	21.69010459
positive regulation of cellular process	66	4.60E-12	9.48E-10	20.77661096
regulation of localization	46	5.29E-12	1.04E-09	20.68528099
cellular response to chemical stimulus	48	5.74E-12	1.08E-09	20.64911906
regulation of transport	38	1.79E-11	3.20E-09	19.55861712
cell death in response to oxidative stress	10	2.45E-11	4.04E-09	19.32648012
response to inorganic substance	20	2.65E-11	4.04E-09	19.32648012
response to oxygen-containing compound	34	2.70E-11	4.04E-09	19.32648012
positive regulation of response to stimulus	40	2.74E-11	4.04E-09	19.32648012
positive regulation of cell communication	35	2.74E-11	4.04E-09	19.32648012
positive regulation of signaling	35	3.13E-11	4.45E-09	19.23079245
regulation of cell communication	50	3.90E-11	5.36E-09	19.04503346
response to organic substance	48	4.79E-11	6.37E-09	18.87122207
positive regulation of cell death	22	5.28E-11	6.81E-09	18.80521152
positive regulation of biological process	68	6.47E-11	8.09E-09	18.63282811
regulation of signaling	50	7.40E-11	8.98E-09	18.52850162
intracellular signal transduction	46	9.39E-11	1.11E-08	18.31922507
response to reactive oxygen species	14	1.09E-10	1.25E-08	18.19857899
system development	60	1.33E-10	1.48E-08	18.02663791
response to organonitrogen compound	24	1.89E-10	2.05E-08	17.70192271
response to oxygen levels	16	1.96E-10	2.08E-08	17.68959659
phosphorylation	41	2.85E-10	2.88E-08	17.36440333
regulation of biological quality	53	2.86E-10	2.88E-08	17.36440333
response to chemical	58	3.23E-10	3.17E-08	17.26669134

positive regulation of multicellular organismal process	32	3.62E-10	3.45E-08	17.18192492
regulation of cellular component organization	41	3.68E-10	3.45E-08	17.18192492
protein phosphorylation	37	3.82E-10	3.50E-08	17.16841782
single-organism localization	48	4.42E-10	3.97E-08	17.0430149
negative regulation of cellular process	59	4.64E-10	4.04E-08	17.02538125
response to corticosteroid	12	4.77E-10	4.04E-08	17.02538125
response to nitrogen compound	25	4.79E-10	4.04E-08	17.02538125
regulation of intracellular signal transduction	35	5.16E-10	4.25E-08	16.97262789
response to organic cyclic compound	25	5.54E-10	4.48E-08	16.9211902
response to hypoxia	15	5.86E-10	4.65E-08	16.88363149
response to amino acid	10	6.89E-10	5.36E-08	16.74117651
neurofilament cytoskeleton organization	5	7.21E-10	5.51E-08	16.71463766
positive regulation of cellular metabolic process	46	7.97E-10	5.98E-08	16.63228657
response to decreased oxygen levels	15	8.69E-10	6.40E-08	16.56427701
regulation of phosphorylation	31	8.94E-10	6.47E-08	16.55337133
multicellular organism development	63	9.43E-10	6.61E-08	16.53208585
negative regulation of biological process	61	9.45E-10	6.61E-08	16.53208585
positive regulation of cellular component organization	28	1.04E-09	7.17E-08	16.45102228
regulation of cellular response to stress	20	1.13E-09	7.51E-08	16.40466832
transport	61	1.18E-09	7.51E-08	16.40466832
phosphate-containing compound metabolic process	48	1.19E-09	7.51E-08	16.40466832
response to stress	53	1.19E-09	7.51E-08	16.40466832
positive regulation of signal transduction	31	1.23E-09	7.51E-08	16.40466832
negative regulation of apoptotic signaling pathway	13	1.23E-09	7.51E-08	16.40466832
localization	70	1.24E-09	7.51E-08	16.40466832
response to lipid	24	1.24E-09	7.51E-08	16.40466832
phosphorus metabolic process	48	1.27E-09	7.60E-08	16.39213567
positive regulation of phosphorylation	25	1.37E-09	8.09E-08	16.33007772
regulation of cellular protein metabolic process	41	1.48E-09	8.57E-08	16.2723513
negative regulation of neuron death	12	1.73E-09	9.93E-08	16.12506118
cellular component organization	71	1.88E-09	1.06E-07	16.05867028
response to glucocorticoid	11	2.19E-09	1.22E-07	15.91794219
positive regulation of phosphorus metabolic process	26	3.21E-09	1.74E-07	15.56393058
positive regulation of phosphate metabolic process	26	3.21E-09	1.74E-07	15.56393058
extrinsic apoptotic signaling pathway	13	3.27E-09	1.75E-07	15.55637609
single-multicellular organism process	70	3.35E-09	1.77E-07	15.54678461
regulation of protein phosphorylation	29	3.45E-09	1.80E-07	15.52810065
establishment of localization	61	3.78E-09	1.92E-07	15.46366105
response to steroid hormone	16	3.78E-09	1.92E-07	15.46366105
multicellular organismal process	77	4.36E-09	2.19E-07	15.33319139
regulation of intrinsic apoptotic signaling pathway	11	4.63E-09	2.30E-07	15.28538856
positive regulation of cell differentiation	23	4.84E-09	2.38E-07	15.25230809
single-organism transport	44	4.97E-09	2.41E-07	15.23845562
positive regulation of apoptotic process	19	5.20E-09	2.49E-07	15.20475764

nervous system development	38	5.32E-09	2.5225E-07	15.19284692
regulation of neuron apoptotic process	12	5.45E-09	2.55382E-07	15.18050691
positive regulation of programmed cell death	19	6.02E-09	2.78603E-07	15.09347669
positive regulation of protein metabolic process	31	6.08E-09	2.78603E-07	15.09347669
positive regulation of cellular protein metabolic process	30	6.28E-09	2.84481E-07	15.07260003
cellular component organization or biogenesis	71	6.43E-09	2.88525E-07	15.05848428
positive regulation of neuron death	8	7.57E-09	3.35861E-07	14.9065695
response to hydrogen peroxide	10	7.84E-09	3.41201E-07	14.89079295
positive regulation of metabolic process	46	7.86E-09	3.41201E-07	14.89079295
regulation of signal transduction	43	8.97E-09	3.85263E-07	14.76934083
regulation of phosphate metabolic process	32	9.16E-09	3.87716E-07	14.76299397
regulation of cell differentiation	31	9.22E-09	3.87716E-07	14.76299397
regulation of phosphorus metabolic process	32	9.31E-09	3.87716E-07	14.76299397
positive regulation of macromolecule metabolic process	44	1.06E-08	4.36049E-07	14.6455107
regulation of protein metabolic process	41	1.13E-08	4.59492E-07	14.59314327
regulation of multicellular organismal process	42	1.34E-08	5.40315E-07	14.43111371
developmental process	68	1.45E-08	5.75948E-07	14.36724917
anatomical structure development	65	1.45E-08	5.75948E-07	14.36724917
transmembrane receptor protein tyrosine kinase signaling pathway	20	1.48E-08	5.81699E-07	14.35731261
positive regulation of protein phosphorylation	23	1.51E-08	5.86525E-07	14.34905116
cellular response to reactive oxygen species	10	1.59E-08	6.09131E-07	14.31123174
enzyme linked receptor protein signaling pathway	24	1.60E-08	6.09131E-07	14.31123174
cellular response to growth factor stimulus	19	1.61E-08	6.09131E-07	14.31123174
neuron apoptotic process	12	1.79E-08	6.70987E-07	14.21451597
single-organism developmental process	67	1.88E-08	6.98173E-07	14.17479866
regulation of protein modification process	32	1.91E-08	7.04249E-07	14.1661336
cellular response to stress	33	2.19E-08	7.98389E-07	14.04066994
regulation of response to stimulus	50	2.26E-08	8.1607E-07	14.01876596
response to extracellular stimulus	16	2.35E-08	8.42992E-07	13.98630798
cellular response to oxygen-containing compound	23	2.60E-08	9.26014E-07	13.89237693
single organism signaling	70	2.71E-08	9.55594E-07	13.86093249
signaling	70	2.86E-08	9.99756E-07	13.81575431
cellular response to organic substance	37	2.95E-08	1.023E-06	13.79277309
generation of neurons	28	3.05E-08	1.04924E-06	13.7674469
response to growth factor	19	3.11E-08	1.05493E-06	13.76203216
cell differentiation	50	3.13E-08	1.05493E-06	13.76203216
neurogenesis	29	3.15E-08	1.05493E-06	13.76203216
aging	13	3.29E-08	1.09339E-06	13.72622788
response to abiotic stimulus	25	3.45E-08	1.13904E-06	13.68532258
homeostatic process	31	3.90E-08	1.26731E-06	13.57861434
positive regulation of developmental process	26	3.90E-08	1.26731E-06	13.57861434
intrinsic apoptotic signaling pathway	13	4.27E-08	1.37465E-06	13.49731263
regulation of oxidative stress-induced intrinsic apoptotic signaling pathway	6	4.41E-08	1.41029E-06	13.47171587
reactive oxygen species metabolic process	12	4.93E-08	1.56283E-06	13.36901082

MAPK cascade	22	5.07E-08	1.59512E-06	13.34856091
positive regulation of nucleobase-containing compound metabolic process	31	5.59E-08	1.7462E-06	13.25806705
response to nutrient levels	15	5.91E-08	1.834E-06	13.20901075
positive regulation of nitrogen compound metabolic process	32	6.56E-08	2.01889E-06	13.11296185
positive regulation of cellular response to oxidative stress	5	7.59E-08	2.31846E-06	12.97460786
positive regulation of intracellular signal transduction	22	7.78E-08	2.35856E-06	12.95745877
positive regulation of protein modification process	25	8.00E-08	2.40826E-06	12.93660416
positive regulation of reactive oxygen species metabolic process	8	8.96E-08	2.67964E-06	12.82982856
regulation of extrinsic apoptotic signaling pathway	10	9.28E-08	2.74582E-06	12.80543011
cell communication	69	9.32E-08	2.74582E-06	12.80543011
positive regulation of response to oxidative stress	5	1.04E-07	3.04313E-06	12.70262408
signal transduction by protein phosphorylation	22	1.05E-07	3.04313E-06	12.70262408
peptidyl-serine phosphorylation	12	1.13E-07	3.25258E-06	12.63606141
regulation of cell proliferation	29	1.21E-07	3.45887E-06	12.57456909
response to toxic substance	11	1.24E-07	3.54136E-06	12.55100087
response to hormone	21	1.28E-07	3.6101E-06	12.53177456
regulation of multicellular organismal development	31	1.30E-07	3.6101E-06	12.53177456
positive regulation of transcription, DNA-templated	27	1.30E-07	3.6101E-06	12.53177456
positive regulation of nucleic acid-templated transcription	27	1.30E-07	3.6101E-06	12.53177456
regulation of response to stress	26	1.71E-07	4.70997E-06	12.26582902
positive regulation of RNA biosynthetic process	27	1.80E-07	4.88152E-06	12.23005358
negative regulation of response to stimulus	27	1.80E-07	4.88152E-06	12.23005358
peptidyl-serine modification	12	1.97E-07	5.27106E-06	12.15327874
single-organism cellular localization	24	1.97E-07	5.27106E-06	12.15327874
cell proliferation	33	2.09E-07	5.57506E-06	12.09720671
tissue development	31	2.15E-07	5.66154E-06	12.081814
positive regulation of biosynthetic process	31	2.15E-07	5.66154E-06	12.081814
positive regulation of transcription from RNA polymerase II promoter	23	2.29E-07	5.98078E-06	12.02695965
response to acid chemical	12	2.32E-07	6.01363E-06	12.02148199
regulation of cysteine-type endopeptidase activity	11	2.45E-07	6.31212E-06	11.97303844
cell development	32	2.86E-07	7.33291E-06	11.82313843
neurofilament bundle assembly	3	3.03E-07	7.72046E-06	11.77163603
positive regulation of macromolecule biosynthetic process	29	3.13E-07	7.88447E-06	11.7506158
chemical homeostasis	23	3.13E-07	7.88447E-06	11.7506158
positive regulation of RNA metabolic process	27	3.50E-07	8.75349E-06	11.64605855
negative regulation of metabolic process	38	3.54E-07	8.79314E-06	11.64153832
regulation of cellular localization	20	3.92E-07	9.67818E-06	11.54563642
regulation of MAPK cascade	18	4.15E-07	1.00862E-05	11.50433804
negative regulation of extrinsic apoptotic signaling pathway	8	4.15E-07	1.00862E-05	11.50433804
positive regulation of smooth muscle cell proliferation	7	4.16E-07	1.00862E-05	11.50433804
epithelial cell proliferation	13	4.50E-07	1.08636E-05	11.43009665
positive regulation of transport	21	4.78E-07	1.14744E-05	11.37539317
positive regulation of cellular biosynthetic process	30	5.04E-07	1.20224E-05	11.32874017
cell surface receptor signaling pathway	39	5.10E-07	1.20609E-05	11.32554023

secretion	23	5.12E-07	1.20609E-05	11.32554023
intrinsic apoptotic signaling pathway in response to oxidative stress	6	5.57E-07	1.30552E-05	11.24632773
cellular developmental process	50	5.99E-07	1.39575E-05	11.17949466
negative regulation of signal transduction	23	6.23E-07	1.44461E-05	11.14508939
regulation of transcription from RNA polymerase II promoter	31	6.29E-07	1.44999E-05	11.14136729
regulation of hydrogen peroxide-mediated programmed cell death	4	6.36E-07	1.45863E-05	11.13542797
regulation of reactive oxygen species metabolic process	9	6.90E-07	1.57282E-05	11.0600577
regulation of smooth muscle cell proliferation	8	6.95E-07	1.5762E-05	11.05791167
regulation of cysteine-type endopeptidase activity involved in apoptotic process	10	7.21E-07	1.61544E-05	11.03331977
single-organism cellular process	101	7.26E-07	1.61544E-05	11.03331977
cellular response to oxygen levels	9	7.31E-07	1.61544E-05	11.03331977
negative regulation of cell communication	24	7.31E-07	1.61544E-05	11.03331977
divalent metal ion transport	14	7.32E-07	1.61544E-05	11.03331977
negative regulation of signaling	24	7.78E-07	1.70686E-05	10.9782683
divalent inorganic cation transport	14	7.99E-07	1.73763E-05	10.96040423
smooth muscle cell proliferation	8	8.00E-07	1.73763E-05	10.96040423
cellular response to hydrogen peroxide	7	8.10E-07	1.74956E-05	10.95356087
regulation of proteolysis	18	8.27E-07	1.77666E-05	10.93819082
response to lipopolysaccharide	12	8.43E-07	1.80216E-05	10.92393714
regulation of developmental process	35	8.96E-07	1.90538E-05	10.86824405
secretion by cell	21	9.44E-07	1.99751E-05	10.82102489
peptidyl-amino acid modification	24	9.77E-07	2.05705E-05	10.79165052
positive regulation of gene expression	29	1.00E-06	2.09518E-05	10.77328519
neuron differentiation	24	1.02E-06	2.13037E-05	10.75662845
regulation of endopeptidase activity	13	1.08E-06	2.22905E-05	10.71134886
signal transduction	63	1.19E-06	2.45434E-05	10.61506895
negative regulation of transport	14	1.25E-06	2.56828E-05	10.56968781
muscle cell proliferation	9	1.26E-06	2.5815E-05	10.56455612
cellular response to stimulus	71	1.32E-06	2.67011E-05	10.53080748
response to molecule of bacterial origin	12	1.32E-06	2.67011E-05	10.53080748
positive chemotaxis	6	1.34E-06	2.69565E-05	10.52128662
hydrogen peroxide-mediated programmed cell death	4	1.36E-06	2.70976E-05	10.51606439
programmed cell death in response to reactive oxygen species	4	1.36E-06	2.70976E-05	10.51606439
regulation of peptidyl-serine phosphorylation	8	1.37E-06	2.70976E-05	10.51606439
regulation of mitochondrial membrane potential	6	1.50E-06	2.96524E-05	10.42596802
negative regulation of cellular metabolic process	35	1.58E-06	3.09836E-05	10.3820516
regulation of autophagy	11	1.59E-06	3.11491E-05	10.37672595
transcription from RNA polymerase II promoter	32	1.84E-06	3.57435E-05	10.23914166
single-organism metabolic process	50	1.99E-06	3.84704E-05	10.1656202
response to external stimulus	32	2.05E-06	3.95013E-05	10.13917688
negative regulation of molecular function	22	2.07E-06	3.97788E-05	10.1321766
regulation of peptidase activity	13	2.13E-06	4.06222E-05	10.111196
regulation of organelle organization	21	2.19E-06	4.156E-05	10.08837307
cellular localization	36	2.26E-06	4.28358E-05	10.05813653

release of cytochrome c from mitochondria	6	2.32E-06	4.37571E-05	10.03685715
regulation of mitochondrion organization	10	2.48E-06	4.64701E-05	9.976702539
positive regulation of oxidative stress-induced cell death	4	2.58E-06	4.79627E-05	9.945086639
positive regulation of neuron projection development	10	2.58E-06	4.79627E-05	9.945086639
response to drug	13	2.90E-06	5.3593E-05	9.834091879
regulation of neuron projection development	13	2.98E-06	5.48452E-05	9.810995278
negative regulation of oxidative stress-induced cell death	5	3.11E-06	5.65602E-05	9.780205032
negative regulation of cellular response to oxidative stress	5	3.11E-06	5.65602E-05	9.780205032
negative regulation of response to oxidative stress	5	3.11E-06	5.65602E-05	9.780205032
response to axon injury	6	3.15E-06	5.70363E-05	9.771823046
cellular response to hypoxia	8	3.36E-06	6.06076E-05	9.711090121
female pregnancy	9	3.39E-06	6.0608E-05	9.711083676
movement of cell or subcellular component	29	3.39E-06	6.0608E-05	9.711083676
negative regulation of neuron apoptotic process	8	3.56E-06	6.32668E-05	9.668149074
regulation of response to reactive oxygen species	5	3.61E-06	6.39416E-05	9.657540522
negative regulation of macromolecule metabolic process	34	3.81E-06	6.63597E-05	9.620420986
cellular protein modification process	47	3.82E-06	6.63597E-05	9.620420986
protein modification process	47	3.82E-06	6.63597E-05	9.620420986
regulation of glucose import	6	3.83E-06	6.63597E-05	9.620420986
regulation of muscle adaptation	6	3.83E-06	6.63597E-05	9.620420986
regulation of cell development	18	3.93E-06	6.77908E-05	9.599083736
macrophage differentiation	5	4.17E-06	7.1701E-05	9.543006395
cell morphogenesis involved in differentiation	16	4.20E-06	7.19603E-05	9.539396602
neuron development	20	4.27E-06	7.27076E-05	9.529064257
regulation of binding	11	4.41E-06	7.47766E-05	9.501005857
organelle organization	42	4.66E-06	7.87054E-05	9.449798332
cellular response to decreased oxygen levels	8	4.67E-06	7.87054E-05	9.449798332
anatomical structure morphogenesis	36	5.04E-06	8.45191E-05	9.378533213
cellular protein metabolic process	55	5.17E-06	8.63762E-05	9.356798398
axo-dendritic transport	5	5.49E-06	9.13942E-05	9.300328092
response to metal ion	11	5.53E-06	9.15069E-05	9.299096557
transport along microtubule	7	5.55E-06	9.15069E-05	9.299096557
cellular response to lipid	14	5.71E-06	9.37901E-05	9.274451175
regulation of cellular metabolic process	62	5.89E-06	9.62689E-05	9.248365414
regulation of glucose transport	7	5.92E-06	9.62689E-05	9.248365414
regulation of protein catabolic process	12	5.97E-06	9.62689E-05	9.248365414
negative regulation of hydrogen peroxide-mediated programmed cell death	3	5.97E-06	9.62689E-05	9.248365414
intermediate filament bundle assembly	3	5.97E-06	9.62689E-05	9.248365414
regulation of anatomical structure morphogenesis	20	6.04E-06	9.66812E-05	9.244091378
positive regulation of endothelial cell proliferation	6	6.05E-06	9.66812E-05	9.244091378
neuron projection development	18	6.34E-06	0.000100913	9.20125593
calcium ion transport	12	6.49E-06	0.000103042	9.180373426
autophagy	13	6.56E-06	0.00010367	9.174299379
cellular response to organic cyclic compound	14	6.86E-06	0.00010808	9.132640879

regulation of proteasomal ubiquitin-dependent protein catabolic process	7	7.64E-06	0.000119868	9.029116986
intermediate filament cytoskeleton organization	5	8.07E-06	0.000126124	8.978247031
positive regulation of cell proliferation	18	8.47E-06	0.000131318	8.93788972
cellular homeostasis	18	8.47E-06	0.000131318	8.93788972
cellular modified amino acid metabolic process	8	9.05E-06	0.000139751	8.875651139
multi-multicellular organism process	9	9.11E-06	0.000139751	8.875651139
intermediate filament-based process	5	9.11E-06	0.000139751	8.875651139
single-organism process	105	9.70E-06	0.000148087	8.817713163
macromolecule modification	48	9.73E-06	0.000148087	8.817713163
regulation of neurogenesis	16	9.80E-06	0.000148594	8.814291102
negative regulation of protein phosphorylation	12	1.00E-05	0.00015151	8.794857913
animal organ development	40	1.01E-05	0.00015151	8.794857913
glucose import	6	1.08E-05	0.000162022	8.727778002
positive regulation of secretion by cell	11	1.09E-05	0.000162315	8.725971981
vasculature development	15	1.12E-05	0.000166232	8.702127851
regulation of macromolecule metabolic process	61	1.15E-05	0.000170173	8.678694859
regulation of release of cytochrome c from mitochondria	5	1.15E-05	0.000170173	8.678694859
cellular component morphogenesis	22	1.18E-05	0.000173807	8.657562456
apoptotic mitochondrial changes	7	1.23E-05	0.000181186	8.615985797
negative regulation of phosphorus metabolic process	14	1.27E-05	0.000185622	8.591800619
negative regulation of phosphate metabolic process	14	1.27E-05	0.000185622	8.591800619
cardiovascular system development	15	1.28E-05	0.000186264	8.588343209
positive regulation of neuron apoptotic process	5	1.2879E-05	0.000186406	8.587580943
regulation of cell projection organization	14	1.3288E-05	0.000191654	8.559820705
regulation of protein localization	19	1.36129E-05	0.000195655	8.539156239
growth	19	1.38208E-05	0.000197954	8.527476486
cellular component assembly	35	1.45467E-05	0.000207631	8.479749356
muscle adaptation	6	1.46509E-05	0.000208396	8.476068471
myeloid cell differentiation	11	1.53324E-05	0.000217341	8.434042859
positive regulation of protein complex assembly	9	1.56196E-05	0.000220653	8.418919342
cell-cell signaling	25	1.59438E-05	0.000222023	8.412728535
removal of superoxide radicals	4	1.60395E-05	0.000222023	8.412728535
positive regulation of cardiac muscle hypertrophy	4	1.60395E-05	0.000222023	8.412728535
positive regulation of positive chemotaxis	4	1.60395E-05	0.000222023	8.412728535
positive regulation of muscle hypertrophy	4	1.60395E-05	0.000222023	8.412728535
regulation of hydrogen peroxide-induced cell death	4	1.60395E-05	0.000222023	8.412728535
regulated exocytosis	10	1.7108E-05	0.000236021	8.351587742
homeostasis of number of cells	9	1.74395E-05	0.000239793	8.335735333
regulation of metabolic process	63	1.75103E-05	0.000239967	8.335009098
embryo implantation	5	1.77605E-05	0.000242589	8.324142085
cation transport	19	1.80865E-05	0.000246228	8.309253842
positive regulation of MAPK cascade	13	1.81704E-05	0.000246556	8.307920554
regulation of growth	15	1.84945E-05	0.000249884	8.294515054
regulation of neuron differentiation	14	1.85368E-05	0.000249884	8.294515054

regulation of superoxide metabolic process	4	1.9147E-05	0.000256433	8.268641413
regulation of positive chemotaxis	4	1.9147E-05	0.000256433	8.268641413
myeloid cell homeostasis	7	1.92193E-05	0.000256569	8.268114782
negative regulation of cysteine-type endopeptidase activity involved in apoptotic process	6	1.95395E-05	0.000260001	8.254825035
signal release	12	1.99112E-05	0.000264096	8.239198925
positive regulation of protein localization to nucleus	7	2.02662E-05	0.000267943	8.224736708
ion transport	24	2.06815E-05	0.00027256	8.207652498
regulation of epithelial cell proliferation	10	2.12538E-05	0.00027921	8.183545702
cytoskeleton-dependent intracellular transport	7	2.13598E-05	0.000279711	8.181752436
response to wounding	15	2.18848E-05	0.00028527	8.162074949
biological regulation	96	2.19777E-05	0.00028527	8.162074949
metal ion transport	17	2.19917E-05	0.00028527	8.162074949
cellular response to biotic stimulus	8	2.2232E-05	0.000287483	8.154347622
reactive oxygen species biosynthetic process	6	2.24345E-05	0.000287785	8.153296239
response to vitamin	6	2.24345E-05	0.000287785	8.153296239
positive regulation of locomotion	12	2.24647E-05	0.000287785	8.153296239
cellular response to oxygen radical	4	2.26752E-05	0.000287801	8.153240644
cellular response to superoxide	4	2.26752E-05	0.000287801	8.153240644
cell death in response to hydrogen peroxide	4	2.26752E-05	0.000287801	8.153240644
positive regulation of secretion	11	2.31079E-05	0.000292393	8.137410187
regulation of primary metabolic process	60	2.39253E-05	0.000301809	8.105714599
negative regulation of phosphorylation	12	2.41301E-05	0.000303466	8.100240929
positive regulation of response to reactive oxygen species	3	2.47226E-05	0.000309972	8.079029008
negative regulation of protein modification process	14	2.50417E-05	0.000313021	8.0692412
regulation of morphogenesis of a branching structure	5	2.63823E-05	0.000328782	8.020115368
protein metabolic process	57	2.66368E-05	0.000330954	8.013531202
establishment of localization in cell	29	2.70044E-05	0.000334514	8.002830646
striated muscle cell differentiation	9	2.75636E-05	0.000340418	7.985335443
cellular response to endogenous stimulus	21	2.76628E-05	0.000340624	7.984731818
multi-organism process	32	2.822E-05	0.00034645	7.967770849
positive regulation of peptidyl-serine phosphorylation	6	2.92608E-05	0.000358163	7.934522711
regulation of catabolic process	13	2.98927E-05	0.000364815	7.91612056
catabolic process	29	3.03106E-05	0.000368823	7.905193209
macromolecular complex subunit organization	33	3.0506E-05	0.000370109	7.901713116
anterograde axonal transport	4	3.11315E-05	0.000375271	7.887861482
response to peptide	12	3.11939E-05	0.000375271	7.887861482
cellular oxidant detoxification	6	3.12044E-05	0.000375271	7.887861482
blood vessel development	14	3.22278E-05	0.000386452	7.858502675
positive regulation of cell projection organization	10	3.31264E-05	0.00039551	7.835333764
regulation of secretion	15	3.31749E-05	0.00039551	7.835333764
negative regulation of cellular component organization	14	3.34809E-05	0.000398008	7.829039272
hormone transport	10	3.50733E-05	0.000414824	7.787657417
regulation of biological process	92	3.50966E-05	0.000414824	7.787657417
cellular detoxification	6	3.54026E-05	0.000417245	7.781835849

myeloid leukocyte differentiation	8	3.60482E-05	0.000423408	7.767175344
response to superoxide	4	3.61308E-05	0.000423408	7.767175344
positive regulation of neuron differentiation	10	3.71173E-05	0.000433737	7.743072877
negative regulation of proteolysis	10	3.81771E-05	0.00044486	7.717751123
positive regulation of cell development	12	3.99936E-05	0.000463979	7.675671876
regulation of endothelial cell proliferation	6	4.00428E-05	0.000463979	7.675671876
response to stimulus	76	4.01605E-05	0.000464039	7.675541925
circulatory system development	18	4.056E-05	0.000467347	7.66843936
stress-activated MAPK cascade	9	4.09655E-05	0.000470704	7.661282066
cellular catabolic process	25	4.11236E-05	0.000471208	7.660211399
superoxide metabolic process	5	4.13744E-05	0.000472524	7.657422191
cell morphogenesis	20	4.14676E-05	0.000472524	7.657422191
response to oxygen radical	4	4.16933E-05	0.000473788	7.654750521
regulation of nervous system development	16	4.2191E-05	0.000478126	7.645635266
regulation of cellular catabolic process	11	4.26454E-05	0.000481951	7.637667528
regulation of hormone secretion	9	4.50671E-05	0.000507571	7.585873928
detoxification	6	4.51584E-05	0.000507571	7.585873928
regulation of muscle system process	8	4.52837E-05	0.000507596	7.585825566
histone H3-T6 phosphorylation	2	4.55352E-05	0.000509032	7.583000562
negative regulation of catabolic process	7	4.66292E-05	0.000519853	7.561964756
stress-activated protein kinase signaling cascade	9	4.79901E-05	0.000532514	7.537900736
positive regulation of endothelial cell chemotaxis	3	4.80836E-05	0.000532514	7.537900736
positive regulation of neurogenesis	11	4.81522E-05	0.000532514	7.537900736
negative regulation of cysteine-type endopeptidase activity	6	5.07849E-05	0.000560128	7.487345621
ion homeostasis	15	5.10203E-05	0.000561223	7.48539237
regulation of peptide hormone secretion	8	5.24769E-05	0.000574183	7.462561833
morphogenesis of a branching structure	8	5.24769E-05	0.000574183	7.462561833
regulation of proteasomal protein catabolic process	7	5.33697E-05	0.000582407	7.448340528
regulation of secretion by cell	14	5.41333E-05	0.000589182	7.436775324
cellular component disassembly	13	5.58826E-05	0.000606621	7.407606558
cellular response to external stimulus	9	5.77394E-05	0.000624834	7.378024356
regulation of molecular function	36	5.78634E-05	0.000624834	7.378024356
ion transmembrane transport	18	5.92014E-05	0.000637613	7.357779565
cellular response to lipopolysaccharide	7	6.08885E-05	0.000653842	7.332644713
muscle system process	11	6.10253E-05	0.000653842	7.332644713
regulation of cellular component movement	16	6.14898E-05	0.000657112	7.327655479
positive regulation of cell migration	11	6.24617E-05	0.000665774	7.314559774
regulation of peptide secretion	8	6.27848E-05	0.000667493	7.311981572
glucose transport	7	6.63665E-05	0.000703757	7.259076784
regulation of peptide transport	8	6.73545E-05	0.000712403	7.246866671
regulation of ion transport	13	6.76862E-05	0.000714081	7.244514368
cellular response to inorganic substance	7	6.92528E-05	0.000728745	7.224186923
cellular response to organonitrogen compound	12	6.97973E-05	0.000732605	7.218903506
cell projection organization	21	7.00718E-05	0.00073362	7.217519057

axonal transport	4	7.03478E-05	0.000734644	7.216123945
positive regulation of epithelial cell migration	6	7.11057E-05	0.000740684	7.207936122
proteolysis	25	7.16054E-05	0.000744011	7.203455374
hexose transport	7	7.2241E-05	0.000746853	7.199642437
monosaccharide transport	7	7.2241E-05	0.000746853	7.199642437
organelle transport along microtubule	5	7.25552E-05	0.000748226	7.197805543
cellular chemical homeostasis	15	7.32258E-05	0.000753258	7.19110277
response to cytokine	16	7.37857E-05	0.000757129	7.185977018
negative regulation of hydrolase activity	11	7.50376E-05	0.000766163	7.174115038
regulation of transmembrane transport	11	7.50376E-05	0.000766163	7.174115038
skeletal muscle tissue development	7	7.85338E-05	0.000795951	7.13597322
endothelial cell migration	7	7.85338E-05	0.000795951	7.13597322
cellular response to molecule of bacterial origin	7	7.85338E-05	0.000795951	7.13597322
negative regulation of cellular protein metabolic process	18	8.17984E-05	0.000823803	7.101579368
positive regulation of epithelial cell proliferation	7	8.1844E-05	0.000823803	7.101579368
hemopoiesis	15	8.1881E-05	0.000823803	7.101579368
regulation of oxidative stress-induced neuron death	3	8.2524E-05	0.000828252	7.096193293
positive regulation of cell motility	11	8.39533E-05	0.000840324	7.081723066
positive regulation of endothelial cell migration	5	8.41343E-05	0.000840324	7.081723066
regulation of establishment of protein localization	16	8.69099E-05	0.00086595	7.051683297
macromolecular complex assembly	25	9.07696E-05	0.000902228	7.010643564
endothelial cell proliferation	6	9.7447E-05	0.000966271	6.942066268
regulation of protein localization to nucleus	8	9.77242E-05	0.000966697	6.941625867
regulation of cardiac muscle hypertrophy	4	9.96939E-05	0.000983821	6.924066373
establishment of organelle localization	11	0.000100106	0.000985529	6.922331923
neuron death in response to oxidative stress	3	0.000104512	0.001021828	6.886161988
positive regulation of intracellular steroid hormone receptor signaling pathway	3	0.000104512	0.001021828	6.886161988
positive regulation of cellular component movement	11	0.000104536	0.001021828	6.886161988
negative regulation of biosynthetic process	23	0.000108279	0.001055912	6.853350445
regulation of membrane potential	10	0.000110662	0.001076601	6.833946427
positive regulation of proteasomal ubiquitin-dependent protein catabolic process	5	0.000111516	0.001082361	6.828610564
skeletal muscle organ development	7	0.000112645	0.001088196	6.823234098
response to ketone	7	0.000112645	0.001088196	6.823234098
muscle structure development	13	0.000112982	0.001088901	6.82258605
muscle cell differentiation	10	0.000116082	0.001116173	6.797849549
cellular component biogenesis	35	0.000122953	0.00117949	6.742672795
cellular response to vascular endothelial growth factor stimulus	4	0.000123632	0.001180514	6.741805713
regulation of muscle hypertrophy	4	0.000123632	0.001180514	6.741805713
regulation of system process	12	0.00012646	0.001204733	6.72149714
cell morphogenesis involved in neuron differentiation	12	0.000128885	0.001225003	6.704811947
mitochondrion transport along microtubule	3	0.000129996	0.001225104	6.704729756
glial cell apoptotic process	3	0.000129996	0.001225104	6.704729756
establishment of mitochondrion localization, microtubule-mediated	3	0.000129996	0.001225104	6.704729756
regulation of biosynthetic process	47	0.000130084	0.001225104	6.704729756

positive regulation of proteolysis	10	0.000130626	0.001227409	6.702849914
cell migration	20	0.00013406	0.001231613	6.699430924
epithelial cell migration	8	0.000134437	0.001231613	6.699430924
positive regulation of superoxide dismutase activity	2	0.000135999	0.001231613	6.699430924
cellular response to beta-amyloid	2	0.000135999	0.001231613	6.699430924
positive regulation of apoptotic process involved in mammary gland involution	2	0.000135999	0.001231613	6.699430924
positive regulation of removal of superoxide radicals	2	0.000135999	0.001231613	6.699430924
response to beta-amyloid	2	0.000135999	0.001231613	6.699430924
apoptotic process involved in mammary gland involution	2	0.000135999	0.001231613	6.699430924
positive regulation of apoptotic process involved in morphogenesis	2	0.000135999	0.001231613	6.699430924
regulation of mammary gland involution	2	0.000135999	0.001231613	6.699430924
positive regulation of mammary gland involution	2	0.000135999	0.001231613	6.699430924
positive regulation of apoptotic process involved in development	2	0.000135999	0.001231613	6.699430924
extrinsic apoptotic signaling pathway in absence of ligand	5	0.000136182	0.001231613	6.699430924
signal transduction in absence of ligand	5	0.000136182	0.001231613	6.699430924
response to nutrient	7	0.000136271	0.001231613	6.699430924
protein complex assembly	22	0.000136922	0.001231613	6.699430924
protein complex biogenesis	22	0.000136922	0.001231613	6.699430924
membrane protein ectodomain proteolysis	4	0.000137045	0.001231613	6.699430924
response to cadmium ion	4	0.000137045	0.001231613	6.699430924
regulation of endothelial cell apoptotic process	4	0.000137045	0.001231613	6.699430924
positive regulation of establishment of protein localization	12	0.000141617	0.001269937	6.668787648
regulation of locomotion	15	0.000144217	0.001290445	6.652768269
positive regulation of molecular function	26	0.000145042	0.001295015	6.649232808
locomotion	23	0.000146696	0.001304898	6.641630144
regulation of insulin secretion	7	0.000146781	0.001304898	6.641630144
epithelium migration	8	0.000147444	0.001307969	6.639279856
regulation of developmental growth	9	0.000150311	0.001327691	6.624313885
hormone secretion	9	0.000150311	0.001327691	6.624313885
hematopoietic or lymphoid organ development	15	0.00015073	0.001328548	6.623668361
positive regulation of apoptotic signaling pathway	7	0.000152278	0.001339328	6.615586941
peptidyl-tyrosine phosphorylation	10	0.000153557	0.00134771	6.609348075
execution phase of apoptosis	5	0.00015481	0.00135582	6.603348975
positive regulation of cellular component biogenesis	11	0.000155686	0.001360606	6.599825221
response to ethanol	6	0.000158088	0.001378674	6.586632905
negative regulation of glucose transport	3	0.000159206	0.00137967	6.58591082
negative regulation of hydrogen peroxide-induced cell death	3	0.000159206	0.00137967	6.58591082
negative regulation of response to reactive oxygen species	3	0.000159206	0.00137967	6.58591082
peptidyl-tyrosine modification	10	0.000160702	0.001389715	6.578656858
positive regulation of sequence-specific DNA binding transcription factor activity	8	0.000161471	0.001393451	6.575971979
negative regulation of protein metabolic process	18	0.000166511	0.001433937	6.547331397
cell motility	21	0.000170451	0.001461764	6.528111564
localization of cell	21	0.000170451	0.001461764	6.528111564
regulation of protein complex assembly	10	0.000171943	0.001471503	6.521470643

synaptic vesicle exocytosis	5	0.000175326	0.001497349	6.504058803
positive regulation of nervous system development	11	0.000175724	0.001497651	6.503857432
negative regulation of endopeptidase activity	8	0.000176581	0.001498006	6.503620596
tissue migration	8	0.000176581	0.001498006	6.503620596
regulation of cellular component biogenesis	15	0.000176855	0.001498006	6.503620596
regulation of cellular process	87	0.000183962	0.001555008	6.466274697
protein localization	31	0.000185982	0.001565409	6.459607941
positive regulation of myeloid cell differentiation	5	0.000186331	0.001565409	6.459607941
negative regulation of cellular catabolic process	5	0.000186331	0.001565409	6.459607941
establishment of mitochondrion localization	3	0.000192368	0.001609568	6.431789207
regulation of endothelial cell chemotaxis	3	0.000192368	0.001609568	6.431789207
peptide hormone secretion	8	0.000192837	0.001610229	6.4313788
organonitrogen compound metabolic process	29	0.000193571	0.001613094	6.429600934
axon development	11	0.00019406	0.001613903	6.429099748
cellular metabolic process	86	0.000194786	0.001616686	6.427376851
organic substance catabolic process	26	0.000197883	0.0016363	6.415317617
response to transition metal nanoparticle	6	0.000197943	0.0016363	6.415317617
response to insulin	8	0.000204343	0.001685832	6.385496288
regulation of ERK1 and ERK2 cascade	8	0.000210304	0.001731549	6.358739131
response to bacterium	12	0.000218879	0.001798556	6.3207709
cellular response to peptide	9	0.000225023	0.001845365	6.295078338
macromolecule localization	34	0.000225486	0.001845498	6.295006078
regulation of neurotransmitter levels	7	0.000232264	0.001897205	6.267373676
anatomical structure formation involved in morphogenesis	19	0.000234909	0.001915016	6.258029131
peptide secretion	8	0.000235597	0.001916841	6.257076612
single-organism membrane organization	16	0.000238722	0.001938439	6.245872331
morphogenesis of a branching epithelium	7	0.000240221	0.001938515	6.245833298
neurotransmitter transport	7	0.000240221	0.001938515	6.245833298
protein insertion into membrane	4	0.000240611	0.001938515	6.245833298
endothelial cell apoptotic process	4	0.000240611	0.001938515	6.245833298
negative regulation of peptidase activity	8	0.000242296	0.001948283	6.24080677
regulation of protein secretion	10	0.000243739	0.001956079	6.236813193
cellular response to metal ion	6	0.000245339	0.001965091	6.232216924
cellular response to insulin stimulus	7	0.000248396	0.001985722	6.221772734
leukocyte differentiation	11	0.000259118	0.002067432	6.181447832
regulation of ion transmembrane transport	10	0.000259673	0.002067859	6.181241279
extrinsic apoptotic signaling pathway via death domain receptors	5	0.000263846	0.002097039	6.167228721
nitrogen compound transport	14	0.000268319	0.002123602	6.154641365
immune system development	15	0.000269086	0.002123602	6.154641365
response to L-ascorbic acid	2	0.000270791	0.002123602	6.154641365
response to electrical stimulus involved in regulation of muscle adaptation	2	0.000270791	0.002123602	6.154641365
regulation of superoxide dismutase activity	2	0.000270791	0.002123602	6.154641365
regulation of ubiquitin-specific protease activity	2	0.000270791	0.002123602	6.154641365
regulation of TRAIL-activated apoptotic signaling pathway	2	0.000270791	0.002123602	6.154641365

response to salt stress	3	0.000271429	0.00212456	6.15419061
negative regulation of binding	6	0.000277797	0.002161537	6.136935565
positive regulation of protein catabolic process	8	0.000278174	0.002161537	6.136935565
neuron projection morphogenesis	12	0.000278763	0.002161537	6.136935565
cellular response to nitrogen compound	12	0.000278763	0.002161537	6.136935565
positive regulation of proteasomal protein catabolic process	5	0.000278836	0.002161537	6.136935565
positive regulation of protein kinase activity	11	0.000279297	0.002161537	6.136935565
carbohydrate transport	7	0.000283369	0.002188948	6.124334283
posttranscriptional regulation of gene expression	11	0.000284544	0.002189676	6.124001612
positive regulation of reactive oxygen species biosynthetic process	4	0.000285056	0.002189676	6.124001612
positive regulation of oxidoreductase activity	4	0.000285056	0.002189676	6.124001612
protein catabolic process	15	0.000287943	0.002207739	6.115786376
protein secretion	11	0.000289875	0.002218427	6.110956758
metal ion homeostasis	12	0.000293266	0.002240228	6.101177618
regulation of oxidoreductase activity	5	0.000294463	0.002245216	6.098953693
regulation of cellular protein localization	12	0.000308407	0.002347196	6.054533866
mitochondrion organization	13	0.000309054	0.002347783	6.054283605
ERK1 and ERK2 cascade	8	0.000309912	0.002349973	6.053351376
regulation of endocytosis	7	0.000312114	0.002362334	6.048105289
exocytosis	10	0.000312814	0.002363292	6.047699689
regulation of macromolecule biosynthetic process	44	0.000317154	0.002374525	6.042957935
response to hyperoxia	3	0.000317755	0.002374525	6.042957935
intermediate filament organization	3	0.000317755	0.002374525	6.042957935
regulation of vascular smooth muscle cell proliferation	3	0.000317755	0.002374525	6.042957935
vascular smooth muscle cell proliferation	3	0.000317755	0.002374525	6.042957935
response to increased oxygen levels	3	0.000317755	0.002374525	6.042957935
response to peptide hormone	10	0.000319243	0.002381335	6.040094102
regulation of cell motility	14	0.000328081	0.002440462	6.015567917
positive regulation of catabolic process	9	0.000328353	0.002440462	6.015567917
signal transduction by p53 class mediator	8	0.000335608	0.002489896	5.995514245
negative regulation of macromolecule biosynthetic process	21	0.000347044	0.002570116	5.963804284
peptide transport	8	0.000353686	0.002613522	5.947056487
organelle localization	11	0.000354303	0.002613522	5.947056487
cellular macromolecule metabolic process	74	0.000354805	0.002613522	5.947056487
lipopolysaccharide-mediated signaling pathway	4	0.000362354	0.00266437	5.92778761
calcium ion transmembrane transport	8	0.000363018	0.002664504	5.927737428
developmental growth	12	0.000363775	0.002665315	5.927433018
regulation of stress-activated MAPK cascade	7	0.000365195	0.002666936	5.92682498
inorganic ion homeostasis	13	0.000365289	0.002666936	5.92682498
cellular metal ion homeostasis	11	0.000367225	0.002676333	5.92330763
negative regulation of oxidative stress-induced intrinsic apoptotic signaling pathway	3	0.000368888	0.002678983	5.922317937
regulation of membrane protein ectodomain proteolysis	3	0.000368888	0.002678983	5.922317937
regulation of stress-activated protein kinase signaling cascade	7	0.000376631	0.002725618	5.905060158
insulin secretion	7	0.000376631	0.002725618	5.905060158

vascular endothelial growth factor receptor signaling pathway	5	0.000382799	0.002755754	5.894064197
negative regulation of intrinsic apoptotic signaling pathway	5	0.000382799	0.002755754	5.894064197
negative regulation of protein catabolic process	5	0.000382799	0.002755754	5.894064197
positive regulation of intrinsic apoptotic signaling pathway	4	0.000391153	0.002810984	5.874220809
negative regulation of catalytic activity	15	0.000395233	0.002834474	5.865898992
response to ionizing radiation	6	0.000395796	0.002834474	5.865898992
striated muscle tissue development	9	0.000401965	0.002873669	5.852165687
axonogenesis	10	0.000421523	0.003008274	5.80638879
cell growth	11	0.000422962	0.003010086	5.805786554
vesicle-mediated transport in synapse	5	0.000423236	0.003010086	5.805786554
regulation of cysteine-type endopeptidase activity involved in apoptotic signaling pathway	3	0.00042503	0.003014229	5.804411102
response to carbohydrate	7	0.00042528	0.003014229	5.804411102
presynaptic process involved in chemical synaptic transmission	6	0.000426668	0.00301787	5.803204043
negative regulation of transcription from RNA polymerase II promoter	14	0.000427257	0.00301787	5.803204043
gland development	10	0.000438147	0.003089501	5.779745697
metabolic process	90	0.000440541	0.003101079	5.776005321
nucleic acid-templated transcription	41	0.000441616	0.003103349	5.775273365
membrane depolarization	5	0.000444624	0.003117575	5.770699889
positive regulation of cellular catabolic process	8	0.000445152	0.003117575	5.770699889
negative regulation of cysteine-type endopeptidase activity involved in apoptotic signaling pathway	2	0.000449318	0.003130804	5.766465585
mammary gland branching involved in pregnancy	2	0.000449318	0.003130804	5.766465585
positive regulation of endothelial cell chemotaxis by VEGF-activated vascular endothelial growth factor receptor signaling p	2	0.000449318	0.003130804	5.766465585
cellular ion homeostasis	12	0.000455309	0.003167197	5.754908159
protein complex subunit organization	23	0.000458529	0.003184231	5.749544477
erythrocyte differentiation	5	0.000466816	0.003236332	5.733314669
negative regulation of MAPK cascade	6	0.000476469	0.003297713	5.714526226
response to tumor necrosis factor	8	0.000479598	0.003313807	5.709657506
angiogenesis	10	0.000482113	0.003325614	5.706100815
tube development	12	0.000484787	0.003328303	5.705292678
positive regulation of kinase activity	11	0.000485649	0.003328303	5.705292678
positive regulation of blood vessel endothelial cell migration	3	0.000486379	0.003328303	5.705292678
skeletal muscle adaptation	3	0.000486379	0.003328303	5.705292678
ovarian follicle development	4	0.000487344	0.003328303	5.705292678
membrane protein proteolysis	4	0.000487344	0.003328303	5.705292678
single-organism organelle organization	23	0.000495938	0.003381392	5.689467793
vesicle-mediated transport	21	0.00049853	0.003393462	5.685904497
regulation of vesicle-mediated transport	10	0.000500698	0.003402603	5.683214512
positive regulation of protein secretion	7	0.000507555	0.003443527	5.671259072
regulation of RNA stability	6	0.00051211	0.003468725	5.663968127
myotube differentiation	5	0.000513695	0.003473756	5.662518912
negative regulation of ERK1 and ERK2 cascade	4	0.000522842	0.003529826	5.646506763
branching morphogenesis of an epithelial tube	6	0.000530691	0.003576964	5.633240867
muscle tissue development	9	0.000543186	0.003655207	5.611602434
ionotropic glutamate receptor signaling pathway	3	0.000553126	0.003709993	5.596725209

endothelial cell chemotaxis	3	0.000553126	0.003709993	5.596725209
muscle organ development	9	0.00055466	0.003714244	5.595580164
regulation of cell migration	13	0.00055707	0.003724336	5.592866823
amide transport	8	0.000582159	0.00388577	5.550434146
positive regulation of MAP kinase activity	7	0.000585405	0.003901127	5.546489767
receptor metabolic process	6	0.0005896	0.003910533	5.54408163
response to alcohol	6	0.0005896	0.003910533	5.54408163
calcium ion regulated exocytosis	5	0.000590544	0.003910533	5.54408163
regulation of catalytic activity	29	0.000590609	0.003910533	5.54408163
regulation of hydrolase activity	20	0.000597785	0.003949021	5.534287689
regulation of monooxygenase activity	4	0.000599294	0.003949021	5.534287689
maternal process involved in female pregnancy	4	0.000599294	0.003949021	5.534287689
epithelium development	17	0.000608193	0.004001273	5.521142785
positive regulation of organelle organization	11	0.000614108	0.004033585	5.513099629
positive regulation of cellular protein localization	9	0.000615061	0.004033585	5.513099629
positive regulation of protein complex disassembly	3	0.000625461	0.004095277	5.497920893
regulation of peptidyl-tyrosine phosphorylation	7	0.000636493	0.004145878	5.485640807
chemical synaptic transmission	12	0.000638214	0.004145878	5.485640807
anterograde trans-synaptic signaling	12	0.000638214	0.004145878	5.485640807
synaptic signaling	12	0.000638214	0.004145878	5.485640807
trans-synaptic signaling	12	0.000638214	0.004145878	5.485640807
positive regulation of developmental growth	6	0.000653469	0.0042383	5.463593146
cellular response to hormone stimulus	12	0.000667385	0.004311263	5.446524276
positive regulation of receptor binding	2	0.000670989	0.004311263	5.446524276
axonal transport of mitochondrion	2	0.000670989	0.004311263	5.446524276
positive regulation of oxidative phosphorylation	2	0.000670989	0.004311263	5.446524276
flavin-containing compound metabolic process	2	0.000670989	0.004311263	5.446524276
positive regulation of cell migration by vascular endothelial growth factor signaling pathway	2	0.000670989	0.004311263	5.446524276
erythrocyte homeostasis	5	0.000675717	0.004334886	5.44105988
glutathione metabolic process	4	0.000683362	0.004370336	5.43291536
import into cell	4	0.000683362	0.004370336	5.43291536
negative regulation of endothelial cell apoptotic process	3	0.000703565	0.004492579	5.405328302
ovulation cycle	5	0.000706054	0.004501502	5.403344241
regulation of homeostatic process	10	0.000721589	0.004593445	5.383124906
cation transmembrane transport	13	0.000744235	0.004730306	5.353765288
cellular response to peptide hormone stimulus	8	0.000751484	0.004769035	5.345611265
synaptic vesicle cycle	5	0.000769772	0.004871303	5.324393873
regulation of axonogenesis	6	0.000771705	0.004871303	5.324393873
regeneration	6	0.000771705	0.004871303	5.324393873
organic cyclic compound biosynthetic process	46	0.000772323	0.004871303	5.324393873
negative regulation of RNA biosynthetic process	18	0.000780823	0.004912829	5.315905386
regulation of nucleic acid-templated transcription	39	0.000782289	0.004912829	5.315905386
immune system process	29	0.00078248	0.004912829	5.315905386
regulation of response to external stimulus	13	0.000795889	0.004978277	5.302671519

organic substance metabolic process	87	0.000797026	0.004978277	5.302671519
regulation of signal transduction by p53 class mediator	6	0.000797217	0.004978277	5.302671519
regulation of sequence-specific DNA binding transcription factor activity	9	0.000797731	0.004978277	5.302671519
nitrogen compound metabolic process	63	0.000808047	0.005027596	5.292813316
cell adhesion	23	0.000808072	0.005027596	5.292813316
cellular response to DNA damage stimulus	14	0.000818673	0.0050771	5.283015007
transcription, DNA-templated	40	0.000822065	0.0050771	5.283015007
positive regulation of protein serine/threonine kinase activity	8	0.000822097	0.0050771	5.283015007
positive regulation of peptidyl-tyrosine phosphorylation	6	0.000823376	0.0050771	5.283015007
cardiac muscle hypertrophy	4	0.000824644	0.0050771	5.283015007
regulation of transcription from RNA polymerase II promoter in response to stress	4	0.000824644	0.0050771	5.283015007
regulation of epithelial cell apoptotic process	4	0.000824644	0.0050771	5.283015007
microtubule-based movement	7	0.000832877	0.005112529	5.276061074
regulation of proteolysis involved in cellular protein catabolic process	7	0.000832877	0.005112529	5.276061074
regulation of fat cell differentiation	5	0.000837691	0.00512682	5.273269617
negative regulation of ion transport	5	0.000837691	0.00512682	5.273269617
activation of protein kinase activity	8	0.000840559	0.005136752	5.271334308
aromatic compound biosynthetic process	45	0.000844523	0.005153341	5.268110048
biological adhesion	23	0.000848528	0.005170129	5.26485768
regulation of cellular biosynthetic process	44	0.000855498	0.005204911	5.258152636
regulation of RNA biosynthetic process	39	0.000866508	0.005264132	5.24683905
RNA biosynthetic process	41	0.000872686	0.005293865	5.24120671
positive regulation of protein transport	10	0.000873969	0.005293865	5.24120671
positive regulation of release of cytochrome c from mitochondria	3	0.000877798	0.005301488	5.23976765
positive regulation of monooxygenase activity	3	0.000877798	0.005301488	5.23976765
macromolecule catabolic process	18	0.000888117	0.005355968	5.229543904
central nervous system development	15	0.000893936	0.005383194	5.224473384
single-organism intracellular transport	13	0.000896503	0.005390779	5.223065443
response to interleukin-1	5	0.000909984	0.005463875	5.209596985
negative regulation of intracellular signal transduction	10	0.000919838	0.005511139	5.200983909
striated muscle hypertrophy	4	0.000929556	0.005511139	5.200983909
mammary gland epithelium development	4	0.000929556	0.005511139	5.200983909
regulation of epithelial cell migration	6	0.000934707	0.005511139	5.200983909
positive regulation of mitochondrion organization	6	0.000934707	0.005511139	5.200983909
peripheral nervous system myelin maintenance	2	0.000935224	0.005511139	5.200983909
positive regulation of oxidative stress-induced intrinsic apoptotic signaling pathway	2	0.000935224	0.005511139	5.200983909
negative regulation of protein ubiquitination involved in ubiquitin-dependent protein catabolic process	2	0.000935224	0.005511139	5.200983909
response to methylmercury	2	0.000935224	0.005511139	5.200983909
regulation of calcidiol 1-monooxygenase activity	2	0.000935224	0.005511139	5.200983909
post-embryonic camera-type eye development	2	0.000935224	0.005511139	5.200983909
histone-threonine phosphorylation	2	0.000935224	0.005511139	5.200983909
TRAIL-activated apoptotic signaling pathway	2	0.000935224	0.005511139	5.200983909
maternal placenta development	3	0.000974272	0.005724886	5.162932564
negative regulation of insulin receptor signaling pathway	3	0.000974272	0.005724886	5.162932564

macromolecule metabolic process	77	0.000983253	0.005765542	5.155856149
negative regulation of ion transmembrane transport	4	0.000985384	0.005765542	5.155856149
regulation of reactive oxygen species biosynthetic process	4	0.000985384	0.005765542	5.155856149
regulation of protein binding	6	0.000994546	0.005810912	5.14801782
gliogenesis	7	0.000996782	0.005815736	5.147187904
regulation of protein kinase activity	13	0.001007621	0.005870676	5.137785535
positive regulation of transferase activity	12	0.001011946	0.005887557	5.134914072
cellular response to steroid hormone stimulus	7	0.001022135	0.005938459	5.126305604
cation homeostasis	12	0.001026072	0.005941632	5.125771507
positive regulation of nucleocytoplasmic transport	5	0.001027002	0.005941632	5.125771507
regulation of endothelial cell migration	5	0.001027002	0.005941632	5.125771507
protein oligomerization	10	0.001034612	0.005977275	5.119790437
muscle hypertrophy	4	0.001043529	0.006020357	5.112608648
macroautophagy	7	0.001074363	0.006168881	5.088237881
negative regulation of fibroblast proliferation	3	0.001077207	0.006168881	5.088237881
protein insertion into mitochondrial membrane involved in apoptotic signaling pathway	3	0.001077207	0.006168881	5.088237881
positive regulation of morphogenesis of an epithelium	3	0.001077207	0.006168881	5.088237881
apoptotic nuclear changes	3	0.001077207	0.006168881	5.088237881
transmembrane transport	19	0.001078246	0.006168881	5.088237881
regulation of DNA-templated transcription in response to stress	4	0.001104043	0.006307723	5.06598048
insulin receptor signaling pathway	5	0.001110992	0.006338645	5.061090286
positive regulation of growth	7	0.00112867	0.006430611	5.046685732
macromolecule biosynthetic process	50	0.001132955	0.006437278	5.045649474
positive regulation of immune system process	15	0.001132961	0.006437278	5.045649474
regulation of hormone levels	10	0.001142117	0.006471472	5.040351728
regulation of cell morphogenesis	10	0.001142117	0.006471472	5.040351728
synaptic vesicle transport	5	0.001154846	0.006525673	5.032011224
establishment of synaptic vesicle localization	5	0.001154846	0.006525673	5.032011224
multi-organism reproductive process	15	0.001158312	0.00652718	5.031780325
regulation of metal ion transport	8	0.00115986	0.00652718	5.031780325
epithelial tube morphogenesis	8	0.00115986	0.00652718	5.031780325
regulation of cell growth	9	0.001163283	0.006537524	5.03019683
epithelial cell differentiation	11	0.001180786	0.006615413	5.01835306
negative regulation of RNA metabolic process	18	0.001185196	0.006615413	5.01835306
response to dexamethasone	3	0.001186765	0.006615413	5.01835306
protein insertion into mitochondrial membrane	3	0.001186765	0.006615413	5.01835306
response to mineralocorticoid	3	0.001186765	0.006615413	5.01835306
negative regulation of cellular response to insulin stimulus	3	0.001186765	0.006615413	5.01835306
regulation of protein transport	13	0.001188402	0.006615596	5.018325401
positive regulation of chemotaxis	5	0.001199971	0.006670998	5.009985791
glial cell differentiation	6	0.001227486	0.006809781	4.989395367
stress-induced premature senescence	2	0.001241444	0.006809781	4.989395367
retrograde axonal transport	2	0.001241444	0.006809781	4.989395367
protein hexamerization	2	0.001241444	0.006809781	4.989395367

skeletal muscle atrophy	2	0.001241444	0.006809781	4.989395367
regulation of oxidative stress-induced neuron intrinsic apoptotic signaling pathway	2	0.001241444	0.006809781	4.989395367
activation of cysteine-type endopeptidase activity involved in apoptotic process by cytochrome c	2	0.001241444	0.006809781	4.989395367
negative regulation of receptor internalization	2	0.001241444	0.006809781	4.989395367
neuron intrinsic apoptotic signaling pathway in response to oxidative stress	2	0.001241444	0.006809781	4.989395367
regulation of protein deubiquitination	2	0.001241444	0.006809781	4.989395367
regulation of anatomical structure size	10	0.001258611	0.00689478	4.976990613
regulation of nucleobase-containing compound metabolic process	42	0.00127082	0.006946153	4.969567316
system process	25	0.001271356	0.006946153	4.969567316
regulation of transferase activity	15	0.001292396	0.007051765	4.95447739
signal transduction in response to DNA damage	5	0.001294121	0.007051847	4.95446571
p38MAPK cascade	3	0.001303105	0.007091436	4.948867444
cellular cation homeostasis	11	0.001307848	0.007107871	4.946552559
regulation of chemotaxis	6	0.001339221	0.0072688	4.924164124
positive regulation of angiogenesis	5	0.001343191	0.007271212	4.923832232
gland morphogenesis	5	0.001343191	0.007271212	4.923832232
negative regulation of transmembrane transport	4	0.001370823	0.007411071	4.904780358
response to monosaccharide	6	0.001378143	0.007440888	4.900765092
single-organism catabolic process	14	0.001382094	0.007452466	4.89921035
regulation of transcription, DNA-templated	38	0.001390366	0.007487284	4.894549151
regulation of organ morphogenesis	7	0.001400317	0.007531041	4.888721979
regulation of cellular macromolecule biosynthetic process	41	0.001404279	0.007542513	4.887199847
inflammatory response	12	0.001417885	0.007605691	4.87885853
regulation of receptor-mediated endocytosis	4	0.001443958	0.007733373	4.862210156
positive regulation of cysteine-type endopeptidase activity	5	0.001445438	0.007733373	4.862210156
regulation of MAP kinase activity	8	0.001450511	0.007750462	4.860002877
cellular divalent inorganic cation homeostasis	9	0.001466367	0.007818832	4.851220049
regulation of cellular protein catabolic process	7	0.001467097	0.007818832	4.851220049
regulation of macroautophagy	5	0.00149866	0.007966458	4.832515307
synaptic vesicle localization	5	0.00149866	0.007966458	4.832515307
negative regulation of nucleobase-containing compound metabolic process	19	0.001513402	0.008034468	4.824014472
blood vessel endothelial cell migration	4	0.001519771	0.008047568	4.822385351
nucleoside phosphate catabolic process	4	0.001519771	0.008047568	4.822385351
heterocycle biosynthetic process	44	0.001534002	0.008104083	4.81538731
membrane organization	16	0.001534373	0.008104083	4.81538731
negative regulation of cellular biosynthetic process	20	0.001548539	0.00814919	4.809836743
regulation of nitrogen compound metabolic process	44	0.001549303	0.00814919	4.809836743
regulation of histone modification	5	0.001553312	0.00814919	4.809836743
organic substance biosynthetic process	57	0.001554526	0.00814919	4.809836743
positive regulation of mitochondrial outer membrane permeabilization involved in apoptotic signaling pathway	3	0.001556742	0.00814919	4.809836743
programmed necrotic cell death	3	0.001556742	0.00814919	4.809836743
regulation of alcohol biosynthetic process	3	0.001556742	0.00814919	4.809836743
ameboidal-type cell migration	8	0.001569527	0.008205703	4.802925926
regulation of branching involved in salivary gland morphogenesis	2	0.001589078	0.008224527	4.800634537

death-inducing signaling complex assembly	2	0.001589078	0.008224527	4.800634537
positive regulation of superoxide anion generation	2	0.001589078	0.008224527	4.800634537
response to cobalt ion	2	0.001589078	0.008224527	4.800634537
regulation of vitamin D biosynthetic process	2	0.001589078	0.008224527	4.800634537
gas homeostasis	2	0.001589078	0.008224527	4.800634537
striated muscle atrophy	2	0.001589078	0.008224527	4.800634537
mammary gland involution	2	0.001589078	0.008224527	4.800634537
response to biotic stimulus	14	0.001600595	0.008273754	4.794666908
calcium ion transport into cytosol	5	0.001609417	0.008308942	4.790423027
regulation of RNA metabolic process	39	0.00161463	0.008325434	4.788440161
cellular response to extracellular stimulus	6	0.001675336	0.008627667	4.752781157
intracellular transport	21	0.001691745	0.008691575	4.745401075
regulation of receptor internalization	3	0.001694336	0.008691575	4.745401075
negative regulation of epithelial cell apoptotic process	3	0.001694336	0.008691575	4.745401075
multicellular organismal homeostasis	8	0.001696174	0.008691575	4.745401075
cellular aromatic compound metabolic process	55	0.001715038	0.008777334	4.73558253
mammary gland development	5	0.00172608	0.008811979	4.731643278
cytosolic calcium ion transport	5	0.00172608	0.008811979	4.731643278
blood vessel morphogenesis	10	0.001745333	0.008899254	4.721787779
positive regulation of exocytosis	4	0.001763795	0.008980174	4.712736056
negative regulation of nucleic acid-templated transcription	17	0.001766713	0.008980174	4.712736056
developmental cell growth	6	0.001768581	0.008980174	4.712736056
reproductive structure development	9	0.001769911	0.008980174	4.712736056
small molecule metabolic process	24	0.001785848	0.009049905	4.705001045
primary metabolic process	83	0.001790692	0.009063318	4.703519971
regulation of cell morphogenesis involved in differentiation	7	0.001799003	0.009094222	4.70011597
extracellular matrix organization	8	0.001830793	0.0092436	4.683823835
hyaluronan metabolic process	3	0.001839303	0.009252591	4.682851644
insulin-like growth factor receptor signaling pathway	3	0.001839303	0.009252591	4.682851644
mitochondrion localization	3	0.001839303	0.009252591	4.682851644
extracellular structure organization	8	0.001865734	0.009374121	4.669802473
reproductive system development	9	0.001892046	0.009494756	4.657015613
alpha-amino acid metabolic process	6	0.001915778	0.009602166	4.645766607
positive regulation of histone modification	4	0.001940807	0.009715812	4.634000611
regulation of kinase activity	13	0.00194562	0.0097281	4.632736702
response to endoplasmic reticulum stress	7	0.001964037	0.009804619	4.62490169
positive regulation of NFAT protein import into nucleus	2	0.001977562	0.009804619	4.62490169
response to mercury ion	2	0.001977562	0.009804619	4.62490169
positive regulation of intracellular estrogen receptor signaling pathway	2	0.001977562	0.009804619	4.62490169
positive regulation of mitochondrial depolarization	2	0.001977562	0.009804619	4.62490169
regulation of apoptotic process involved in morphogenesis	2	0.001977562	0.009804619	4.62490169
regulation of apoptotic process involved in development	2	0.001977562	0.009804619	4.62490169
myoblast fusion	3	0.001991783	0.009827876	4.622532401
response to electrical stimulus	3	0.001991783	0.009827876	4.622532401

negative regulation of proteolysis involved in cellular protein catabolic process	3	0.001991783	0.009827876	4.622532401
regulation of transcription regulatory region DNA binding	3	0.001991783	0.009827876	4.622532401
positive regulation of response to external stimulus	7	0.002007075	0.009891498	4.616079654
regulation of ion homeostasis	6	0.002018947	0.009938132	4.611376188
divalent inorganic cation homeostasis	9	0.002054122	0.010099229	4.595296232
organic cyclic compound metabolic process	56	0.00207797	0.010204318	4.584944361
regulation of gene expression	43	0.002084514	0.010224281	4.582989863
activation of cysteine-type endopeptidase activity involved in apoptotic process	4	0.002129757	0.010421406	4.563893281
negative regulation of protein binding	4	0.002129757	0.010421406	4.563893281
animal organ morphogenesis	15	0.002176125	0.010635684	4.543540496
neurotransmitter secretion	5	0.002183614	0.010647053	4.542472154
signal release from synapse	5	0.002183614	0.010647053	4.542472154
oxidation-reduction process	15	0.002219996	0.010811668	4.527129398
positive regulation of autophagy	4	0.002228833	0.010841907	4.524336347
glucose metabolic process	6	0.00223785	0.010872949	4.521477362
nucleobase-containing compound biosynthetic process	43	0.002241263	0.010876719	4.521130624
positive regulation of endopeptidase activity	5	0.00225554	0.010933139	4.515956818
response to radiation	9	0.00226308	0.010956813	4.513793856
biosynthetic process	57	0.002322448	0.011206946	4.491221499
positive regulation of vasculature development	5	0.002329178	0.011206946	4.491221499
regulation of epidermal growth factor receptor signaling pathway	4	0.002331045	0.011206946	4.491221499
ovulation cycle process	4	0.002331045	0.011206946	4.491221499
nuclear envelope organization	4	0.002331045	0.011206946	4.491221499
receptor internalization	4	0.002331045	0.011206946	4.491221499
nucleus organization	5	0.002404552	0.011515234	4.464084462
dopamine biosynthetic process	2	0.002406335	0.011515234	4.464084462
vitamin D biosynthetic process	2	0.002406335	0.011515234	4.464084462
regulation of removal of superoxide radicals	2	0.002406335	0.011515234	4.464084462
tube morphogenesis	8	0.002412651	0.011532082	4.462622411
regulation of fibroblast proliferation	4	0.002436441	0.011632312	4.453968507
morphogenesis of an epithelium	10	0.002478185	0.011817933	4.438137176
placenta development	5	0.002481686	0.011820963	4.437880834
positive regulation of glucose import	3	0.002495627	0.011832712	4.436887357
response to antibiotic	3	0.002495627	0.011832712	4.436887357
striated muscle adaptation	3	0.002495627	0.011832712	4.436887357
cellular modified amino acid biosynthetic process	3	0.002495627	0.011832712	4.436887357
fibroblast proliferation	4	0.00254507	0.012039467	4.419565075
positive regulation of chromatin organization	4	0.00254507	0.012039467	4.419565075
regulation of cation transmembrane transport	6	0.002599224	0.012281557	4.399656577
single organism reproductive process	17	0.002605051	0.012295007	4.398562058
cellular nitrogen compound metabolic process	58	0.002613951	0.01232023	4.396512639
establishment of protein localization	24	0.002616369	0.01232023	4.396512639
organonitrogen compound catabolic process	8	0.00263592	0.012398142	4.39020863
regulation of ERBB signaling pathway	4	0.002656983	0.012454609	4.385664516

regulation of generation of precursor metabolites and energy	4	0.002656983	0.012454609	4.385664516
regulation of calcium ion transport into cytosol	4	0.002656983	0.012454609	4.385664516
positive regulation of ion transport	6	0.002663478	0.012460735	4.385172806
negative regulation of fat cell differentiation	3	0.002679467	0.012460735	4.385172806
negative regulation of stress-activated MAPK cascade	3	0.002679467	0.012460735	4.385172806
positive regulation of cell-matrix adhesion	3	0.002679467	0.012460735	4.385172806
negative regulation of stress-activated protein kinase signaling cascade	3	0.002679467	0.012460735	4.385172806
negative regulation of mitochondrion organization	3	0.002679467	0.012460735	4.385172806
regulation of mitochondrial outer membrane permeabilization involved in apoptotic signaling pathway	3	0.002679467	0.012460735	4.385172806
protein localization to nucleus	8	0.002682456	0.012460735	4.385172806
negative regulation of cell differentiation	11	0.002717168	0.012607781	4.373441118
regulation of mRNA stability	5	0.002723884	0.012610576	4.373219436
kidney epithelium development	5	0.002723884	0.012610576	4.373219436
positive regulation of peptide hormone secretion	4	0.002772228	0.012805645	4.35786918
epithelial cell apoptotic process	4	0.002772228	0.012805645	4.35786918
tissue morphogenesis	11	0.002822032	0.013021122	4.341182458
regulation of immune system process	18	0.002839692	0.013087968	4.33606195
positive regulation of macrophage differentiation	2	0.002874843	0.013148215	4.33146927
positive regulation of execution phase of apoptosis	2	0.002874843	0.013148215	4.33146927
regulation of vitamin metabolic process	2	0.002874843	0.013148215	4.33146927
myelin maintenance	2	0.002874843	0.013148215	4.33146927
muscle atrophy	2	0.002874843	0.013148215	4.33146927
positive regulation of membrane depolarization	2	0.002874843	0.013148215	4.33146927
regulation of DNA metabolic process	8	0.002875076	0.013148215	4.33146927
neuron projection extension	5	0.002894587	0.013222781	4.325814105
cellular response to abiotic stimulus	7	0.00291445	0.01329879	4.320082205
regulation of nucleocytoplasmic transport	6	0.002932497	0.013366356	4.315014504
cellular nitrogen compound biosynthetic process	47	0.002976804	0.013553331	4.301122947
response to other organism	13	0.003000868	0.013632797	4.295276853
response to external biotic stimulus	13	0.003000868	0.013632797	4.295276853
hydrogen peroxide metabolic process	3	0.003071712	0.013914073	4.27485447
positive regulation of regulated secretory pathway	3	0.003071712	0.013914073	4.27485447
protein kinase B signaling	5	0.003072902	0.013914073	4.27485447
reproductive process	18	0.003107723	0.014056311	4.26468382
reproduction	18	0.003158665	0.014271078	4.249520308
ossification	8	0.003184106	0.014370282	4.242592929
cell part morphogenesis	13	0.00319859	0.014419875	4.23914784
negative regulation of cytokine production	6	0.003221381	0.014506766	4.233140101
regulation of chromatin organization	5	0.003259021	0.014660264	4.222614571
positive regulation of peptide secretion	4	0.003267498	0.014682384	4.221106904
nuclear import	7	0.003277964	0.014692108	4.220444806
positive regulation of glucose transport	3	0.003280347	0.014692108	4.220444806
regulation of insulin receptor signaling pathway	3	0.003280347	0.014692108	4.220444806
positive regulation of cell growth	5	0.003355067	0.014996664	4.199927474

inorganic cation transmembrane transport	11	0.003355617	0.014996664	4.199927474
negative regulation of glucose import	2	0.003382537	0.015003189	4.199492489
positive regulation of vascular smooth muscle cell proliferation	2	0.003382537	0.015003189	4.199492489
hepatocyte growth factor receptor signaling pathway	2	0.003382537	0.015003189	4.199492489
response to vitamin E	2	0.003382537	0.015003189	4.199492489
regulation of proton transport	2	0.003382537	0.015003189	4.199492489
induction of positive chemotaxis	2	0.003382537	0.015003189	4.199492489
regulation of superoxide anion generation	2	0.003382537	0.015003189	4.199492489
female gonad development	4	0.003400128	0.015065012	4.195380319
positive regulation of peptidase activity	5	0.003453136	0.015283459	4.180984117
organic substance transport	29	0.003463086	0.01531107	4.179179174
nucleocytoplasmic transport	9	0.003473292	0.015339753	4.17730758
negative regulation of cell proliferation	11	0.003479903	0.015352512	4.176476161
response to nicotine	3	0.003497471	0.015397084	4.173577121
mammary gland morphogenesis	3	0.003497471	0.015397084	4.173577121
mitochondrial membrane organization	4	0.003536377	0.015551765	4.163581114
pallium development	5	0.003553251	0.015609329	4.159886554
behavior	10	0.003674604	0.016115527	4.127972036
positive regulation of protein import into nucleus	4	0.003676294	0.016115527	4.127972036
cellular calcium ion homeostasis	8	0.00369554	0.016182698	4.123812647
endomembrane system organization	10	0.003722847	0.016200592	4.122707499
positive regulation of DNA binding	3	0.003723191	0.016200592	4.122707499
regulation of myoblast differentiation	3	0.003723191	0.016200592	4.122707499
syncytium formation by plasma membrane fusion	3	0.003723191	0.016200592	4.122707499
necrotic cell death	3	0.003723191	0.016200592	4.122707499
negative regulation of cellular protein catabolic process	3	0.003723191	0.016200592	4.122707499
positive regulation of cell adhesion	8	0.003756082	0.016309303	4.116019588
proteasome-mediated ubiquitin-dependent protein catabolic process	8	0.003756082	0.016309303	4.116019588
regulation of intracellular transport	9	0.003789074	0.016421548	4.109160887
nucleic acid metabolic process	48	0.003789894	0.016421548	4.109160887
nuclear transport	9	0.003899208	0.016877474	4.081775432
negative regulation of growth of symbiont in host	2	0.003928875	0.016881883	4.081514218
myoblast proliferation	2	0.003928875	0.016881883	4.081514218
response to lead ion	2	0.003928875	0.016881883	4.081514218
fat-soluble vitamin biosynthetic process	2	0.003928875	0.016881883	4.081514218
positive regulation of nuclease activity	2	0.003928875	0.016881883	4.081514218
spongiotrophoblast layer development	2	0.003928875	0.016881883	4.081514218
response to stimulus involved in regulation of muscle adaptation	2	0.003928875	0.016881883	4.081514218
telencephalon development	6	0.003948617	0.016934789	4.078385259
acute-phase response	3	0.003957609	0.016934789	4.078385259
neuron projection regeneration	3	0.003957609	0.016934789	4.078385259
T cell apoptotic process	3	0.003957609	0.016934789	4.078385259
positive regulation of protein import	4	0.003967313	0.016941165	4.078008841
development of primary female sexual characteristics	4	0.003967313	0.016941165	4.078008841

regulation of protein serine/threonine kinase activity	9	0.004011843	0.0171136	4.067881786
response to gamma radiation	3	0.004200826	0.017845938	4.025979341
cellular component disassembly involved in execution phase of apoptosis	3	0.004200826	0.017845938	4.025979341
positive regulation of myeloid leukocyte differentiation	3	0.004200826	0.017845938	4.025979341
regulation of extrinsic apoptotic signaling pathway in absence of ligand	3	0.004200826	0.017845938	4.025979341
regulation of DNA binding	4	0.004273553	0.018136221	4.009844173
positive regulation of hemopoiesis	5	0.004313367	0.018286369	4.001599347
protein autophosphorylation	6	0.004401804	0.018642135	3.982330925
negative regulation of nitrogen compound metabolic process	19	0.004432518	0.018752959	3.976403709
mitochondrial outer membrane permeabilization	3	0.004452938	0.018786645	3.974609017
G1/S transition of mitotic cell cycle	6	0.004496867	0.018786645	3.974609017
3'-UTR-mediated mRNA stabilization	2	0.004513318	0.018786645	3.974609017
sequestering of triglyceride	2	0.004513318	0.018786645	3.974609017
positive regulation of membrane protein ectodomain proteolysis	2	0.004513318	0.018786645	3.974609017
positive regulation of histone deacetylation	2	0.004513318	0.018786645	3.974609017
positive regulation of transcription regulatory region DNA binding	2	0.004513318	0.018786645	3.974609017
positive regulation of vascular endothelial growth factor receptor signaling pathway	2	0.004513318	0.018786645	3.974609017
hemoglobin metabolic process	2	0.004513318	0.018786645	3.974609017
drug catabolic process	2	0.004513318	0.018786645	3.974609017
regulation of growth of symbiont in host	2	0.004513318	0.018786645	3.974609017
negative regulation of growth of symbiont involved in interaction with host	2	0.004513318	0.018786645	3.974609017
regulation of NFAT protein import into nucleus	2	0.004513318	0.018786645	3.974609017
regulation of oxidative phosphorylation	2	0.004513318	0.018786645	3.974609017
post-embryonic animal organ development	2	0.004513318	0.018786645	3.974609017
heterocycle metabolic process	53	0.004519256	0.018786645	3.974609017
RNA metabolic process	44	0.004522184	0.018786645	3.974609017
cell-cell adhesion	16	0.004522458	0.018786645	3.974609017
calcium ion homeostasis	8	0.004544265	0.018858241	3.970805256
regulation of cytokine production	10	0.004565668	0.018928019	3.967111976
cellular response to glucocorticoid stimulus	3	0.004714039	0.019445411	3.940144179
syncytium formation	3	0.004714039	0.019445411	3.940144179
regulation of blood vessel endothelial cell migration	3	0.004714039	0.019445411	3.940144179
positive regulation of mitochondrial membrane permeability involved in apoptotic process	3	0.004714039	0.019445411	3.940144179
mitochondrial outer membrane permeabilization involved in programmed cell death	3	0.004714039	0.019445411	3.940144179
cerebral cortex development	4	0.004762246	0.019624639	3.930969387
gene expression	49	0.004778146	0.019670511	3.928634666
single-organism behavior	8	0.004833489	0.019878508	3.918116111
establishment of vesicle localization	6	0.004892351	0.020100547	3.907008269
intrinsic apoptotic signaling pathway in response to DNA damage	4	0.004933143	0.020247976	3.899700432
regulation of steroid biosynthetic process	3	0.004984219	0.020416986	3.891388069
regulation of smooth muscle cell migration	3	0.004984219	0.020416986	3.891388069
multicellular organismal reproductive process	12	0.005011838	0.020509753	3.886854747
organic hydroxy compound biosynthetic process	5	0.005051822	0.020652892	3.879899935
leukocyte cell-cell adhesion	9	0.005075733	0.020730099	3.876168557

negative regulation of calcium ion transport into cytosol	2	0.005135334	0.020849657	3.870417798
response to vitamin A	2	0.005135334	0.020849657	3.870417798
modulation of growth of symbiont involved in interaction with host	2	0.005135334	0.020849657	3.870417798
NFAT protein import into nucleus	2	0.005135334	0.020849657	3.870417798
nitric-oxide synthase biosynthetic process	2	0.005135334	0.020849657	3.870417798
regulation of nitric-oxide synthase biosynthetic process	2	0.005135334	0.020849657	3.870417798
rhythmic process	7	0.005178153	0.021002831	3.863098034
spliceosomal complex assembly	3	0.005263567	0.021307372	3.848702173
positive regulation of mitochondrial membrane permeability	3	0.005263567	0.021307372	3.848702173
regulation of regulated secretory pathway	4	0.005287201	0.021382061	3.845202986
hexose metabolic process	6	0.005312883	0.02146488	3.841337187
urogenital system development	7	0.005359791	0.021633209	3.833525676
single organism cell adhesion	12	0.005499292	0.022174564	3.808809431
cell cycle G1/S phase transition	6	0.005532826	0.022287997	3.803707007
cellular response to corticosteroid stimulus	3	0.005552165	0.022322299	3.802169124
positive regulation of epithelial cell differentiation	3	0.005552165	0.022322299	3.802169124
multicellular organism reproduction	12	0.005612599	0.022543301	3.792317335
positive regulation of cell cycle	7	0.005641187	0.022636087	3.788209878
DNA damage response, signal transduction by p53 class mediator	4	0.005657894	0.022681061	3.786225009
cellular biosynthetic process	54	0.005720505	0.022909791	3.776190909
positive regulation of proteolysis involved in cellular protein catabolic process	5	0.00573238	0.022935079	3.775087709
negative regulation of release of cytochrome c from mitochondria	2	0.005794396	0.023049067	3.770129992
thymocyte apoptotic process	2	0.005794396	0.023049067	3.770129992
positive regulation of microtubule polymerization or depolymerization	2	0.005794396	0.023049067	3.770129992
regulation of receptor binding	2	0.005794396	0.023049067	3.770129992
neurotransmitter reuptake	2	0.005794396	0.023049067	3.770129992
negative regulation of receptor-mediated endocytosis	2	0.005794396	0.023049067	3.770129992
cell chemotaxis	6	0.005875159	0.023347812	3.757251992
lung development	5	0.006021898	0.023888467	3.734359473
muscle contraction	7	0.006033466	0.023888467	3.734359473
platelet degranulation	4	0.006045561	0.023888467	3.734359473
calcium ion transmembrane import into cytosol	4	0.006045561	0.023888467	3.734359473
calcium ion import into cytosol	4	0.006045561	0.023888467	3.734359473
proteasomal protein catabolic process	8	0.006045954	0.023888467	3.734359473
cellular macromolecule biosynthetic process	46	0.006076453	0.023986	3.730284954
negative regulation of multicellular organismal process	14	0.006110949	0.024060717	3.727174764
regulation of extrinsic apoptotic signaling pathway via death domain receptors	3	0.006157437	0.024060717	3.727174764
regulation of mitochondrial membrane permeability involved in apoptotic process	3	0.006157437	0.024060717	3.727174764
vesicle localization	6	0.006232732	0.024060717	3.727174764
female sex differentiation	4	0.006450534	0.024060717	3.727174764
mammary gland alveolus development	2	0.006489982	0.024060717	3.727174764
growth of symbiont in host	2	0.006489982	0.024060717	3.727174764
mammary gland lobule development	2	0.006489982	0.024060717	3.727174764
regulation of mitochondrial depolarization	2	0.006489982	0.024060717	3.727174764

catechol-containing compound biosynthetic process	2	0.006489982	0.024060717	3.727174764
catecholamine biosynthetic process	2	0.006489982	0.024060717	3.727174764
cell junction organization	6	0.006605931	0.024060717	3.727174764
respiratory tube development	5	0.006631596	0.024060717	3.727174764
organic hydroxy compound metabolic process	8	0.006685424	0.024060717	3.727174764
monosaccharide metabolic process	6	0.006733869	0.024060717	3.727174764
negative regulation of branching involved in lung morphogenesis	1	0.006777831	0.024060717	3.727174764
positive regulation of leukocyte adhesion to arterial endothelial cell	1	0.006777831	0.024060717	3.727174764
Cajal-Retzius cell differentiation	1	0.006777831	0.024060717	3.727174764
mitotic spindle disassembly	1	0.006777831	0.024060717	3.727174764
nitric oxide storage	1	0.006777831	0.024060717	3.727174764
glycogen cell differentiation involved in embryonic placenta development	1	0.006777831	0.024060717	3.727174764
negative regulation of protein kinase activity by protein phosphorylation	1	0.006777831	0.024060717	3.727174764
positive regulation of calcium-dependent cell-cell adhesion	1	0.006777831	0.024060717	3.727174764
positive regulation of adrenergic receptor signaling pathway involved in heart process	1	0.006777831	0.024060717	3.727174764
cellular response to lead ion	1	0.006777831	0.024060717	3.727174764
positive regulation of DNA-templated transcription, termination	1	0.006777831	0.024060717	3.727174764
positive regulation of termination of RNA polymerase II transcription, poly(A)-coupled	1	0.006777831	0.024060717	3.727174764
male somatic sex determination	1	0.006777831	0.024060717	3.727174764
activation of prostate induction by androgen receptor signaling pathway	1	0.006777831	0.024060717	3.727174764
positive regulation of myofibroblast differentiation	1	0.006777831	0.024060717	3.727174764
flavin adenine dinucleotide catabolic process	1	0.006777831	0.024060717	3.727174764
positive regulation of protein K63-linked deubiquitination	1	0.006777831	0.024060717	3.727174764
positive regulation of Lys63-specific deubiquitinase activity	1	0.006777831	0.024060717	3.727174764
release of matrix enzymes from mitochondria	1	0.006777831	0.024060717	3.727174764
B cell receptor apoptotic signaling pathway	1	0.006777831	0.024060717	3.727174764
negative regulation of erythrocyte apoptotic process	1	0.006777831	0.024060717	3.727174764
positive regulation of Rho guanyl-nucleotide exchange factor activity	1	0.006777831	0.024060717	3.727174764
intermediate-density lipoprotein particle remodeling	1	0.006777831	0.024060717	3.727174764
nuclear inner membrane organization	1	0.006777831	0.024060717	3.727174764
D-serine catabolic process	1	0.006777831	0.024060717	3.727174764
D-alanine catabolic process	1	0.006777831	0.024060717	3.727174764
age-dependent response to reactive oxygen species	1	0.006777831	0.024060717	3.727174764
vasodilation by acetylcholine involved in regulation of systemic arterial blood pressure	1	0.006777831	0.024060717	3.727174764
negative regulation of T cell antigen processing and presentation	1	0.006777831	0.024060717	3.727174764
cellular response to iron ion starvation	1	0.006777831	0.024060717	3.727174764
negative regulation of antigen processing and presentation of endogenous peptide antigen via MHC class I	1	0.006777831	0.024060717	3.727174764
initiation of primordial ovarian follicle growth	1	0.006777831	0.024060717	3.727174764
phosphocreatine biosynthetic process	1	0.006777831	0.024060717	3.727174764
positive regulation of connective tissue growth factor production	1	0.006777831	0.024060717	3.727174764
negative regulation of lung ciliated cell differentiation	1	0.006777831	0.024060717	3.727174764
positive regulation of lung goblet cell differentiation	1	0.006777831	0.024060717	3.727174764
positive regulation of pancreatic stellate cell proliferation	1	0.006777831	0.024060717	3.727174764
positive regulation of protein kinase D signaling	1	0.006777831	0.024060717	3.727174764

negative regulation of cellular organofluorine metabolic process	1	0.006777831	0.024060717	3.727174764
enzyme active site formation via L-cysteine sulfinic acid	1	0.006777831	0.024060717	3.727174764
cellular response to glyoxal	1	0.006777831	0.024060717	3.727174764
peptidyl-cysteine deglycation	1	0.006777831	0.024060717	3.727174764
peptidyl-arginine deglycation	1	0.006777831	0.024060717	3.727174764
peptidyl-lysine deglycation	1	0.006777831	0.024060717	3.727174764
protein deglycation, glyoxal removal	1	0.006777831	0.024060717	3.727174764
protein deglycation, methylglyoxal removal	1	0.006777831	0.024060717	3.727174764
glutathione deglycation	1	0.006777831	0.024060717	3.727174764
glycolate biosynthetic process	1	0.006777831	0.024060717	3.727174764
detoxification of mercury ion	1	0.006777831	0.024060717	3.727174764
negative regulation of death-inducing signaling complex assembly	1	0.006777831	0.024060717	3.727174764
negative regulation of TRAIL-activated apoptotic signaling pathway	1	0.006777831	0.024060717	3.727174764
positive regulation of pyrroline-5-carboxylate reductase activity	1	0.006777831	0.024060717	3.727174764
positive regulation of tyrosine 3-monooxygenase activity	1	0.006777831	0.024060717	3.727174764
positive regulation of dopamine biosynthetic process	1	0.006777831	0.024060717	3.727174764
glyoxal metabolic process	1	0.006777831	0.024060717	3.727174764
glyoxal catabolic process	1	0.006777831	0.024060717	3.727174764
positive regulation of L-dopa biosynthetic process	1	0.006777831	0.024060717	3.727174764
positive regulation of L-dopa decarboxylase activity	1	0.006777831	0.024060717	3.727174764
positive regulation of oxidative phosphorylation uncoupler activity	1	0.006777831	0.024060717	3.727174764
response to vitamin B2	1	0.006777831	0.024060717	3.727174764
heterochromatin maintenance	1	0.006777831	0.024060717	3.727174764
negative regulation of G-protein coupled receptor internalization	1	0.006777831	0.024060717	3.727174764
positive regulation of histone H3-K27 acetylation	1	0.006777831	0.024060717	3.727174764
spindle disassembly	1	0.006777831	0.024060717	3.727174764
regulation of DNA-templated transcription, termination	1	0.006777831	0.024060717	3.727174764
termination of RNA polymerase II transcription, poly(A)-coupled	1	0.006777831	0.024060717	3.727174764
regulation of termination of RNA polymerase II transcription	1	0.006777831	0.024060717	3.727174764
positive regulation of termination of RNA polymerase II transcription	1	0.006777831	0.024060717	3.727174764
regulation of termination of RNA polymerase II transcription, poly(A)-coupled	1	0.006777831	0.024060717	3.727174764
prostate induction	1	0.006777831	0.024060717	3.727174764
prostate field specification	1	0.006777831	0.024060717	3.727174764
flavin-containing compound catabolic process	1	0.006777831	0.024060717	3.727174764
regulation of protein K63-linked deubiquitination	1	0.006777831	0.024060717	3.727174764
erythrocyte apoptotic process	1	0.006777831	0.024060717	3.727174764
regulation of erythrocyte apoptotic process	1	0.006777831	0.024060717	3.727174764
positive regulation of guanyl-nucleotide exchange factor activity	1	0.006777831	0.024060717	3.727174764
D-alanine family amino acid metabolic process	1	0.006777831	0.024060717	3.727174764
D-alanine metabolic process	1	0.006777831	0.024060717	3.727174764
age-dependent response to oxidative stress	1	0.006777831	0.024060717	3.727174764
age-dependent general metabolic decline	1	0.006777831	0.024060717	3.727174764
regulation of systemic arterial blood pressure by acetylcholine	1	0.006777831	0.024060717	3.727174764
regulation of systemic arterial blood pressure by neurotransmitter	1	0.006777831	0.024060717	3.727174764

regulation of antigen processing and presentation of endogenous peptide antigen via MHC class I	1	0.006777831	0.024060717	3.727174764
phosphagen metabolic process	1	0.006777831	0.024060717	3.727174764
phosphocreatine metabolic process	1	0.006777831	0.024060717	3.727174764
phosphagen biosynthetic process	1	0.006777831	0.024060717	3.727174764
regulation of connective tissue growth factor production	1	0.006777831	0.024060717	3.727174764
regulation of lung goblet cell differentiation	1	0.006777831	0.024060717	3.727174764
regulation of protein kinase D signaling	1	0.006777831	0.024060717	3.727174764
regulation of cellular organohalogen metabolic process	1	0.006777831	0.024060717	3.727174764
regulation of cellular organofluorine metabolic process	1	0.006777831	0.024060717	3.727174764
negative regulation of cellular organohalogen metabolic process	1	0.006777831	0.024060717	3.727174764
protein deglycation	1	0.006777831	0.024060717	3.727174764
regulation of death-inducing signaling complex assembly	1	0.006777831	0.024060717	3.727174764
regulation of pyrroline-5-carboxylate reductase activity	1	0.006777831	0.024060717	3.727174764
regulation of tyrosine 3-monooxygenase activity	1	0.006777831	0.024060717	3.727174764
regulation of dopamine biosynthetic process	1	0.006777831	0.024060717	3.727174764
L-dopa metabolic process	1	0.006777831	0.024060717	3.727174764
L-dopa biosynthetic process	1	0.006777831	0.024060717	3.727174764
regulation of L-dopa biosynthetic process	1	0.006777831	0.024060717	3.727174764
regulation of L-dopa decarboxylase activity	1	0.006777831	0.024060717	3.727174764
regulation of G-protein coupled receptor internalization	1	0.006777831	0.024060717	3.727174764
regulation of morphogenesis of an epithelium	5	0.00679054	0.024064415	3.727021076
negative regulation of homeostatic process	5	0.00679054	0.024064415	3.727021076
negative regulation of autophagy	3	0.006800652	0.024079562	3.726391838
positive regulation of catalytic activity	19	0.006836766	0.024186672	3.721953561
axon extension	4	0.006873136	0.024273703	3.718361693
regulation of striated muscle tissue development	4	0.006873136	0.024273703	3.718361693
cell projection morphogenesis	12	0.007054618	0.024893328	3.69315547
smooth muscle cell migration	3	0.007136669	0.025161332	3.682446923
negative regulation of transcription, DNA-templated	15	0.007200731	0.025223543	3.679977473
response to muscle stretch	2	0.007221577	0.025223543	3.679977473
regulation of microtubule-based movement	2	0.007221577	0.025223543	3.679977473
L-glutamate transport	2	0.007221577	0.025223543	3.679977473
decidualization	2	0.007221577	0.025223543	3.679977473
positive regulation of calcium ion-dependent exocytosis	2	0.007221577	0.025223543	3.679977473
negative regulation of smooth muscle cell migration	2	0.007221577	0.025223543	3.679977473
growth involved in symbiotic interaction	2	0.007221577	0.025223543	3.679977473
growth of symbiont involved in interaction with host	2	0.007221577	0.025223543	3.679977473
regulation of macrophage differentiation	2	0.007221577	0.025223543	3.679977473
positive regulation of protein deacetylation	2	0.007221577	0.025223543	3.679977473
positive regulation of cysteine-type endopeptidase activity involved in apoptotic process	4	0.007313685	0.025502071	3.668995614
regulation of muscle organ development	4	0.007313685	0.025502071	3.668995614
positive regulation of cyclase activity	3	0.007482381	0.026024303	3.64872444
positive regulation of lyase activity	3	0.007482381	0.026024303	3.64872444
positive regulation of tyrosine phosphorylation of STAT protein	3	0.007482381	0.026024303	3.64872444

regulation of muscle tissue development	4	0.007540787	0.026183287	3.642633968
positive regulation of hormone secretion	4	0.007540787	0.026183287	3.642633968
regulation of myeloid cell differentiation	5	0.007800702	0.027062992	3.609588104
regulation of protein acetylation	3	0.007837855	0.027169036	3.605677337
positive regulation of cellular protein catabolic process	5	0.007978685	0.027529871	3.592483628
apoptotic DNA fragmentation	2	0.007988668	0.027529871	3.592483628
regulation of protein homodimerization activity	2	0.007988668	0.027529871	3.592483628
neuronal stem cell population maintenance	2	0.007988668	0.027529871	3.592483628
vitamin D metabolic process	2	0.007988668	0.027529871	3.592483628
regulation of neuron projection regeneration	2	0.007988668	0.027529871	3.592483628
positive regulation of lymphocyte apoptotic process	2	0.007988668	0.027529871	3.592483628
positive regulation of transmembrane transport	4	0.008008835	0.027576332	3.590797393
purine nucleotide metabolic process	9	0.008046322	0.027682301	3.58696203
negative regulation of gene expression	18	0.008114543	0.027893741	3.579352954
protein acetylation	5	0.008159476	0.028024844	3.574663873
regulation of calcium ion-dependent exocytosis	3	0.008203152	0.028034798	3.574308769
positive regulation of striated muscle tissue development	3	0.008203152	0.028034798	3.574308769
positive regulation of muscle organ development	3	0.008203152	0.028034798	3.574308769
regulation of neurotransmitter transport	3	0.008203152	0.028034798	3.574308769
regulation of intracellular steroid hormone receptor signaling pathway	3	0.008203152	0.028034798	3.574308769
regulation of cellular response to insulin stimulus	3	0.008203152	0.028034798	3.574308769
protein homooligomerization	6	0.008262595	0.028214573	3.567916672
regulation of cellular component size	7	0.00843677	0.028785506	3.547883275
negative regulation of developmental process	12	0.008454875	0.028823438	3.546566405
negative regulation of neuron differentiation	5	0.008529567	0.029054057	3.538597147
astrocyte differentiation	3	0.00857833	0.02917198	3.534546629
positive regulation of muscle tissue development	3	0.00857833	0.02917198	3.534546629
regulation of epithelial cell differentiation	4	0.008746079	0.029550063	3.521669415
regulation of receptor activity	4	0.008746079	0.029550063	3.521669415
organophosphate catabolic process	4	0.008746079	0.029550063	3.521669415
single organismal cell-cell adhesion	11	0.008755736	0.029550063	3.521669415
cytokine production	10	0.008758714	0.029550063	3.521669415
response to copper ion	2	0.00879075	0.029550063	3.521669415
endoplasmic reticulum calcium ion homeostasis	2	0.00879075	0.029550063	3.521669415
positive regulation of protein oligomerization	2	0.00879075	0.029550063	3.521669415
positive regulation of peptidyl-serine phosphorylation of STAT protein	2	0.00879075	0.029550063	3.521669415
axonal fasciculation	2	0.00879075	0.029550063	3.521669415
response to corticosterone	2	0.00879075	0.029550063	3.521669415
positive regulation of focal adhesion assembly	2	0.00879075	0.029550063	3.521669415
acidic amino acid transport	2	0.00879075	0.029550063	3.521669415
mitochondrial depolarization	2	0.00879075	0.029550063	3.521669415
histone modification	8	0.008796964	0.029550063	3.521669415
kidney development	6	0.008875919	0.029791023	3.513548179
inorganic ion transmembrane transport	11	0.008931843	0.029954352	3.508080658

developmental process involved in reproduction	10	0.008950062	0.029987201	3.506984634
regulation of mitochondrial membrane permeability	3	0.008963447	0.029987201	3.506984634
nitric oxide biosynthetic process	3	0.008963447	0.029987201	3.506984634
positive regulation of cell morphogenesis involved in differentiation	4	0.009261444	0.030959042	3.475090184
JNK cascade	5	0.009304388	0.031052264	3.47208355
epithelial cell development	5	0.009304388	0.031052264	3.47208355
positive regulation of phosphatidylinositol 3-kinase signaling	3	0.009358556	0.031182589	3.467895397
response to osmotic stress	3	0.009358556	0.031182589	3.467895397
organelle fusion	5	0.009505433	0.031646416	3.453130365
negative regulation of neuron projection development	4	0.009526394	0.031690624	3.451734418
lymphocyte activation	10	0.009543477	0.031719413	3.450826403
negative regulation of myoblast differentiation	2	0.009627322	0.031719413	3.450826403
negative regulation of lipid catabolic process	2	0.009627322	0.031719413	3.450826403
embryonic hemopoiesis	2	0.009627322	0.031719413	3.450826403
negative regulation of cytokine production involved in immune response	2	0.009627322	0.031719413	3.450826403
vitamin biosynthetic process	2	0.009627322	0.031719413	3.450826403
myelination in peripheral nervous system	2	0.009627322	0.031719413	3.450826403
peripheral nervous system axon ensheathment	2	0.009627322	0.031719413	3.450826403
regulation of nuclease activity	2	0.009627322	0.031719413	3.450826403
regulation of cellular respiration	2	0.009627322	0.031719413	3.450826403
regulation of peptidyl-serine phosphorylation of STAT protein	2	0.009627322	0.031719413	3.450826403
positive regulation of adherens junction organization	2	0.009627322	0.031719413	3.450826403
T cell activation	8	0.009643078	0.03172065	3.450787399
T cell aggregation	8	0.009643078	0.03172065	3.450787399
regulation of cell adhesion	10	0.009747854	0.032031856	3.441024357
vasodilation	3	0.00976371	0.032031856	3.441024357
nucleotide catabolic process	3	0.00976371	0.032031856	3.441024357
lymphocyte aggregation	8	0.009768746	0.032031856	3.441024357
response to UV	4	0.009796233	0.032096476	3.43900905
cell junction assembly	5	0.009916477	0.032464658	3.42760324
response to mechanical stimulus	5	0.010126518	0.033126	3.407436795
skeletal muscle cell differentiation	3	0.010178957	0.033244811	3.403856598
positive regulation of insulin secretion	3	0.010178957	0.033244811	3.403856598
regulation of calcium ion transport	5	0.010339598	0.033716081	3.389780367
positive regulation of DNA metabolic process	5	0.010339598	0.033716081	3.389780367
epidermal growth factor receptor signaling pathway	4	0.010350719	0.033725684	3.389495585
establishment of protein localization to membrane	7	0.010390484	0.033828529	3.386450793
chemotaxis	9	0.010425261	0.033910563	3.384028709
positive regulation of erythrocyte differentiation	2	0.010497888	0.033910563	3.384028709
misfolded or incompletely synthesized protein catabolic process	2	0.010497888	0.033910563	3.384028709
mitochondrial fusion	2	0.010497888	0.033910563	3.384028709
branching involved in salivary gland morphogenesis	2	0.010497888	0.033910563	3.384028709
regulation of execution phase of apoptosis	2	0.010497888	0.033910563	3.384028709
positive regulation of transcription from RNA polymerase II promoter in response to stress	2	0.010497888	0.033910563	3.384028709

branching involved in mammary gland duct morphogenesis	2	0.010497888	0.033910563	3.384028709
positive regulation of nucleoside metabolic process	2	0.010497888	0.033910563	3.384028709
positive regulation of ATP metabolic process	2	0.010497888	0.033910563	3.384028709
anatomical structure homeostasis	7	0.010541656	0.033982159	3.381919614
sulfur compound metabolic process	7	0.010541656	0.033982159	3.381919614
taxis	9	0.010544767	0.033982159	3.381919614
cellular response to drug	3	0.010604343	0.034041179	3.380184332
positive regulation of axonogenesis	3	0.010604343	0.034041179	3.380184332
regulation of membrane permeability	3	0.010604343	0.034041179	3.380184332
cellular response to ketone	3	0.010604343	0.034041179	3.380184332
regulation of tyrosine phosphorylation of STAT protein	3	0.010604343	0.034041179	3.380184332
regulation of lipid biosynthetic process	4	0.010635431	0.034061455	3.379588886
positive regulation of nucleotide metabolic process	4	0.010635431	0.034061455	3.379588886
positive regulation of purine nucleotide metabolic process	4	0.010635431	0.034061455	3.379588886
leukocyte aggregation	8	0.010682969	0.034187157	3.375905221
steroid metabolic process	6	0.010730337	0.034312125	3.37225648
neural precursor cell proliferation	4	0.010925166	0.034908064	3.355037409
respiratory system development	5	0.010997279	0.035111283	3.34923275
purine-containing compound metabolic process	9	0.011033168	0.035138615	3.348454621
nitric oxide metabolic process	3	0.011039914	0.035138615	3.348454621
muscle cell migration	3	0.011039914	0.035138615	3.348454621
lymphocyte apoptotic process	3	0.011039914	0.035138615	3.348454621
proteolysis involved in cellular protein catabolic process	10	0.011159341	0.03549135	3.338466282
fat cell differentiation	5	0.01122272	0.035665424	3.333573582
renal system development	6	0.0112805	0.035821448	3.32920846
superoxide anion generation	2	0.011401958	0.036013074	3.323873228
negative regulation of peptidyl-serine phosphorylation	2	0.011401958	0.036013074	3.323873228
negative regulation of proteasomal ubiquitin-dependent protein catabolic process	2	0.011401958	0.036013074	3.323873228
positive regulation of translational initiation	2	0.011401958	0.036013074	3.323873228
regulation of histone deacetylation	2	0.011401958	0.036013074	3.323873228
DNA catabolic process, endonucleolytic	2	0.011401958	0.036013074	3.323873228
vascular endothelial growth factor signaling pathway	2	0.011401958	0.036013074	3.323873228
phosphatidylinositol-mediated signaling	5	0.0114513	0.036086031	3.321849446
positive regulation of homeostatic process	5	0.0114513	0.036086031	3.321849446
regulation of purine nucleotide metabolic process	5	0.0114513	0.036086031	3.321849446
sprouting angiogenesis	3	0.011485711	0.036111704	3.321138243
glutamate receptor signaling pathway	3	0.011485711	0.036111704	3.321138243
peripheral nervous system development	3	0.011485711	0.036111704	3.321138243
positive regulation of intracellular transport	6	0.011658204	0.036626117	3.306993718
striated muscle cell development	4	0.011824825	0.037121311	3.293564062
cellular response to mechanical stimulus	3	0.011941773	0.037431469	3.285243499
multicellular organismal response to stress	3	0.011941773	0.037431469	3.285243499
positive regulation of NF-kappaB transcription factor activity	4	0.012134965	0.03797931	3.270713735
vitamin metabolic process	4	0.012134965	0.03797931	3.270713735

inositol lipid-mediated signaling	5	0.012156076	0.038016539	3.269733969
regulation of astrocyte differentiation	2	0.012339044	0.038327227	3.26159474
beta-amyloid metabolic process	2	0.012339044	0.038327227	3.26159474
Schwann cell development	2	0.012339044	0.038327227	3.26159474
positive regulation of G-protein coupled receptor protein signaling pathway	2	0.012339044	0.038327227	3.26159474
inorganic cation import into cell	2	0.012339044	0.038327227	3.26159474
inorganic ion import into cell	2	0.012339044	0.038327227	3.26159474
positive regulation of tissue remodeling	2	0.012339044	0.038327227	3.26159474
apoptotic process involved in morphogenesis	2	0.012339044	0.038327227	3.26159474
regulation of vascular endothelial growth factor receptor signaling pathway	2	0.012339044	0.038327227	3.26159474
animal organ regeneration	3	0.012408138	0.038397278	3.259768716
reactive nitrogen species metabolic process	3	0.012408138	0.038397278	3.259768716
regulation of cell junction assembly	3	0.012408138	0.038397278	3.259768716
regulation of release of sequestered calcium ion into cytosol	3	0.012408138	0.038397278	3.259768716
tyrosine phosphorylation of STAT protein	3	0.012408138	0.038397278	3.259768716
acute inflammatory response	4	0.012450283	0.038412428	3.259374234
regulation of blood vessel size	4	0.012450283	0.038412428	3.259374234
regulation of cell division	4	0.012450283	0.038412428	3.259374234
regulation of G1/S transition of mitotic cell cycle	4	0.012450283	0.038412428	3.259374234
regulation of organelle assembly	4	0.012770807	0.038754711	3.250502966
regulation of tube size	4	0.012770807	0.038754711	3.250502966
alpha-amino acid catabolic process	3	0.012884842	0.038754711	3.250502966
developmental growth involved in morphogenesis	5	0.012889809	0.038754711	3.250502966
neuron migration	4	0.013096567	0.038754711	3.250502966
positive regulation of binding	4	0.013096567	0.038754711	3.250502966
regulation of nucleotide metabolic process	5	0.01314091	0.038754711	3.250502966
negative regulation of cellular macromolecule biosynthetic process	16	0.013146932	0.038754711	3.250502966
receptor-mediated endocytosis	6	0.013258717	0.038754711	3.250502966
regulation of p38MAPK cascade	2	0.013308668	0.038754711	3.250502966
response to arsenic-containing substance	2	0.013308668	0.038754711	3.250502966
cell differentiation involved in embryonic placenta development	2	0.013308668	0.038754711	3.250502966
glutamine family amino acid catabolic process	2	0.013308668	0.038754711	3.250502966
peptidyl-cysteine modification	2	0.013308668	0.038754711	3.250502966
neurotransmitter uptake	2	0.013308668	0.038754711	3.250502966
intrinsic apoptotic signaling pathway by p53 class mediator	3	0.013371916	0.038754711	3.250502966
regulation of neural precursor cell proliferation	3	0.013371916	0.038754711	3.250502966
positive regulation of protein binding	3	0.013371916	0.038754711	3.250502966
response to temperature stimulus	5	0.013395303	0.038754711	3.250502966
protein tetramerization	4	0.01342759	0.038754711	3.250502966
myoblast differentiation involved in skeletal muscle regeneration	1	0.013510127	0.038754711	3.250502966
calcium-dependent cell-matrix adhesion	1	0.013510127	0.038754711	3.250502966
positive regulation of chronic inflammatory response to antigenic stimulus	1	0.013510127	0.038754711	3.250502966
positive regulation of translational initiation by iron	1	0.013510127	0.038754711	3.250502966
positive regulation of blood microparticle formation	1	0.013510127	0.038754711	3.250502966

response to antipsychotic drug	1	0.013510127	0.038754711	3.250502966
smooth endoplasmic reticulum calcium ion homeostasis	1	0.013510127	0.038754711	3.250502966
positive regulation of dense core granule biogenesis	1	0.013510127	0.038754711	3.250502966
cellular response to cell-matrix adhesion	1	0.013510127	0.038754711	3.250502966
response to insulin-like growth factor stimulus	1	0.013510127	0.038754711	3.250502966
negative regulation of adrenergic receptor signaling pathway involved in heart process	1	0.013510127	0.038754711	3.250502966
calcium ion import across plasma membrane	1	0.013510127	0.038754711	3.250502966
response to platinum ion	1	0.013510127	0.038754711	3.250502966
negative regulation of integrin biosynthetic process	1	0.013510127	0.038754711	3.250502966
lateral sprouting involved in mammary gland duct morphogenesis	1	0.013510127	0.038754711	3.250502966
tertiary branching involved in mammary gland duct morphogenesis	1	0.013510127	0.038754711	3.250502966
inorganic diphosphate transport	1	0.013510127	0.038754711	3.250502966
positive regulation of ubiquitin-specific protease activity	1	0.013510127	0.038754711	3.250502966
B cell negative selection	1	0.013510127	0.038754711	3.250502966
B cell homeostatic proliferation	1	0.013510127	0.038754711	3.250502966
fasciculation of motor neuron axon	1	0.013510127	0.038754711	3.250502966
nuclear fragmentation involved in apoptotic nuclear change	1	0.013510127	0.038754711	3.250502966
D-serine metabolic process	1	0.013510127	0.038754711	3.250502966
base-excision repair, base-free sugar-phosphate removal	1	0.013510127	0.038754711	3.250502966
positive regulation of cardiac muscle hypertrophy in response to stress	1	0.013510127	0.038754711	3.250502966
positive regulation of ferrous iron import into cell	1	0.013510127	0.038754711	3.250502966
positive regulation of ferrous iron binding	1	0.013510127	0.038754711	3.250502966
positive regulation of transferrin receptor binding	1	0.013510127	0.038754711	3.250502966
response to iron ion starvation	1	0.013510127	0.038754711	3.250502966
negative regulation of CD8-positive, alpha-beta T cell activation	1	0.013510127	0.038754711	3.250502966
low-density lipoprotein particle mediated signaling	1	0.013510127	0.038754711	3.250502966
intermediate filament polymerization or depolymerization	1	0.013510127	0.038754711	3.250502966
response to sodium arsenite	1	0.013510127	0.038754711	3.250502966
response to acrylamide	1	0.013510127	0.038754711	3.250502966
positive regulation of tyrosine phosphorylation of Stat6 protein	1	0.013510127	0.038754711	3.250502966
coronary vein morphogenesis	1	0.013510127	0.038754711	3.250502966
VEGF-activated neuropilin signaling pathway	1	0.013510127	0.038754711	3.250502966
positive regulation of retinal ganglion cell axon guidance	1	0.013510127	0.038754711	3.250502966
positive regulation of mitochondrial electron transport, NADH to ubiquinone	1	0.013510127	0.038754711	3.250502966
negative regulation of protein K48-linked deubiquitination	1	0.013510127	0.038754711	3.250502966
negative regulation of hydrogen peroxide-induced neuron intrinsic apoptotic signaling pathway	1	0.013510127	0.038754711	3.250502966
negative regulation of nitrosative stress-induced intrinsic apoptotic signaling pathway	1	0.013510127	0.038754711	3.250502966
negative regulation of ubiquitin-specific protease activity	1	0.013510127	0.038754711	3.250502966
positive regulation of androgen receptor activity	1	0.013510127	0.038754711	3.250502966
leukocyte adhesion to arterial endothelial cell	1	0.013510127	0.038754711	3.250502966
regulation of leukocyte adhesion to arterial endothelial cell	1	0.013510127	0.038754711	3.250502966
blood microparticle formation	1	0.013510127	0.038754711	3.250502966
regulation of blood microparticle formation	1	0.013510127	0.038754711	3.250502966
regulation of calcium-dependent cell-cell adhesion	1	0.013510127	0.038754711	3.250502966

regulation of adrenergic receptor signaling pathway involved in heart process	1	0.013510127	0.038754711	3.250502966
somatic sex determination	1	0.013510127	0.038754711	3.250502966
myofibroblast differentiation	1	0.013510127	0.038754711	3.250502966
regulation of myofibroblast differentiation	1	0.013510127	0.038754711	3.250502966
flavin adenine dinucleotide metabolic process	1	0.013510127	0.038754711	3.250502966
B cell selection	1	0.013510127	0.038754711	3.250502966
regulation of Rho guanyl-nucleotide exchange factor activity	1	0.013510127	0.038754711	3.250502966
positive regulation of cardiac muscle adaptation	1	0.013510127	0.038754711	3.250502966
regulation of T cell antigen processing and presentation	1	0.013510127	0.038754711	3.250502966
regulation of iron ion transport	1	0.013510127	0.038754711	3.250502966
positive regulation of iron ion transport	1	0.013510127	0.038754711	3.250502966
regulation of iron ion import	1	0.013510127	0.038754711	3.250502966
regulation of ferrous iron import into cell	1	0.013510127	0.038754711	3.250502966
negative regulation of antigen processing and presentation of peptide antigen via MHC class I	1	0.013510127	0.038754711	3.250502966
regulation of ferrous iron binding	1	0.013510127	0.038754711	3.250502966
regulation of transferrin receptor binding	1	0.013510127	0.038754711	3.250502966
lipoprotein particle mediated signaling	1	0.013510127	0.038754711	3.250502966
connective tissue growth factor production	1	0.013510127	0.038754711	3.250502966
regulation of lung ciliated cell differentiation	1	0.013510127	0.038754711	3.250502966
pancreatic stellate cell proliferation	1	0.013510127	0.038754711	3.250502966
regulation of pancreatic stellate cell proliferation	1	0.013510127	0.038754711	3.250502966
neuropilin signaling pathway	1	0.013510127	0.038754711	3.250502966
cellular organohalogen metabolic process	1	0.013510127	0.038754711	3.250502966
cellular organofluorine metabolic process	1	0.013510127	0.038754711	3.250502966
enzyme active site formation	1	0.013510127	0.038754711	3.250502966
positive regulation of mitochondrial ATP synthesis coupled electron transport	1	0.013510127	0.038754711	3.250502966
regulation of protein K48-linked deubiquitination	1	0.013510127	0.038754711	3.250502966
neuron intrinsic apoptotic signaling pathway in response to hydrogen peroxide	1	0.013510127	0.038754711	3.250502966
regulation of hydrogen peroxide-induced neuron intrinsic apoptotic signaling pathway	1	0.013510127	0.038754711	3.250502966
regulation of nitrosative stress-induced intrinsic apoptotic signaling pathway	1	0.013510127	0.038754711	3.250502966
intrinsic apoptotic signaling pathway in response to nitrosative stress	1	0.013510127	0.038754711	3.250502966
chromatin maintenance	1	0.013510127	0.038754711	3.250502966
gonad development	5	0.013653008	0.039137357	3.240677855
nucleobase-containing compound metabolic process	50	0.01368392	0.039198729	3.239110954
adult behavior	4	0.013763906	0.039400494	3.233976925
regulation of angiogenesis	5	0.014178423	0.040558942	3.204998994
regulation of glycogen biosynthetic process	2	0.014310351	0.040794886	3.199198553
regulation of protein localization to cell surface	2	0.014310351	0.040794886	3.199198553
regulation of glucan biosynthetic process	2	0.014310351	0.040794886	3.199198553
serine phosphorylation of STAT protein	2	0.014310351	0.040794886	3.199198553
positive regulation of cell junction assembly	2	0.014310351	0.040794886	3.199198553
myoblast differentiation	3	0.014377299	0.040900937	3.19660231
negative regulation of lipid metabolic process	3	0.014377299	0.040900937	3.19660231
regulation of steroid metabolic process	3	0.014377299	0.040900937	3.19660231

canonical Wnt signaling pathway	6	0.014556145	0.041381184	3.184929
ubiquitin-dependent protein catabolic process	9	0.014634281	0.041574662	3.18026439
cell redox homeostasis	3	0.014895662	0.042200966	3.165312165
endoderm development	3	0.014895662	0.042200966	3.165312165
positive regulation of JAK-STAT cascade	3	0.014895662	0.042200966	3.165312165
positive regulation of STAT cascade	3	0.014895662	0.042200966	3.165312165
development of primary sexual characteristics	5	0.014991831	0.042444271	3.159563325
organ growth	4	0.015162626	0.042898376	3.148921319
regulation of inflammatory response	6	0.015237008	0.043079271	3.144713354
protein acylation	5	0.01526978	0.04311475	3.143890124
necroptotic process	2	0.015343625	0.04311475	3.143890124
positive regulation of protein insertion into mitochondrial membrane involved in apoptotic signaling pathway	2	0.015343625	0.04311475	3.143890124
positive regulation of histone acetylation	2	0.015343625	0.04311475	3.143890124
calcium-dependent cell-cell adhesion via plasma membrane cell adhesion molecules	2	0.015343625	0.04311475	3.143890124
regulation of cGMP metabolic process	2	0.015343625	0.04311475	3.143890124
positive regulation of cardiac muscle tissue growth	2	0.015343625	0.04311475	3.143890124
positive regulation of leukocyte apoptotic process	2	0.015343625	0.04311475	3.143890124
regulation of protein insertion into mitochondrial membrane involved in apoptotic signaling pathway	2	0.015343625	0.04311475	3.143890124
peptidyl-threonine phosphorylation	3	0.015424507	0.043312519	3.139313574
regulation of phosphatidylinositol 3-kinase signaling	4	0.015525801	0.043567298	3.133448454
cellular protein catabolic process	10	0.015588013	0.043712136	3.1301295
maintenance of location	6	0.015703047	0.044004801	3.123456527
regulation of cell cycle G1/S phase transition	4	0.015894424	0.044497178	3.11232951
modification-dependent protein catabolic process	9	0.015900325	0.044497178	3.11232951
glial cell development	3	0.015963855	0.044644678	3.109020171
protein localization to membrane	8	0.016051441	0.04485921	3.104226363
activation of MAPK activity	4	0.01626852	0.045435102	3.091470303
adult walking behavior	2	0.016408021	0.045508352	3.089859403
negative regulation of cation channel activity	2	0.016408021	0.045508352	3.089859403
response to amphetamine	2	0.016408021	0.045508352	3.089859403
regulation of myelination	2	0.016408021	0.045508352	3.089859403
epithelial cell morphogenesis	2	0.016408021	0.045508352	3.089859403
energy homeostasis	2	0.016408021	0.045508352	3.089859403
multicellular organism aging	2	0.016408021	0.045508352	3.089859403
negative regulation of production of molecular mediator of immune response	2	0.016408021	0.045508352	3.089859403
walking behavior	2	0.016408021	0.045508352	3.089859403
positive regulation of peptidyl-lysine acetylation	2	0.016408021	0.045508352	3.089859403
cell-cell junction organization	5	0.016416104	0.045508352	3.089859403
autophagosome assembly	3	0.016513727	0.045717532	3.085273431
adult locomotory behavior	3	0.016513727	0.045717532	3.085273431
negative regulation of cell projection organization	4	0.016648113	0.045996962	3.079179921
regulation of JAK-STAT cascade	4	0.016648113	0.045996962	3.079179921
regulation of STAT cascade	4	0.016648113	0.045996962	3.079179921
muscle cell development	4	0.017033225	0.047029487	3.056980496

regulation of organ growth	3	0.017074141	0.047110924	3.05525037
blood circulation	8	0.017171488	0.047347852	3.050233831
modification-dependent macromolecule catabolic process	9	0.017245501	0.047520167	3.046601084
protein transport	20	0.017355549	0.047791483	3.040907841
ERBB signaling pathway	4	0.01742388	0.04791567	3.038312689
calcium-mediated signaling	4	0.01742388	0.04791567	3.038312689
cellular response to epidermal growth factor stimulus	2	0.01750308	0.047941702	3.03776955
response to iron ion	2	0.01750308	0.047941702	3.03776955
negative regulation of DNA biosynthetic process	2	0.01750308	0.047941702	3.03776955
calcium ion-regulated exocytosis of neurotransmitter	2	0.01750308	0.047941702	3.03776955
regulation of intracellular estrogen receptor signaling pathway	2	0.01750308	0.047941702	3.03776955
apoptotic process involved in development	2	0.01750308	0.047941702	3.03776955
regulation of JUN kinase activity	3	0.017645114	0.048202713	3.032339972
autophagosome organization	3	0.017645114	0.048202713	3.032339972
peptidyl-threonine modification	3	0.017645114	0.048202713	3.032339972
cellular amino acid catabolic process	3	0.017645114	0.048202713	3.032339972
circulatory system process	8	0.017948932	0.049000228	3.01593032
Ras protein signal transduction	6	0.018182368	0.049604674	3.003670211
positive regulation of cell division	3	0.018226661	0.049659827	3.002558985
regulation of carbohydrate biosynthetic process	3	0.018226661	0.049659827	3.002558985
