# Supplementary material for: Molecular Taxonomy of Sporadic Amyotrophic Lateral Sclerosis Using Disease-Associated Genes
Source: Front Neurol. 2017 Apr 19;8:152. doi: 10.3389/fneur.2017.00152 (PMC5395696; doi:10.3389/fneur.2017.00152)
Supplement: Supplementary file 7 [file Table_7.PDF]

Supplementary Table 7. List of top 50 significantly enriched GO biological processes for 100 randomly selected SGALS (subset n.1)

GO biological process	SGALS genes in category	p value	fdr p value	-LOG(corr.pValue)
localization	58	5.65939E-12	1.8625E-08	17.79875869
protein phosphorylation	32	2.22E-11	3.65E-08	17.1254255
nervous system development	32	8.79E-10	9.65E-07	13.8515332
neuron projection extension	10	1.75E-09	1.44E-06	13.44990442
phosphorylation	32	2.19E-09	1.44E-06	13.44990442
axon extension	9	3.24E-09	1.64E-06	13.32015215
regulation of protein phosphorylation	24	3.49E-09	1.64E-06	13.32015215
neurogenesis	25	4.86E-09	1.90E-06	13.17257962
regulation of protein modification process	27	5.20E-09	1.90E-06	13.17257962
programmed cell death	28	8.47E-09	2.79E-06	12.79026029
cell surface receptor signaling pathway	34	1.09E-08	3.26E-06	12.6347215
regulation of phosphorylation	24	1.35E-08	3.70E-06	12.50819085
movement of cell or subcellular component	27	2.22E-08	5.37E-06	12.13398544
axon development	14	2.29E-08	5.37E-06	12.13398544
developmental cell growth	10	2.46E-08	5.39E-06	12.13108578
cell death	28	3.04E-08	6.09E-06	12.00906788
phosphate-containing compound metabolic proces	36	3.33E-08	6.09E-06	12.00906788
generation of neurons	23	3.38E-08	6.09E-06	12.00906788
phosphorus metabolic process	36	3.52E-08	6.09E-06	12.00906788
positive regulation of response to stimulus	28	4.57E-08	7.52E-06	11.79740166
system development	43	5.40E-08	7.55E-06	11.79440218
developmental growth involved in morphogenesis	10	5.58E-08	7.55E-06	11.79440218
regulation of phosphate metabolic process	25	5.89E-08	7.55E-06	11.79440218
positive regulation of phosphorus metabolic proces	20	5.91E-08	7.55E-06	11.79440218
positive regulation of phosphate metabolic process	20	5.91E-08	7.55E-06	11.79440218
regulation of phosphorus metabolic process	25	5.96E-08	7.55E-06	11.79440218
axonogenesis	13	6.91E-08	8.42E-06	11.68431702
neuron development	19	7.79E-08	8.74E-06	11.64719295
regulation of axonogenesis	9	7.97E-08	8.74E-06	11.64719295
multicellular organism development	46	7.97E-08	8.74E-06	11.64719295
positive regulation of cell communication	24	9.13E-08	9.69E-06	11.54404743

cell morphogenesis involved in neuron differentiati	14	9.66E-08	9.76E-06	11.53679254
positive regulation of signaling	24	9.97E-08	9.76E-06	11.53679254
MAPK cascade	18	1.01E-07	9.76E-06	11.53679254
regulation of anatomical structure morphogenesis	19	1.12E-07	1.03E-05	11.48104212
positive regulation of neuron projection developme	10	1.13E-07	1.03E-05	11.48104212
apoptotic process	26	1.26E-07	1.09E-05	11.42783011
regulation of cell development	17	1.28E-07	1.09E-05	11.42783011
establishment of localization	45	1.33E-07	1.09E-05	11.42783011
regulation of cellular protein metabolic process	30	1.34E-07	1.09E-05	11.42783011
positive regulation of protein phosphorylation	18	1.36E-07	1.09E-05	11.42783011
cell migration	21	1.54E-07	1.20E-05	11.32754692
macromolecule modification	41	1.64E-07	1.24E-05	11.29501131
positive regulation of endothelial cell chemotaxis	4	1.66E-07	1.24E-05	11.29501131
cell morphogenesis	20	1.82E-07	1.31E-05	11.24176652
transport	44	1.85E-07	1.31E-05	11.24176652
signal transduction by protein phosphorylation	18	1.87E-07	1.31E-05	11.24176652
regulation of axon extension	7	1.94E-07	1.33E-05	11.22947049
single-organism developmental process	50	2.02E-07	1.36E-05	11.20586577
neuron projection development	17	2.09E-07	1.37E-05	11.1946159
