# Supplementary material for: Molecular Taxonomy of Sporadic Amyotrophic Lateral Sclerosis Using Disease-Associated Genes
Source: Front Neurol. 2017 Apr 19;8:152. doi: 10.3389/fneur.2017.00152 (PMC5395696; doi:10.3389/fneur.2017.00152)
Supplement: Supplementary file 8 [file Table_8.PDF]

Supplementary Table 8. List of top 50 significantly enriched GO biological processes for 100 randomly selected SGALS (subset n.2)

GO biological process	SGALS genes in category	p value	fdr p value	-LOG(corr.pValue)
response to oxidative stress	15	3.16877E-10	9.46196E-07	13.87081635
ionotropic glutamate receptor signaling pathway	5	9.44E-08	8.81E-05	9.337270195
response to organonitrogen compound	17	1.02E-07	8.81E-05	9.337270195
response to abiotic stimulus	20	1.18E-07	8.81E-05	9.337270195
response to inorganic substance	13	2.72E-07	1.59E-04	8.747847173
cellular component organization	51	3.19E-07	1.59E-04	8.747847173
response to nitrogen compound	17	6.40E-07	2.03E-04	8.503331906
cellular component organization or biogenesis	51	7.77E-07	2.03E-04	8.503331906
chemical synaptic transmission	14	8.15E-07	2.03E-04	8.503331906
anterograde trans-synaptic signaling	14	8.15E-07	2.03E-04	8.503331906
synaptic signaling	14	8.15E-07	2.03E-04	8.503331906
trans-synaptic signaling	14	8.15E-07	2.03E-04	8.503331906
glutamate receptor signaling pathway	6	1.02E-06	2.35E-04	8.355377956
response to cadmium ion	5	1.21E-06	2.58E-04	8.263636152
response to growth factor	14	1.44E-06	2.87E-04	8.156558017
response to endogenous stimulus	22	1.74E-06	3.01E-04	8.106938688
response to hydrogen peroxide	7	1.78E-06	3.01E-04	8.106938688
cellular response to hydrogen peroxide	6	1.82E-06	3.01E-04	8.106938688
response to stress	36	2.60E-06	3.97E-04	7.83040527
regulation of cell communication	32	2.66E-06	3.97E-04	7.83040527
localization	48	3.02E-06	4.26E-04	7.76044576
regulation of biological quality	35	3.19E-06	4.26E-04	7.76044576
single-organism cellular localization	18	3.28E-06	4.26E-04	7.76044576
nervous system development	26	3.62E-06	4.50E-04	7.705233305
regulation of signaling	32	3.90E-06	4.66E-04	7.672257707
cellular response to growth factor stimulus	13	5.11E-06	5.72E-04	7.465881762
response to acid chemical	9	5.27E-06	5.72E-04	7.465881762
cell-cell signaling	21	5.63E-06	5.72E-04	7.465881762
cellular response to chemical stimulus	29	5.86E-06	5.72E-04	7.465881762
response to amino acid	6	5.93E-06	5.72E-04	7.465881762
aging	9	5.95E-06	5.72E-04	7.465881762

response to organic substance	30	6.13E-06	5.72E-04	7.465881762
cell death	24	6.57E-06	5.95E-04	7.427104187
positive regulation of multicellular organismal process	20	7.31E-06	6.42E-04	7.351244137
response to oxygen-containing compound	20	9.64E-06	8.23E-04	7.102788746
regulation of developmental process	26	1.08E-05	8.93E-04	7.020872593
cellular response to oxidative stress	8	1.14E-05	9.19E-04	6.992364127
regulation of localization	27	1.19E-05	9.28E-04	6.982741585
regulation of anatomical structure morphogenesis	16	1.21E-05	9.28E-04	6.982741585
transport	40	1.48E-05	1.08E-03	6.831451102
response to oxygen levels	9	1.51E-05	1.08E-03	6.831451102
system development	38	1.52E-05	1.08E-03	6.831451102
single-multicellular organism process	47	1.58E-05	1.10E-03	6.815975707
response to chemical	37	1.68E-05	1.14E-03	6.774781747
regulation of hydrogen peroxide-mediated programmed cell de	3	1.77E-05	1.18E-03	6.745488258
response to drug	10	2.18E-05	1.39E-03	6.580986613
single-organism localization	30	2.18E-05	1.39E-03	6.580986613
neuron projection development	14	2.61E-05	1.62E-03	6.424054708
programmed cell death	22	2.84E-05	1.72E-03	6.366091467
establishment of localization	40	2.98E-05	1.72E-03	6.366091467
